# Hitchhiker’s Guide to the Preparation of Novel Benzimidazoline-Based n‑Type Dopants

**DOI:** 10.1021/acs.chemmater.5c01479

**Published:** 2025-09-15

**Authors:** Francesca Pallini, Giulia Garavaglia, Gabriele Paoli, Giuseppe Mattioli, Francesco Porcelli, Lorenzo Mezzomo, Domenico Antonio Florenzano, Riccardo Ruffo, Pietro Rossi, Mario Caironi, Mauro Sassi, Sara Mattiello

**Affiliations:** † Department of Materials Science, 9305 University of Milano-Bicocca, Via R. Cozzi 55, 20125 Milano, Italy; ‡ 9327 Consiglio nazionale delle ricerche, Istituto di struttura della materia (CNR-ISM), Area della Ricerca di Roma 1, Via Salaria km 29.300, Monterotondo 00015, Italy; § Center for Nano Science and Technology, Istituto Italiano di Tecnologia, Via Rubattino 81, 20131 Milano, Italy

## Abstract

1*H*-Benzimidazoline-based molecular n dopant precursors, such as 4-(2,3-dihydro-1,3-dimethyl-1*H*-benzimidazol-2-yl)-*N*,*N*-dimethylbenzenamine (N-DMBI-H), enable efficient doping of high-electron affinity (EA) organic semiconductors. Chemical modification of the molecular structure of such compounds proved to be a fundamental tool to tune their properties and doping efficiencies according to the desired application. Versatile and efficient synthetic strategies, giving access to the widest range of substitution motifs, are expected to improve access to known derivatives and enable the preparation of new and improved ones. The literature reports several synthetic approaches, but due to a lack of rationalization and a comprehensive analysis, the selection of that best suited for a specific target derivative still mostly relies on a trial and error approach. In this work, we compare the features of the two most popular synthetic strategies in the preparation of a wide variety of benzimidazoline dopants having diverse substitution patterns and electronic features. We thus propose guidelines for the selection of the best synthetic approach depending on the structure of the target dopant, known as well as original.

## Graphical abstract

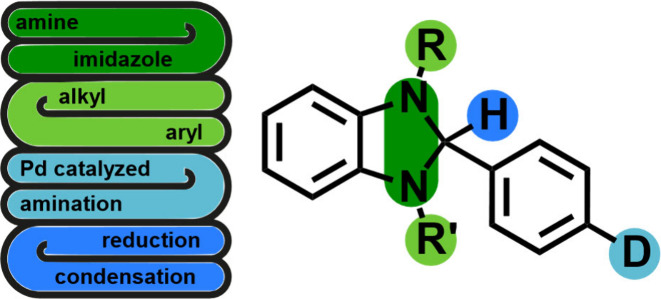

Molecular dopants have a fundamental role in tuning the electrical properties of organic semiconductors for a plethora of device applications.^1–3^ Direct doping of organic semiconductors requires a direct electron transfer reaction between the dopant and the target material. In the case of n doping, due to the relatively high energy of the LUMO levels of common organic semiconductors (around −4.0 eV for most high-EA “n-type” materials), direct n doping requires the use of strong reducing agents having low ionization energies (IEs), which are very susceptible to oxidation in air and, consequently, difficult to handle and synthesize.^4,5^ A valuable approach to overcome this issue is the use of kinetically air-stable n dopant precursors. These are metastable compounds having an IE that is too high to promote direct electron transfer to semiconductors but that can undergo an *in situ* chemical transformation resulting in the formation of a very low IE species (effective dopant) upon activation via thermal treatment or light irradiation.^6–10^ The most studied n dopant precursors are the benzimidazoline derivatives, whose most representative example is 4-(2,3-dihydro-1,3-dimethyl-1*H*-benzimidazol-2-yl)-*N*,*N*-dimethylbenzenamine (N-DMBI-H) (Scheme 1).^8,9^ Despite possessing several advantages, such as good solubility in common processing solvents and commercial availability, the doping performances of this derivative with some semiconductors are still too poor for applications requiring high doping levels. Notably, bulk and vertical phase segregation processes occurring in the dopant/semiconductor blend were proven to be a key limiting factor with several semiconductors and inversely correlate with doping efficiency.^8,11–13^ Such segregation is an expected phenomenon as molecular dopants likely form metastable or kinetically stable mixtures with conjugated polymers as already observed in donor–acceptor blends for OPV.^14^ In this scenario, the required thermal annealing step that leads to the activation of the doping cascade also enhances the diffusion of the dopant itself and its segregation into a separate phase where it remains inactive. Structural modifications of the dopant can tune both the kinetic stability of the dopant/semiconductor blend and its diffusivity within the matrix, thus providing a tool to enhance performance. The introduction of solubilizing *N*-alkyl substituents on the dopant molecule (Scheme 1, class 1 derivatives) or glycol chains has been reported to be a viable strategy to limit phase segregation phenomena and improve doping efficiency.^11,15,16^ 1,3-Dimethyl-2-phenylbenzimidazoline (DMBI) derivatives bearing substituents with extended π-conjugation (Scheme 1, class 2 derivatives) were also shown to provide a slower segregation kinetic with improved doping efficiency, possibly as the result of interaction of the extended π-system with the semiconductor^12,17^ and/or as an effect of the reduced diffusivity resulting from the increased Stokes radius.^18^ Multifunctional dopants with more than one active functionality (Scheme 1, class 3 derivatives) can act as electrostatic cross-linkers after activation, further hindering the diffusion processes as a result of the modification of the matrix.^19^ It is well established that the introduction and modulation of pendant π-releasing/withdrawing groups in conjugation with the π-system have a direct impact on the energies of FMOs. When applied to DMBI dopants, this approach has been reported to influence both doping efficiency and stability.^18,20–22^ Further improvement of all-around performances of DMBI dopants by core functionalization can thus leverage both the aforementioned diffusion control and FMO tuning strategies.

**Scheme 1:**
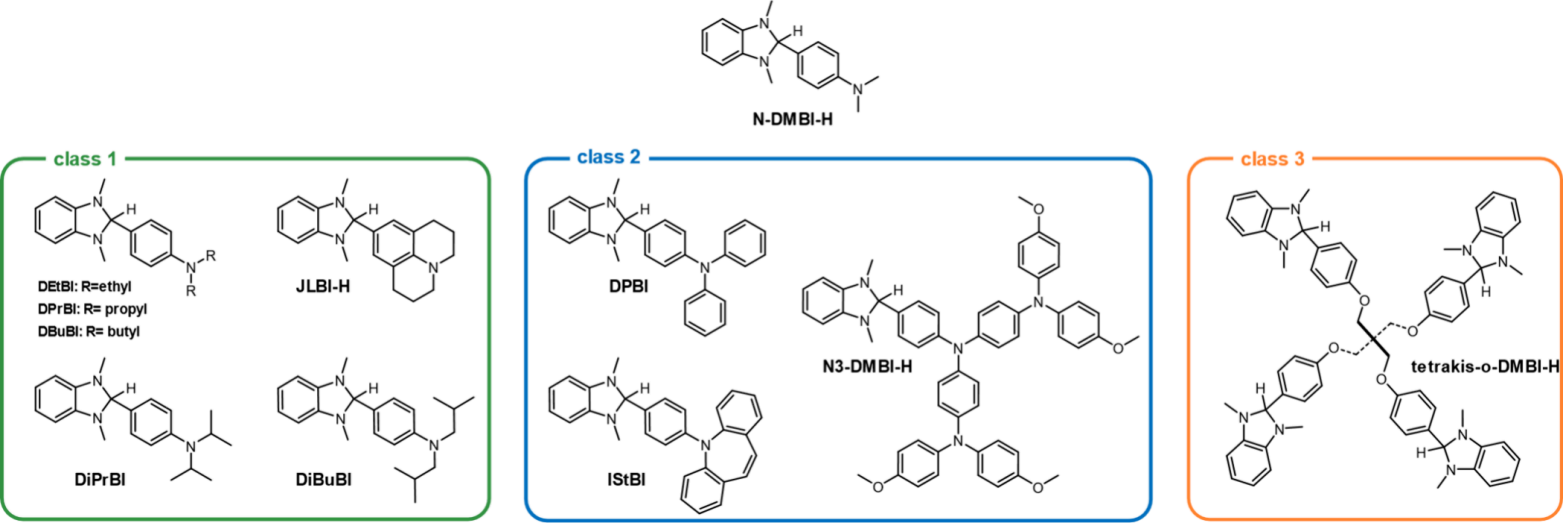
Chemical Structures of N-DMBI-H and Other DMBI-like Derivatives Studied in the Literature and Categorized on the Basis of Their Structural Features^a^

a Class 1: derivatives with different alkyl substituents at the aniline nitrogen. Class 2: derivatives with aromatic substituents at the aniline nitrogen. Class 3: multifunctional derivatives.

This in turn requires the widening of the available structural diversity without making the synthesis overly challenging. Despite the existence of several protocols for the preparation of 1*H*-benzimidazoline derivatives, no study of their generality is present in the literature, and guidelines in the selection of the best synthetic approach for a specific functionalization pattern are missing. In this work, we compare the pros and cons of known synthetic protocols for the preparation of a wide library of N-DMBI derivatives, known and original, characterized by different functionalization patterns as well as electronic properties. We guide the reader in the selection of the most appropriate approach, depending on the features of the target dopant. All of the original derivatives are completely characterized and could represent the platform for further improvement in the understanding of the doping process.

An initial version of this work was deposited in chemRxiv on February 13, 2025.^23^

## Results and Discussion

### Synthesis of Dopants

Scheme 2 shows the main literature approaches for the preparation of DMBI derivatives. A first approach consists of the direct condensation reaction between an *N*,*N*′-dialkyl-phenylene-1,2-diamine derivative and an aldehyde, generally performed in the presence of glacial acetic acid as the catalyst (route A).^11,15,19,24^ The same reaction can also be performed in the heterogeneous phase in the presence of an acidic clay as the catalyst, as we recently reported.^25^ The alternative route (route B) requires the reduction of a benzimidazolium salt with sodium borohydride. The former can be obtained either via condensation of a phenylene-1,2-diamine derivative and an acyl chloride^10^ (route B1) or via alkylation of 2-arylbenzimidazoles (route B2). 2-Arylbenzimidazoles can in turn be prepared by reaction between a phenylene-1,2-diamine and an aldehyde (or carboxylic acid) or via reductive cyclization between an aldehyde and a 2-nitroaniline activated in the presence of sodium dithionite as the reducing agent.^24,26^ All of these protocols are in principle suitable for a diverse functionalization of the *para* position of the 2-phenyl ring of the DMBI scaffold and for the preparation of derivatives bearing different alkyl chains on the benzimidazole nitrogen atoms, via selection of the proper starting reagents. In terms of the number of reaction steps, route A would be preferable, especially if the starting aldehyde and *o*-phenylenediamine are commercially available. The main drawback of this protocol is the high cost of *N*,*N*′-dialkyl-substituted 1,2-phenylenediamine.

**Scheme 2:**
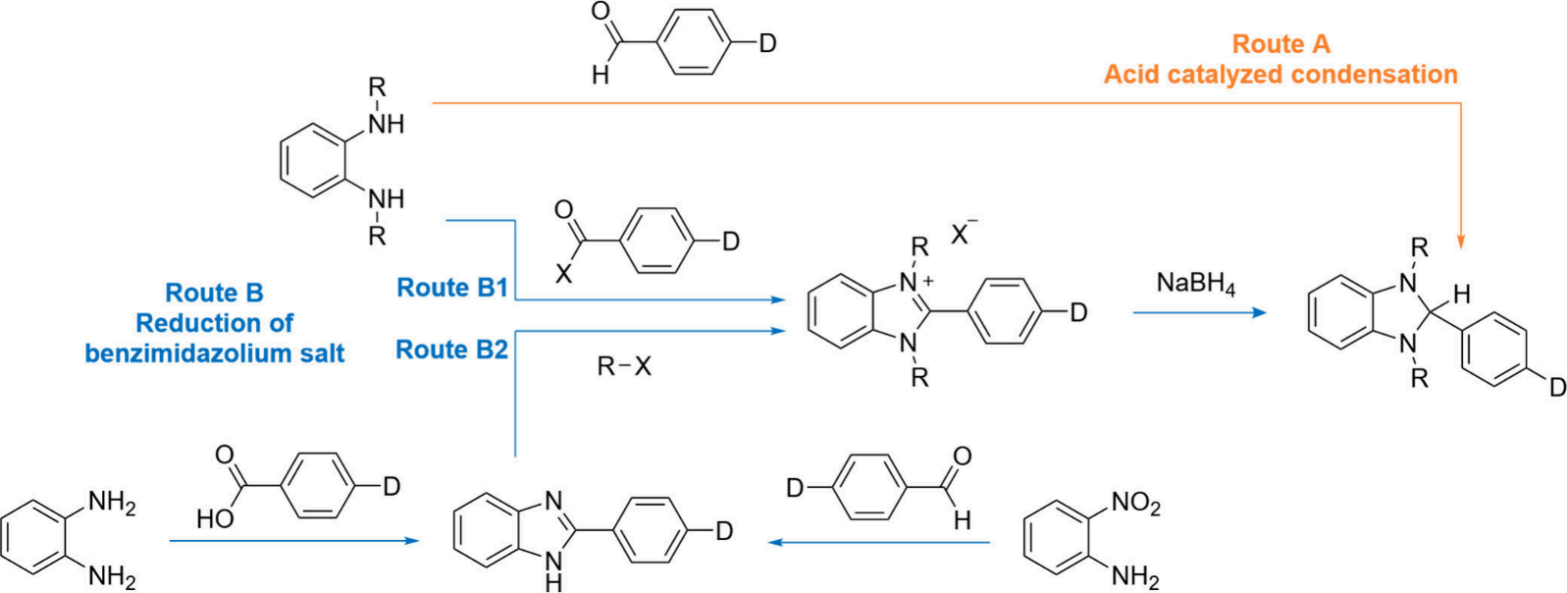
Literature Routes for the Synthesis of Benzimidazoline Derivatives

In addition, the reaction is an acid-catalyzed condensation reaction (aminal formation) whose equilibrium might prove difficult to shift toward complete conversion. Route B1 instead suffers from the limited commercial availability of acyl chlorides as more reactive alternatives to the corresponding acids. Route B2 is preferable for the preparation of derivatives bearing different substituents on the two imidazoline nitrogen atoms, since the alkylation can happen in different steps. The use of 2-nitroanilines as a starting reagent is, within this protocol, particularly advantageous. Several N-substituted 2-nitroanilines are commercially available. Whenever this is not the case, *N*-aryl or alkyl derivatives can be prepared by S_N_Ar reacting 2-halo-nitrobenzene (F or Cl) with the desired amines,^27,28^ a reaction that is possible to perform in one pot with the subsequent reductive cyclization according to a work by Yang et al.^27^ Notably, such a synthetic scheme gives access to asymmetrically and aryl *N*,*N*′-disubstituted benzimidazoles.

All such considerations are qualitative, and most researchers involved in the field would probably stick to the preferred route, unless suggested to do otherwise by solid arguments. We thus decided to test the various types of synthetic access we mentioned in the synthesis of a variety of different dopants and evaluated the outcome in terms of yield, number of steps, and ease of purification. In doing so, we also introduced meaningful process optimizations with respect to literature protocols, leading to updated versions identified as route A* and route B*. Scheme 3 summarizes such updated synthetic pathways and the corresponding conditions.

**Scheme 3:**
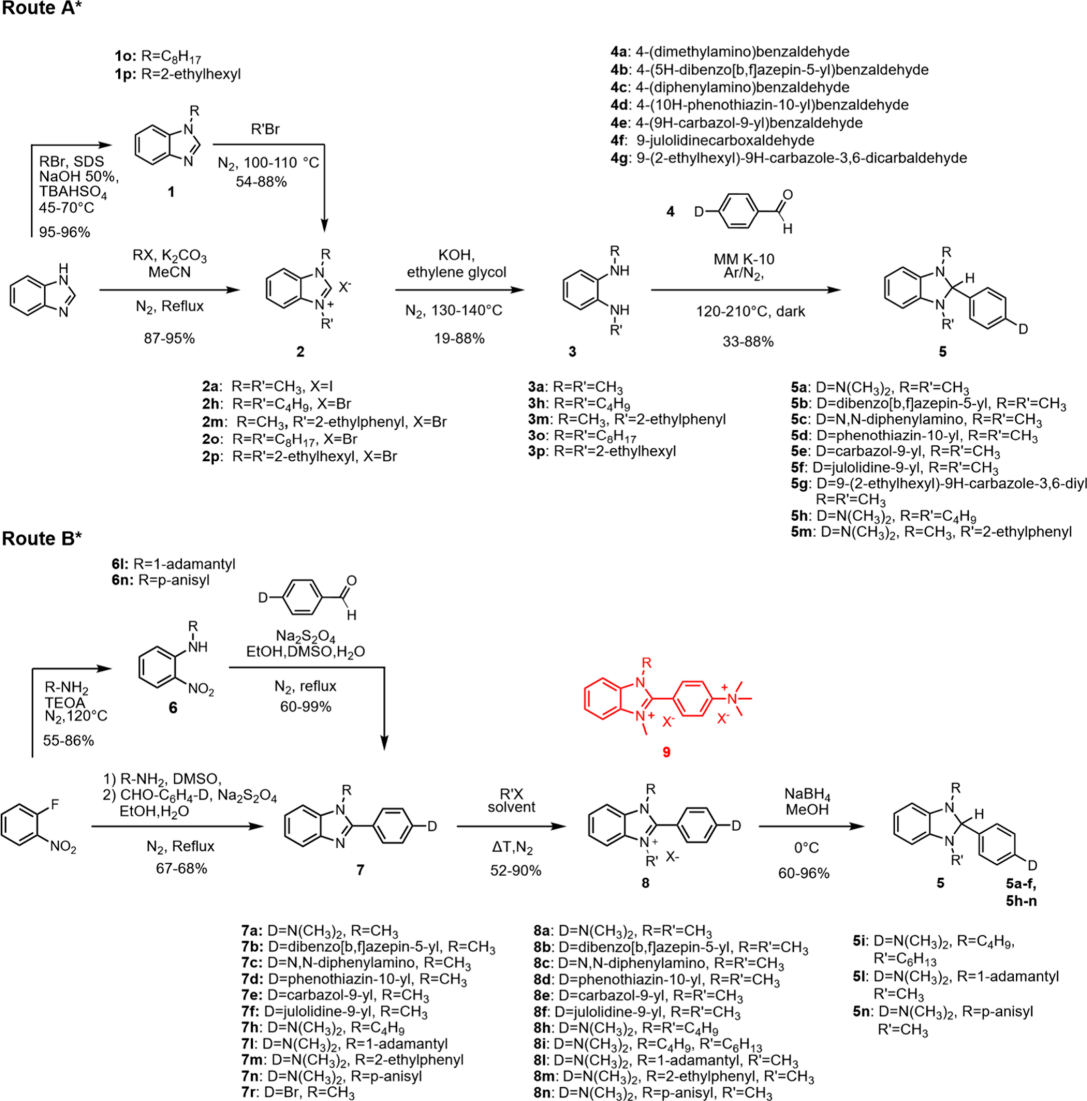
Synthetic Routes A* and B* for the Synthesis of Substituted Benzimidazolines

### Condensation Route A*

Route A* provides the final product through condensation between the corresponding aldehyde and *N*,*N*′-dialkyl-1,2-phenylenediamine. The synthesis of aldehydes having general structure **4** is typically straightforward, either by formylation of the corresponding anilines or by Buchwald–Hartwig amination of 4-bromobenzaldehyde (see also section S1.4 of the Supporting Information). Conversely, the common route to *N*,*N*′-dialkyl-1,2-phenylenediamine is a tedious multistep process, requiring the protection of 1,2-diaminobenzene as the corresponding ditosylate and N-alkylation of the resulting sulfonamide, followed by deprotection under harsh acidic conditions. The overall yield can reach 70%, but the atom economy is very poor. We recently improved on this method showing that *N*,*N*′-dimethyl-1,2-phenylenediamine (**3a**) can be prepared in two steps by alkylation of benzimidazole followed by alkaline hydrolysis of salt **2a**.^25^ Our protocol offers the additional advantage of being compatible with the preparation of asymmetrically substituted diamines, as the alkylation of benzimidazole can be easily carried out in two steps using different alkylating agents. Indeed, we prepared the *N*,*N*′-dibutyl-, *N*,*N*′-dioctyl, and *N*,*N*′-(2-ethylhexyl)­benzimidazolium salts (**2h**, **2o**, and **2p**, respectively), by reaction with an excess of the corresponding halide. We also prepared asymmetric benzimidazolium salt **2m**, featuring a methyl and a 2-ethyl phenyl chain, by alkylation of commercially available 1-methylbenzimidazole with 1-phenyl-2-bromoethane. Unfortunately, the alkaline hydrolysis step leads to the formation of byproducts, such as the corresponding ureas, that can be easily removed only in the case of **3a** and **3h** and that instead reduce the purity of the target derivative below 90% in the case of products **3m**, **3o**, and **3p**. In the case of asymmetric diamine **3m**, we succeeded in purifying the product, but with a reduction of the isolated yield to 19% due to the severe instability of this species when handled in air.

The condensation of aldehyde and diamine is generally performed under homogeneous conditions and acid catalysis, sometimes under sonication.^11^ We recently reported an acid-catalyzed solvent-free method that is superior in terms of both yield and sustainability.^17^ The approach requires the use of montmorillonite clay K-10 that acts both as a catalyst and as a dehydrating agent, a feature critical to address the reversible nature of the condensation reaction. Due to the limited stability of DMBI derivatives,^25,29^ the reaction is performed under a nitrogen atmosphere in the dark. Provided that the reaction temperature reaches the melting points of the starting reagents and effective mixing is obtained, the protocol allows the preparation of products **5a**–**g** and **5m**. Yields above 60% were obtained for derivatives **5b**–**d** and **5f**, characterized by alkyl or π-conjugated substituents on the nitrogen in position 4 of the 2-phenyl ring. Products **5e** and **5g** bearing a carbazole substituent were obtained in more modest 54% and 44% yields, respectively, due to their high melting point (>200 °C, vide infra). In fact, as the product concentration increases during the process, it induces solidification of the melted reaction mixture, making it difficult to achieve complete conversion. In all cases, the reaction proceeded smoothly, and the products were isolated either by taking up with the appropriate solvent and filtration or by chromatography after removal of the clay.

Derivatives **3a** and **4e** are commercially available from different suppliers. Without considering the preparation of these two reactants in the evaluation, overall yields for the syntheses are generally higher than 40%, with the only exception being dimeric dopant **5g**, isolated in 16% yield. As a comparison, the original route A leads to N-DMBI-H in 26% yield.^11^ We also extended the protocol to a derivative characterized by an asymmetric functionalization pattern (**5m**). Nonetheless, due to the poor yield of the preparation of diamine **3m**, we could isolate the product in an only overall 6% yield.

### Reduction Route B*

For route B*, we optimized the procedure originally reported by Yang et al.^27^ The key step is a one-pot condensation/reductive cyclization reaction between an *o*-nitroaniline (general structure **6**) and an aldehyde (general structure **4**) in the presence of Na_2_S_2_O_4_. The reaction is generally performed in a refluxing mixture of ethanol, dimethyl sulfoxide (DMSO), and water under an inert atmosphere. For derivatives **7c**, **7f**, and **7l**, the reaction proceeded smoothly even without addition of DMSO due to the good solubility of the starting reagents in ethanol. The addition of water to the mixture favors conversion, as reported by Oda and co-workers.^30^ If not commercial, the starting 1,2-nitroanilines can be obtained by reacting 1-fluoro-2-nitrobenzene with selected amines R-NH_2_ in the presence of triethanolamine (TEOA). The preparation of products **6** and the subsequent reductive cyclization to the desired aryl benzimidazoles (general structure **7**) can also be performed in one pot with no need to isolate the 1,2-nitroaniline. The reductive cyclization reaction afforded the 2-arylbenzimidazole precursors (general structure **7**) with isolated yields varying between 60% and 99%. The synthesis of the required N-substituted 2-nitroaniline and the following reductive cyclization step were successfully performed in one pot in the case of products **7h**, **7i**, and **7m**, with fair yields of around 67%. The preparation of benzimidazoles functionalized with conjugated aromatic amines was also possible via Buchwald–Hartwig amination of derivative **7r**, as discussed in the Supporting Information. Alkylation of products **7**, followed by reduction with NaBH_4_ of obtained benzimidazolium salts **8** in methanol, finally affords the desired products. The preparation of benzimidazolium salts (general structure **8**) via alkylation of products **7** is unfortunately dependent on the substrate. In particular, methylation reactions of 2-arylbenzimidazoles functionalized with *N*,*N*-dimethylaniline required careful optimization of the solvent, temperature, and methylation agent to avoid quaternization of the aniline nitrogen (byproducts **9**). Performing the reaction under conditions leading to the precipitation of the monomethylated product was indeed fundamental to limit the formation of this undesired impurity. In the case of derivatives functionalized with non-nucleophilic aromatic amines (**8b**–**e**), the reaction proceeded smoothly, even when using strong methylation agents, such as methyl iodide and methyl triflate. In general, no dialkylation side product was detected when alkyl bromides were used. In the case of derivative **8a**, the selection of toluene as the reaction medium allowed the formation of **9** to be hampered and prevented complete conversion of reagent **7a** due to its poor solubility in this solvent, with consequent contamination of the recovered product with the residual reagent. Route B* enables the preparation of most of the compounds that can be prepared via route A*. The only exception is dimeric derivative **5g** whose poorly soluble key intermediates strongly limited conversion. In the final reduction step, all of the products precipitated from the reaction medium and were then isolated via simple filtration, with the exception of derivatives **5a**, **5h**, and **5i**. In the case of derivative **5a**, the product was isolated with a purity of around 95%, due to the presence of traces of residual **7a** already contaminating **8a**. Figure 1 compares the adopted synthetic approaches in terms of overall reaction yields, the number of reaction steps, and ease of purification for the preparation of **5a**–**n** derivatives. Whenever both protocols are applicable and the derivative possesses a symmetric functionalization pattern, route A* is mostly superior in terms of yields. However, this path has the relevant flaw of featuring an equilibrium reaction in the last step. Whenever the final dopant cannot be purified in the solid state via taking up with the proper solvent and filtration, removal of unreacted aldehyde and diamine requires chromatography. DMBI-H derivatives are reported to be particularly prone to oxidation, especially when adsorbed on silica, dissolved and under light exposure.^25,29^ We observed similar behaviors for the obtained derivatives, as highlighted in section S7, reporting degradation kinetics in solution for a subset of products. Chromatography is a combination of all such conditions, which dramatically reduces yields and, in the case of **5h**, also prevents isolation of analytical samples (Figure S1 shows that **5h** isolated through route A* has a maximum purity of ∼83%). Not all DMBI degradation derivatives are detrimental for the doping process,^25^ yet the precise control of the composition even when using dopant/nucleating agent blends is paramount and requires the availability of pure samples. Route B* has a higher number of steps and in most cases a lower yield but leads to the target dopants through the irreversible and high-yield reduction of shelf-stable benzimidazolium salts **8**. In summary, whenever the target dopant is highly crystalline or poorly soluble and diamine **3** is symmetrically substituted and easily available, route A* is preferable. In all other cases, and especially when the dopant has a nonsymmetric substitution pattern and it is particularly prone to oxidation, route B* should be preferred. The latter route gives access to derivatives that so far could not be prepared and whose electronic and physicochemical features combined could offer additional tools for the precision doping of organic semiconductors. To benefit researchers looking for the best possible trade-offs among the stability, solubility, and doping capability over different target semiconductors, we carried out an extensive comparative characterization of the original versus established dopants, also including a computational rationalization. The results are discussed in the following section.

**Figure 1:**
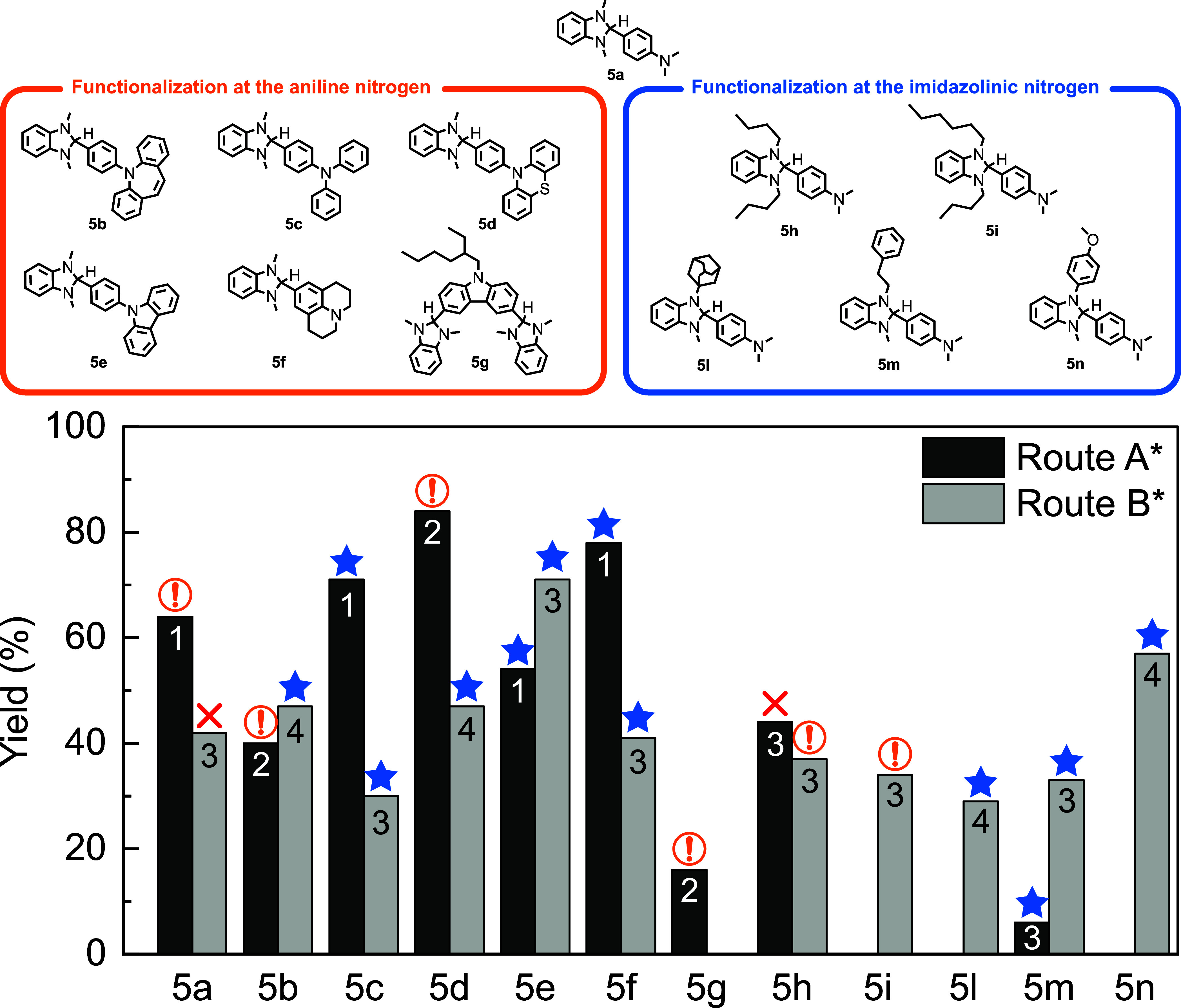
Structures of the obtained products (**5a**–**n**), together with a comparison between routes A* and B* in terms of their overall yield, the number of steps, and the ease of purification of the final product (the blue star indicates that the product can be isolated by straightforward procedures; the orange exclamation mark indicates issues due to product instability during purification; the red cross indicates that the attempted isolation of the high-purity product failed). The synthesis of reactants **3a** and **4e** was not considered for the overall yield evaluation, due to the commercial availability at fair cost (<$7000/mol) of these chemicals.

### Characterization of Dopants

As discussed above, DMBI-like dopants usually require *in situ* thermal activation.^8,9,11^ If the dopant melting point is lower than the activation temperature, its interdiffusion in the polymer phase is favored, in some cases leading to the enhancement of doping efficiency.^17^ This effect is particularly noticeable in sequential doping, as demonstrated by Fabiano et al.^31^ Annealing also improves the degree of crystallinity of the host polymer, often enhancing mobility.^32,33^ However, as the evolution of the polymer morphology happens while doping, it is necessary to carefully balance all thermally activated phenomena to prevent extensive phase segregation. Tuning of the melting temperature and of diffusion-promoted phase segregation (demixing) within the polymeric matrix is as important as the intrinsic doping capabilities. We thus performed comparative thermal characterization of the new dopants with respect to N-DMBI-H. Table 1 shows the degradation temperatures of all dopants according to a thermogravimetric analysis (TGA) performed under an inert atmosphere (see Figure S2 for the corresponding thermograms).

**Table 1:** Melting Points, Degradation Temperatures, HOMO Energy Levels of the Synthesized Derivatives (DH), and SOMO Energy Levels (D^•^) of the Corresponding Benzimidazolium Salts^a^

				DH HOMO (eV)			D^•^ SOMO (eV)	
product (DH)	mp (*T*_d_)^a^	solubility (mg/mL)^b^	*E*_ox_ (V) (DH^•+^/DH)	DPV^c^ (IE)	DFT^d^	*E*_red_ (V) (D^+^/D^•^)	DPV^c^ (EA)	DFT^d^
**5a**	106–110 °C (194 °C)^25^	139	–0.22 ^17^	–4.6 ^17^	–4.78	–2.28 ^17^	–2.5 ^17^	–2.54
**5b**	180–184 °C (229 °C)^17^	15	–0.21 ^17^	–4.6 ^17^	–4.78	–2.20 ^17^	–2.6 ^17^	–2.64
**5c**	143–147 °C (234 °C)	182	–0.21 ^17^	–4.6 ^17^	–4.84	–2.00 ^17^	–2.8 ^17^	–2.80
**5d**	181–185 °C (252 °C)	15	–0.10	–4.7	–4.90	–1.99	–2.8	–2.97
**5e**	248–252 °C (251 °C)	1.7	–0.09	–4.7	–4.89	–1.99	–2.8	–2.95
**5f**	159–163 °C (196 °C)	136	–0.17	–4.6	–4.77	–2.37	–2.4	–2.50
**5g**	209–213 °C (234 °C)	36	–0.16	–4.6	–4.78	–	–	–2.65
**5h**	<RT (200 °C)	–	–0.22	–4.6	–4.77	–2.24	–2.6	–2.48
**5i**	<RT (197 °C)	–	–0.23	–4.6	–4.69	–2.26	–2.5	–2.48
**5l**	209–222 °C (193 °C)	15	–0.23	–4.6	–4.65	–2.32	–2.5	–2.54
**5m**	82–85 °C (191 °C)	>50	–0.20	–4.6	–4.71	–2.17	–2.6	–2.52
**5n**	151–152 °C (212 °C)	28	–0.11	–4.7	–4.79	–2.15	–2.7	–2.63

a HOMO and SOMO levels are reported as calculated via DFT along with ionization energies evaluated via DPV. Oxidation (*E*_ox_) and reduction (*E*_red_) potentials reported vs Fc/Fc^+^ and evaluated from DPV measurements are also reported.

b mp values were evaluated considering the onset and the peak temperature of DSC endothermic peaks. Degradation temperature *T*_d_ is extrapolated from TGA thermograms. Further details of the evaluation of *T*_d_ are reported in section S3.

c Solubility in toluene, at RT. Further information about the evaluation of dopants solubilities is reported in section S4. Solubility was not evaluated for samples that are liquid at room temperature.

d IEs and EAs were evaluated with respect to the Fc/Fc^+^ couple using the formula *E*_vac_ = −*e*(*E*_0_ + 4.8 V), where *E*_0_ is the redox potential measured from DPV analysis. Further details are reported in section S5.

e Calculated via the composite M062X+D3+ZPE method (details in the Supporting Information).

All derivatives decompose above 190 °C, consistently with the behavior of parent N-DMBI-H (**5a**). Dopants featuring a π-excessive donating ring are particularly stable. We then measured the melting points via differential scanning calorimetry (DSC), still under a nitrogen atmosphere. Data obtained for derivative **5c** agree well with those reported in the literature for the same dopants.^34^ In the case of derivative **5f**, literature reports a melting point of 147 °C, below the value we measured (∼163 °C).^15^ We nonetheless observed the appearance of a second melting peak located at 148 °C when performing a second heating cycle on the same sample (see section S2). This suggests that this specific product might exist in two different crystal polymorphs having different melting temperatures, in analogy to our previous observations on N-DMBI-H (**5a**).^25^ Derivatives **5h** and **5i** are liquids at room temperature due to the effect of the solubilizing alkyl chains. Derivative **5m** also melts at a lower temperature (85 °C) with respect to N-DMBI-H (110 °C). All other derivatives melt at a significantly higher temperature with carbazole-functionalized **5e** reaching a mp of 252 °C. The trend in the melting points is partially reflected in the solubility in toluene, with the high-melting point materials having solubility (<40 mg/mL) significantly lower than that of N-DMBI-H (>100 mg/mL). From the standpoint of electronic properties, the dopant main features depend upon the HOMO and singly occupied molecular orbital (SOMO) energies of the relevant chemical species. According to the literature, doping mediated by DMBI-like derivatives happens via the transfer of H^–^ to the semiconductor. Mechanisms mediated by H^•^ abstraction and formation of highly reducing DMBI^•^ species have also been proposed.^8,9,20^ Since these dopants are not doped by direct, ground state electron transfer to the polymer, the HOMO gives an indication of the environmental stability rather than ranking the thermodynamic doping capabilities. Doping instead usually depends on the position of the SOMO of DMBI^•^, since this is the true radical doping species in the hypothesis of doping mediated by homolytic cleavage of the C–H aminal bond, and the hydride donor strength depends on the radical ionization potential as well.[Fn notes-3],^8,9,20,22^ Cyclic voltammetry (CV) and differential pulse voltammetry (DPV) allow estimation of both the IE value of the neutral dopants and the EA of the corresponding cationic precursors **8** from their respective solution redox potentials (see section S5 for the details). Table 1 compares these experimental results (IE and EA) obtained via DPV, with their corresponding energies calculated at the density functional theory (DFT-M062X+D3+ZPE) level. Electrochemically derived IEs and EAs and DFT-calculated energies show similar trends, with calculated values slightly lower on average by 0.16 eV in the case of HOMO levels and IEs and in closer agreement with DPV measurements in the case of SOMO levels and EAs. These results support the advantageous use of calculations in the presynthetic design of tailored molecules in this and other fields of applications. Parent N-DMBI-H features an electrochemical HOMO of −4.6 eV and a corresponding SOMO of −2.5 eV.^17^ The substitution of the benzimidazoline methyl groups with alkyl chains (**5h** and **5i**) has very little impact on the HOMO and SOMO position. A similar effect is observed in the cases of derivatives **5l** and **5m**, showing no or small stabilization of both energy levels with respect to the parent molecule. The presence of the anisidyl ring in **5n** leads to the stabilization of both the HOMO (−4.7 eV) and the SOMO (−2.7 eV). This suggests that the delocalization of the imidazoline nitrogen lone pair over the benzene ring dominates over the electron-donating effect of the methoxy substituent. Derivatives **5b**–**e** (all featuring an electron rich heteroaromatic residue on the benzene ring) behave differently. They all feature a negligibly stabilized HOMO with respect to N-DMBI-H, in agreement with previous reports showing that the HOMO level of DMBI-like derivatives is mainly localized on the imidazole core.^20^ The SOMOs are more significantly affected by the donating heterocycles with energies of −2.6 eV for **5b** and −2.8 eV for **5c**–**e**. The particularly high SOMO of **5b** reflects the antiaromatic character of dibenzoazepine.^35^ In the case of multifunctional dopant **5g**, again featuring a heteroaromatic residue, a similar effect is observed in terms of HOMO stabilization. The SOMO level of this derivative corresponds to the IE of the dopant having lost only one of the two active hydrogen atoms and as such characterized by one DMBI^•^-like site. It was thus not possible to measure this energy level, due to the difficulty in synthesizing the corresponding monocationic precursor required for the electrochemical analysis. Nonetheless, according to DFT data, this dopant is expected to have a slightly stabilized SOMO level with respect to the parent molecule, in line with our previous observations on derivatives with extended conjugation.

Derivative **5f**, functionalized with an electron-donating julolidine moiety, features a HOMO level of −4.6 eV, close to that of the parent molecule and in line with that previously reported for this dopant.^15^ The SOMO level is instead localized at −2.4 eV and is destabilized with respect to that of N-DMBI-H, reflecting the stronger electron donor character of the rigid julolidine moiety. DFT simulations confirm and enrich the description of structure–property relationships in this class of molecules, also explaining counterintuitive results. The spatial distribution of the SOMO orbitals of neutral radicals obtained from **5a** and all of the **5b**–**n** derivatives is shown in the top part of Figure 2. Focusing on **5b**–**f** derivatives functionalized at the aniline N, we correlate the marked diminution of SOMO values to the involvement of such an N atom in the orbital itself. Indeed, we can separate species **5c**–**e** having a reduced level of participation of N to the SOMO from **5b** and **5f** where such participation is very similar to that of parent molecule **5a**. In turn, this involvement can be correlated to the possibility of aligning the p orbitals along the C–N bond highlighted in the figure, hindered in the case of **5c**–**e**. On the other hand, in the case of **5h**–**n** derivatives functionalized at the imidazolinic nitrogen, the SOMO values are all closer to that of **5a**, in agreement with a similar involvement of the aniline N in the SOMO for all molecules. Further details can be obtained through an analysis of selected difference density maps, shown in the bottom part of Figure 2. The maps show the difference between the electronic density of the neutral radical and the closed shell cation, highlighting the electronic rearrangement following the doping process. Charge is depleted from red regions and accumulated in blue regions when one electron is subtracted to the neutral radical. A comparison between **5d** and **5f** supports the idea that conjugation along the C–N bond is active in the case of **5f** (green arrow) and blocked in the case of **5d** (blue arrow). **5h** and **5n** confirm that, irrespective of the weak (yellow arrow) or strong (red arrow) electron-donating nature of substituents attached to the imidazolinic nitrogen, there is a negligible contribution of both in the stabilization of the cation, leading to charge displacements very similar to those of **5a**.

**Figure 2:**
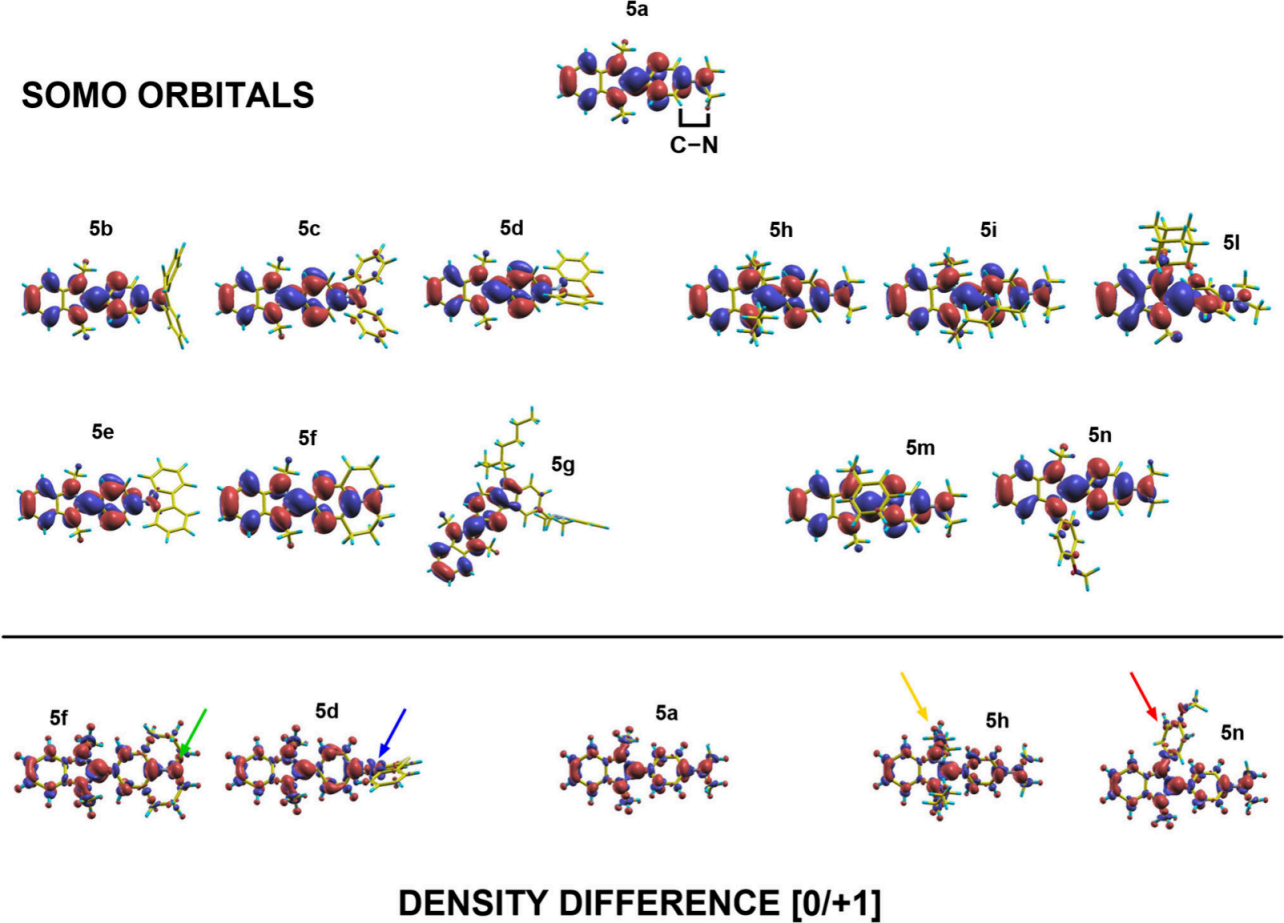
Spatial distribution of the SOMO orbitals of neutral radicals **5a**–**n** (top). Difference density maps for selected derivatives (bottom), obtained as the difference between the electronic density of the neutral radical and the closed shell cation.

In summary, the HOMO and SOMO positions suggest that all derivatives can dope typical high-EA semiconductors (having LUMO energies around or below −4.0 eV), provided that they are suitably activated. The fine details in the solubility and thermal properties can help in tuning the morphology prior to and after thermal annealing, providing extra tools to control the intrinsically multivariate doping approach. The doping of poly­{[*N*,*N*′-bis­(2-octyldodecyl)-naphthalene-1,4,5,8-bis­(dicarboximide)-2,6-diyl]-alt-5,5′-(2,2′-bithiophene)} (also known as P­(NDI2OD-T2)) with a representative subset of the synthesized derivatives has been fully studied electrically and morphologically, and the results have been published elsewhere.^36^ Here we report electrical conductivities of P­(NDI2OD-T2)/dopant blend films containing 40 mol % dopant (with respect to the polymer repeating unit) in Figure 3 for all of the derivatives.

**Figure 3:**
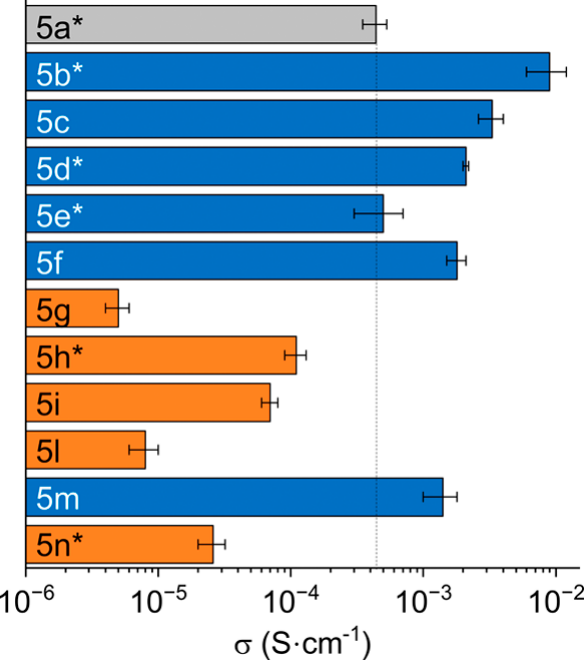
Electrical conductivity (σ) of P­(NDI2OD-T2) films containing 40% mol derivative **5**, spin coated from toluene and annealed at 180 °C for 2 h under a N_2_ atmosphere. An asterisk indicates that the conductivity is reported from ref 36.

The pristine polymer has an electrical conductivity on the order of ∼10^–7^ S cm^–1^,^25,36^ while the conductivities of all blends exceed 10^–5^ S cm^–1^, with derivatives **5b**–**d**, **5f**, and **5m** overcoming the performance of parent dopant **5a**. Such values confirm the capability of all of the presented derivatives to dope the selected polymer.

## Conclusions

In this work, we summarized, further optimized, and comparatively analyzed the two most common synthetic approaches for the preparation of benzimidazoline-based dopants for organic semiconductors: acid-catalyzed condensation of substituted diaminobenzenes with aromatic aldehydes (direct condensation) and a multistep reductive amination protocol (reductive amination). We applied the modified protocols to the synthesis of a wide library of known and original N-DMBI derivatives, characterized by different functionalization patterns, including both alkyl and aryl substituents at different nitrogen sites. We concluded that the direct condensation approach is suitable for air-stable compounds with limited solubility. The possibility of isolating the product by filtration in the last step is critical to avoid contamination from reagents. Conversely, whenever the higher solubility prevents filtration or the air sensitivity limits the applicability of chromatography, the reductive amination is preferable. The latter is also more versatile for the preparation of unsymmetrically substituted and multifunctional derivatives. To the benefit of the growing community of printed electronics, we also tested all of the dopants in terms of thermal and electronic features, thus contributing to widening the space of available tools to improve doping efficiency and performance.

## Materials and Methods

Reagents were purchased from TCI, BLDpharm, Sigma-Aldrich, and Fluorochem. Solvents were bought from Merck, Carlo-Erba, and Acros and used as received unless otherwise stated. Palladium catalysts were purchased from Apollo Scientific. Chromatographic purifications were performed using a Davisil LC 60A silica gel (pore size of 60 Å, 70–200 μm). The composition of solvent mixtures used as eluents is indicated as volume/volume ratios. Melting points were determined using a Buchi M-560 apparatus. NMR spectra were recorded on a Bruker NMR Avance 400 NEO instrument. Microwave-activated reactions were performed by using a Discover-S CEM microwave.

The synthetic procedures for all of the reported intermediates and products are reported in section S1.

### Thermal Characterization

DSC measurements were performed with a DSC 1 STARe system from Mettler Toledo, using aluminum crucibles. Calibration was performed with an indium standard. All DSC measurements were conducted under a N_2_ flow (80 mL/min) at a rate of 10 °C/min. DSC crucibles were prepared and closed inside a glovebox under an argon atmosphere (<0.1 ppm O_2_, <0.1 ppm H_2_O) and then punctured just before the analysis was performed.

TGA measurements were performed with a TGA/DSC1 STARe system from Mettler Toledo, using alumina crucibles. All measurements were conducted under a N_2_ flow (50 mL/min) at a rate of 10 °C/min. Thermal ramps from 30 to 600 °C were generally used. Due to their very poor stability in air, crucibles containing compounds **5h** and **5i** were prepared inside a glovebox under an argon atmosphere (<0.1 ppm O_2_, <0.1 ppm H_2_O) and then exposed to air just before the analysis was performed.

### Electrochemical Characterization

Electrochemical characterization of the synthesized dopants and of the corresponding benzimidazolium salts was performed inside an argon-filled glovebox (<0.1 ppm O_2_, <0.1 ppm H_2_O) using a three-electrode system. An AMEL glassy carbon pin electrode (3 mm diameter) mirror polished with deagglomerated alumina paste (0.3 μm, purchased from Buelher) and milliq H_2_O was used as the working electrode, a platinum wire as the counter electrode, and a Ag/AgCl wire as the quasi reference electrode (a Ag reference electrode was used for the characterization of derivatives **5f**–**m**). The obtained potentials were then referred to the Fc/Fc^+^ redox couple. Anhydrous acetonitrile (99.8%, Alfa Aesar) containing between 2.0 and 5.0 mM substrate (depending on solubility) and 0.1 M tetrabutylammonium perchlorate (99%, Thermo Scientific) as a supporting salt was used as the electrolyte.

Cyclic voltammetry (CV) analysis was performed at 50 mV/s, while differential pulse voltammetry (DPV) was performed using either steps of 5 mV/s and a modulation amplitude of 50 mV or steps of 2 mV/s and a modulation amplitude of 25 mV. Since the systems showed high resistance, an ohmic drop compensation between 160 and 200 Ω was applied.

### Computational Methods

Atomistic simulations of dopants have been carried out using a multilevel protocol. The most stable conformers of pristine (**R-H**) and activated (**R·**) dopant molecules have been individuated using a conformer–rotamer ensemble sampling tool (CREST)^37^ based on the GFN2-xTB tight-binding Hamiltonian as the “engine” for the calculations of energies and forces.^37,38^ The electronic properties of the most stable structures found by CREST have been investigated in the framework of density functional theory simulations, using the ORCA suite of programs.^39,40^ In detail, the Kohn–Sham orbitals have been expanded on the all-electron def2-TZVPP Gaussian-type basis set.^41,42^ The corresponding def2/J basis has also been used as an auxiliary basis set for Coulomb fitting in a resolution-of-identity/chain-of-spheres (RIJCOSX) level of approximation. Molecular geometries have been fully optimized using the B3LYP functional^43^ with the addition of the pairwise D3 correction for the calculation of dispersion forces.^44^ Redox potentials of all of the molecules have been calculated using the M06-2X hybrid functional^45^ and the same combination of def2-TZVPP-def2/J basis sets, with all of the investigated neutral and charged species immersed in an implicit CH_2_Cl_2_ solvent using a conductor-like polarizable continuum model (CPCM)^46^ to calculate electronic and solvation energies. Thermochemical properties of the same molecules were calculated using the B3LYP functional introduced above. Redox potentials were calculated as Δ*G* values between neutral and charged species.

### Electrical Conductivity Measurements

Samples for electrical conductivity characterization were prepared according to the following procedure. Metallic contacts were patterned on 15 mm × 15 mm Corning glass (low alkali, 1737F) by means of a MB-ProVap-3 thermal evaporator, depositing a 3 nm Cr adhesion layer followed by a 50 nm Au layer. Active channels were characterized by a 15 mm width and an interelectrode distance of 5 mm. Substrates were cleaned with deionized water, acetone, and 2-propanol (ultrasonic bath, 10 min per solvent) and then exposed to a O_2_ plasma treatment (Femto Diener electronic) at 100 W for 10 min. The whole film preparation procedure was then performed inside a N_2_-filled glovebox. A 10 mg/mL stock solution of P­(NDI2OD-T2) polymer (*M*_n_ of 26.2 kDa, polydispersity index of 2.65, synthesized according to a previously reported protocol)^36^ was prepared in toluene. Aliquots of this polymer solution and dopant stock solutions in toluene were mixed to reach a dopant concentration of 40% in moles with respect to the polymer repeating unit and a final polymer concentration of 7 mg/mL. Thin films were obtained from these solutions via spin coating (1000 rpm for 60 s, 3000 rpm for 10 s) and then annealed at 180 °C for 2 h. Electrical conductivity was then measured using a Wentworth Laboratories probe station connected to an Agilent B1500A semiconductor device parameter analyzer in a two-point contact configuration. The *I–V* characteristics were collected at room temperature inside a glovebox, under a nitrogen atmosphere. Forward and backward scans were performed to exclude the presence of hysteresis, and the electrical conductivity (σ) was calculated according to the formula σ = *l*(*Rwt*)^−1^, where *t* is the film thickness, *l* is the active channel length, *w* is the active channel width, and *R* is the resistance extrapolated from the *I–V* curves. The thickness of the obtained films was evaluated using an Alpha-step IQ, KLA Tencor profilometer, yielding average thicknesses of 45 ± 5 nm for derivatives **5g** and **5m**, 50 ± 5 nm for **5c**, 60 ± 5 nm for **5f** and **5l**, and 70 ± 5 nm for **5i**.

## Supplementary Material



## Footnotes

a To improve readability here, we simply refer to the HOMO and SOMO level of derivatives. Note that whenever we mention a DMBI-H (DH) derivative SOMO we are actually referring to the SOMO of the corresponding D^•^ active dopant that is formed upon activation and H^•^ abstraction.

## Notes

Details on the synthesis of all of the described derivatives, NMR spectra, TGA and DSC characterization, complete electrochemical characterization, further details of dopant solubility and stability evaluation, and information about electric conductivity measurements and further computational details (PDF)
