# Supplementary material for: Hitchhiker’s Guide to the Preparation of Novel Benzimidazoline-Based n‑Type Dopants
Source: Chem Mater. 2025 Sep 15;37(19):7823–33. doi: 10.1021/acs.chemmater.5c01479 (PMC12530191; doi:10.1021/acs.chemmater.5c01479)
Supplement: Supplementary file 1 [file cm5c01479_si_001.pdf]

Supporting Information: Hitchhiker's guide to the preparation of novel benzimidazoline based n-type dopants

Francesca Pallini,† Giulia Garavaglia,† Gabriele Paoli,† Giuseppe Mattioli,‡ Francesco Porcelli,‡ Lorenzo Mezzomo,† Domenico Antonio Florenzano,† Riccardo Ruffo,† Pietro Rossi,¶ Mario Caironi,¶ Mauro Sassi,† and Sara Mattiello*,†

†*Department of Materials Science, University of Milano-Bicocca, Via R. Cozzi 55, 20125 Milano, Italy.*

‡*Consiglio nazionale delle ricerche - Istituto di struttura della materia (ISM-CNR) Area della Ricerca di Roma 1, Via Salaria km 29.300, Monterotondo 00015, Italy*

¶*Center for Nano Science and Technology, Istituto Italiano di Tecnologia Via Rubattino 81, 20131 Milano, Italy*

E-mail: sara.mattiello@unimib.it

Contents

List of Figures **S-3**

1 Synthetic procedures **S-6**

1.1 Synthesis of N-alkylbenzimidazoles S-6

1.2 Synthesis of N,N′-dialkylimidazolium salts S-8

1.3 Synthesis of N,N′-dialkyl-*o*-phenylenediamines S-12

1.4 Synthesis of benzaldehydes S-16

1.5 Synthesis of N-alkyl-2-nitroanilines S-20

1.6 Synthesis of N-alkyl-2-arylbenzimidazoles S-22

1.7 Synthesis of N,N′-diakyl-2-aryl-benzimidazolium salt S-33

1.8 Synthesis of dopants S-43

2 Thermal characterization - DSC **S-58**

3 Thermal characterization - TGA **S-67**

4 Dopants solubility evaluation **S-69**

5 Electrochemical characterization **S-70**

6 Computational methods **S-85**

7 Dopants stability **S-87**

8 NMR spectra **S-95**

References for the Supporting Information **S-163**

List of Figures

S1 Details of the aromatic portion of the ^{1}H NMR in $CDCl_3$ of derivative **5h** as synthesized via condensation reaction. S-54

S2 TGA characterization of derivatives **5a-5n** S-68

S3 Plausible oxidation products of DMBI-like derivatives, according to previous reports on oxidation by-products of **5a** and **5b**.[S1–S3] S-69

S4 Evolution of ^{1}H NMR of derivative **5d** in $CDCl_3$. S-88

S5 Evolution of ^{1}H NMR of derivative **5e** in $CDCl_3$. S-89

S6 Evolution of ^{1}H NMR of derivative **5f** in $CDCl_3$. S-90

S7 Evolution of ^{1}H NMR of derivative **5i** in $CDCl_3$. S-91

S8 Evolution of ^{1}H NMR of derivative **5l** in $CDCl_3$. S-92

S9 Evolution of ^{1}H NMR of derivative **5n** in $CDCl_3$. S-93

S10 Degradation kinetic of products **5d** (A), **5e** (B), **5f** (C), **5i** (D), **5l** (E) and **5n** (F) dissolved in chloroform. S-94

S11 ^{1}H NMR of derivative **2m** in CD_3OD S-99

S12 $^{13}C\{^{1}H\}$ NMR of derivative **2m** in CD_3OD S-100

S13 ^{1}H NMR of derivative **2p** in $CDCl_3$. S-101

S14 ^{1}H NMR of derivative **3m** in $CDCl_3$. S-102

S15 $^{13}C\{^{1}H\}$ NMR of derivative **3m** in $CDCl_3$. S-103

S16 ^{1}H NMR of derivative **3p** in $CDCl_3$. S-104

S17 $^{13}C\{^{1}H\}$ NMR of derivative **3p** in $CDCl_3$. S-105

S18 ^{1}H NMR of derivative **7b** in DMSO$-d_6$. S-106

S19 $^{13}C\{^{1}H\}$ NMR of derivative **7b** in DMSO$-d_6$. S-107

S20 ^{1}H NMR of derivative **7d** in DMSO$-d_6$. S-108

S21 $^{13}C\{^{1}H\}$ NMR of derivative **7d** in DMSO$-d_6$. S-109

S22 ^{1}H NMR of derivative **7f** in DMSO$-d_6$. S-110

S23 $^{13}C\{^{1}H\}$ NMR of derivative **7f** in DMSO$-d_6$. S-111

S24 ^{1}H NMR of derivative **7h** in $CDCl_3$. S-112
S25 ^{13}C{^{1}H} NMR of derivative **7h** in $CDCl_3$. S-113
S26 ^{1}H NMR of derivative **7l** in $CDCl_3$. S-114
S27 ^{13}C{^{1}H} NMR of derivative **7l** in $CDCl_3$. S-115
S28 ^{1}H NMR of derivative **7m** in DMSO−d_6. . . . . S-116
S29 ^{13}C{^{1}H} NMR of derivative **7m** in DMSO−d_6. . . . . S-117
S30 ^{1}H NMR of derivative **7n** in DMSO−d_6. . . . . S-118
S31 ^{13}C{^{1}H} NMR of derivative **7n** in DMSO−d_6. . . . . S-119
S32 ^{1}H NMR of derivative **8c** in DMSO−d_6. . . . . S-120
S33 ^{13}C{^{1}H} NMR of derivative **8c** in DMSO−d_6. . . . . S-121
S34 ^{1}H NMR of derivative **8d** in DMSO−d_6. . . . . S-122
S35 ^{13}C{^{1}H} NMR of derivative **8d** in DMSO−d_6. . . . . S-123
S36 ^{19}F NMR of derivative **8d** as triflate salt in DMSO−d_6. . . . . S-124
S37 ^{1}H NMR of derivative **8e** in DMSO−d_6. . . . . S-125
S38 ^{13}C{^{1}H} NMR of derivative **8e** in DMSO−d_6. . . . . S-126
S39 ^{19}F NMR of derivative **8e** as triflate salt in DMSO−d_6. . . . . S-127
S40 ^{1}H NMR of derivative **8f** as iodide salt in DMSO−d_6. . . . . S-128
S41 ^{13}C{^{1}H} NMR of derivative **8f** as iodide salt in DMSO−d_6. . . . . S-129
S42 ^{1}H NMR of derivative **8f** as methyl sulfate salt in DMSO−d_6. . . . . S-130
S43 ^{13}C{^{1}H} NMR of derivative **8f** as methyl sulfate salt in DMSO−d_6. . . . . . S-131
S44 ^{1}H NMR of derivative **8h** in $CDCl_3$. S-132
S45 ^{13}C{^{1}H} NMR of derivative **8h** in $CDCl_3$. S-133
S46 ^{1}H NMR of derivative **8i** in $CDCl_3$. S-134
S47 ^{13}C{^{1}H} NMR of derivative **8i** in $CDCl_3$. S-135
S48 ^{1}H NMR of derivative **8l** in DMSO−d_6. . . . . S-136
S49 ^{13}C{^{1}H} NMR of derivative **8l** in DMSO−d_6. . . . . S-137
S50 ^{1}H NMR of derivative **8m** in DMSO−d_6. . . . . S-138

S51 $^{13}C\{^1H\}$ NMR of derivative **8m** in DMSO$-d_6$. S-139
S52 ^1H NMR of derivative **8n** in DMSO$-d_6$. S-140
S53 $^{13}C\{^1H\}$ NMR of derivative **8n** in DMSO$-d_6$. S-141
S54 ^1H NMR of derivative **5a** in DMSO$-d_6$. S-142
S55 ^1H NMR of derivative **5b** in C_6D_6. . . . . S-143
S56 ^1H NMR of derivative **5c** in $CDCl_3$. S-144
S57 $^{13}C\{^1H\}$ NMR of derivative **5c** in $CDCl_3$. S-145
S58 ^1H NMR of derivative **5d** in C_6D_6. . . . . S-146
S59 $^{13}C\{^1H\}$ NMR of derivative **5d** in C_6D_6. . . . . S-147
S60 ^1H NMR of derivative **5e** in $CDCl_3$. S-148
S61 $^{13}C\{^1H\}$ NMR of derivative **5e** in $CDCl_3$. S-149
S62 ^1H NMR of derivative **5f** in DMSO$-d_6$. S-150
S63 ^1H NMR of derivative **5g** in C_6D_6. . . . . S-151
S64 $^{13}C\{^1H\}$ NMR of derivative **5g** in C_6D_6. . . . . S-152
S65 ^1H NMR of derivative **5h** in DMSO$-d_6$. S-153
S66 $^{13}C\{^1H\}$ NMR of derivative **5h** in DMSO$-d_6$. S-154
S67 ^1H NMR of derivative **5i** in $CDCl_3$. S-155
S68 $^{13}C\{^1H\}$ NMR of derivative **5i** in $CDCl_3$. S-156
S69 ^1H NMR of derivative **5l** in $CDCl_3$. S-157
S70 $^{13}C\{^1H\}$ NMR of derivative **5l** in $CDCl_3$. S-158
S71 ^1H NMR of derivative **5m** in DMSO$-d_6$. S-159
S72 $^{13}C\{^1H\}$ NMR of derivative **5m** in DMSO$-d_6$. S-160
S73 ^1H NMR of derivative **5n** in DMSO$-d_6$. S-161
S74 $^{13}C\{^1H\}$ NMR of derivative **5e** in DMSO$-d_6$. S-162

1 Synthetic procedures

Reagents were purchased from TCI, BLDpharm, Sigma-Aldrich and Fluorochem. Solvents were bought from Merck, Carlo-Erba and Acros, and used as received unless otherwise stated. Palladium catalysts were purchased from Apollo Scientific. Chromatographic purifications were performed using Davisil LC 60A silica gel (pore size 60 Å, 70-200 µm). Composition of solvent mixtures used as eluents are indicated as volume/volume ratios.

Melting points were determined using a Buchi M-560 apparatus.

GC-MS spectra were collected on a Clarus 560 S PerkinElmer having an Elite-5MS 30.0 m $\times$ 250 µm column. Helium was used as carrier gas.

NMR spectra were collected on a Bruker NMR Avance 400 NEO.

Microwave activated reactions were performed using a Discover-S CEM microwave.

1.1 Synthesis of N-alkylbenzimidazoles

1.1.1 Synthesis of derivative 1o

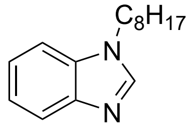

In a 50 mL round-bottom flask, benzimidazole (5.000 g, 42.32 mmol), 1-bromooctane (8.173 g, 42.32 mmol), sodium dodecylsulfate (610 mg, 2.17 mmol) and tetrabutylammonium hydrogen sulfate (540 mg, 1.59 mmol) are dispersed in 11 mL of water, then NaOH (11.000 g, 275.08 mmol) is added. The reaction is stirred at 45 °C for 18 hours. Reaction progress can be monitored by TLC using toluene/AcOEt 1:1 as the eluent. 50 mL of water and 30 mL of Et_2O are added, and the mixture is extracted with a separating funnel. The aqueous phase is extracted three more times with Et_2O (25 mL each time). The organic phase is dried over Na_2SO_4, which is then filtered, and the solvent is evaporated under reduced pressure. Residual 1-bromooctane is evaporated under reduced pressure (~0.02 mbar) at 50 °C. The product is isolated as a colorless oil (9.370 g, 96.2% yield).

^1H NMR (400 MHz, $CDCl_3$): δ[ppm] 7.90 (s, 1H), 7.82-7.80 (m, 1H), 7.41-7.39 (m, 1H),

7.33-7.27 (m, 2H), 4.17 (t, J = 7.2 Hz, 2H), 1.88 (quin, J = 7.2 Hz, 2H), 1.33-1.25 (m, 10H), 0.87 (t, J = 7.1 Hz, 3H).[S4]

1.1.2 Synthesis of derivative 1p

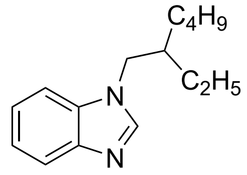

In a 100 mL roundbottom flask, NaOH (17.605 g, 440.13 mmol) is dissolved in water (17.605 g), then benzimidazole (8.000 g, 67.71 mmol) is added and the mixture is heated at 45 °C. Sodium dodecylsulfate (976 mg, 3.38 mmol) and tetrabutylammonium hydrogen sulfate (1.148 g, 3.381 mmol) are added and the reaction stirred for 5 minutes before adding 2-ethylhexyl bromide (13.078 g, 67.716 mmol). The temperature is raised to 50 °C and the reaction is stirred for 18 hours. Reaction progress can be monitored by TLC using toluene/AcOEt 1:1 as the eluent. Since benzimidazole is still present after 18 hours, 1.310 g (6.777 mmol) of 2-ethylhexyl bromide are added. The temperature is raised to 70 °C and the reaction is stirred for 4 hours. At this point, reaction is complete and the mixture is cooled down to room temperature. 70 mL of water and 40 mL of Et_2O are added, and the mixture is extracted with a separating funnel. The aqueous phase is extracted two more times with Et_2O (30 mL each time). The organic phase is dried over Na_2SO_4, which is then filtered, and the solvent is evaporated under reduced pressure. Residual 2-ethylhexyl bromide is evaporated under reduced pressure (~0.02 mbar) at 50 °C. The product is isolated as a pale yellow oil (14.781 g, 94.8% yield).

^{1}H NMR (400 MHz, $CDCl_3$): δ[ppm] 7.87-7.86 (m, 1H), 7.82-7.79 (m, 1H), 7.39-7.37 (m, 1H), 7.31-7.25 (m, 2H), 4.03 (d, J = 7.1 Hz, 2H), 1.95-1.86 (m, 1H), 1.36-1.24 (m, 8H), 0.91 (t, J = 7.5 Hz, 3H), 0.87 (t, J = 6.9 Hz, 3H).[S5]

1.2 Synthesis of N,N′-dialkylimidazolium salts

1.2.1 Synthesis of derivative 2a

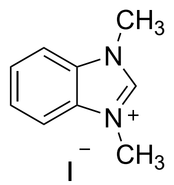

In a 500 mL roundbottom flask, benzimidazole (10.000 g, 84.645 mmol) and K_2CO_3 (11.700 g, 84.660 mmol) are put under nitrogen atmosphere. Acetonitrile (80 mL) is then added, and the mixture is heated to 80 °C. Methyl iodide (36.041 g, 253.92 mmol) is then added, and the reaction is refluxed for 24 hours. 50 mL of solvent are distilled before lowering the temperature to rt. The mixture is diluted with Et_2O, and the obtained precipitate is filtered off. Solvent is evaporated, and the raw mixture is extracted with dichloromethane using a Soxhlet apparatus. Solvent is evaporated under reduced pressure, and the obtained solid is crystallized using 300 mL of toluene/MeCN 1:1. 20.430 g of product are recovered by filtration and other 1.697 g precipitate after concentrating the organic solution of half volume. Overall, 22.127 g of product are recovered as a white powder (80.726 mmol, 95.4% yield). mp 189-194 °C.

^{1}H NMR (400 MHz, DMSO−d_6) δ[ppm] 9.66 (s, 1H), 8.03 (m, 2H), 7.72 (m, 2H), 4.09 (s, 6H).[S6]

1.2.2 Synthesis of derivative 2h

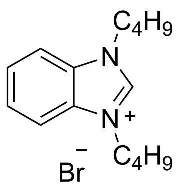

In a 500 mL roundbottom flask, benzimidazole (15.000 g, 126.97 mmol) and K_2CO_3 (17.547 g, 126.97 mmol) are put under nitrogen atmosphere. Acetonitrile (80 mL) is then added, followed by 1-bromobutane (26.637 g, 194.40 mmol), and the reaction is heated at reflux. Reaction progress can be monitored by TLC using DCM/AcOEt 1:1 as the eluent. After 26 hours, no more benzimidazole is present, so the mixture is cooled down to room temperature, and the precipitate ($KHCO_3$ and KBr) is filtered off and washed with 50mL of acetonitrile. Solvent is evaporated under reduced pressure, and the crude

is put again under nitrogen atmosphere. 75 mL of anhydrous toluene and 1-bromobutane (28.10 g, 205.1 mmol) are added, and the mixture is refluxed for 26 hours. The reaction is finally cooled down to room temperature, and the precipitate is filtered off and washed with 10 mL of toluene and 10 mL of Et_2O. The solid is dried under reduced pressure at 65 °C to constant weight. 34.437 g of pure product are recovered in the form of a white powder (110.63 mmol, 87.1% yield). mp 131-132 °C

^{1}H NMR (400 MHz, DMSO−d_6) δ[ppm] 9.94 (s, 1H), 8.13-8.11 (m, 2H), 7.70-7.68 (m, 2H), 4.51 (t, J = 7.2 Hz, 4H), 1.90 (quin, J = 7.4 Hz, 4H), 1.37-1.30 (m, 4H), 0.92 (t, J = 7.4 Hz, 6H).[S7]

1.2.3 Synthesis of derivative 2m

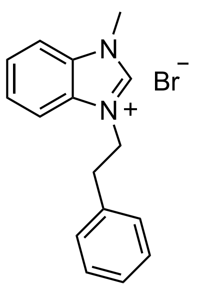

In a two neck 50 mL roundbottom flask, 1-methylbenzimidazole (5.00 g, 37.8 mmol) is added and the system is put under N_2 atmosphere. After being flushed with nitrogen, (2-Bromoethyl)benzene (7.73 g, 41.8 mmol) is added to the flask and the mixture is heated at 110 °C. The reaction progress is monitored by TLC using DCM/AcOEt 1:1 as the eluent. After 16 hours reagents conversion is complete and the reaction is stopped. The crude is dissolved in methanol (50 mL) and then extracted with petroleum ether (2 x 30 mL). The recovered methanol is evaporated under reduced pressure to afford an oil. 30 mL of Et_2O are added to the oil and the mixture is stirred for 30 minutes. The Et_2O is removed and the procedure is repeated twice. After the second washing, a pale yellow solid is obtained and recovered by filtration. The solid is finally dried under reduced pressure at 45 °C to constant weight. 10.66 g of product are recovered (33.6 mmol, 88.9% yield). mp 103-109 °C. Anal. Calcd for $C_{16}H_{17}BrN_2$: C, 60.58; H, 5.40; N, 8.83. Found: 60.32; H, 5.47; N, 8.78.

^{1}H NMR (400 MHz, CD_3OD) δ[ppm] 9.43 (s, 1H), 7.94 (m, 1H), 7.88 (m, 1H), 7.73-7.65 (m, 2H), 7.28-7.22 (m, 3H), 7.19-7.17 (m, 2H), 4.83 (t, J = 7.1 Hz, 2H), 4.12 (s, 3H), 3.33

(t, J = 7.1 Hz, 2H).

$^{13}C\{^1H\}$ NMR (100 MHz, $CDCl_3$) δ[ppm] 143.28, 137.99, 133.39, 132.61, 129.90, 129.88, 128.26, 128.11, 114.49, 114.30, 49.76, 36.34, 33.90.

1.2.4 Synthesis of derivative 2o

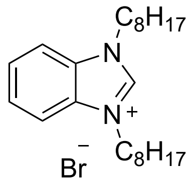

In a 100 mL roundbottom flask, 1-octylbenzimidazole (derivative **1o**, 6.300 g, 27.35 mmol) and 1-bromooctane (5.810 g, 30.08 mmol) are put under N_2 atmosphere and the solution is heated up to 110 °C for 3 hours. 10 mL of toluene are then added, and the reaction is heated for 3 hours more. After cooling down to room temperature, a white precipitate forms. 15 mL of Et_2O are added, and the precipitate is recovered by filtration and then washed with 10 mL of petroleum ether. The solid is dried under reduced pressure at 65 °C to constant weight. 10.223 g of pure product are recovered in the form of a white powder (24.141 mmol, 88.3% yield). mp 106-107 °C

^1H NMR (400 MHz, $CDCl_3$) δ[ppm] 11.54 (s, 1H), 7.71-7.64 (m, 4H), 4.62 (t, J = 7.6 Hz, 4H), 2.05 (quin, J = 7.6 Hz, 4H), 1.44-1.21 (m, 20H), 0.86 (t, J = 7.2 Hz, 6H).[S8]

1.2.5 Synthesis of derivative 2p

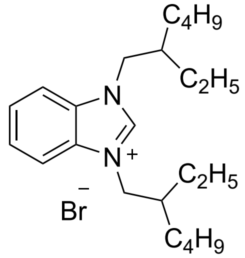

In a 50 mL roundbottom flask, 1-ethylhexylbenzimidazole (derivative **1p**, 4.000 g, 17.36 mmol) and 2-ethylhexyl bromide (3.689 g, 19.10 mmol) are put under N_2 atmosphere and the solution is heated up to 110 °C for 20 hours. After cooling to room temperature, 30 mL of petroleum ether are added and the mixture is stirred for 20 minutes. Phase separation is observed, so the supernatant is removed, and the residue is dried under reduced pressure. The oily residue is dissolved in AcOEt (30 mL), 30 mL of petroleum ether are added and the mixture is stirred for additional 20 minutes. Phase separation is observed, so the supernatant is removed, and the residue is finally dried under

reduced pressure. A mixture of the desired product and of 1-ethylhexylbenzimidazolium bromide[1] is obtained, in the form of a very viscous oil. The crude is used as is in the following step. (product to impurity ratio ~1.6:1)

^{1}H NMR (400 MHz, $CDCl_3$) δ[ppm] 11.40 (s, 1H), 7.69-7.63 (m, 4H), 4.61-4.49 (m, 4H), 2.01-2.05 (m, 2H), 1.47-1.24 (m, 16H), 0.94 (t, J = 7.4 Hz, 6H), 0.85 (t, J = 7.2 Hz, 6H).

As the product is a mixture of two substances, ^{13}C NMR spectrum was not recorded for this derivative.

[1]Probably, 2-ethylhexyl bromide partially undergoes elimination, with subsequent protonation of 1-ethylhexylbenzimidazole by the formed HBr

1.3 Synthesis of N,N′-dialkyl-*o*-phenylenediamines

1.3.1 Synthesis of derivative 3a

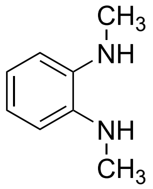

In a 100 mL roundbottom flask, derivative **2a** (33.500 g, 122.22 mmol), KOH (85%, 68.443 g, 1219.8 mmol) and ethylene glycol (56 mL) are put under nitrogen atmosphere. Mixture is heated at 130 °C for 2 hours and 20 minutes. The reaction is cooled down to room temperature, then water (75 mL) and Et_2O (110 mL) are added. Mixture is extracted, and the aqueous phase is the extracted with further Et_2O (7×55 mL). The organic phase is dried over KOH, then filtered and the organic solvent is evaporated under reduced pressure. The obtained product (brown oil, 16.147 g, 97%) is finally distilled under vacuum at 90 °C to afford 12.485 g of the pure diamine as a white waxy solid (91.667 mmol, 75% yield).

^{1}H NMR (400 MHz, DMSO$-d_6$) δ[ppm] 6.57 (m, 2H), 6.41 (m, 2H), 4.54 (m, 2H), 2.71 (d, J = 5.1 Hz, 6H).[S9]

The product was stored in the glovebox at 4 °C to avoid oxidation from atmosphere exposure.

1.3.2 Synthesis of derivative 3h

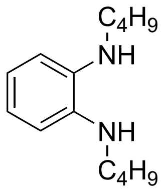

In a 100 mL roundbottom flask, derivative **2h** (10.000 g, 32.126 mmol), KOH (85%, 21.165 g, 321.26 mmol) and ethylene glycol (15 mL) are put under nitrogen atmosphere. Mixture is heated at 130 °C for 7 hours and reaction progress is monitored by TLC using heptane:AcOEt 85:15 as the eluent. The reaction is cooled to room temperature, then water (50 mL) and Et_2O (50 mL) are added. Mixture is extracted, and the aqueous phase is the extracted with further Et_2O (3×25 mL). The organic phase is dried over KOH, then filtered and the organic solvent is evaporated under reduced pressure. The

obtained product (brown oil, 6.712 g, 95%) is finally distilled under vacuum at 144 °C to afford 6.200 g of the diamine as a viscous oil (28.14 mmol, 87.5% yield).

Alternatively, the product can be purified by gradient elution column chromatography using heptane/AcOEt 99:1 → heptane/AcOEt 95:5 as eluent.

^{1}H NMR (400 MHz, $CDCl_3$) δ[ppm] 6.81-6.77 (m, 2H), 6.71-6.66 (m, 2H), 3.10 (t, J =7.2 Hz, 4H), 1.71-1.63 (m, 4H), 1.52-1.42 (m, 4H), 0.98 (t, J =7.4 Hz, 6H).[S10]

The product was stored in the glovebox at 4 °C to avoid oxidation from atmosphere exposure.

1.3.3 Synthesis of derivative 3m

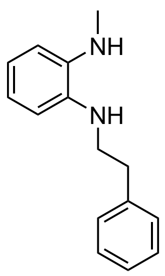

In a two neck 50 mL roundbottom flask, derivative **2m** (1.546 g, 4.873 mmol) and KOH (85%, 3.235 g, 49.01 mmol), are added. The system is put under N_2 atmosphere and degassed ethylene glycol (1.4 mL) is added to the flask. The reaction is heated at 135 °C under magnetic stirring to obtain a yellow mud. The reaction progress is monitored by TLC using DCM:AcOEt 3:1 as eluent. After 31 hours, the reaction is stopped and let cool down to room temperature. Water (45 mL) and Et_2O are added. The mixture is extracted and the aqueous phase is extracted with further Et_2O (3 ×20 mL). The organic phase is then washed with 20 mL of a 5% solution of NaOH in water, dried over KOH and filtered. The organic solvent is evaporated under reduced pressure to give a brown oil which is dissolved in 35 mL of Et_2O and extracted twice with 55 mL of aqueous HCl (5 wt%). The recovered aqueous phase is washed with further 45 mL of Et_2O and is then added with a 10% NaOH aqueous solution until pH 9 is reached and a pink solution is obtained. The solution is then extracted with Et_2O (3×55 mL) and the recovered organic phase is washed with water (50 mL). The recovered organic phase is dried over KOH, filtered and the solvent is evaporated under reduced pressure and finally under N_2. Black oil, 210 mg (0.928 mmol, 19% yield). Anal. Calcd for $C_{15}H_{18}N_2$: C, 79.61; H, 8.02; N, 12.38. Found:

79.41; H, 8.17; N, 12.23.

^{1}H NMR (400 MHz, $CDCl_3$) δ[ppm] 7.34-7.30 (m, 2H), 7.26-7.21 (m, 3H), 6.81 (m, 2H), 6.72 (m, 1H), 6.67 (m, 1H), 3.38 (t, J = 7.1 Hz, 2H), 2.97 (t, J = 7.1 Hz, 2H), 2.82 (s, 3H).

$^{13}C\{^1H\}$ NMR (100 MHz, $CDCl_3$) δ[ppm] 139.65, 138.69, 136.96, 128.90, 128.72, 126.54, 119.67, 119.14, 111.98, 111.00, 45.73, 36.02, 31.25.

The product was stored in the glovebox at 4 °C to avoid oxidation from atmosphere exposure.

1.3.4 Synthesis of derivative 3o

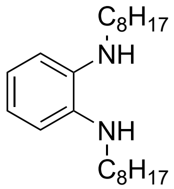

In a 100 mL roundbottom flask, derivative **2o** (5.000 g, 11.81 mmol), KOH (85%, 7.778 g, 118.1 mmol) and ethylene glycol (8 mL) are put under nitrogen atmosphere. Mixture is heated at 130 °C for 1 hour, then temperature is raised to 135 °C for 1 hour, then raised again to 140 °C for 24 hours. Reaction progress can be monitored by TLC using heptane/AcOEt 9:1 as the eluent. The reaction is cooled down to room temperature, then water (20 mL) and heptane (30 mL) are added. Mixture is extracted, and the aqueous phase is the extracted with further heptane (2×10 mL). The organic phase is washed with 2% aqueous NaOH (3×15 mL), and subsequently with brine (15 mL). The organic phase is dried over KOH, then filtered and the solvent is evaporated under reduced pressure. The obtained product is finally filtered on a silica pad (gradient elution, heptane → heptane/AcOEt 25:5 as eluent) to afford 2.672 g of the diamine as a viscous oil (8.034 mmol, 68.0% yield). Two impurities are present, lowering the purity of the product down to ~80-85%.

^{1}H NMR (400 MHz, $CDCl_3$) δ[ppm] 6.81-6.77 (m, 2H), 6.70-6.66 (m, 2H), 3.09 (t, J = 7.2 Hz, 4H), 1.71-1.64 (m, 4H), 1.47-1.27 (m, 20H), 0.90 (t, J =7.1 Hz, 6H).[S10]

The product was stored in the glovebox at 4 °C to avoid oxidation from atmosphere exposure.

1.3.5 Synthesis of derivative 3p

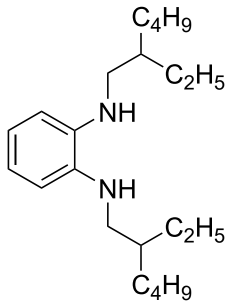

In a 100 mL roundbottom flask, derivative **2p** (2.090 g, 4.935 mmol), KOH (85%, 3.250 g, 49.33 mmol) and ethylene glycol (4 mL) are put under nitrogen atmosphere. Mixture is heated at 140 °C for 48 hours. Reaction progress can be monitored by TLC using heptane/AcOEt 9:1 as the eluent. The reaction is cooled down to room temperature, then water (20 mL) and heptane (30 mL) are added. Mixture is extracted, and the aqueous phase is the extracted with further heptane (2×10 mL), then organic phase is washed with 10% aqueous NaOH (3×15 mL). The organic phase is dried over KOH, then filtered and the solvent is evaporated under reduced pressure. The obtained product is finally filtered on a silica pad (gradient elution, heptane → heptane/AcOEt 95:5 as eluent) to afford 880 mg of the target phenylenediamine as a viscous oil (2.65 mmol, 60.2% yield). An impurity is present, lowering the purity of the product down to ∼85-90%.

^{1}H NMR (400 MHz, $CDCl_3$) δ[ppm] 6.81-6.77 (m, 2H), 6.71-6.69 (m, 2H), 3.00 (d, J = 6.1 Hz, 4H), 1.65-1.57 (m, 2H), 1.50-1.30 (m, 16H), 0.95-0.89 (m, 12H).

$^{13}C\{^1H\}$ NMR (100 MHz, $CDCl_3$) δ[ppm] 138.08, 119.07, 111.72, 47.66, 39.47, 31.77, 29.22, 25.01, 23.27, 14.22, 11.21.

The product was stored in the glovebox at 4 °C to avoid oxidation from atmosphere exposure.

1.4 Synthesis of benzaldehydes

Aldehydes **4b-e** were prepared by Buchwald-Hartwig amination following a previously reported protocol from our group.[S11] Aldehyde **4g** was prepared by formylation of N-ethylhexyl-carbazole according to published conditions.

1.4.1 Synthesis of derivative 4b

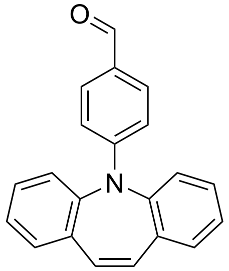

In a 100 mL roundbottom flask, 4-bromobenzaldehyde (1.851 g, 10.00 mmol), iminostilbene (2.421 g, 12.53 mmol), K_3PO_4 (3.188 g, 15.02 mmol), $Pd(OAc)_2$ (22.66 mg, 0.1009 mmol), XPhos (96.66 mg, 0.2027 mmol) and poly(ethylene glycol) dimethyl ether 2000 (2OMePEG2000, 195 mg) are added, and put under N_2 atmosphere. Toluene (6 mL) is added, and the reaction is heated at 110 °C for 2 hours and 10 minutes. Reaction progress can be monitored by TLC using DCM/heptane 8:2 as the eluent. Reaction is cooled down to room temperature, and filtered on celite to remove inorganic salts (eluting with AcOEt). Solvent is evaporated, and 5 mL of toluene are added: an orange precipitate forms (unreacted iminostilbene, 302 mg), which is filtered off. The toluene solution is then passed on a silica cake, eluting with toluene first (70 mL) and then toluene/AcOEt 95:5 (140 mL). Fractions containing product are collected, solvent is evaporated, and the crude (1.770 g) is taken up with 10 mL of Et_2O: a white precipitate forms, corresponding to the pure product (1.100 g), which is filtered off. The residue (670 mg) is finally crystallized with heptane (12 mL), affording 580 mg more of pure product in the form of a white powder. Overall, 1.680 g of product were recovered, with a yield of 56.5%. mp 120-121 °C.

^{1}H NMR (400 MHz, $CDCl_3$): δ[ppm] 9.69 (s, 1H), 7.56 - 7.47 (m, 8H), 7.41 (ddd, J = 7.8, 6.9, 1.5 Hz, 2H), 6.86 (s, 2H), 6.35 (d, J = 8.9 Hz, 2H).[S12]

Alternative conditions and workup tested: 4-bromobenzaldehyde (1.118 g, 6.043 mmol), iminostilbene (0.968 g, 5.01 mmol), K_3PO_4 (1.641 g, 7.731 mmol), $Pd(OAc)_2$ (13.48

mg, 0.0600 mmol), XPhos (49.42 mg, 0.1037 mmol) and poly(ethylene glycol) dimethyl ether 2000 (2OMePEG2000, 94 mg), toluene (1 mL). Reaction is heated at 110 °C for 3 hours, then cooled down to room temperature. Toluene is added and the crude is filtered on celite. Product is isolated by gradient elution column chromatography using toluene → toluene/AcOEt 95:5 as the eluent. 0.925 g (3.11 mmol) of pure product recovered as yellow powder, with a yield of 62.1%.

1.4.2 Synthesis of derivative 4d

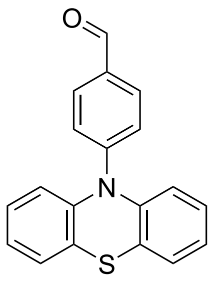

In a 100 mL roundbottom flask, 4-bromobenzaldehyde (557 mg, 3.01 mmol), phenothiazine (501 mg, 2.51 mmol), K_3PO_4 (819 mg, 3.86 mmol), $Pd(OAc)_2$ (6.30 mg, 0.0281 mmol), XPhos (24.08 mg, 0.0505 mmol) and poly(ethylene glycol) dimethyl ether 2000 (2OMePEG2000, 47 mg) are added, and put under N_2 atmosphere. Toluene (0.66 mL) is added, and the reaction is heated at 110 °C for 3 hours. Reaction progress can be monitored by TLC using DCM/heptane 3:1 as the eluent. Reaction is cooled down to room temperature, and filtered a silica pad, eluting with toluene. Fractions containing product are collected and solvent is evaporated to afford 742 mg of product as a yellow powder, with a yield of 95.9%. mp 107-109 °C.

^1H NMR (400 MHz, $CDCl_3$): δ[ppm] 9.86 (s, 1H), 7.75 (d, J = 8.8 Hz, 2H), 7.43 (dd, J = 7.7, 1.0 Hz, 2H), 7.32 - 7.26 (m, 4H), 7.21 - 7.15 (m, 4H).[S13]

1.4.3 Synthesis of derivative 4e

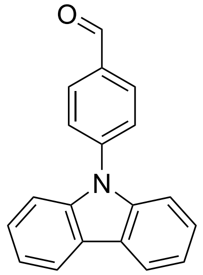

In a 100 mL roundbottom flask, 4-bromobenzaldehyde (1.119 g, 6.048 mmol), carbazole (0.845 g, 5.05 mmol), K_3PO_4 (1.643 g, 7.740 mmol), $Pd(OAc)_2$ (12.56 mg, 0.0559 mmol), XPhos (48.26 mg, 0.1012 mmol) and poly(ethylene glycol) dimethyl ether 2000 (2OMePEG2000, 94 mg) are added, and put under N_2 atmosphere. Toluene (1.2 mL) is

added, and the reaction is heated at 110 °C for 1 hour and 45 minutes. Reaction progress can be monitored by TLC using DCM:heptane 8:2 as the eluent. Reaction is cooled down to room temperature, toluene and water added, and the organic phase is washed in a separating funnel to remove the inorganic salts. The organic phase is dried over Na_2SO_4, filtered, and solvent is evaporated under reduced pressure. The crude is refluxed in 80 mL of EtOH, hot filtered, and the product crystallized in the cooled solvent. 860 mg of product are recovered as pale yellow crystals, with a yield of 62.7%. mp 156-158 °C.

^{1}H NMR (400 MHz, $CDCl_3$): δ[ppm] 10.12 (s, 1H), 8.16 - 8.13 (m, 4H), 7.80 (d, J = 8.3 Hz, 2H), 7.51 (dt, J = 8.2, 0.9 Hz, 2H), 7.44 (ddd, J = 8.2, 7.1, 1.2 Hz, 2H), 7.33 (ddd, J = 7.7, 7.1, 1.1 Hz, 2H).[S14]

1.4.4 Synthesis of derivative 4g

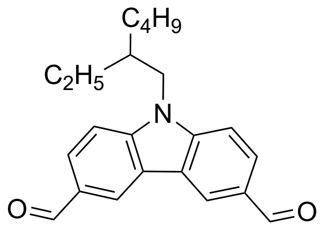

In a 100 mL roundbottom flask, DMF (12.032 g, 164.6 mmol) is put under N_2 atmosphere and cooled down to 0 °C with an ice bath. $POCl_3$ (27.434 g, 178.93 mmol) is added and the mixture is kept cold for 30 minutes. The ice bath is removed, and N-ethylhexylcarbazole (2.000 g, 7.157 mmol) is added into the flask as solution in CH_2Cl_2. The mixture is heated at 95 °C for 1.5 hours, then temperature is reaised at 105 °C for 5 hours. Reaction progress can be monitored by TLC using heptane/AcOEt 4:1 as the eluent. Reaction is cooled down to room temperature and ice (150 g) is added. NaOH 32% is added until the aqueous phase reaches pH >12, toluene is then added and the mixture is heated at 55 °C for 1 hour. After cooling down to room temperature, the mixture is extracted with toluene (4×50 mL), and the organic phase is then washed with brine (2×100 mL). The organic phase is dried over Na_2SO_4, which is then filtered, and the solvent is evaporated under reduced pressure. The crude is filtered on a silica pad using toluene, followed by toluene/AcOEt 9:1, as the eluent. Solid is taken up with petroleum ether (5 mL), filtered, and finally crystallized from cyclohexane

(20 mL) to afford 861 mg of pure product (2.57 mmol, 35.9% yield). m.p. 109-111 °C.

^{1}H NMR (400 MHz, $CDCl_3$): δ[ppm] 10.13 (s, 2H), 8.66 (d, J = 1.7 Hz, 2H), 8.08 (dd, J = 8.6, 1.6 Hz, 2H), 7.53 (d, J = 8.6 Hz, 2H), 4.25 (d, J = 7.6 Hz, 2H), 2.11-2.02 (m, 1H), 1.47-1.21 (m, 8H), 0.94 (t, J = 7.4 Hz, 3H), 0.85 (t, J = 7.4 Hz, 3H).[S15]

1.5 Synthesis of N-alkyl-2-nitroanilines

1.5.1 Synthesis of derivative 6l

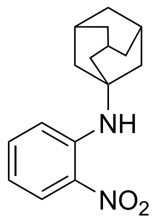

In a 100 mL roundbottom flask, 2-fluoronitrobenzene (2.117 g, 15.00 mmol) and 1-adamantylamine (2.269 g, 15.00 mmol) are put under nitrogen atmosphere. Triethanolamine (2.244 g, 15.04 mmol) is added, and the mixture is heated at 120 °C for 2 hours. The reaction is cooled down to room temperature, then 5% HCl (20 mL) is added (final pH∼2). After 1 hour stirring at room temperature, the mixture is filtered on a Hirsh funnel. The solid is taken up with EtOH/water 1:5 mixture (12 mL), filtered and dried until weight stabilization. 2.244 g of pure product (8.240 mmol, 54.9% yield) are recovered as a bright orange powder. mp 155-156 °C

^{1}H NMR (400 MHz, $CDCl_3$) δ[ppm] 8.31 (br, 2H), 8.16 (dd, J = 8.7, 1.7 Hz, 1H), 7.33 (ddd, J = 8.6, 7.0, 1.5 Hz, 1H), 7.19 (dd, J = 8.7, 0.9 Hz, 1H), 6.57 (ddd, J = 8.7, 6.8, 1.1 Hz, 1H), 2.18 (m, 3H), 2.10 (d, J = 2.7 Hz, 6H), 1.75 (m, 6H).[S16]

1.5.2 Synthesis of derivative 6n

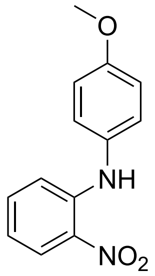

In a 100 mL roundbottom flask, 2-fluoronitrobenzene (4.000 g, 28.35 mmol) and p-anisidine (3.492 g, 28.36 mmol) are put under nitrogen atmosphere. Triethanolamine (4.229 g, 28.35 mmol) is added, and the mixture is heated at 120 °C for 5 hours. The reaction is cooled down to room temperature, then water (30 mL) is added, and the mixture is acidified by addition of 5% HCl (final pH∼2). After 2 hours stirring at room temperature, the mixture is filtered on a Büchner funnel, and the solid is dried until weight stabilization. 5.930 g of pure product (24.28 mmol, 85.6% yield) are recovered as a red-purple powder. mp 88-90 °C

^{1}H NMR (400 MHz, $CDCl_3$) δ[ppm] 9.40 (br, 1H), 8.19 (dd, J = 8.7, 1.5 Hz, 1H), 7.32

(ddd, J = 8.6, 7.0, 1.5 Hz, 1H), 7.20 (d, J = 8.9 Hz, 2H), 7.00 (dd, J = 8.7, 1.0 Hz, 1H), 6.96 (d, J = 8.9 Hz, 2H), 6.71 (ddd, J = 8.6, 7.0, 1.2 Hz, 1H), 3.84 (s, 3H).[S17]

1.6 Synthesis of N-alkyl-2-arylbenzimidazoles

1.6.1 Synthesis of derivative 7a

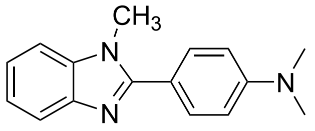

In a 50 mL roundbottom flask, N-methyl-2-nitroaniline (4.000 g, 25.29 mmol), 4-dimethylaminobenzaldehyde (3.775 g, 25.30 mmol) and $Na_2S_2O_4$ (85%, 13.989 g, 68.294 mmol) are put under N_2 atmosphere, then ethanol (20 mL) is added. Reaction is heated at 100 °C for 24 hours, then 5 mL of DMSO are added and heating is prolonged for 24 hours more. The mixture is cooled down to room temperature, moved in a 250 mL beacker, then 50 mL of 3% NH_3 are added, and the mixture is stirred for 30 minutes. The solid is filtered on a Hirsh funnel and washed with water (10 mL). The off white solid is dried in vacuum at 65 °C until weight stabilization (6.289 g, 25.02 mmol, 98.9% yield). mp 150-156 °C

^{1}H NMR (400 MHz, $CDCl_3$) δ[ppm] 7.79-7.75 (m, 1H), 7.65 (d, J= 8.9 Hz, 2H), 7.33-7.30 (m, 1H), 7.27-7.23 (m, 2H), 6.77 (d, J = 8.9 Hz, 2H), 3.83 (s, 3H), 3.01 (s, 6H).[S18]

Note: on this small scale, the amount of water naturally present in EtOH and DMSO was enough to bring the reaction to completion.

1.6.2 Synthesis of derivative 7b

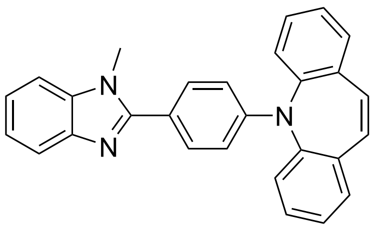

In a 50 mL roundbottom flask, N-methyl-2-nitroaniline (270 mg, 1.77 mmol), derivative **4b** (498 mg, 1.67 mmol), and $Na_2S_2O_4$ (85%, 928 mg, 4.53 mmol), then ethanol (3 mL), water (0.3 mL) and DMSO (0.75 mL) are added. Reaction is heated at 100 °C for 4 hours and 30 minutes, at which point conversion is complete. The mixture is cooled down to room temperature, then 10 mL of 3% NH_3 are added, and the mixture is stirred for 30 minutes. The solid is filtered on a Hirsh funnel, washed with 10 mL of 3% NH_3 and then with water. The procedure is repeated once and the obtained white solid is dried in vacuum at 45 °C

until weight stabilization (0.612 g, 1.53 mmol, 91.7% yield). mp 240-242 °C. Anal. Calcd for $C_{28}H_{21}N_3$: C, 84.18; H, 5.30; N, 10.52. Found: 83.95; H, 5.58; N, 10.39.

^{1}H NMR (400 MHz, DMSO$-d_6$): δ[ppm] 7.65-7.57 (m, 7H), 7.53-7.46 (m, 5H), 7.23-7.15 (m, 2H), 6.98 (s, 2H), 6.28 (d, J = 9.0 Hz, 2H), 3.78 (s, 3H).

^{13}C{^{1}H} NMR (100 MHz, DMSO$-d_6$): δ[ppm] 153.33, 149.39, 142.52, 141.75, 136.60, 135.74, 130.61, 130.40, 130.21, 129.92, 129.90, 127.67, 121.72, 121.58, 119.42, 118.46, 111.07, 110.14, 31.69.

Alternatively, the product can be prepared by Buchwald-Hartwig amination: Before the reaction beginning, anisole is carefully degassed by freeze-pump-thaw. In a 50 mL roundbottom flask, derivative **7r** (1.000 g, 3.482 mmol), iminostilbene (740 mg, 3.83 mmol), K_3PO_4 (1.119 g, 5.223 mmol) and poly(ethylene glycol) dimethyl ether (MW = 2000, 150 mg) are put under N_2 atmosphere. In a second flask, $Pd(OAc)_2$ (7.82 mg, 0.0348 mmol) and XPhos (33.2 mg, 0.0696 mmol) are dissolved under N_2 atmosphere in 1.0 mL of anisole at 60 °C. The solution is transferred in the first flask, and the mixture is heated at 125 °C. 0.5 mL of anisole are then added, and the mixture is kept at 125 °C for 6.5 h. After cooling down to room temperature, 30 mL of water are added and the mixture is acidified by addition of citric acid. The solid is filtered on a Hirsh funnel, and subsequently refluxed in 50 mL of EtOH. The solid is filtered, then refluxed in 2 mL of Et_2O, and filtered again. The obtained off white solid is dried in vacuum at 65 °C until weight stabilization (720 g, 1.802 mmol, 51.8% yield).

1.6.3 Synthesis of derivative 7c

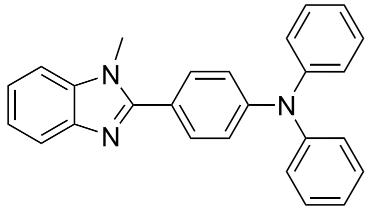

In a 50 mL two neck roundbottom flask, N-methylnitroaniline (1.217 g, 8,000 mmol), 4-(N,N-Diphenylamino)benzaldehyde (2.062 g, 7.545 mmol) and $N_2S_2O_4$ (3.776g, 21.69 mmol), then ethanol (9.5 mL) and water (0.8 mL) are added. The system is put under N_2 and the reaction is heated under reflux for 23 hours. The reaction is stopped and cooled down to room temperature, then 32 mL of 3% NH_3 are added. A precipitate forms, which is then filtered on a Hirsh funnel. The obtained solid is then taken up with methanol, filtered and dried under reduced pressure at 40 °C until weight stabilization. Brownish solid, 1.765 g, 4.700 mmol, 62.3% yield. mp: 167-170 °C.

^1H NMR (400 MHz, $CDCl_3$): δ[ppm] 7.81-7.79 (m, 1H), 7.63 (d, J = 7.6 Hz, 2H), 7.39-7.37 (m, 1H), 7.32-7.28 (m, 6H), 7.18-7.16 (m, 6H), 7.09 (t, J = 7.4 Hz, 2H), 3.89 (s, 3H).[S18]

1.6.4 Synthesis of derivative 7d

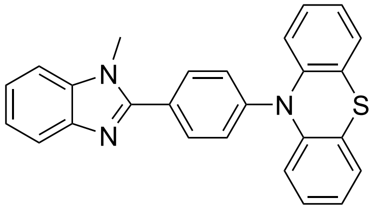

In a 50 mL roundbottom flask, N-methyl-2-nitroaniline (161 mg, 1.06 mmol), derivative **4d** (302 mg, 0.995 mmol), and $Na_2S_2O_4$ (85%, 547 mg, 2.67 mmol), then ethanol (4 mL), water (0.5 mL) and DMSO (1 mL) are added. The mixture is heated at 100 °C for 1 hour, then 0.5 mL of water are added. Reaction progress can be monitored by TLC (Heptane/AcOEt 7:3). The mixture is kept at 100 °C for 2 more hours, at which point conversion is complete. The mixture is cooled down to room temperature, then 50 mL of 3% NH_3 are added and the mixture is stirred for 30 minutes. The solid is filtered on a Hirsh funnel and washed with water (10 mL). The powder is then taken up with methanol (5 mL) and filtered. The pale yellow solid is dried in vacuum at 65 °C until weight stabilization (242 g, 0.597 mmol, 60.0% yield). mp 211-215 °C . Anal. Calcd for $C_{26}H_{19}N_3S$: C, 77.01; H, 4.72; N, 10.36. Found: 76.85; H, 4.84; N, 10.23.

^1H NMR (400 MHz, DMSO$-d_6$): δ[ppm] 8.06 (d, J = 8.5 Hz, 2H), 7.70 (d, J = 7.8 Hz,

1H), 7.64 (d, J = 7.9 Hz, 1H), 7.51 (d, J = 8.4 Hz, 2H), 7.33 - 7.26 (m, 2H), 7.23 (dd, J = 7.6, 1.3 Hz, 2H), 7.12-7.07 (m, 2H), 7.01-6.98 (m, 2H), 6.59 (d, J = 8.0 Hz, 2H), 3.96 (s, 3H).

$^{13}C\{^1H\}$ NMR (100 MHz, DMSO$-d_6$): δ[ppm] 152.32, 142.88, 142.60, 142.50, 136.68, 131.60, 128.41, 127.50, 127.23, 127.15, 123.74, 122.64, 122.43, 122.00, 119.01, 118.71, 110.61, 31.81.

Alternatively, the product can be prepared by Buchwald-Hartwig amination: Before the reaction beginning, anisole is carefully degassed by freeze-pump-thaw. In a 50 mL roundbottom flask, derivative **7r** (745 mg, 2.59 mmol), phenothiazine (659 mg, 3.31 mmol), K_3PO_4 (858 mg, 4.04 mmol) and poly(ethylene glycol) dimethyl ether (MW = 2000, 71 mg) are put under N_2 atmosphere. In a second flask, $Pd(OAc)_2$ (5.86 mg, 0.0261 mmol) and XPhos (25.0 mg, 0.0524 mmol) are dissolved under N_2 atmosphere in 1.0 mL of anisole at 60 °C. The solution is transfered in the flask containing all the other reagents, anf the mixture is heated at 135 °C. 0.5 mL of anisole are then added, and the mixture is kept at 135 °C for 7 h, and subsequently temperature is raised to 150 °C for 2 h. After cooling down to room temperature, 50 mL of water are added and the mixture is acidified by addition of citric acid. The solid (933 mg) is filtered on a Hirsh funnel, and subsequently refluxed in 10 mL of AcOEt and filtered again. The obtained off white solid is dried in vacuum at 65 °C until weight stabilization (503 g, 1.24 mmol, 47.9% yield).

1.6.5 Synthesis of derivative 7e

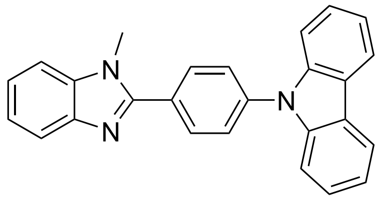

In a 25 mL roundbottom flask, N-methyl-2-nitroaniline (145 mg, 0.953 mmol), derivative **4e** (228 mg, 0.840 mmol), $Na_2S_2O_4$ (85%, 465 mg, 2.27 mmol) are added. H_2O (0.10 mL) and Ethanol (1.00 mL) are added to the flask and the mixture is heated under reflux. DMSO (0.25 mL) is added to the flask and a yellow precipitate forms progressevely. The reaction is monitored via TLC using a mixture of acetate and heptane (1:1) as eluent. After 4.5 hours conversion of A3 is complete and the reaction is stopped. 5 mL of ethanol are added to the mixture, which is then heated under reflux. 10 mL of a 3% solution of NH_3 are then added to the flask. the mixture is then cooled down to room temperature and the obtained white dispersion is filtered on a Hirsh funnel. The product is recovered as a gray powder and is dried under vacuum at 65 °C until weight stabilization (263 mg, 0.704 mmol, 83.8 % yield). mp 174-176 °C.

^1H NMR (400 MHz, DMSO−d_6): δ[ppm] 8.29 (d, J = 7.7 Hz, 2H), 8.17 (d, J=8.4 Hz, 2H), 7.86 (d, J = 8.4 Hz, 2H), 7.74 (d, J = 7.6 Hz, 1H), 7.67 (d, J = 7.8 Hz, 1H), 7.55 (d, J = 8.2 Hz, 2H), 7.51-7.45 (m, 2H), 7.38-7.25 (m, 4H), 4.02 (s, 3H).[S18]

Alternatively, the product can be prepared by Buchwald-Hartwig amination: Before the reaction beginning, anisole is carefully degassed by freeze-pump-thaw. In a 50 mL roundbottom flask, derivative **7r** (0.800 g, 2.78 mmol), carbazole (513 mg, 3.06 mmol), K_3PO_4 (0.887 g, 4.18 mmol) and poly(ethylene glycol) dimethyl ether (MW = 2000, 80 mg) are put under N_2 atmosphere. In a second flask, $Pd(OAc)_2$ (6.20 mg, 0.0278 mmol) and XPhos (26.5 mg, 0.0557 mmol) are dissolved under N_2 atmosphere in 1.0 mL of anisole at 60 °C. The solution is transferred in the flask containing all the other reagents, and the mixture is heated at 125 °C for 2 hours. Reaction progress is monitored via TLC using a mixture of heptane:AcOEt 8:2 as eluent. After cooling down to room temperature, the

residual solvent is evaporated under reduced pressure. 25 mL of water are added to the crude and the mixture is acidified by addition of citric acid. After 30 minutes of sonication in an ultrasonic bath, the suspension is filtered on a Hirsh funnel. The recovered solid is suspended again in ethanol, sonicated in an ultrasonic bath and filtered to give a beige solid which is dried under reduced pressure at 65 °C until weight stabilization (0.710 g, 1.90 mmol, 68.3% yield).

1.6.6 Synthesis of derivative 7f

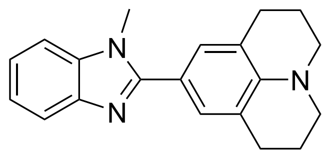

In a two neck 100 mL roundbottom flask, N-methyl-2-nitroaniline (1.500 g, 9.868 mmol), 9-julolidinecarboxaldehyde (1.984 g, 9.857 mmol), $Na_2S_2O_4$ (85%, 5.354 g, 26.14 mmol) are added.The system is put under N_2 and a Ethanol:H_2O 5:1 mixture is added to the system. The obtained suspension is put at 88 °C and progressively turns from orange to yellow. After 19 hours the reaction is stopped and the solid is hot filtered and washed with ethanol. The recovered powder is taken up with 150 mL of a 3% NH_3 solution and the obtained mixture is left stirring until a pale yellow suspension forms. The suspension is filtered on a Hirsch funnel and the filtrated is washed with 1% NH_3 solution and then with water. The recovered solid is dried under reduced pressure 50 °C to afford 2.23 g of product (7.34 mmol, 74.5% yield). mp: does not melt, degradation above 150 °C. Anal. Calcd for $C_{20}H_{21}N_3$: C, 79.17; H, 6.98; N, 13.85. Found: 79.09; H, 7.09; N, 13.77.

^{1}H NMR (400 MHz, DMSO$-$d$_6$): δ[ppm] 7.58-7.55 (m, 1H), 7.52-7.50 (m, 1H), 7.23 (s, 2H), 7.21-7.16 (m, 4H), 3.84 (s, 3H), 3.21 (t, J = 5.7 Hz, 4H), 2.76 (t, J = 6.3 Hz, 4H), 1.91 (quin, J = 6.0 Hz, 4H).

^{13}C{^{1}H} NMR (100 MHz, DMSO$-$d$_6$): δ[ppm] 154.13, 143.59, 142.61, 136.67, 127.62, 121.47, 121.41, 120.46, 118.17, 115.87, 109.96, 49.18, 31.78, 27.19, 21.27.

1.6.7 Synthesis of derivative 7h

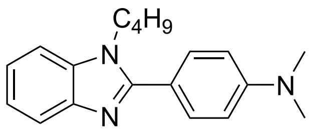

In a 2 neck 100 mL roundbottom flask, 2-fluoronitrobenzene (2.137 g, 15.14 mmol), and butylamine (1.153 g, 15.76 mmol) are added, followed by DMSO (4 mL). The mixture is heated at 100°C for 1 hour to reach complete conversion. The reaction is then let cool down to room temperature. 4-dimethylaminobenzaldehyde (2.248 g, 15.07 mmol) is dissolved in ethanol (16 mL) and the obtained solution is added to the reaction, followed by $Na_2S_2O_4$ (7.875 g, 45.23 mmol) and water (0.5 mL). The re-action is heated under reflux and a yellow suspension forms. The reaction progress is monitored via TLC using a mixture of heptane/acetate 1:1 as eluent. After 17 hours conversion of the reagents is complete and the reaction is stopped. The mixture is moved in a 250 mL beaker and 70 mL of a 3% NH3 solution are added. The mixture is let stirring for 30 minutes. A waxy solid separates from the aqueous phase. The solid is recovered via filtration and is redispersed in a AcOEt/heptane 1:1 mixture. A suspension of a white solid is obtained and filtered on a Hirsh funnel to give a first product fraction. The residual solvent is evaporated under reduced pressure and the obtained solid is redispersed in 4 mL of Et_2O. The dispersion is let rest at -18 °C for 15 hours and the solid is recovered by filtration. The obtained powder is refluxed in a 5% NH3 solution for 15 minutes, and the dispersion is let cooling down to room temperature. The white solid is recovered via filtration to give a second solid fraction. Both fractions are dried in vacuum at 40°C until weight stabilization to afford 3.000 g of product (10.23 mmol, 67.9% yield). mp 80-82 °C. Anal. Calcd for $C_{19}H_{23}N_3$: C, 77.78; H, 7.90; N, 14.32. Found: 77.72; H, 7.98; N, 14.25.

^{1}H NMR (400 MHz, $CDCl_3$): δ[ppm] 7.83-7.80 (m, 1H), 7.64 (d, J = 8.9 Hz, 2H), 7.39-7.37 (m, 1H), 7.28-7.27 (m, 2H), 6.81 (d, J = 8.9 Hz, 2H), 4.24 (t, J = 7.7 Hz, 2H), 3.04 (s, 6H), 1.89-1.81 (m, 2H), 1.38-1.29 (m, 2H), 0.91 (t, J = 7.40, 3H).

^{13}C{^{1}H} NMR (100 MHz, $CDCl_3$): δ[ppm] 154.69, 151.22, 143.44, 135.97, 130.38, 122.05, 119.60, 118.02, 111.94, 109.92, 44.75, 40.38, 32.04, 20.20, 13.79.

1.6.8 Synthesis of derivative 7l

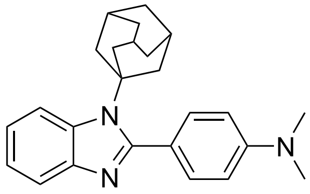

In a 100 mL roundbottom flask, derivative **6l** (1.365 g, 5.012 mmol), 4-dimethylaminobenzaldehyde (0.750 g, 5.03 mmol) are dissolved in EtOH (6.0 mL), then water (0.5 mL) and $Na_2S_2O_4$ (85%, 2.770 g, 13.52 mmol) are added. Reaction is refluxed for 16 hours, turning from orange to a yellow dispersion. Reaction progress can be monitored by TLC (eluent AcOEt/heptane 1:1). After cooling down to room temperature, 20 mL of 3% NH_3 are added, the mixture is stirred for 30 minutes, then the solid is filtered on a Hirsh funnel and washed with water (5 mL). The yellow solid is taken up with heptane to remove residual unreacted 4-dimethylaminobenzaldehyde, and filtered again. The light yellow powder if finally dried in vacuum at 65 °C until weight stabilization (1.520 g, 4.091 mmol, 81.6% yield). mp: degradation at T >185 °C. Anal. Calcd for $C_{25}H_{29}N_3$: C, 80.82; H, 7.87; N, 11.31. Found: 80.89; H, 7.95; N, 11.12.

^{1}H NMR (400 MHz, $CDCl_3$) δ[ppm] 7.80-7.74 (m, 2H), 7.29 (d, J = 8.8 Hz, 2H), 7.25-7.18 (m, 2H), 6.70 (d, J = 7.7 Hz, 2H), 3.00 (s, 6 H), 2.29 (m, 6H), 2.14 (m, 3H), 1.70 (m, 6H).

^{13}C{^{1}H} NMR (100 MHz, $CDCl_3$): δ[ppm] 154.54, 150.75, 143.62, 134.45, 130.63, 123.91, 121.56, 121.30, 120.23, 115.47, 111.30, 60.82, 43.00, 40.56, 36.18, 30.17.

1.6.9 Synthesis of derivative 7m

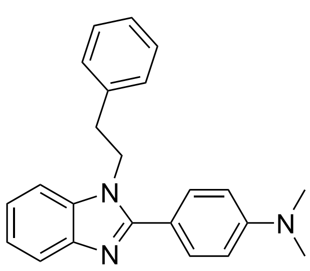

In a 250 mL roundbottom flask, 1-fluoro-2-nitrobenzene (5.000 g, 35.426 mmol) and 2-phenetylamine (4.294 g, 35.436 mmol) are added and the system is put under N_2 atmosphere. 10 mL of DMSO are added to the flask and the mixture is heated at 100°C. After 1 hour, the reaction is complete. The mixture is cooled down to room temperature. A solution of 4-(dimethylamino)benzaldehyde (5.287 g, 35.436 mmol) in ethanol (40 mL) and $Na_2S_2o_4$ (18.51 g, 106.31 mmol) are then added to the flask and the mixture is heated at 80°C. After 20

hours, the reaction is stopped and cooled down to room temperature, then 50 mL of 3% NH_3 are added. A precipitate forms, which is then filtered on a Hirsh funnel and washed with further NH_3 solution. The recovered solid is dried under reduced pressure at 90°C. A fraction of the solid (2g) is crystallized in a H_2O:Ethanol (3:2) mixture (4 mL) basified with NH_3 to afford 1.063 g of product (3.114 mmol). The rest of the crude (12.379g) is taken up with 55 mL of a 3% NH_3 solution. The obtained mixture is filtered and the recovered solid is dried under reduced pressure at 80°C to afford further 6.970 g of product (20.412 mmol). Reaction yield: 67.2%. m.p. 134-136 °C. Anal. Calcd for $C_{23}H_{23}N_3$: C, 80.90; H, 6.79; N, 12.31. Found: 80.85; H, 6.84; N, 12.27.

^{1}H NMR (400 MHz, DMSO$-d_6$) δ[ppm] 7.61 (dd, J = 6.7, 2.3 Hz, 2H), 7.50 (d, J = 8.8 Hz, 2H), 7.25-7.16 (m, 5H), 7.09-7.07 (m, 2H), 6.82 (d, J = 8.8 Hz, 2H), 4.47 (t, J = 7.6 Hz, 2H), 3.03 (t, J = 7.6 Hz, 2H), 3.00 (s, 6H).

^{13}C{^{1}H} NMR (100 MHz, DMSO$-d_6$): δ[ppm] 153.77, 150.83, 142.67, 137.81, 135.58, 129.86, 128.62, 128.36, 126.49, 121.69, 121.57, 118.53, 117.20, 111.58, 110.55, 45.60, 39.77, 34.99.

1.6.10 Synthesis of derivative 7n

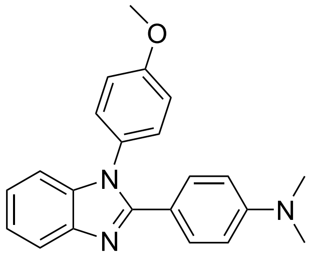

In a 50 mL roundbottom flask, derivative **6n** (2.000 g, 8.188 mmol), 4-dimethylaminobenzaldehyde (1.222 g, 8.188 mmol) and $Na_2S_2O_4$ (85%, 4.530 g, 22.11 mmol) are put under N_2 atmosphere, then ethanol (10 mL) and DMSO (2.5 mL) are added. Reaction is heated at 100 °C for 18 hours, then cooled down to room temperature. 55 mL of 3% NH_3 are added, and the mixture is stirred for 30 minutes, then the solid is filtered on a Hirsh funnel and washed with further 3% NH_3 (10 mL). The off white solid is dried in vacuum at 65 °C until weight stabilization (2.574 g, 7.495 mmol, 91.5% yield). mp 158-160 °C. Anal. Calcd for $C_{22}H_{21}N_3O$: C, 76.94; H, 6.16; N, 12.24. Found: 76.88; H, 6.20; N, 12.19.

^{1}H NMR (400 MHz, DMSO$-d_6$) δ[ppm] 7.68 (d, J = 7.8 Hz, 1H), 7.38 (d, J = 9.0 Hz, 2H), 7.34 (d, J = 8.9 Hz, 2H), 7.25 (ddd, J = 8.2, 7.2, 1.2 Hz, 1H), 7.19 (ddd, J = 8.2, 7.2, 1.1 Hz, 1H), (t, J = 7.6 Hz, 1H), 7.12 (d, J = 8.9 Hz, 2H), 7.04 (d, J = 7.8 Hz, 1H), 6.64 (d, J = 8.9 Hz, 2H), 3.85 (s, 3H), 2.92 (s, 6H).

$^{13}C\{^{1}H\}$ NMR (100 MHz, DMSO$-d_6$): δ[ppm] 159.07, 152.67, 150.69, 142.63, 137.64, 129.85, 129.63, 128.83, 122.24, 122.16, 118.51, 116.61, 115.14, 111.30, 109.94, 55.44, 39.65.

Note for reaction scaling up: on the small scale, the amount of water naturally present in EtOH and DMSO was enough to bring the reaction to completion. Repeating the reaction on a bigger scale, addition of water was necessary in order to observe product formation. Reagents amount: derivative **6n** (17.000 g), 4-dimethylaminobenzaldehyde (10.383 g), $Na_2S_2O_4$ (85%, 38.490 g), EtOH (80 mL), DMSO (20 mL), H_2O (10 mL).

1.6.11 Synthesis of derivative 7r

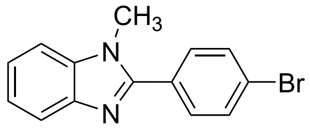

In a 50 mL roundbottom flask, N-methyl-2-nitroaniline (4.000 g, 25.29 mmol), 4-bromobenzaldehyde (4.681 g, 25.30 mmol) and $Na_2S_2O_4$ (85%, 13.989 g, 68.294 mmol) are put under N_2 atmosphere, then ethanol (20 mL) and DMSO (5 mL) are added. The reaction is heated at 100 °C for 7 hours, at which point conversion is complete. The mixture is cooled down to room temperature, moved in a 250 mL becker, then 50 mL of 3% NH_3 are added, and the mixture is stirred for 30 minutes. The solid is filtered on a Hirsh funnel and washed with water (10 mL). The off white solid is dried in vacuum at 65 °C until weight stabilization (7.116 g, 24.78 mmol, 98.0% yield). mp 108-111 °C.

^{1}H NMR (400 MHz, $CDCl_3$) δ[ppm] 7.84-7.82 (m, 1H), 7.69-7.64 (m, 4H), 7.41-7.31 (m, 3H), 3.86 (s, 3H).[S19]

^{1}H NMR (400 MHz, DMSO$-d_6$) δ[ppm] 7.84-7.77 (m, 4H), 7.69 (m, 1H), 7.63 (m, 1H), 7.31 (m, 1H), 7.26 (m, 1H), 3.86 (s, 3H).

Note: on this small scale, the amount of water naturally present in EtOH and DMSO was enough to bring the reaction to completion.

1.7 Synthesis of N,N′-diakyl-2-aryl-benzimidazolium salt

1.7.1 Synthesis of derivative 8a as triflate salt

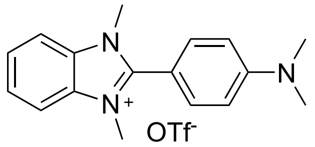

In a two neck, 50 mL flask, derivative **7a** (307 mg, 1.22 mmol) is added and the system is put under N_2 atmosphere. Anhydrous toluene (18 mL) is added to the flask and a pale yellow dispersion is obtained. Methyl triflate (197 mg, 1.20 mmol) is added to the mixture and the suspension turns white. After 2 hours, further 145 mg (0.883 mmol) of methyl triflate are added. The reaction is stopped after an overall time of 3 hours and the suspension is cooled down to room temperature. The mixture is filtered on a Hirsh funnel and the recovered solid is dried under reduced pressure at 40°C. The obtained powder is crystallized twice in anisole to afford 230 mg of product as gray powder (0.554 mmol, 45.4% yield). Presence of residual reagent and the corresponding byproduct **9** lowers the product purity to ∼93%.

^{1}H NMR (400 MHz, DMSO−d_6): δ[ppm] 8.05 (dd, J = 6.2, 3.2 Hz, 2H), 7.71 (dd, J = 6.2, 3.2, 2H), 7.67 (d, J = 8.9 Hz, 2H), 6.98 (d, J = 9.0 Hz, 2H), 3.91 (s, 6H), 3.08 (s, 6H).[S20]

1.7.2 Synthesis of derivative 8b as iodide salt

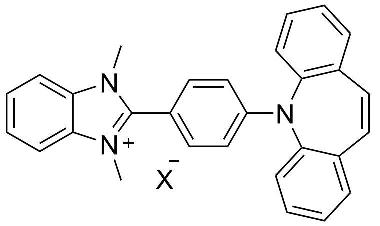

Derivative **7b** (193 mg, 0.483 mmol) is added to a two neck, 50 mL roundbottom flask. The system is put under N_2 and anhydrous acetonitrile (5 mL) is added to obtain a white suspension. Methyl iodide (82.29 mg, 0.580 mmol) is added and the reaction is refluxed to obtain a solution. The reaction is stopped after 5 hours, let cool down and left at room temperature for 18 hours. The product crystallizes in brownish crystals which are filtered on a Hirsh funnel. The recovered acetonitrile is then evaporated under reduced pressure and the obtained solid is washed with toluene. Both solid fractions are collected and dried under

vacuum at 65 °C to give 210 mg of product (0.388 mmol, 80.3% yield). mp: degradation at T >257 °C.

^{1}H NMR and ^{13}C{^{1}H} NMR are identical to those of the triflate salt (vide infra).

1.7.3 Synthesis of derivative 8b as triflate salt

In a two neck 50 mL roundbottom flask, derivative **7b** (209 mg, 0.523 mmol) is added. The system is put under N_2 atmosphere. Anhydrous acetonitrile (10 mL) is added to the flask to obtain a suspension. Methyl triflate (113 mg, 0.689 mmol) is then added to the mixture and a solution is obtained. The reaction is stopped after 1 hour and 30 minutes. The solvent is evaporated under reduced pressure and the obtained solid is washed with toluene and water and is then dried under reduced pressure at 65 °C until weight stabilization (261 mg, 0.463 mmol, 88.5% yield). mp: degradation at >210 °C.

^{1}H NMR (400 MHz, DMSO−d_6): δ[ppm] 8.03 (dd, J = 6.2, 3.1 Hz, 2H), 7.69 (dd, J = 6.2, 3.1 Hz, 2H), 7.66-7.63 (m, 6H), 7.54-7.50 (m, 4H), 7.03 (s, 2H), 6.42 (d, J = 9.1 Hz, 2H), 3.84 (s, 6H).[S3]

^{13}C{^{1}H} NMR (100 MHz, DMSO−d_6): δ[ppm] 151.47, 150.89, 140.76, 135.32, 131.97, 131.69, 130.64, 130.37, 129.40, 128.02, 126.27, 113.04, 111.25, 108.79, 32.83.

^{19}F NMR (400 MHz, DMSO−d_6): δ[ppm] -77.74.

1.7.4 Synthesis of derivative 8c as iodide salt

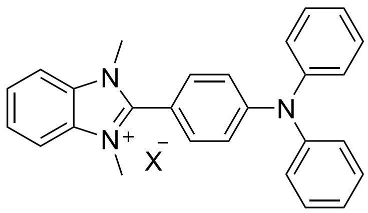

Derivative **7c** (1.739 mg, 4.632 mmol) is added to a two neck 100 mL roundbottom flask. The system is provided of a condenser and put under N_2 atmosphere. 13 mL of anhydrous toluene are added to the flask and the mixture is heated at 80°C to obtain a suspension. Methyl iodide (628 mg, 4.42 mmol) is then added to the reaction. The reaction progress is monitored via TLC using a mixture of dichloromethane and ethyl acetate (9:1 vol:vol) as eluent. After 26 hours

other 215 mg of methyl iodide (1.51 mmol) are added to the mixture. After 4 days, the reaction is stopped and the obtained precipitate is filtered on a Hirsh funnel. Once dried under vacuum at 65 °C, the obtained solid is washed with toluene (10 mL) under reflux, hot filtered and dried again until weight stabilization to give 1.872 g of product as a pale yellow powder (3.618 mmol, 78.1% yield). mp: does not melt, degradation at T >242 °C. Anal. Calcd for $C_{27}H_{24}IN_3 \cdot \frac{1}{2}H_2O$: C, 61.60; H, 4.79; N, 7.98. Found: 61.41; H, 4.83; N, 7.88.

^{1}H NMR (400 MHz, DMSO$-d_6$): δ[ppm] 8.08 (dd, J = 6.2, 3.1 Hz, 2H), 7.72 (dd, J= 6.2, 3.1 Hz, 2H), 7.69 (d, J=8.9 Hz, 2H), 7.48-7.44 (m, 4H), 7.28-7.24 (m ,6H), 7.06 (d, J=8.9 Hz, 2H), 3.92 (s, 6H).

^{13}C{^{1}H} NMR (100 MHz, DMSO$-d_6$): δ[ppm] 151.10, 150.64, 145.50, 132.16, 131.76, 130.11, 126.41, 126.36, 125.51, 118.43, 113.18, 111.00, 32.86.

1.7.5 Synthesis of derivative 8d as iodide salt

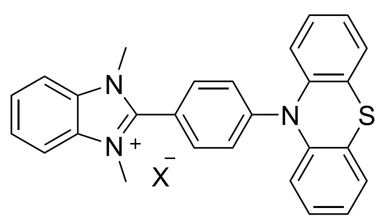

Derivative **7d** (240 mg, 0.592 mmol) is added to a 2 neck, 50 mL roundbottom flask. The system is put under N_2 and anhydrous acetonitrile (5 mL) is added, followed by methyl iodide (161.3 mg, 1.136 mmol). The reaction is refluxed with the formation of a yellow precipitate. After 23 hours, the reaction is stopped and the precipitate is filtered on a Hirsh funnel and washed with toluene. The recovered solution is evaporated under reduced pressure to obtain a second solid fraction which is washed with toluene and then filtered on a Hirsh funnel. Both solid fractions are recovered and dried under vacuum at 65 °C to afford the product as a pale yellow powder (275 mg, 0.502 mmol, 84.7% yield). mp: Degradation at T >260 °C. Anal. Calcd for $C_{27}H_{22}IN_3S$: C, 59.24; H, 4.05; N, 7.68. Found: 59.01; H, 4.21; N, 7.55.

^{1}H NMR (400 MHz, DMSO$-d_6$): δ[ppm] 8.11 (dd, J = 6.2, 3.1 Hz, 2H), 7.85 (d, J = 8.8 Hz, 2H), 7.75 (dd, J = 6.2, 3.1 Hz, 2H), 7.52 (dd, J = 7.8, 1.2 Hz, 2H), 7.40-7.36 (m, 4H), 7.30-7.24 (m, 4H), 3.92 (s, 6H).

$^{13}C\{^1H\}$ NMR (100 MHz, DMSO−d_6): δ[ppm] 150.31, 147.71, 140.96, 132.77, 131.76, 129.76, 128.44, 127.83, 126.50, 125.96, 124.54, 119.00, 114.33, 113.25, 32.85.

1.7.6 Synthesis of derivative 8d as triflate salt

Derivative **7d** (251 mg, 0.616 mmol) is added to a 2 neck, 50 mL roundbottom flask. The system is put under N_2 and anhydrous acetonitrile (10 mL) is added, followed by methyl triflate (161.3 mg, 0.676 mmol). The obtained solution is left stirring at room temperature. A precipitate forms and after 3 hours the reaction is stopped. The reaction is quenched with 0.3 mL of methanol and the suspension is filtered on a Hirsh funnel to give a grey powder. The recovered liquid phase is evaporated under reduced pressure and the obtained solid is washed with 5 mL of a 4:1 (vol:vol) methanol/toluene mixture and then with 5 mL of toluene. Both solid fractions are then dried under vacuum at 65 °C to afford the product as a gray powder (241 mg, 0.423 mmol, 68.7% yield). Degradation at T >260 °C.

^1H NMR and $^{13}C\{^1H\}$ NMR are identical to those of the iodide salt.

^{19}F NMR (400 MHz, DMSO−d_6): δ[ppm] -77.76

1.7.7 Synthesis of derivative 8e as iodide salt

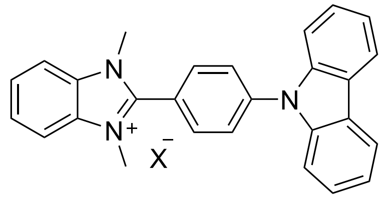

Derivative G5-3 (207 mg, 0.554 mmol) is added to a 2 neck, 50 mL roundbottom flask. The system is put under N_2 and anhydrous acetonitrile (5 mL) is added, followed by methyl iodide (150.8 mg, 1.062 mmol). The reaction is refluxed with the formation of a precipitate. After 18 hours, the reaction is stopped and the precipitate is filtered on a Hirsh funnel. The recovered solid is dried under vacuum at 65 °C to afford the product as a brownish powder (245 mg, 0.475 mmol, 85.8% yield). mp: degradation at T >305 °C.

^1H NMR and $^{13}C\{^1H\}$ NMR are identical to those of the triflate salt (vide infra).

1.7.8 Synthesis of derivative 8e as triflate salt

Derivative **7e** (314 mg, 0.840 mmol) is added to a 2 neck, 100 mL roundbottom flask. The system is put under N_2 and anhydrous acetonitrile (20 mL) is added, followed by methyl triflate (300 mg, 1.83 mmol). The obtained solution is left stirring at room temperature. The reaction is stopped after 2 hours and is quenched with addition of 1 mL of ethanol. The solvent is evaporated under reduced pressure and the obtained solid is first washed with toluene and then with water. The obtained powder is dried under vacuum at 65 °C to afford the product as a grey powder (408 mg, 0.759 mmol, 90.3% yield). mp: degradation at T>284 °C. Anal. Calcd for $C_{28}H_{22}F_3N_3O_3S \cdot H_2O$: C, 61.53; H, 4.35; N, 7.56. Found: 61.25; H, 4.48; N, 7.46.

^{1}H NMR (400 MHz, DMSO−d_6): δ[ppm] 8.31 (d, J = 7.8 Hz, 2H), 8.20-8.16 (m, 4H), 8.10 (d, J = 8.6 Hz, 2H), 7.80 (dd, J = 6.2, 3.1 Hz, 2H), 7.64 (d, J = 8.24, 2H), 7.53-7.49 (m, 2H), 7.39-7.35 (m, 2H), 4.04 (s, 6H).

^{13}C{^{1}H} NMR (100 MHz, DMSO−d_6): δ[ppm] 149.84, 140.89, 139.58, 132.90, 131.84, 127.22, 126.71, 126.45, 123.20, 120.78, 120.70, 119.38, 113.43, 109.89, 32.90.

^{19}F NMR (400 MHz, DMSO−d_6): δ[ppm] -77.72

1.7.9 Synthesis of derivative 8f as iodide salt

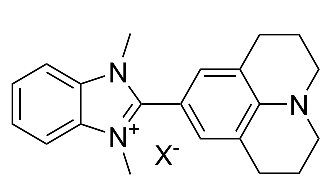

In a two neck, 100 mL roundbottom flask, derivative **7f** (537 mg, 1.77 mmol) is added. The system is put under N_2 atmosphere and acetone (20 mL, dried over $CaSO_4$) is added to the flask. A yellow suspension forms. The mixture is put under reflux and Methyl iodide (296 mg, 2.08 mmol) is added to the flask. After 1 hour and 20 minutes, the reaction is stopped and the mixture is left to cool down to room temperature. The mixture is filtered on a Hirsh funnel and the obtained yellow solid is washed with acetone and subsequently dried under reduced pressure to afford a first fraction of the product (340 mg, 0.763 mmol). The recovered acetone is evaporated under reduced pressure

to give a second solid fraction, which is hot filtered from Et_2O (15 mL) on a hirsh funnel and washed with further 15 mL of cool Et_2O. The procedure is repeated three more times to afford a second solid fraction, which is dried under reduced pressure to afford 148 mg (0.332 mmol) of product. Overall reaction yield: 61.9%. m.p. : does not melt, degradation at T >220 °C. Anal. Calcd for $C_{21}H_{24}IN_3 \cdot H_2O$: C, 54.43; H, 5.66; N, 9.07. Found: 54.18; H, 5.81; N, 8.94.

^{1}H NMR (400 MHz, DMSO$-d_6$): δ[ppm] 8.02 (dd, J = 6.2, 3.2 Hz, 2H), 7.68 (dd, J = 6.2, 3.1 Hz, 2H), 7.20 (s, 2H), 3.91 (s, 6H), 3.31 (m, 4H), 2.78 (t, J = 6.2 Hz, 4H), 1.92 (quin, J = 6.0 Hz, 4H).

^{13}C{^{1}H} NMR (100 MHz, DMSO$-d_6$): δ[ppm] 151.56, 145.74, 131.74, 128.91, 126.05, 120.65, 112.90, 104.07, 49.10, 32.93, 27.06, 20.60.

1.7.10 Synthesis of derivative 8f as methyl sulfate salt

In a 2 neck, 50 mL roundbottom flask, derivative **7f** (600 mg, 1.98 mmol) is added. The system is put under N_2 atmosphere and acetone (25 mL, dried over $CaSO_4$) is added to the flask to give a suspension. The mixture is put under reflux and Me_2SO_4 (274 mg, 2.17 mmol) is added to the flask. A red solution forms. The reaction is stopped and left to cool down to room temperature. After 21 hours and a precipitate forms. The precipitate is filtered on a Hirsh funnel, washed with acetone and then with Et_2O. The obtained powder is then recrystallized in anisole. The recovered solid is then dried under reduced pressure to afford 711 mg of product (1.66 mmol, 83.9% yield). m.p. does not melt, degradation at T >150 °C.

^{1}H NMR (400 MHz, DMSO$-d_6$): δ[ppm] 8.02 (dd, J = 6.2, 3.2 Hz, 2H), 7.68 (dd, J = 6.2, 3.1 Hz, 2H), 7.21 (s, 2H), 3.92 (s, 6H), 3.56 (s, 3H), 3.31 (m, 4H), 2.78 (t, J = 6.2 Hz, 4H), 1.92 (quin, J = 6.0 Hz, 4H).

^{13}C{^{1}H} NMR (100 MHz, DMSO$-d_6$): δ[ppm] 151.56, 145.74, 131.75, 128.93, 126.05, 120.65, 112.91, 104.10, 52.73, 49.11, 32.91, 27.07, 20.61.

1.7.11 Synthesis of derivative 8h

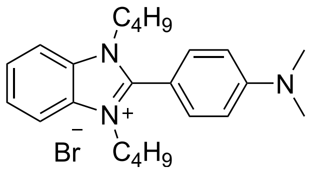

Derivative **7h** (2.000 g, 6.816 mmol) is added to a 2 neck 50 mL roundbottom flask and the system is put under N_2. 1-bromobutane (1.168 g, 8.52 mmol) is added to the flask and the mixture is heated at 90°C, obtaining a homogeneous solution. The mixture is left stirring and a precipitate forms. After 3 hours and 45 minutes, 6 mL of toluene are added to the flask. The mixture is then left stirring for 1 more hour. The reaction is then stopped and let cool down to room temperature. The obtained white precipitate is filtered on a Hirsh funnel and washed with diethyl ether. The recovered solid is dried in vacuum and then purified via crystallization in anisole (7 mL). The obtained product is filtered, washed with Et_2O and toluene and dried under reduced pressure at 65 °C until weight stabilization (2.080 g, 4.832 mmol, 70.9% yield). mp 223-225 °C. Anal. Calcd for $C_{23}H_{32}BrN_3$: C, 64.18; H, 7.49; N, 9.76. Found: 64.08; H, 7.61; N, 9.68.

^{1}H NMR (400 MHz, $CDCl_3$): δ[ppm] 7.88 (dd, J = 6.3, 3.1 Hz, 2H), 7.64 (dd, J = 6.2, 3.1 Hz, 2H), 7.60 (d, J = 8.9 Hz, 2H), 6.92 (d, J = 8.9, 2H), 4.46 (t, J = 7.6 Hz, 4H), 3.12 (s, 6H), 1.84-1.76 (m, 4H), 1.31-1.21 (m, 4H), 0.83 (t, J = 7.4 Hz, 6H).

^{13}C{^{1}H} NMR (100 MHz, $CDCl_3$): δ[ppm] 152.71, 151.43, 131.47, 131.38, 127.48, 127.21, 113.71, 112.86, 46.92, 40.50, 31.44, 19.94, 13.59.

1.7.12 Synthesis of derivative 8i

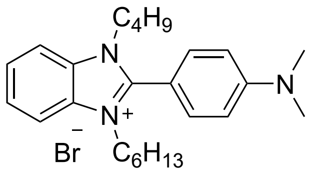

Derivative **7h** (1.200 g, 4.082 mmol) is added to a 2 neck 50 mL roundbottom flask and the system is put under N_2 atmosphere. 1-bromohexhane (742.6 g, 4.498 mmol) is added to the flask and the mixture is heated at 110 °C. After 1.5 hour, 1 mL of toluene is added to the mixture followed by 100 mg (0.606 mmol)of 1-bromohexhane. After 9 more hours, the reaction is stopped and

let cool down to room temperature. Toluene (8 mL) is then added to the flask and a white solid precipitate. The solid is filtered on a Hirsh funnel and washed with toluene and Et_2O. The deliquescent solid is recovered and refluxed in AcOEt. The obtained suspension is let cool down and the solid is filtered. The procedure is repeated once more and the recovered product is dried under reduced pressure at 65°C (1.422g, 3.101 mmol, 76.0% yield). mp 134-137 °C. Anal. Calcd for $C_{25}H_{36}BrN_3$: C, 65.49; H, 7.91; N, 9.17. Found: 65.18; H, 8.01; N, 8.99.

^{1}H NMR (400 MHz, $CDCl_3$): δ[ppm] 7.91-7.84 (m, 2H), 7.66-7.63 (m, 2H), 7.60 (d, J = 8.9 Hz, 2H), 6.97 (d, J = 8.7 Hz, 2H), 4.48-4.42 (m, 4H), 3.13 (s, 6H), 1.83-1.76 (m, 4H), 1.29-1.18 (m, 8H), 0.83 (t, J = 7.3 Hz, 3H), 0.80 (t, J = 7.0 Hz, 3H).

^{13}C{^{1}H} NMR (100 MHz, $CDCl_3$): δ[ppm] 152.63, 151.33, 131.39, 131.31, 131.29, 127.54, 127.23, 113.74, 113.60, 112.94, 47.00, 46.87, 40.53, 31.42, 31.05, 29.34, 26.27, 22.45, 19.92, 14.01, 13.59.

1.7.13 Synthesis of derivative 8l

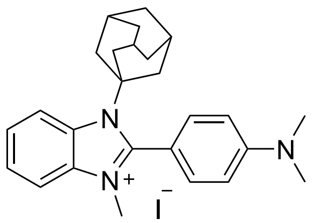

In a 2 neck, 50 mL rounbottom flask, derivative **7l** (743 mg, 2.00 mmol) is added and the system is put under N_2 atmosphere. Anhydrous toluene (6.0 mL) is added to the flask and a suspension is obtained. Methyl iodide (292 mg, 2.0 mmol) is then slowly added to the flask and the mixture is heated under reflux. A precipitate forms. After 12 hours, the reaction is cooled down to 80 °C and the solid is recovered by filtration. The crude is refluxed in toluene (20 mL), then hot filtered, and the powder is washed with 10 mL of hot toluene. The recovered solid is then dried under vacuum at 65°C until weight stabilization to afford 930 mg of product (1.81 mmol, 90.4 % yield). mp: degradation at T >208 °C. Anal. Calcd for $C_{26}H_{32}IN_3$: C, 60.82; H, 6.28; N, 8.18. Found: 60.90; H, 6.36; N, 8.03.

^{1}H NMR (400 MHz, DMSO$-d_6$): δ[ppm] 8.45 (d, J = 8.3 Hz, 1H), 8.08-8.01 (m, 1H),

7.73-7.64 (m, 2H), 7.55 (d, J = 8.9 Hz, 2H), 6.89 (d, J = 8.9 Hz, 2H), 3.54 (s, 3H), 3.04 (s, 6H), 2.30 (m, 6H), 2.13 (br, 3H), 1.75-1.72 (m, 3H), 1.65-1.62 (m, 3H).

$^{13}C\{^1H\}$ NMR (100 MHz, DMSO$-d_6$): δ[ppm] 151.79, 151.44, 132.25, 131.12, 129.90, 125.89, 125.61, 118.13, 113.69, 110.90, 110.67, 99.55, 65.69, 64.30, 41.43, 34.81, 31.99, 29.49.

1.7.14 Synthesis of derivative 8m

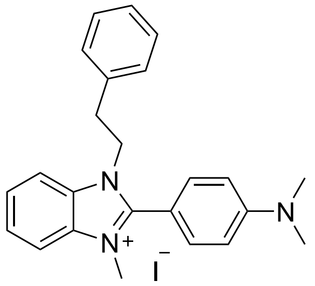

Derivative **7m** (683 mg, 2,00 mmol) is added to a two neck, 50 mL roundbottom flask and the system is put under N_2 atmosphere. Toluene (5.0 mL) is added to the flask and the system is heated at 80 °C. Methyl iodide (284 mg, 2.00 mmol) is added to the obtained solution. A yellow precipitate progressively forms. The reaction is stopped after 18.5 hours and the mixture is filtered on a hirsh funnel. The recovered solid is washed in toluene (10 mL) at 70°C and then filtered again. The recovered yellow powder is dried under vacuum at 65°C until weight stabilization (780 mg, 1.61 mmol, 80.5 % yield). m.p. : degradation at T >200 °C. Anal. Calcd for $C_{24}H_{26}IN_3$: C, 59.63; H, 5.42; N, 8.69. Found: 59.55; H, 5.50; N, 8.58.

^1H NMR (400 MHz, DMSO$-d_6$): δ[ppm] 8.10-8.05 (m, 2H), 7.70-7.67 (m, 2H), 7.45 (d, J = 8.9 Hz, 2H), 7.22-7.20 (m, 3H), 7.00-6.98 (m, 2H), 6.93 (d, J = 6.9 Hz), 4.59 (t, J = 7.3 Hz, 2H), 3.84 (s, 3H), 3.08 (s, 6H), 3.05-3.00 (m, 2H).

$^{13}C\{^1H\}$ NMR (100 MHz, DMSO$-d_6$): δ[ppm] 151.79, 151.44, 132.25, 131.12, 129.90, 125.89, 125.61, 118.13, 113.69, 110.90, 110.67, 99.55, 65.69, 64.30, 41.43, 34.81, 31.99, 29.49.

1.7.15 Synthesis of derivative 8n

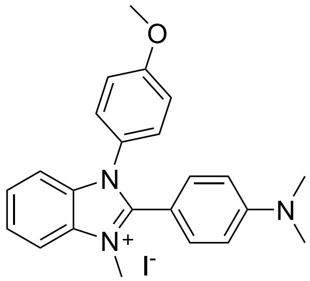

Derivative **7n** (2.000 g, 5.823 mmol) is added to a two neck, 100 mL roundbottom flask. The system is put under N_2 and ethyl acetate (50 mL) is added. The mixture is heated up under reflux and a solution forms. Methyl iodide (0.970 g, 6.834 mmol) is then added. The mixture is kept under magnetic stirring and the reaction progress is monitored via TLC using a mixture of heptane/AcOEt 7:3 as eluent. A white precipitate forms. After complete conversion, the reaction is stopped. The obtained precipitate is filtered on a Hirsh funnel and washed with 20 mL of ethyl acetate. The collected solid is dried under reduced pressure at 65 °C until weight stabilization to afford 2.081 g of product as a white powder (4.309 mmol, 74.0% yield). mp: degradation at T >100 °C. Anal. Calcd for $C_{23}H_{24}IN_3O \cdot \frac{1}{2}H_2O$: C, 55.88; H, 5.10; N, 8.50. Found: 55.71; H, 5.20; N, 8.39.

^{1}H NMR (400 MHz, DMSO-d6): δ[ppm] 8.18 (d, J=8.2 Hz, 1H), 7.75 (t, J = 7.8 HZ, 1H), 7,65 (t, J = 7.8 Hz, 1H), 7.49 (d, J = 9.0 Hz, 2H), 7.44-7.42 (m, 3H), 7.14 (d, J = 9.0 Hz, 2H), 6.78 (d, J = 9.0 Hz, 2H), 4.02 (s, 3H), 3.82 (s, 3H), 2.99 (s, 6H).

^{13}C{^{1}H} NMR (100 MHz, DMSO-d6): δ[ppm] 160.11, 152.13, 151.77, 132.78, 132.22, 131.81, 129.01, 126.82, 126.39, 125.71, 115.25, 113.33, 112.64, 111.17, 105.86, 55.61, 39.49, 33.11.

1.8 Synthesis of dopants

NMR characterization of the products reported in this section was performed by preparing the samples under argon atmosphere to avoid oxidation of the dopantss due to air exposure. In the case of acid clay catalyzed condensation reactions, we tested both microwave heating and traditional oil bath heating without noticing any difference in the reaction outcome. In both cases, Montmorillonite clay was oven dried at 130 °C before use.

1.8.1 Synthesis of derivative 5a

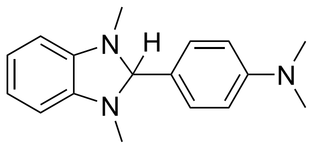

In a 50 mL two neck round bottom flask derivative **8a** (218 mg, 0.525 mmol) is added. Methanol is then added to the flask (3.5 mL) and the obtained solution is cooled down with an ice bath to 0°C. $NaBH_4$ (39.7 mg, 1.05 mmol) is then slowly added to the flask. A white precipitate forms. The reaction is stopped after 15 minutes and the methanol is evaporated under reduced pressure. The product is extracted with toluene (12 mL). The obtained solution is then recovered and filtered on a 0.2 μm syringe filter and the solvent is evaporated under reduced pressure. The obtained solid is then dried under vacuum to afford the product as a pale yellow powder (131 mg, 0.490 mmol, 93.3% yield). Residual impurities, already present in reagent **8a**, lowers the product purity to 95%.

^{1}H NMR (400 MHz, DMSO–d6): delta[ppm] 7.34 (d, J = 8.8 Hz, 2H), 6.76 (d, J=8.8 Hz, 2H), 6.60 (m, 2H), 6.41 (m, 2H), 4.72 (s, 1H), 2.93 (s, 6H), 2.44 (s, 6H).[S2]

1.8.2 Alternatively, the product can be prepared by acid catalyzed condensation:

The reaction mixture is prepared inside a glove box, under argon atmosphere. All purification steps are performed in the dark. Derivative **3a** (136 mg, 0.998 mmol), 4-(dimethylamino)benzaldehyde (153.07 mg, 1.0260 mmol) and Montmorillonite clay K10 (118 mg) are added to

a 10 mL microwave test tube. The mixture is then put under magnetic stirring and heated at 120 °C, in the dark, observing formation of a yellow mud. The reaction progress is monitored via TLC using toluene/Et_2O 9:1 as eluent. After 1 hour the reaction is stopped and let cool down to room temperature. The crude is taken up with toluene and extraction of the product from montmorillonite clay is helped using an ultrasonic bath. The obtained suspension is filtered through a 0.45 μm syringe filter and the obtained solution is evaporated under reduced pressure. The crude is dissolved in 5 mL of toluene and filtered through a silica pad using toluene/Et_2O as solvent. Fractions containing products were collected and evaporated under reduced pressure to afford the product as a pale yellow powder which is then dried under vacuum (170 mg, 0.636 mmol, 63.7% yield).

1.8.3 Synthesis of derivative 5b via reduction reaction

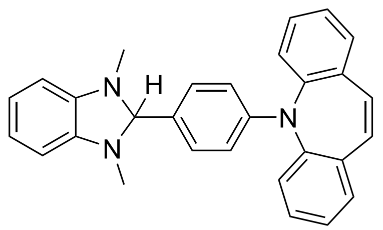

In a 50 ml round bottom flask, derivative **8b** (iodide salt, 200 mg, 0.369 mmol) is added and dissolved in 15 mL of methanol. The flask is placed at 0 °C in an ice bath and $NaBH_4$ (58 mg, 1.5 mmol) is slowly added. A white precipitate immediately forms. After 1 h, the reaction is stopped and the obtained precipitate is filtered on an Hirsh funnel and then dried under vacuum (142 mg, 0.342 mmol, 92.6% yield). The product is stored under argon atmosphere at 4 °C to avoid oxidation from atmosphere.

^{1}H NMR (400 MHz, C_6D_6): δ[ppm] 7.38 (dd, J = 7.9, 1.1 Hz, 2H), 7.23 (d, J = 8.8 Hz, 2H), 7.15 - 7.12 (m, 2H), 7.09 (m, 2H), 7.01 (m, 2H), 6.82 (dd, J = 5.4, 3.2 Hz, 2H), 6.53 (d, J = 8.8 Hz, 2H), 6.52 (s, 2H), 6.33 (dd, J = 5.4, 3.2 Hz, 2H), 4.51 (s, 1H), 2.25 (s, 6H).[S3]

^{13}C{^{1}H} NMR (100 MHz, C_6D_6): δ[ppm] : 150.30, 143.55, 142.83, 136.88, 130.89, 130.76, 130.73, 129.95, 129.75, 128.92, 127.30, 119.63, 112.23, 105.98, 94.27, 32.92.

1.8.4 Synthesis of 5b via acid catalyzed condensation

The reaction mixture is prepared inside a glove box, under argon atmosphere. All purification steps are performed in the dark. Derivative 3a (149 mg, 1.09 mmol), derivative **4b** (270 mg, 0.909 mmol) and Montmorillonite clay K10 (102 mg) are added to a 10 mL microwave test tube. The mixture is heated at 185 °C in the dark and mixed via magnetic stirring, observing formation of a yellow mud. Reaction progress is monitored by TLC using toluene/Et_2O 9:1 as eluent. After 1h the reaction is stopped and let cool down at room temperature. The crude is taken up with toluene and the extraction of the product from Montmorillonite clay is helped with the use of a ultrasonic bath. The obtained suspension is filtered through a Teflon 0.45 μm syringe filter and the recovered solvent is evaporated under reduced pressure. The obtained crude is dissolved in 5 mL of toluene and filtered on a silica pad using toluene/Et_2O 9:1 as eluent (100 mL). Fractions containing product are collected and solvent is evaporated under reduced pressure to afford a white powder. The powder is dried under vacuum to give 243 mg of product (0.585 mmol, 64.3% yield). The product is stored under argon atmosphere at 4 °C to avoid oxidation from atmosphere.

1.8.5 Alternative conditions and purification tested

From **3a** (125 mg, 0.918 mmol), derivative **4b** (271 mg, 0.911 mmol) and Montmorillonite clay K10 (119 mg). Reaction is heated at 150°C for 1 hour and then at 165 °C for further 40 minutes. Crude is taken up with dichloromethane (10 mL) and the obtained solution is filtered on a Teflon 0.45 μm syringe filter. The recovered solvent is evaporated under reduced pressure and the obtained solid is taken up with MeOH (10 mL) and filtered on a Hirsh funnel, and then washed with further 10 mL of MeOH. The procedure is repeated three times. The obtained white powder is dried under reduced pressure at 40 °C to afford 269 mg of product (0.647 mmol, 71.1% yield, 96% purity).

1.8.6 Synthesis of derivative 5c

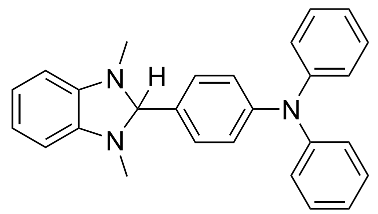

In a 50 mL roundbottom flask, derivative **8c** (1.551 g, 2.998 mmol) is added and then dissolved in methanol (12 mL). The flask is cooled down to 0 °C with an ice bath and $NaBH_4$ (228 mg, 6.03 mmol) is slowly added to the mixture. A precipitate forms. After 2 hours and 30 minutes the reaction si stopped and the precipitate is filtered on a hirsh funnel. A second solid fraction is recovered from the residual solvent via ultracentrifugation. The two solids are collected together and dried under vacuum at 65 °C to give the product as a white powder (725 mg, 1.85 mmol, 61.7% yield). The product is stored under argon atmosphere at 4 °C to avoid oxidation from atmosphere.

^{1}H NMR (400 MHz, $CDCl_3$): δ[ppm] 7.41 (d, J = 8.5 Hz, 2H), 7.28-7.24 (m, 4H), 7.13 - 7.11 (m, 4H), 7.09 (d, J = 8.5 Hz, 2H), 7.04 (m, 2H), 6.71 (dd, J = 5.4, 3.1 Hz, 2H), 6.44 (dd, J = 5.4, 3.2 Hz, 2H), 4.81 (s, 1H), 2.60 (s, 6H).[S21]

^{13}C{^{1}H} NMR (100 MHz, $CDCl_3$): δ[ppm] 148.91, 147.78, 142.29, 132.58, 129.65, 129.44, 124.79, 123.26, 123.12, 119.42, 105.94, 93.80, 33.46.

1.8.7 Alternatively, the product can be prepared by acid catalyzed condensation

The reaction mixture is prepared inside a glove box, under argon atmosphere. All purification steps are performed in the dark. Derivative **3a** (103.7 mg, 0.7614 mmol), 4-(N,N Diphenylamino)benzaldehyde (201.7 mg, 0.7379 mmol) and Montmorillonite clay K10 (20.06 mg) are added to a 10 mL microwave test tube. The mixture is then put under magnetic stirring, in the dark, and heated with at 120 °C for 20 minutes, observing formation of a yellow mud. The test tube is then opened to remove condensed water and to monitor the reaction progress via TLC using dichloromethane as eluent. The reaction is then put under N_2 and heated again at 120 °C for 20 more minutes. The same procedure is repeated once more.

The reaction is then stopped, taken up with dichloromethane to dissolve the crude and the obtained suspension is filtered on a Teflon 0.45 μm syringe filter. The obtained solution is then evaporated under reduced pressure to afford a waxy crude. The crude is taken up with heptane and the obtained suspension is filtered on a Hirsh funnel. The recovered powder is dried under vacuum to give 205 mg of product (0.524 mmol, 71.0% yield). The product is stored under argon atmosphere at 4 °C to avoid oxidation from atmosphere.

1.8.8 Synthesis of derivative 5d

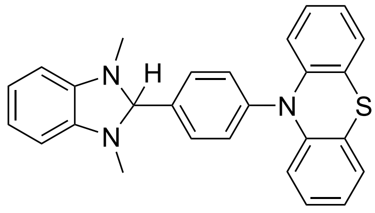

Derivative **8d** (iodide salt, 161 mg, 0.294 mmol) is added to a 20 ml vial and methanol (3 mL) is added to obtain a pale yellow suspension. The mixture is put under stirring and cooled down with an ice bath and $NaBH_4$ (66.58 mg, 1.76 mmol) is slowly added. A white suspension is obtained. The system is then put under argon to avoid oxidation of the product. The reaction is stopped after 40 minutes and the suspension is filtered on a Hirsh funnel. The obtained solid is then dried under vacuum to give the product as a white powder (119 mg, 0.282 mmol, 95.9% yield). The product is stored under argon atmosphere at 4 °C to avoid oxidation from atmosphere. Anal. Calcd for $C_{27}H_{23}N_3S$: C, 76.93; H, 5.50; N, 9.97. Found: 76.84; H, 5.55; N, 9.91.

^{1}H NMR (400 MHz, C_6D_6): δ[ppm] 7.42 (d, J = 8.3 Hz, 2H), 7.03 (d, J = 8.3 Hz, 2H), 6.98 (m, 2H), 6.87 (dd, J = 5.4, 3.1 Hz, 2H), 6.67-6.59 (m , 4H), 6.38 (dd, J = 5.4, 3.1 Hz, 2H), 6.26 (m, 2H), 4.55 (s, 1H), 2.23 (s, 1H).

^{13}C{^{1}H} NMR (100 MHz, C_6D_6): δ[ppm] 144.75, 142.48, 139.55, 131.36, 130.61, 127.32, 127.08, 123.10, 121.53, 120.08, 116.92, 106.33, 93.81, 33.31.

1.8.9 Alternatively, the product can be prepared by acid catalyzed condensation

The reaction mixture is prepared inside a glove box, under argon atmosphere. All purification steps are performed in the dark. Derivative **3a** (136 mg, 0.998 mmol), derivative **4d** (309.62 mg, 1.0040 mmol) and Montmorillonite clay K10 (155.76 mg) are added to a 10 mL microwave test tube. The mixture is then put under magnetic stirring, in the dark, and heated at 120 °C for 30 minutes, observing formation of a yellow mud. The test tube is then opened to remove condensed water and to monitor the reaction progress via TLC using toluene/Et_2O 9:1 as eluent. The reaction is then put under N_2 and heated again at 120 °C for 30 more minutes. The procedure is repeated once more, this time heating at 150 °C for 30 minutes. The reaction is then stopped, taken up with toluene (17 mL) to dissolve the crude and the obtained suspension is filtered on a Teflon 0.45 μm syringe filter. The obtained solution is evaporated under reduced pressure and the obtained crude is filtered on a silica pad using toluene/Et_2O 9:1 as eluent (155 mL). Fractions containing product are collected and the solvent is evaporated under reduced pressure to afford a pale yellow powder. The powder is dried under vacuum to give 369 mg of product (0.877 mmol, 87.7% yield). The product is stored under argon atmosphere at 4 °C to avoid oxidation from atmosphere.

1.8.10 Synthesis of derivative 5e

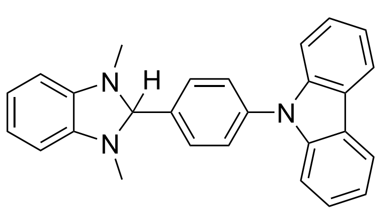

In a 50 mL roundbottom flask, derivative **8e** (triflate salt, 282 mg, 0.492 mmol) and methanol (25 mL) are added to obtain a suspension. The mixture is put at 0 °C with an ice bath and $NaBH_4$ (185 mg, 5.47 mmol) is added slowly to the suspension (86 mg are firstly added, then 99 mg are again added after 1 hour and 30 minutes). The reaction progress is monitored by TLC using toluene/Et_2O as eluent. After 2 hours and 30 minutes the reaction is stopped and the suspension is filtered on a Hirsh funnel to afford a grey powder. The powder is dried under

vacuum at 65 °C to give 179 mg of product (0.460 mmol, 93.4% yield). The product is stored under argon atmosphere at 4 °C to avoid oxidation from atmosphere.

^{1}H NMR (400 MHz, $CDCl_3$): δ[ppm] 8.16 (d, J = 7.6 Hz, 2H), 7.83 (d, J = 8.3 Hz, 2H), 7.65 (d, J = 8.3 Hz, 2H), 7.49-7.47 (m, 2H), 7.43 (m, 2H), 7.31 (m, 2H), 6.77 (dd, J = 5.4, 3.2 Hz, 2H), 6.51 (dd, J = 5.4, 3.2 Hz, 2H), 5.02 (s, 1H), 2.69 (s, 6H).[S21]

^{13}C{^{1}H} NMR (100 MHz, $CDCl_3$): δ[ppm] 142.15, 140.89, 138.82, 138.48, 130.38, 127.10, 126.11, 123.63, 120.49, 120.22, 119.67, 109.95, 106.11, 93.76, 33.61.

1.8.11 Alternatively, the product can be prepared by acid catalyzed condensation:

The reaction mixture is prepared inside a glove box, under argon atmosphere. All purification steps are performed in the dark. Derivative **3a** (86 mg, 0.63 mmol), derivative **4e** (170 mg, 0.627 mmol) and Montmorillonite clay K10 (80 mg) are added to a 10 mL test tube. The mixture is heated at 160 °C for 30 minutes. A hard gray mud forms. The test tube is opened to remove condensed water and to monitor the reaction progress via TLC using toluene/Et_2O 95:5 as eluent. The reaction is then put under N_2 and heated again at 160 °C for 20 more minutes. The reaction is taken up with dichloromethane (10 mL x 3) to extract the product from the montmorillonite clay. The obtained suspension is filtered on a 0.45 μm syringe filter to remove the residual solid and the collected solvent is evaporated under reduced pressure. The crude is taken up with methanol (10 mL), then the obtained suspension is filtered on a Hirsh funnel and the filtrated powder is washed with further 20 mL of methanol. This procedure is repeated twice. The recovered solid is then taken up with toluene (10 mL) and filtered again to afford the product as a white powder. The product is dried under reduced pressure and stored under inert atmosphere at 4 °C (132 mg, 0.339 mmol, 54.0% yield).

Note: due to the high melting point of the product (vide infra, DSC characterization), the reaction mixture turns into a very hard mud over time, which does not allow stirring.

Nonetheless, complete conversion of the reagent was obtained and it was not necessary to increase the reaction temperature.

1.8.12 Synthesis of derivative 5f

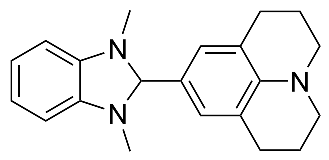

Derivative **8f** (314 mg, 0.705 mmol) is added to a a 20 ml vial, followed by methanol (7 mL). The flask is placed at 0 °Cin an ice bath and $NaBH_4$ (81.7 mg, 2.16 mmol) is slowly added. Starting reagents form a suspension. Reaction progress is monitored via TLC using Toluene:AcOet 7:3 as eluent. After 1 hour, further 25.2 mg (0.666 mmol) of $NaBH_4$ are added to the mixture. The reaction is stopped after 1 hour and 30 minutes. The pale yellow suspension is filtered on a Hirsh funnel and the recovered solid is taken up with further 3 mL of methanol. The obtained suspension is again filtered and the recovered powder is dried under reduced pressure at 50 °C to afford 146 mg of product (0.457 mmol, 64.8% yield).

^{1}H NMR (400 MHz, DMSO$-d_6$): δ[ppm] 6.85 (s, 2H), 6.57 (dd, J = 5.4, 3.2 Hz, 2H), 6.38 (dd, J = 5.4, 3.2 Hz, 2H), 4.59 (s, 1H), 3.12 (t, J = 5.6 Hz, 4H), 2.69 (t, J = 6.4, 4H), 2.43 (s, 6H), 1.88 (quin, J = 6.0 Hz, 4H).[S22]

1.8.13 Alternatively, the product can be prepared by acid catalyzed condensation:

Derivative **3a** (440 mg, 3.23 mmol), 9-julolidinecarboxaldehyde (650 mg, 3.23 mmol) and Montmorillonite K10 (400 mg) are added to a 50 mL roundbottom flask. The system is put under N_2 atmosphere and the flask is heated at 120°C. A brownish melt forms. After 30 minutes, the system is let to cool down to room temperature and the system is opened to remove condensed water on the condenser walls. The system is put again under N_2, and heated up at 120 °C. After 3 hours, the reaction is stopped and let to cool down to room temperature. The crude is taken up with 30 mL of toluene, which is then filtered on a 0.45

μm syringe filter. The solvent is then evaporated under reduced pressure to afford a pale yellow solid (1.266 g) which is crystallized from methanol (80 mL). The suspension is then left at -20 °C and the obtained solid is filtered and then dried under reduced pressure to afford 801 mg of product (2.50 mmol, 77.6% yield).

1.8.14 Synthesis of derivative 5g

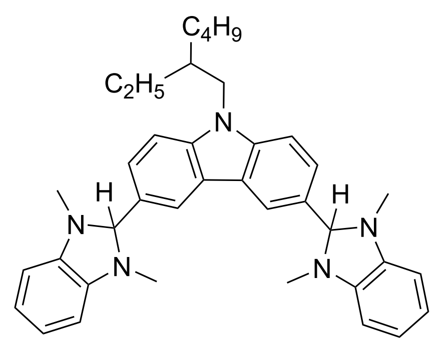

Note: Due to the high melting point of product **5g**, the reaction mixture becomes solid over time. DiMeOPEG2000 was thus added to the mixture to maintain the melt during the reaction time. The reaction mixture is prepared inside a glove box, under argon atmosphere. All purification steps are performed in the dark. Derivative **3a** (149 mg, 1.09 mmol), derivative **4g** (162.9 mg, 0.4856 mmol), Montmorillonite clay K10 (96.0 mg) and DiMeOPEG2000 (43.2 mg) are added to a 10 mL microwave test tube. The mixture is then put under magnetic stirring, in the dark, and heated at 150 °C for 1 hour, observing formation of a yellow mud. The test tube is then opened to remove condensed water and to monitor the reaction progress via TLC using toluene/Et_2O 95:5 as eluent. The reaction is then put under N_2 and heated at 180 °C for 1 hour. The same procedure is repeated once more and the temperature is increased to 210 °C. At this temperature, a softer mud is obtained, thus allowing efficient stirring. After 40 minutes, the reaction is then stopped and let cool down to room temperature. The mixture is taken up with 12 mL of toluene and the extraction of the product from the montmorillonite clay is helped using an ultrasonic bath. The obtained suspension is then filtered on a 0.45 μm syringe filter to remove the residual solid and the collected solvent is evaporated under reduced pressure. The crude is filtered on a pad of silica using toluene/Et_2O 95:5 as eluent. Fractions containing the product are collected and the solvent is evapotared under reduced pressure affording a pale yellow powder. The product is then

taken up with petroleum ether, dried under vacuum and stored under inert atmosphere at 4 °C (120 mg, 0.210 mmol, 43.7% yield). The product is stored under argon atmosphere at 4 °C to avoid oxidation from atmosphere. Anal. Calcd for $C_{38}H_{45}N_5$: C, 79.82; H, 7.93; N, 12.25. Found: 79.89; H, 7.98; N, 12.08.

^{1}H NMR (400 MHz, C_6D_6): delta[ppm] 8.33 (d, J = 1.4 Hz, 2H), 7.87 (dd, J = 8.5, 1.6 Hz, 2H), 7.31 (d, J = 8.6 Hz, 2H), 6.92 (dd, J = 5.4, 3.2 Hz, 4H), 6.46 (dd, J = 5.4, 3.2, 4H), 4.94 (s, 2H), 3.79 (m, 2H), 2.41 (s,12H), 1.91 (m, 1H), 1.18-1.04 (m, 8H), 0.79 (t, J = 6.9 Hz, 3H), 0.68 (t, J = 7.4 Hz, 3H).

^{13}C{^{1}H} NMR (100 MHz, C_6D_6): δ[ppm] 142.80, 142.53, 130.73, 127.01, 123.18, 122.01, 119.95, 109.45, 106.19, 95.19, 47.64, 39.58, 33.16, 31.18, 28.96, 24.61, 23.29, 14.18, 10.91.

1.8.15 Synthesis of derivative 5h

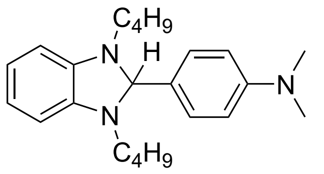

Derivative **8h** (998 mg, 2.32 mmol) is added to a a 50 ml round bottom flask, followed by methanol (8 mL). The flask is placed at 0 °Cin an ice bath and $NaBH_4$ (175.60 mg, 4.64 mmol) is slowly added. Starting reagents form a solution. After 20 minutes complete conversion is reached and the reaction is stopped. Methanol is evaporated under reduced pressure. The flask is put under N_2 atmosphere and the product is extracted with petroleum ether (15 mL + 3 × 10 mL). The obtained solution is dried on $MgSO_4$ in a second flask kept under N_2. The organic phase is then recovered and filtered on a 0.45 μm syringe filter to remove residual $MgSO_4$ and the solvent is evaporated under reduced pressure. The obtained liquid is dried under vacuum to afford the product as a colourless oil (622 mg, 1.77 mmol, 76.2% yield). Anal. Calcd for $C_{23}H_{33}N_3$: C, 78.58; H, 9.46; N, 11.95. Found: 78.36; H, 9.55; N, 11.79.

^{1}H NMR (400 MHz, DMSO−d_6): δ[ppm] 7.31 (d, J = 8.8 Hz, 2H), 6.73 (d, J = 8.8 Hz, 2H), 6.47 (dd, J = 5.4, 3.2 Hz, 2H), 6.26 (dd, J = 5.4, 3.2 Hz, 2H), 5.41 (s, 1H), 2.92 (s, 6H), 2.94-2.86 (m, 2H), 2.81-2.74 (m, 2H), 1.31-1.25 (m,4H), 1.18-1.11 (m, 4H), 0.76 (t, J =

7.3 Hz, 6H).

$^{13}C\{^{1}H\}$ NMR (100 MHz, DMSO−d_6): δ[ppm] 150.89, 140.86, 129.12, 126.46, 117.70, 111.74, 103.81, 87.91, 44.96, 40.02, 28.10, 19.69, 13.67.

1.8.16 Alternatively, the product can be prepared by acid catalyzed condensation:

The reaction mixture is prepared inside a glove box, under argon atmosphere. All purification steps are performed in the dark. Derivative **3h** (225 mg, 1.02 mmol), 4-(dimethylamino)benzaldehyde (150.57 mg, 1.0092 mmol) and Montmorillonite clay K10 (226.40 mg) are added to a 10 mL microwave test tube. The mixture is then put under magnetic stirring and heated at 120 °C for 30 minutes, in the dark, observing formation of a yellow mud. The test tube is then opened to remove condensed water and to monitor the reaction progress via TLC using toluene/Et_2O 95:5 as eluent. The reaction is then put under N_2 and heated again at 120 °C for 30 more minutes. The same procedure is repeated two more times for an overall reaction time of 2h. After 2 hours, the reaction is taken up with 12 mL of toluene to extract the product from the montmorillonite clay. The obtained suspension is then filtered on a 0.45 μm syringe filter to remove the residual solid and the collected solvent is evaporated under reduced pressure. The crude is filtered on a pad of silica using toluene/Et_2O 95:5 as eluent. Fractions containing the product are collected and the solvent is evapotared under reduced pressure affording the product as an oil. The product is then dried under vacuum and stored under inert atmosphere at 4 °C (190 mg, 0.587 mmol, 58.2% yield). Presence of oxidation impurities and residual amine reduce the purity of the product to ∼83%.

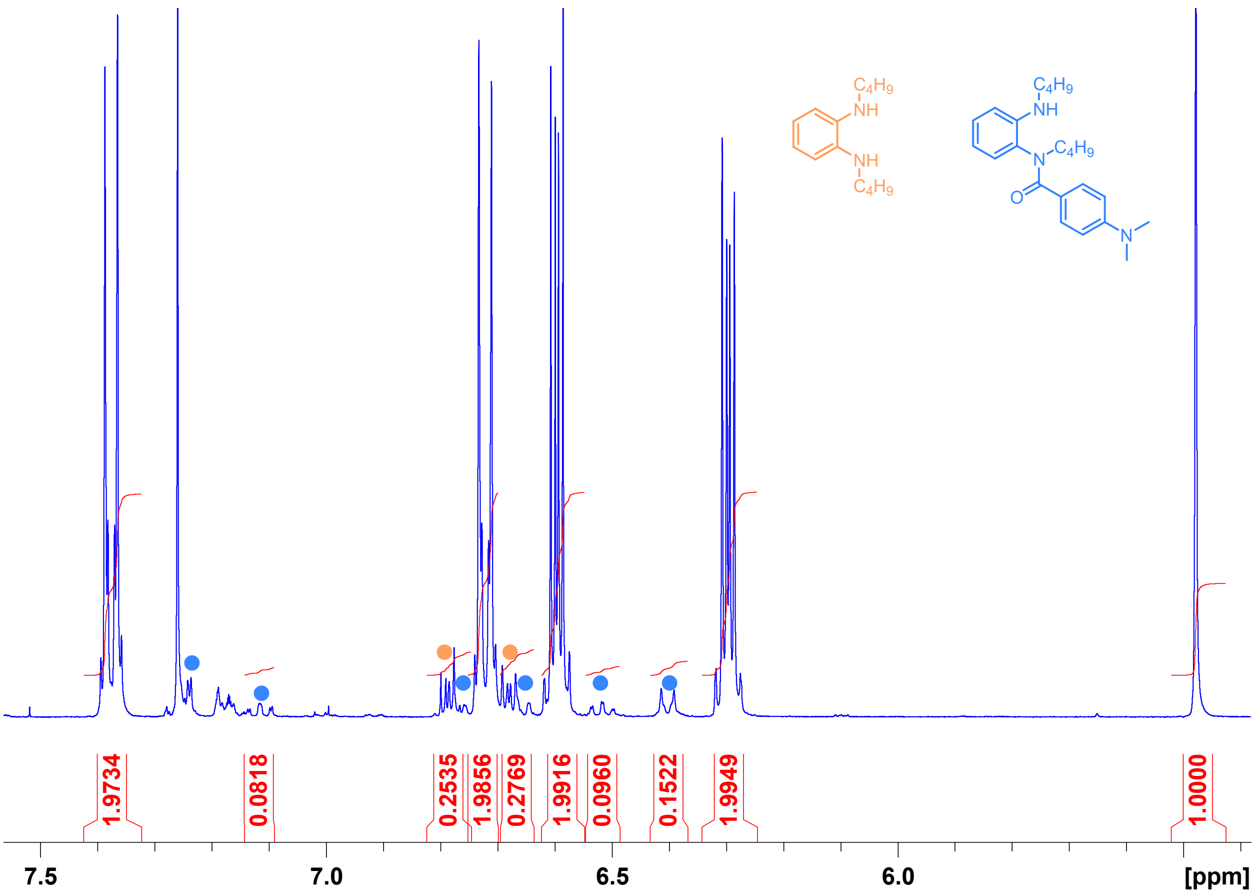

Figure S1: Details of the aromatic portion of the ^{1}H NMR in $CDCl_3$ of derivative **5h** as synthesized via condensation reaction. Peaks associated to residual amine **3h** and an unknown impurity are highlighted with orange and blue dots respectively. Peaks related to the unknown impurity are tentatively associated to the indicated oxidation byproduct on the base of previous studies on N-DMBI-H (**5a**) oxidation reactions.[S1]

1.8.17 Synthesis of derivative 5i

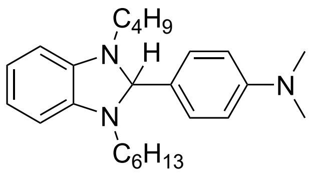

Derivative **8i** (460 mg, 1.00 mmol) is added to a 50 mL round bottom flask, followed by methanol (4 mL). The flask is placed at 0 °C in an ice bath and and $NaBH_4$ (76 mg, 2.0 mmol) is slowly added. The reagents form a solution. After 20 minutes the reagent conversion is complete. Methanol is evaporated under reduced pressure and the flask is put under N_2 atmosphere. The product is then extracted with petroleum ether (3 × 15 mL). The obtained solution is dried on $MgSO_4$

in a second flask kept under N_2. The organic phase is then recovered and filtered on a 0.45 μm syringe filter to remove residual $MgSO_4$ and the solvent is evaporated under reduced pressure. The obtained liquid is dried under vacuum to afford the product as a colourless oil (262 mg,0.664 mmol, 66.2% yield). Anal. Calcd for $C_{25}H_{37}N_3$: C, 79.10; H, 9.83; N, 11.07. Found: 78.85; H, 9.94; N, 11.14.

^{1}H NMR (400 MHz, $CDCl_3$): δ[ppm] 7.37 (d, J = 8.7 Hz, 2H), 6.72 (d, J = 8.6 Hz, 2H), 6.59 (dd, J = 5.4, 3.2, 2H), 6.31-6.28 (m, 2H), 5.47 (s, 1H), 2.98 (s, 6H), 2.93-2.87 (m, 4H), 1.42-1.34 (m, 4H), 1.26-1.14 (m, 8H), 0.82 (t, J = 6.8 Hz, 3H), 0.81 (t, J = 7.3 Hz, 3H).

$^{13}C\{^1H\}$ NMR (100 MHz, $CDCl_3$): δ[ppm] 151.29, 141.41, 141.39, 129.76, 127.61, 117.91, 112.08, 103.87, 103.85, 88.99, 46.03, 45.70, 40.68, 31.68, 28.92, 26.93, 26.75, 22.74, 20.49, 14.17, 14.00.

1.8.18 Synthesis of derivative 5l

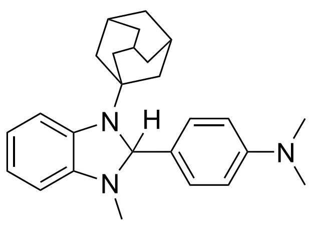

In a 10 mL round bottom flask, derivative **8l** (257 mg, 0.500) is added, followed by methanol (2.0 mL). The reagent is not completely dissolved and a suspension forms. $NaBH_4$ (55 mg, 1.5 mmol) is then slowly added to the mixture. After 1.5 hours the reaction is stopped. The mixture is filtered on a Hirsh funnel. The recovered solid is washed with 10 mL of methanol and then dried under vacuum at 65 °C to afford the product (140 mg, 0.361 mmol, 72% yield). Anal. Calcd for $C_{26}H_{33}N_3$: C, 80.58; H, 8.58; N, 10.84. Found: 80.69; H, 8.63; N, 10.60.

^{1}H NMR (400 MHz, $CDCl_3$): δ[ppm] 7.22 (d, J = 8.7 Hz, 2H), 6.74 (dd, J = 7.5, 1.0 Hz, 1H), 6.66 (d, J = 8.8 Hz, 2H), 6.59 (m, 1H), 6.52 (m, 1H), 6.17 (dd, J = 7.3, 1.3 Hz, 1H), 5.75 (s, 1H), 2.95 (s, 6H), 2.46 (s, 3H), 2.03-1.96 (m, 9H), 1.61 (m, 6H).

$^{13}C\{^1H\}$ NMR (100 MHz,$CDCl_3$): δ [ppm] 150.51, 143.32, 139.19, 131.29, 127.91, 118.18, 117.00, 112.19, 109.82, 103.73, 84.85, 55.94, 40.69, 40.52, 36.63, 31.61, 29.88.

1.8.19 Synthesis of derivative 5m

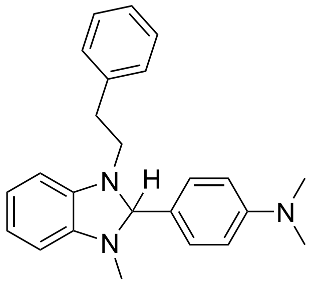

In a 10 mL roundbottom flask, derivative **8m** is added (485 mg, 1.00 mmol), followed by methanol (3.0 mL). The system is cooled down to 6 °C with an ice bath, then $NaBH_4$ (115 mg, 3.04 mmol) is slowly added to the flask. After 5 minutes, a yellow wax forms. The crude is decanted and separated from the solvent and then taken up with further 1.5 mL of methanol. The obtained mixture is filtered on a Hirsh funnel and the recovered solid is dried under vacuum at 65 °Cto afford the product as a white powder (215 mg, 0.601 mmol, 60.1% yield). Anal. Calcd for $C_{24}H_{27}N_3$: C, 80.63; H, 7.61; N, 11.75. Found: 80.46; H, 7.75; N, 11.62.

^{1}H NMR (400 MHz, DMSO$-$d$_6$): δ[ppm] 7.34 (d, J = 8.5 Hz, 2H), 7.26-7.22 (m, 2H), 7.18-7.14 (m, 1H), 7.11 (d, J = 7.2 Hz, 2H), 6.75 (d, J = 8.6 Hz, 2H), 6.56-6.54 (m, 2H), 6.39-6.37 (m, 2H), 5.16 (s, 1H), 3.24-3.16 (m, 1H), 3.00-2.96 (m, 1H), 2.93 (s, 6H), 2.71-2.64 (m, 1H), 2.58-2.53 (m, 1H), 2.44 (s, 3H).

^{13}C{^{1}H} NMR (100 MHz,DMSO$-$d$_6$) δ[ppm] 151.02, 141.79, 140.37, 139.58, 129.28, 128.66, 128.32, 125.99, 125.58, 118.71, 118.08, 111.88, 105.09, 104.62, 90.14, 47.08, 40.03, 32.64, 31.69.

1.8.20 Alternatively, the product can be prepared by acid catalyzed condensation:

The reaction mixture is prepared inside a glove box, under argon atmosphere. All purification steps are performed in the dark. Derivative **3m** (110 mg, 0.486 mmol), 4-(dimethylamino)-benzaldehyde (77.15 mg, 0.5171 mmol) and Montmorillonite clay K10 (110 mg) are added to a 10 mL microwave test tube. The mixture is then put under magnetic stirring and heated at 120 °C for 30 minutes, in the dark, observing formation of a yellow mud. The test tube is then let cool to room temperature and is opened to remove condensed water and to monitor

the reaction progress via TLC using toluene/Et_2O 9:1 as eluent. The reaction is then put under N_2 and heated again at 120 °C for 10 more minutes. The reaction is then stopped and let cool to room temperature and the crude is taken up with DCM (5 mL). The obtained suspension is filtered on a 0.45 μm syringe filter and the solvent is evaporated under reduced pressure. The obtained brown oil is taken up with methanol (4 mL) and sonicated to afford a white precipitate, that is filtered on a Hirsh funnel. The residual methanol is evaporated under reduced pressure and the procedure is repeated once more using 1.5 mL of methanol. The obtained solid is dried under vacuum at 45 °C to afford the product as a white powder (57 mg, 0.16 mmol mmol, 33% yield)

1.8.21 Synthesis of derivative 5n

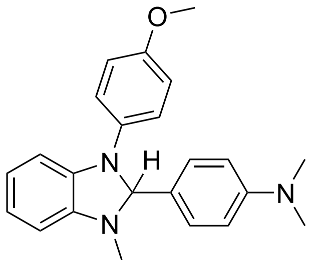

Derivative **8n** (1.340 g, 2.761 mmol) is added to a 100 mL round bottom flask and is then dissolved in 11 mL of methanol. The reaction is put at 0 °C using an ice bath and $NaBH_4$ (424 mg, 11.2 mmol) is slowly added to the solution. A brownish solid immediately precipitates. After 1h the reaction is stopped and the precipitate is filtered on a Hirsh funnel. The recovered powder is then dried in vacuum at 65 °C to afford 985 mg of product as a brownish powder (2.74 mmol, 99% yield). The product is stored under argon atmosphere at 4 °C to avoid oxidation from atmosphere. Anal. Calcd for $C_{23}H_{25}N_3O$: C, 76.85; H, 7.01; N, 11.69. Found: 76.58; H, 7.13; N, 11.51.

^{1}H NMR (400 MHz, DMSO$-d_6$): δ[ppm] 7.31 (d, J = 8.7 Hz, 2H), 7.07 (d, J = 9.0 Hz, 2H), 6.84 (d, J = 9.0 Hz, 2H), 6.66 (d, J = 8.7 Hz, 2H), 6.62 (m, 1H), 6.52 (m, 1H), 6.46 (m, 1H), 6.42 (m, 1H), 5.79 (s, 1H), 3.68 (s, 3H), 2.87 (s, 6H), 2.52 (s, 3H).

^{13}C{^{1}H} NMR (100 MHz, DMSO$-d_6$): δ[ppm] 155.49, 150.69, 141.73, 138.94, 134.76, 129.23, 125.46, 123.86, 119.16, 118.24, 114.32, 111.70, 105.38, 104.65, 89.36, 55.09, 32.34.

2 Thermal characterization - DSC

Due to their air sensitivity, we evaluated melting point of solid dopants via Differential Scanning Calorimetry analysis. Measurements were performed with a DSC 1 STAR^e system from Mettler Toledo, using aluminium crucibles. Calibration was performed with an Indium standard. All DSC measurements were conducted under N_2 flow (80 mL/min) and with a rate of 10 °C/min. DSC crucibles were prepared and closed inside a glovebox, under argon atmosphere (O_2 <0.1 ppm, H_2O level <0.1 ppm) and then punctured just before performing the analysis. For the evaluation of melting ranges, only endothermic events appearing in the 1st heating cycles were taken into consideration.

2.0.1 DSC of derivative 5b

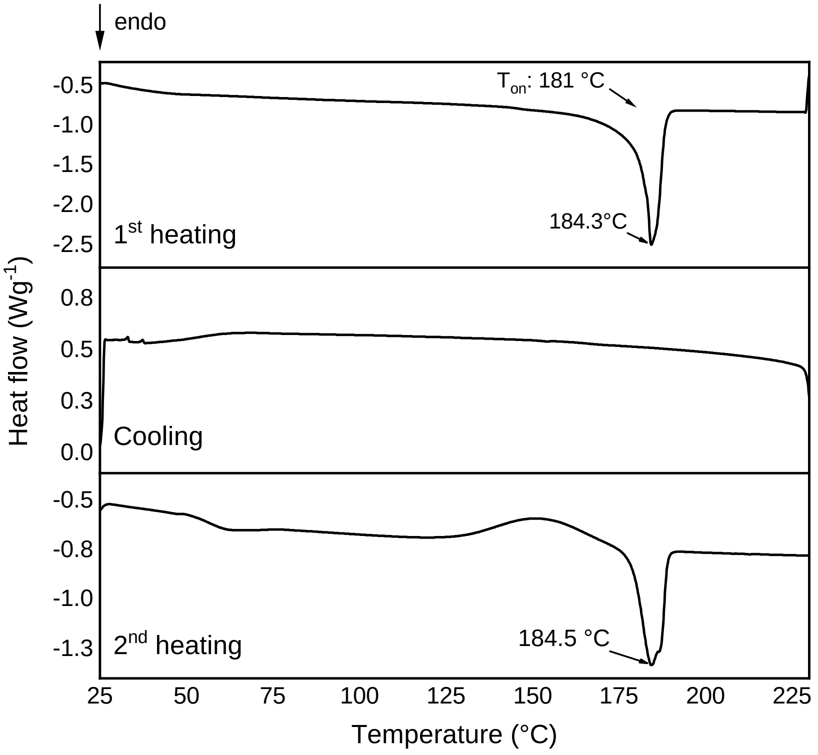

Method:

- heating from 25 °C to 230 °C
- isotherm at 230 °C for 2 minutes

• cooling from 230 °C to 25 °C

• isotherm at 25 °C for 2 minutes

• heating from 25 °C to 230 °C

Sample weight: 6.87 mg

2.0.2 DSC of derivative 5c

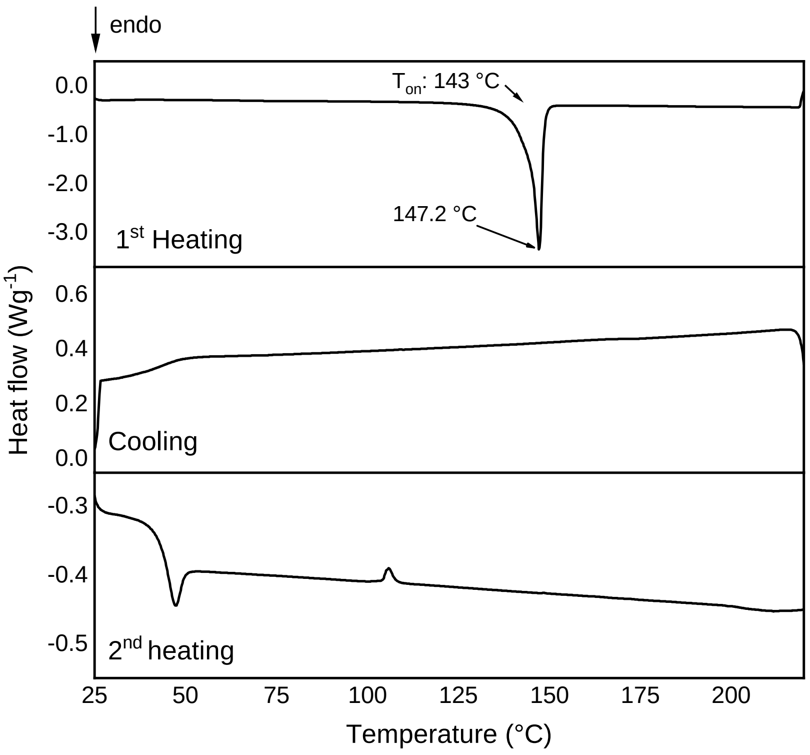

Method:

• heating from 25 °C to 220 °C

• isotherm at 220 °C for 2 minutes

• cooling from 220 °C to 25 °C

• isotherm at 25 °C for 2 minutes

• heating from 25 °C to 220 °C

Sample weight: 7.88 mg

2.0.3 DSC of derivative 5d

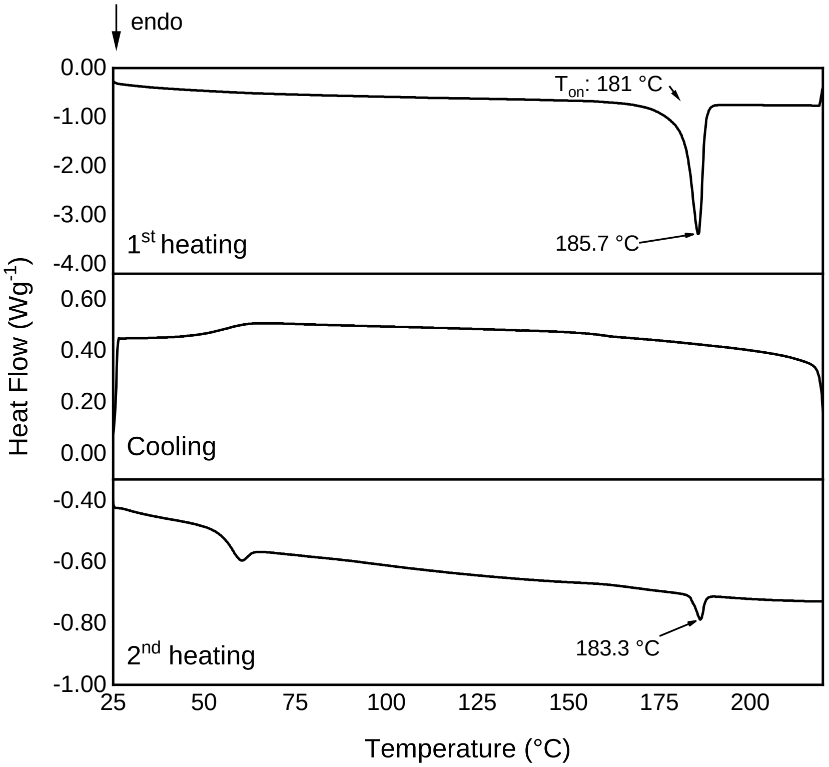

Method:

- heating from 25 °C to 220 °C
- isotherm at 220 °C for 2 minutes
- cooling from 220 °C to 25 °C
- isotherm at 25 °C for 2 minutes
- heating from 25 °C to 220 °C

Sample weight: 8.50 mg

2.0.4 DSC of derivative 5e

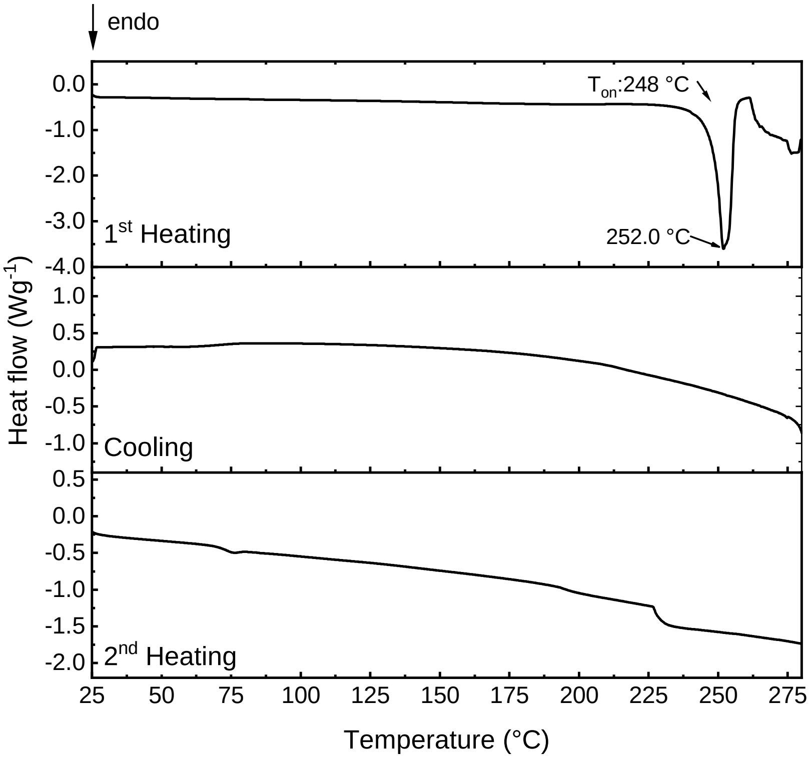

Method:

- heating from 25 °C to 280 °C
- isotherm at 280 °C for 2 minutes
- cooling from 280 °C to 25 °C
- isotherm at 25 °C for 2 minutes
- heating from 25 °C to 280 °C

Sample weight: 8.05 mg

2.0.5 DSC of derivative 5f

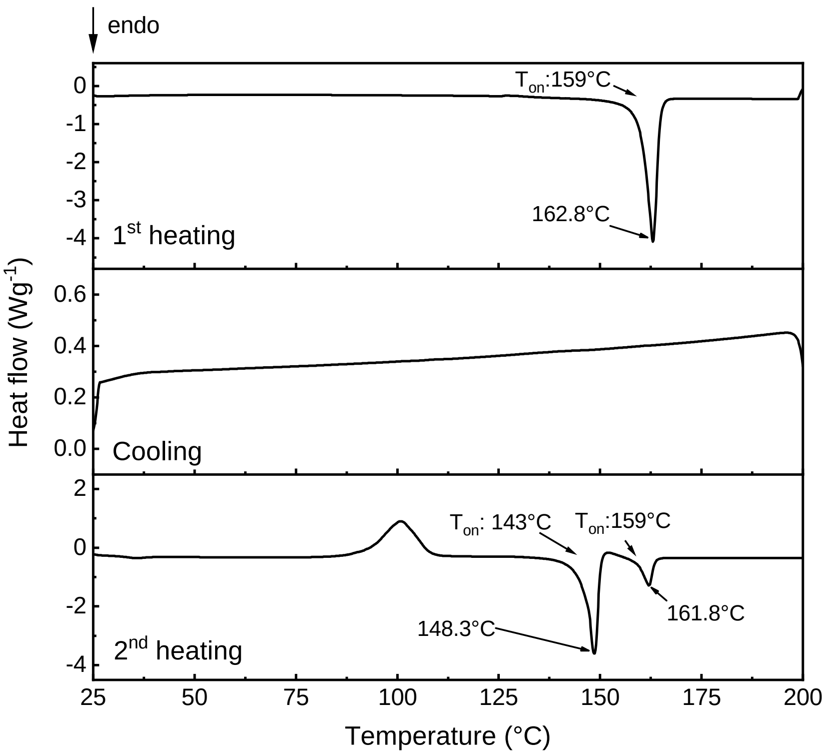

Method:

- heating from 25 °C to 200 °C
- isotherm at 200 °C for 2 minutes
- cooling from 200 °C to 25 °C
- isotherm at 25 °C for 2 minutes
- heating from 25 °C to 200 °C

Sample weight: 6.10 mg

2.0.6 DSC of derivative 5g

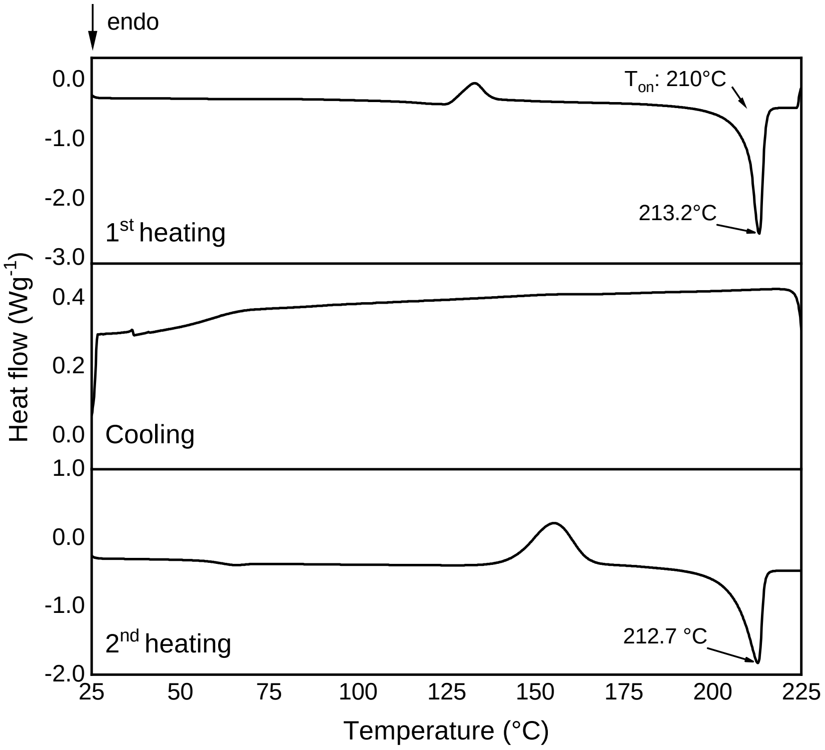

Method:

- heating from 25 °C to 225 °C
- isotherm at 225 °C for 2 minutes
- cooling from 225 °C to 25 °C
- isotherm at 25 °C for 2 minutes
- heating from 25 °C to 225 °C

Sample weight: 6.74 mg

2.0.7 DSC of derivative 5l

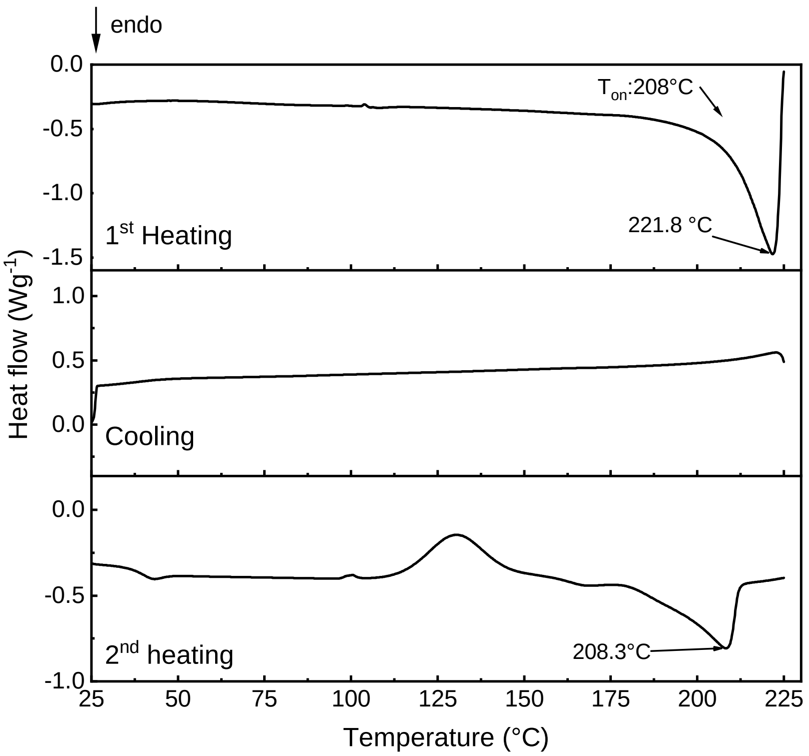

Method:

- heating from 25 °C to 225 °C
- isotherm at 225 °C for 2 minutes
- cooling from 225 °C to 25 °C
- isotherm at 25 °C for 2 minutes
- heating from 25 °C to 225 °C

Sample weight: 5.21 mg

2.0.8 DSC of derivative 5m

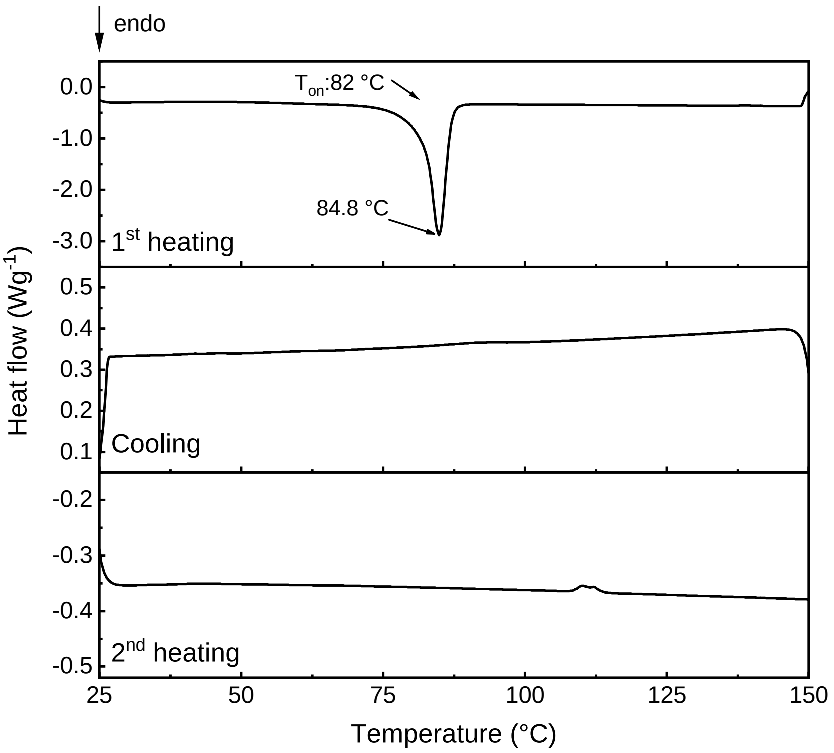

Method:

- heating from 25 °C to 150 °C
- isotherm at 150 °C for 2 minutes
- cooling from 150 °C to 25 °C
- isotherm at 25 °C for 2 minutes
- heating from 25 °C to 150 °C

Sample weight: 9.46 mg

2.0.9 DSC of derivative 5n

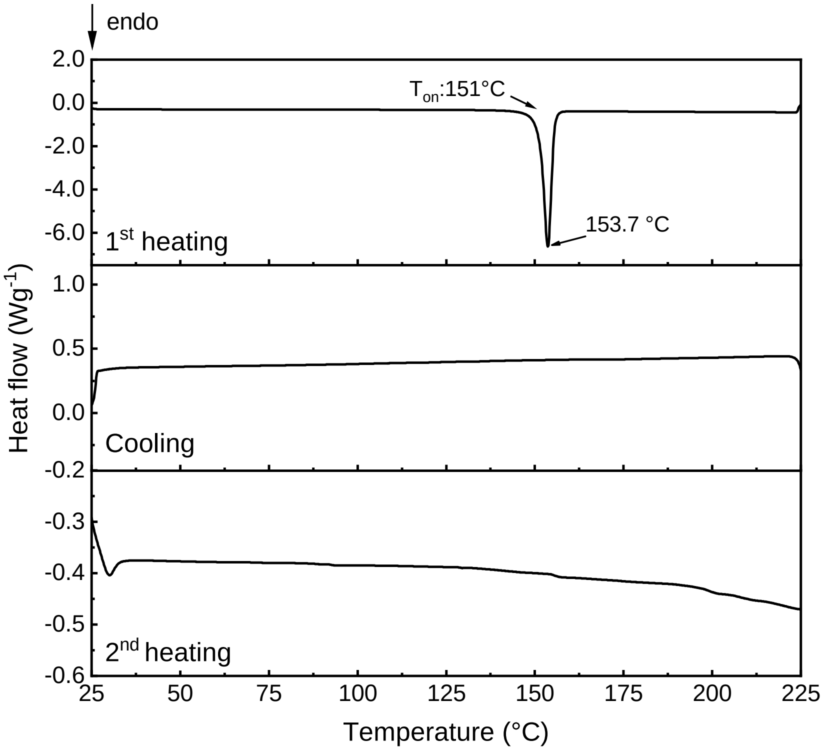

Method:

- heating from 25 °C to 225 °C
- isotherm at 225 °C for 2 minutes
- cooling from 225 °C to 25 °C
- isotherm at 25 °C for 2 minutes
- heating from 25 °C to 225 °C

Sample weight: 6.29 mg

3 Thermal characterization - TGA

We evaluated the thermal stability of all the derivatives via thermogravimetric analysis (TGA). Measurements were performed with a TGA/DSC1 STAR^e system from Mettler Toledo, using alumina crucibles. All measurements were conducted under N_2 flow (50 mL/min) and with a rate of 10 °C/min. Thermal ramps from 30 to 600 °C were generally used, with the exception of derivative **5g** and **5n**, analysed with a heating treatment going from 30 to 550 °C. Due to their very poor stability in air, crucibles containing compounds **5h** and **5i** were prepared inside a glovebox, under argon atmosphere (O_2 <0.1 ppm, H_2O level <0.1 ppm) and then exposed to air just before performing the analysis.

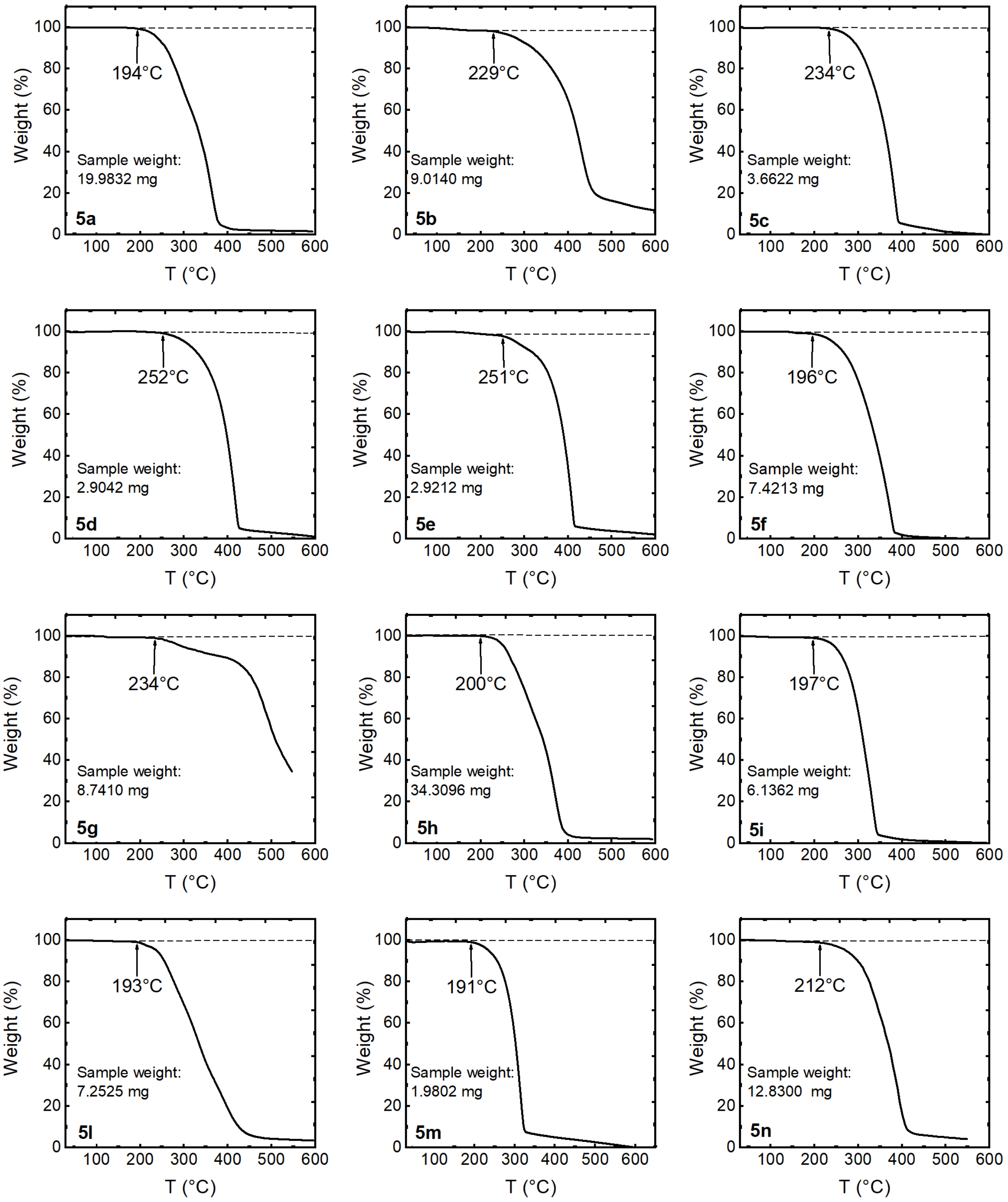

Figure S2: Results of TGA characterization of derivatives **5a-5n**. Temperature corresponding to the onset of weight loss is indicated on each graph, together with sample weight.

4 Dopants solubility evaluation

Solubility in toluene of the obtained derivatives was evaluated according to the following procedure. For each product a suspension was obtained in 500 μL of toluene. The suspension was then filtered on cotton in a 2 mL vial to remove the residual undissolved product, and the recovered saturated solution was weighted. Toluene was then allowed to evaporate under laboratory atmosphere for two days and the as recovered powder was dried under reduced pressure at 45 °C until weight stabilization. The recovered powder was weighted and the solubility in toluene was evaluated by subtracting the powder weight from the weight of the recovered saturated solution, and converting the obtained toluene weight to a volume amount (toluene density: 867 mg/mL). Solubility was not evaluated for products **5h** and **5i**, since they are liquid at room temperature.

Since DMBI-like derivatives are known to undergo oxidation when exposed to air, especially in solution, the measured solubility is likely an overestimated value, due to the weight gain associated to dopant molecules oxidation (see the below scheme for oxidized product structures). As such, the reported value is to be considered at least affected by a 5% error, associated to this weight gain, which should be added to the experimental error.

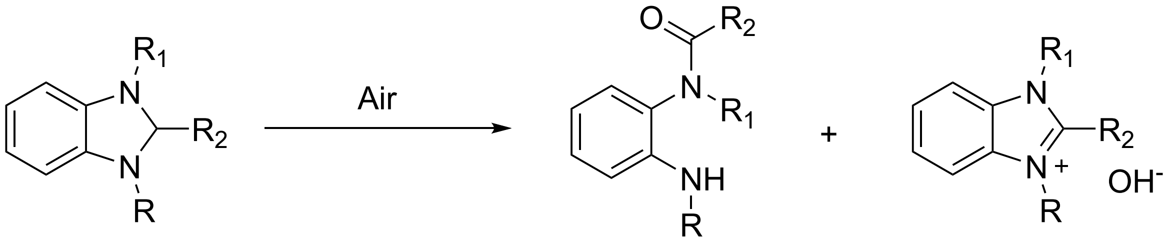

Figure S3: Plausible oxidation products of DMBI-like derivatives, according to previous reports on oxidation by-products of **5a** and **5b**.[S1–S3]

5 Electrochemical characterization

Electrochemical characterization of the synthesized dopants and of the corresponding benzimidazolium salts was performed inside an argon filled glove box (O_2 <0.1 ppm, H_2O <0.1 ppm) using a three electrode system. An AMEL glassy carbon pin electrode (3 mm diameter) mirror polished with deagglomerated alumina paste (0.3 μm, purchased from Buelher) and milliq H_2O was used as the working electrode, while a platinum wire was used as the counter electrode and an Ag / AgCl wire as the quasi reference electrode (an Ag reference electrode was used for the characterization of derivatives **5f-m**). The obtained potentials were then reffered to the Fc/Fc^+ redox couple. Anhydrous acetonitrile (99.8% Alfa Aesar) containing between 2.0 mM and 5.00 mM of substrate (depending on solubility) and 0.1 M of tetrabutylammonium perchlorate (99% Thermo Scientific) as a supporting salt was used as electrolyte.

Cyclic voltammetry (CV) analysis was performed at 50 mV/s, while Differential Pulse Voltammetry (DPV) was performed using either steps of 5mV/s and modulation amplitude of 50 mV or step of 2mV/s and modulation amplitude of 25 mV. Since the systems showed high resistance, an Ohmic drop compensation between 160-200 Ω was applied. Oxidation and reduction potentials were evaluated from DPV analysis using the following formula[S20]

$$E_0 = E_p \pm \frac{\Delta E}{2}$$

were ΔE is the modulation amplitude (ΔE >0 for oxidation processes and ΔE <0 for reduction processes).

Oxidation and reduction potentials were extrapolated from Cyclic Voltammetry curves as mean value between reduction and oxidation peaks, if present, otherwise the peak onset was considered a good estimation.

Once the oxidation and reduction potentials were measured, IE and EA were evaluated with respect to Fc/Fc^+ couple using the formula

$$E_{vac} = -e(E_0 + 4.8V)$$

in agreement to previous literature on the characterization of this class of compounds.[S20] Such values were then used as an estimate of the HOMO and LUMO energies. It is worth mentioning that the oxidation potential of Fc/Fc^+ couple in the selected electrolyte (acetonitrile) is reported to be +0.69 vs SHE.[S23] We thus here report also HOMO and SOMO energy levels obtained taking into account this value and evaluated according to the formula:

$$E_{vac} = -e(E_0 + 5.2V)$$

Reduction potentials of the corresponding 2-arylbenzimidazolium salts were measured to evaluate the dopants SOMO energies. For this purpose, triflate, methylsulfate or hexafluorophosphate 2-arylbenzimidazolium salts were used, since these salts are stable in the thermodynamic potential window of the selected electrolyte. If not already obtained via methylation of the corresponding 2-arylbenzimidazole with MeOTf, triflate salts were obtained via oxidation of the corresponding dopant with AgOTf. Hexafluorophosphate salts were instead obtained via ion exchange reactions from the corresponding iodide or bromide salts. The two procedures are briefly described in the following paragraphs.

Preparation of hexafluorophosphate salts via ion exchange The required derivative $\mathbf{8}_{-}$ (50 mg) is added to a 4 mL vial and 3 g of a NH_4PF_6 solution (33% wt. in water) are added. The obtained dispersion is stirred for 24 hours at room temperature. The solid is filtered and the procedure is repeated. The obtained solid is dried in vacuum at 65°C until weight stabilization.

The effectiveness of the ion exchange procedure was checked both by staining of the final aqueous solution with Oxone™ (running a third ion exchange with NH_4PF_6 never resulted in the development of a brown coloration of the filtered aqueous solution), and by comparing peaks integration in the ^{1}H NMR and ^{19}F NMR of the isolated salt, adding trifluoroethanol

as internal reference in the tube.

Preparation of triflate salts via oxidation with AgOTf The required derivative **5_** (1.00 mmol) is added to a test tube. AgOTf (1.10 mmol) is dissolved in methanol (2 mL) and the obtained solution is added to the test tube. A dark grey powder forms immediately. The mixture is kept stirring in the dark and the reaction progress is monitored via TLC using a mixture of toluene/Et_2O 9:1 as eluent. Tipically, after 2 hours the mixture is filtered and the recovered solution is evaporated under reduced pressure. The solid is dissolved in methanol and filtered on a celite pad. The recovered solution is then evaporated under reduced pressure to afford the desired product, which is then dried under vacuum at 65 °C until weight stabilization.

The following sections collect the obtained CV and DPV plots for each dopant. Oxidation (E_{ox}) and reduction (E_{red}) potentials reported vs Ferrocene/Ferrocenium and evaluated from both DPV and CV measurements are reported as well, together with the corresponding HOMO and SOMO energy values. The plots are similar for all the compounds. The first oxidation event, relevant for the evaluation of the HOMO level, is non reversible for all the neutral dopants. Several oxidation events are then visible at more positive potentials. Derivatives functionalized at the imdazolinic nitrogen atoms are characterized in particular by two, only partially reversible oxidation peaks, located around 0 V and 0.5 V respectively. These peaks, that are not present in dopants characterized by heteroaromatic substituents on the 2-phenyl ring, can be associated to oxidation events involving the N-dimethyl, in line to previous electrochemical characterizations of benzimidazoline derivatives.[S20] For the neutral dopants analysed in a potential window between -2.5 V and +1.25 V (vs Fc/Fc^+), a not very intense non-reversible reduction peak is then observed when going to more negative potentials after the occurrence of the oxidation events. This peak is located at the same potentials observed for the reduction of the corresponding benzimidazolium salts. This suggests formation of the dopants' cations following the non reversible oxidation process. The reduction events observed in the CV and DPV plots of the analysed benzimidazolium

salts are all non-reversible as well. Since these events are associated to formation of the dopant corresponding radicals, the poor reversibility of these reactions can be correlated to the low stability of such species. A series of oxidation peaks can then be observed in all the plots related to these salts, going to more positive potentials. All these oxidation events are non reversible, except for the oxidation peak located at +0.68 V Fc/Fc^{+} in the CV plot of **8c** and the second oxidation peak located around +0.5 vs Fc/Fc^{+} in the CV plot of **8f**, that appear to be reversible. These oxidation events might be in part be associated to oxidation of species forming after the salts' reduction, since we performed the measurements scanning toward negative potentials first. In the case of derivatives **8d** and **8n** the effects is highlighted in the reported CV plots, collecting the result of a measurement performed by scanning toward oxidation potentials as a comparison.

5.0.1 Derivative 5a

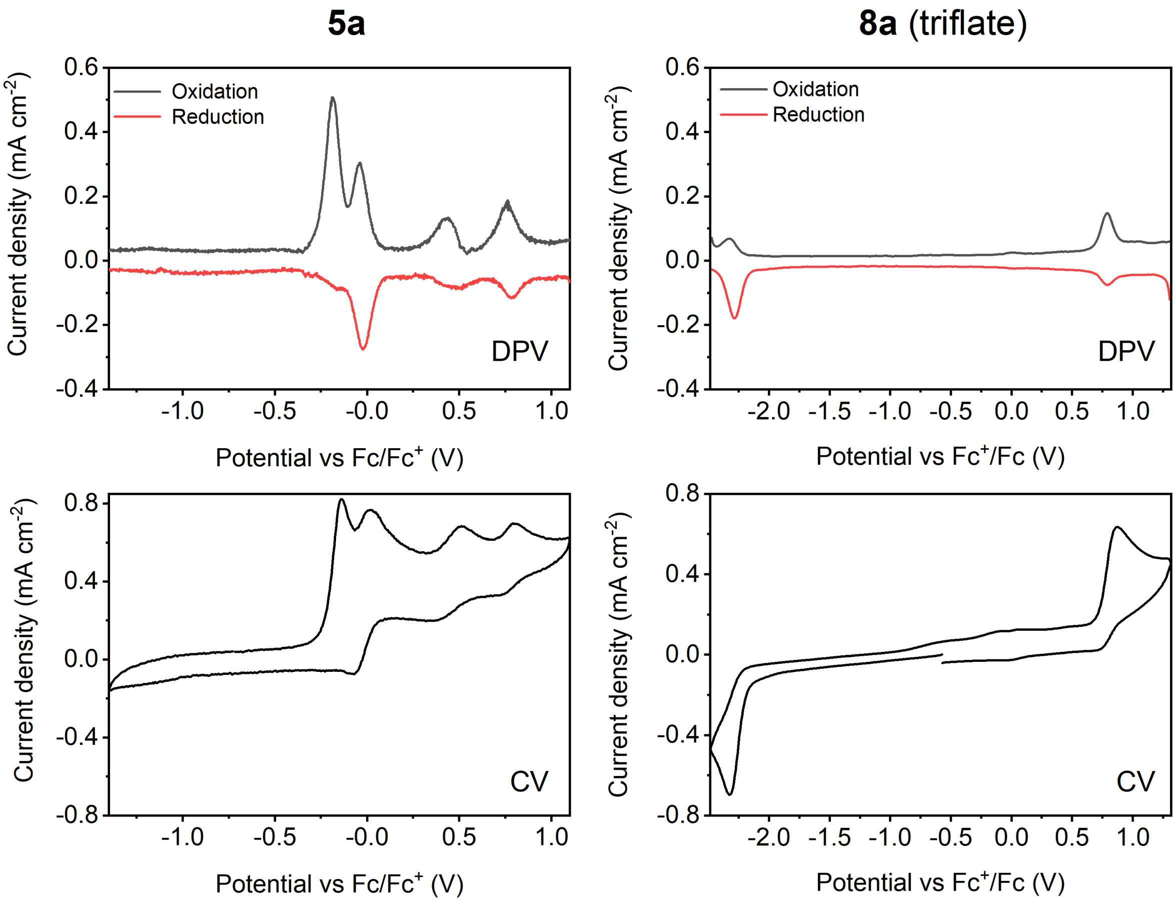

	E_{Ox} (V)	HOMO eV)[a]	E_{Red} (V)	SOMO(eV)[a]
DPV	-0,22	-4.6 (-5.0)	-2,28	-2.5 (-2.9)
CV	-0,19	-4.6 (-5.0)	-2,26	-2.5 (-2.9)

[a]Values in brackets are evaluated using the formula: E_{vac}=-e(E_0 + 5.2 V)

5.0.2 Derivative 5b

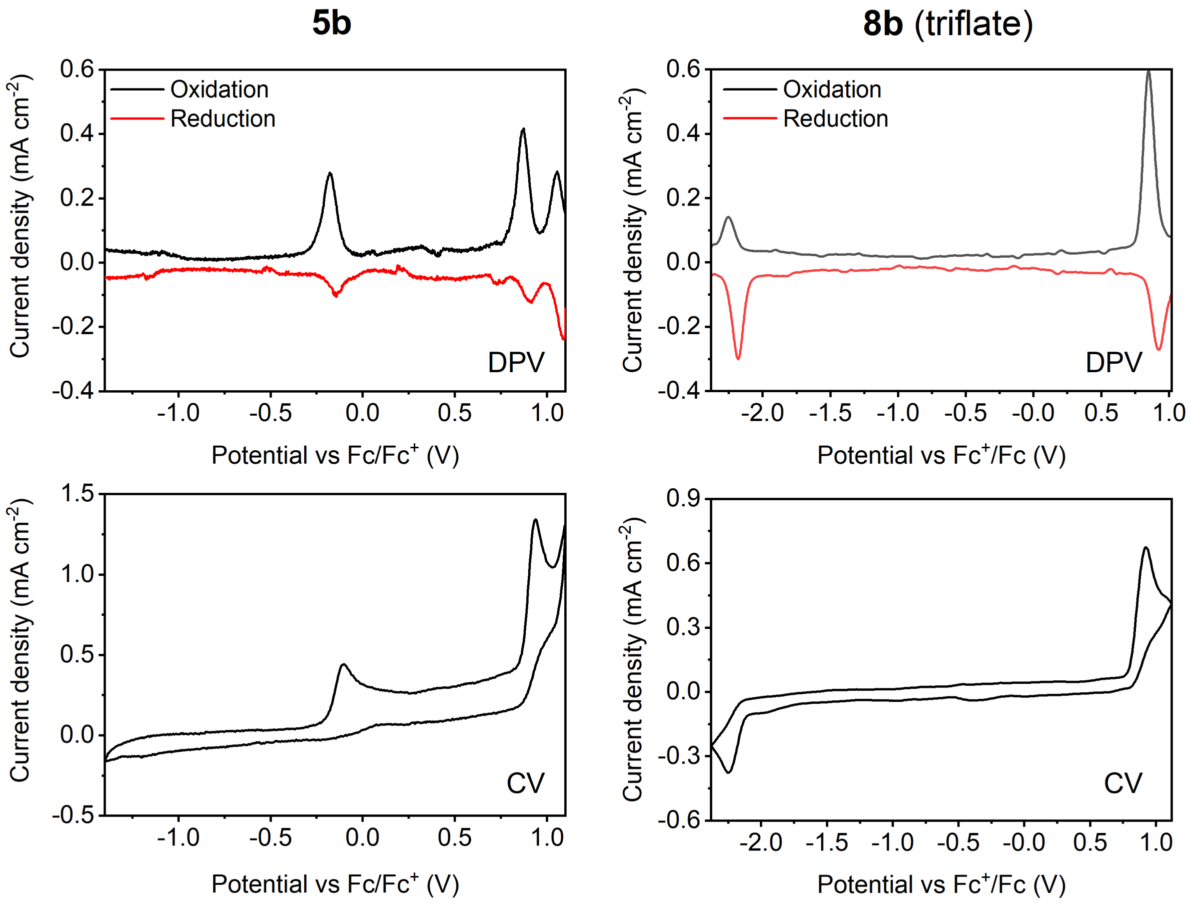

	E_{Ox} (V)	HOMO (eV)[a]	E_{Red} (V)	SOMO (eV)[a]
DPV	-0,21	-4.6 (-5.0)	-2,20	-2.6 (-3.0)
CV	-0,19	-4.6 (-5.0)	-2,17	-2.6 (-3.0)

[a]Values in brackets are evaluated using the formula: E_{vac}=-e(E_0 + 5.2 V)

5.0.3 Derivative 5c

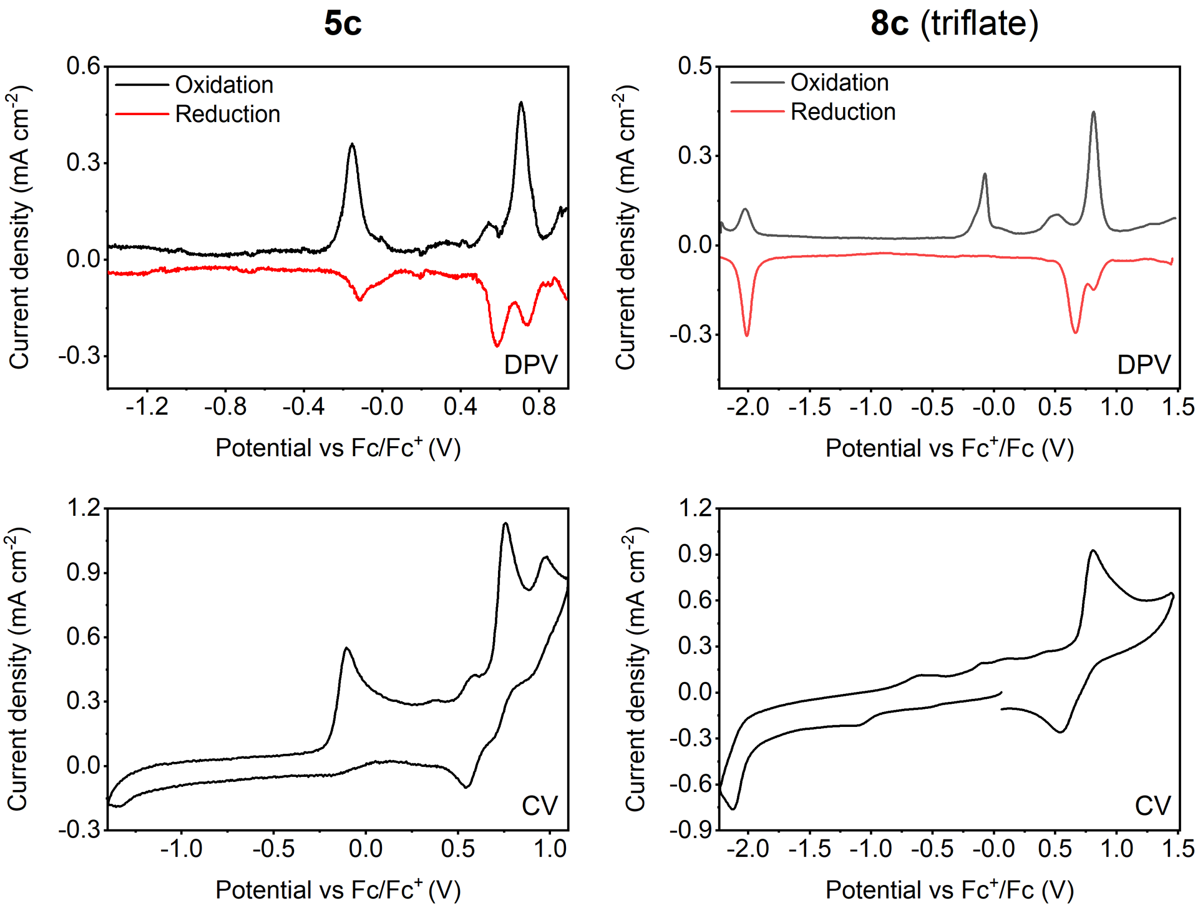

	E_{Ox} (V)	HOMO (eV)[a]	E_{Red} (V)	SOMO (eV)[a]
DPV	-0,21	-4.6 (-5.0)	-2,00	-2.8 (-3.2)
CV	-0,20	-4.6 (-5.0)	-1,99	-2.8 (-3.2)

[a]Values in brackets are evaluated using the formula: E_{vac}=-e(E_0 + 5.2 V)

5.0.4 Derivative 5d

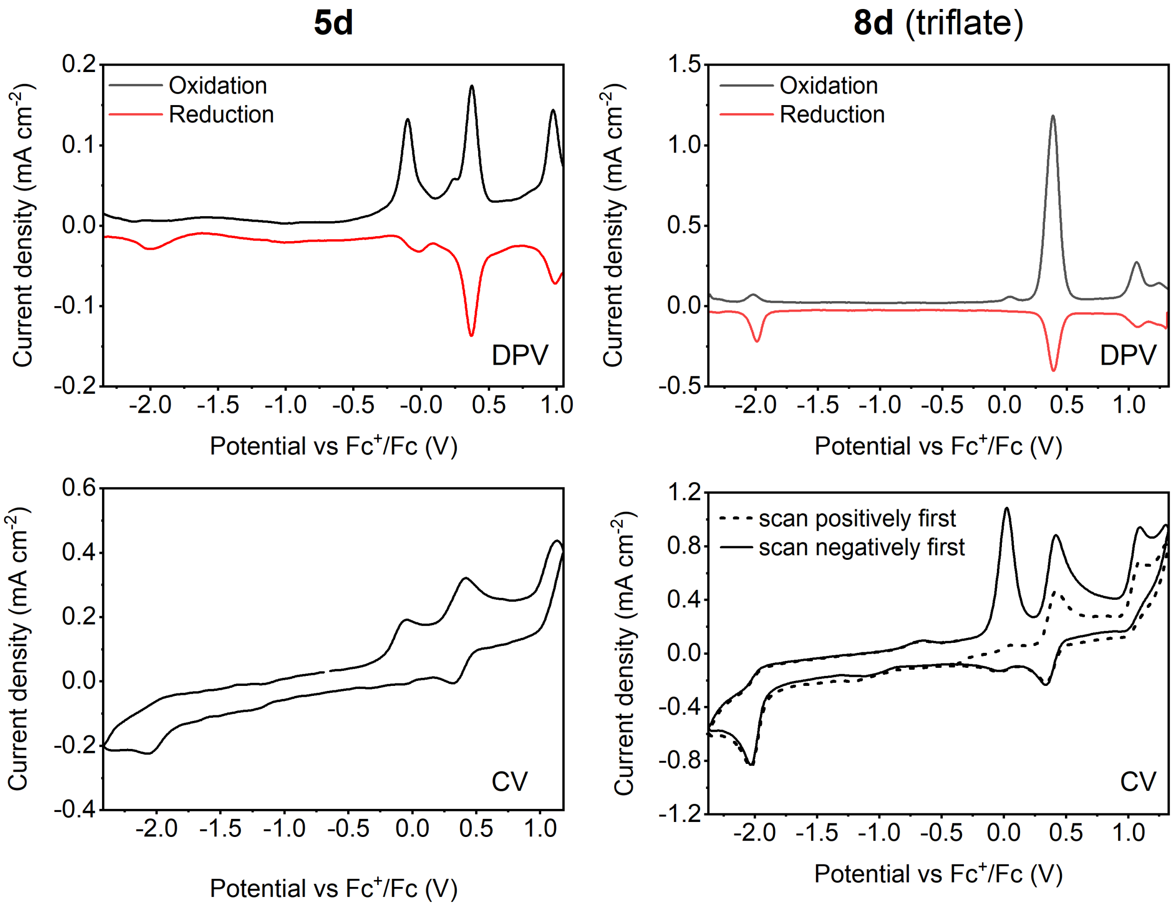

	E_{Ox} (V)	HOMO (eV)[a]	E_{Red} (V)	SOMO (eV)[a]
DPV	-0,10	-4.7 (-5.1)	-1,99	-2.8 (-3.2)
CV	-0,12	-4.7 (-5.1)	-1,98	-2.8 (-3.2)

[a]Values in brackets are evaluated using the formula: E_{vac}=-e(E_0 + 5.2 V)

5.0.5 Derivative 5e

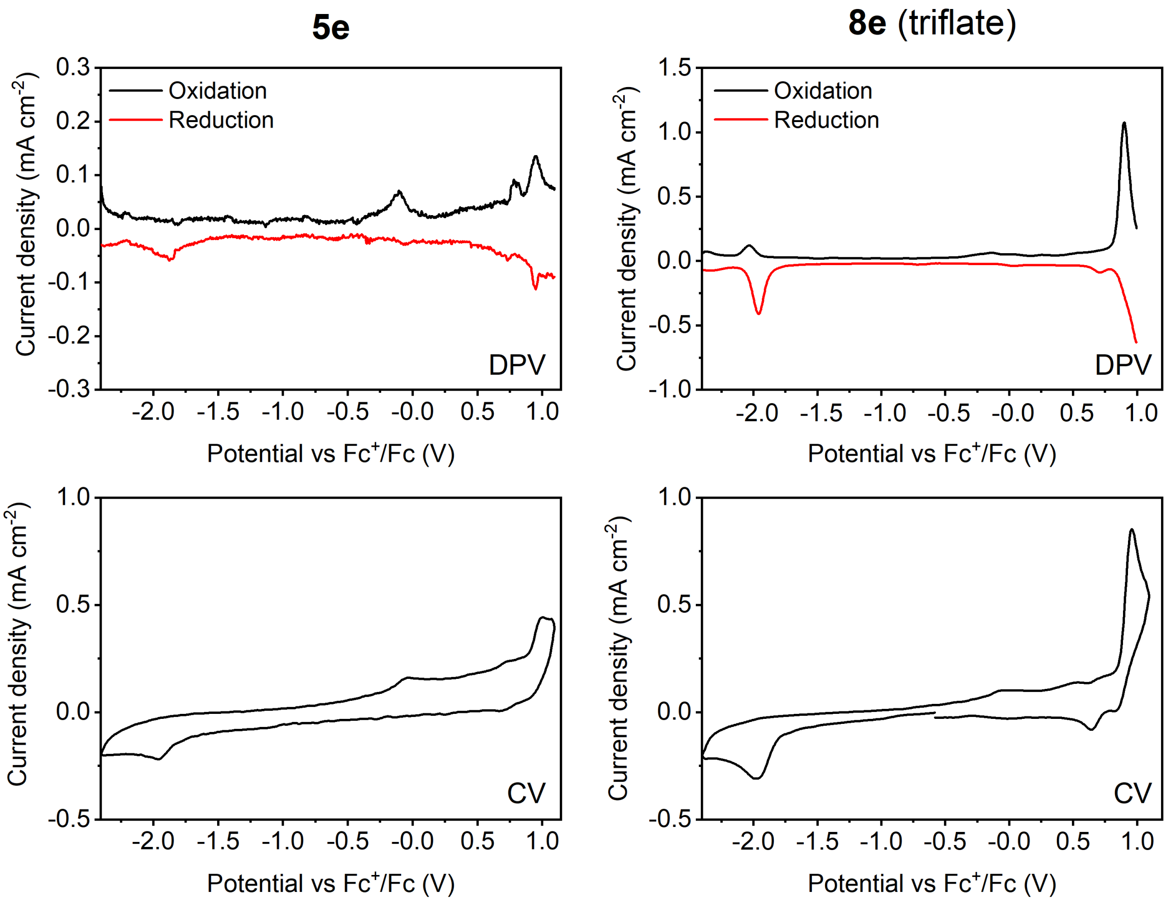

	E_{Ox} (V)	HOMO (eV)[a]	E_{Red} (V)	SOMO (eV)[a]
DPV	-0.09	-4.7 (-5.1)	-1.99	-2.8 (-3.2)
CV	-0.11	-4.7 (-5.1)	-1.90	-2.9 (-3.3)

[a]Values in brackets are evaluated using the formula: E_{vac}=-e(E_0 + 5.2 V)

5.0.6 Derivative 5f

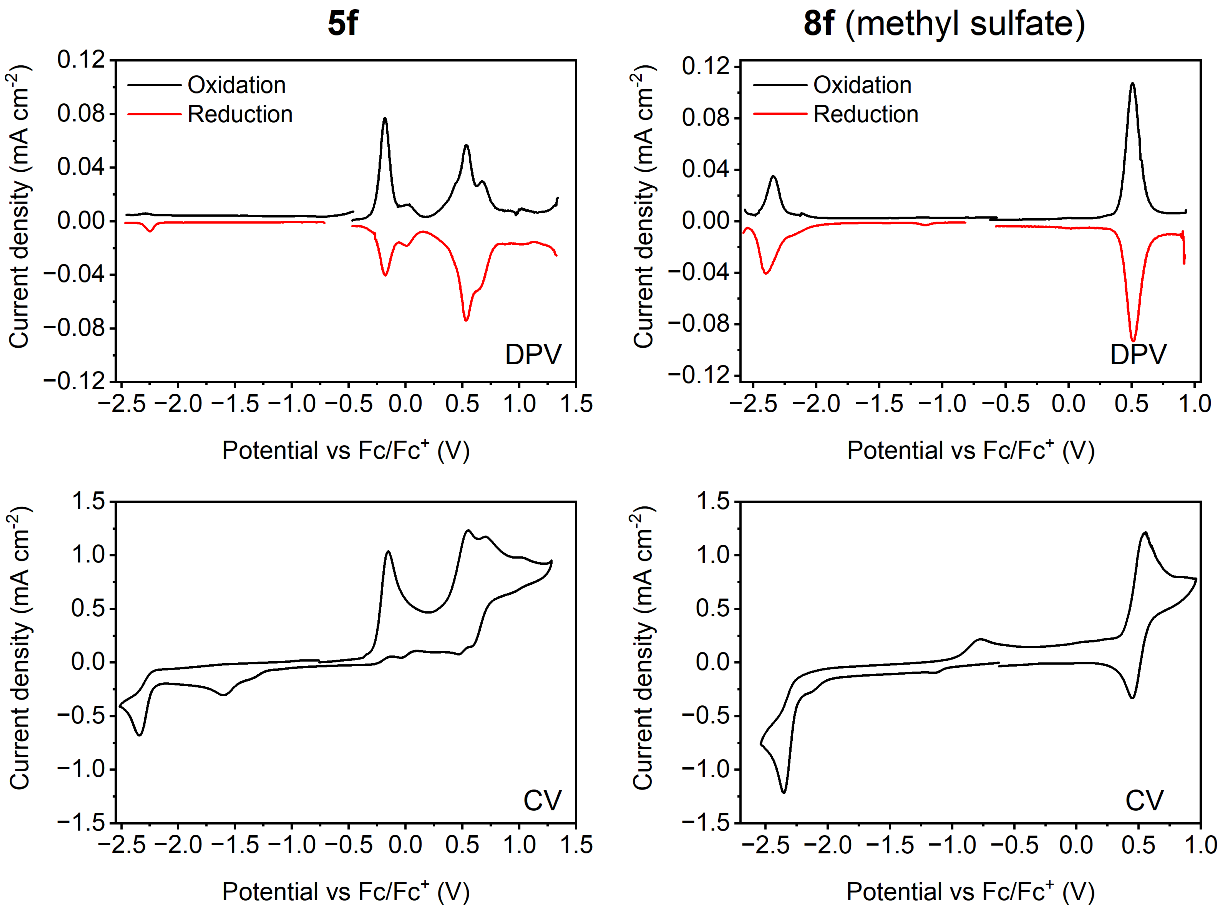

	E_{Ox} (V)	HOMO (eV)[a]	E_{Red} (V)	SOMO (eV)[a]
DPV	-0.17	-4.6 (-5.0)	-2.37	-2.4 (-2.8)
CV	-0.27	-4.5 (-4.9)	-2.22	-2.6 (-3.0)

[a]Values in brackets are evaluated using the formula: E_{vac}=-e(E_0 + 5.2 V)

5.0.7 Derivative 5g

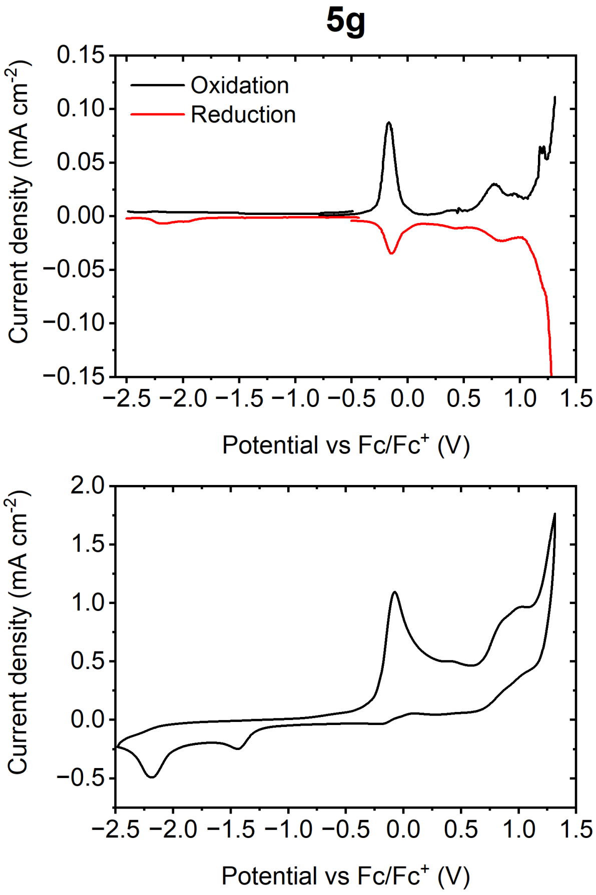

	E_{Ox} (V)	HOMO (eV)[a]
DPV	-0.16	-4.6 (-5.0)
CV	-0.24	-4.6 (-5.0)

[a]Values in brackets are evaluated using the formula: E_{vac}=-e(E_0 + 5.2 V)

5.0.8 Derivative 5h

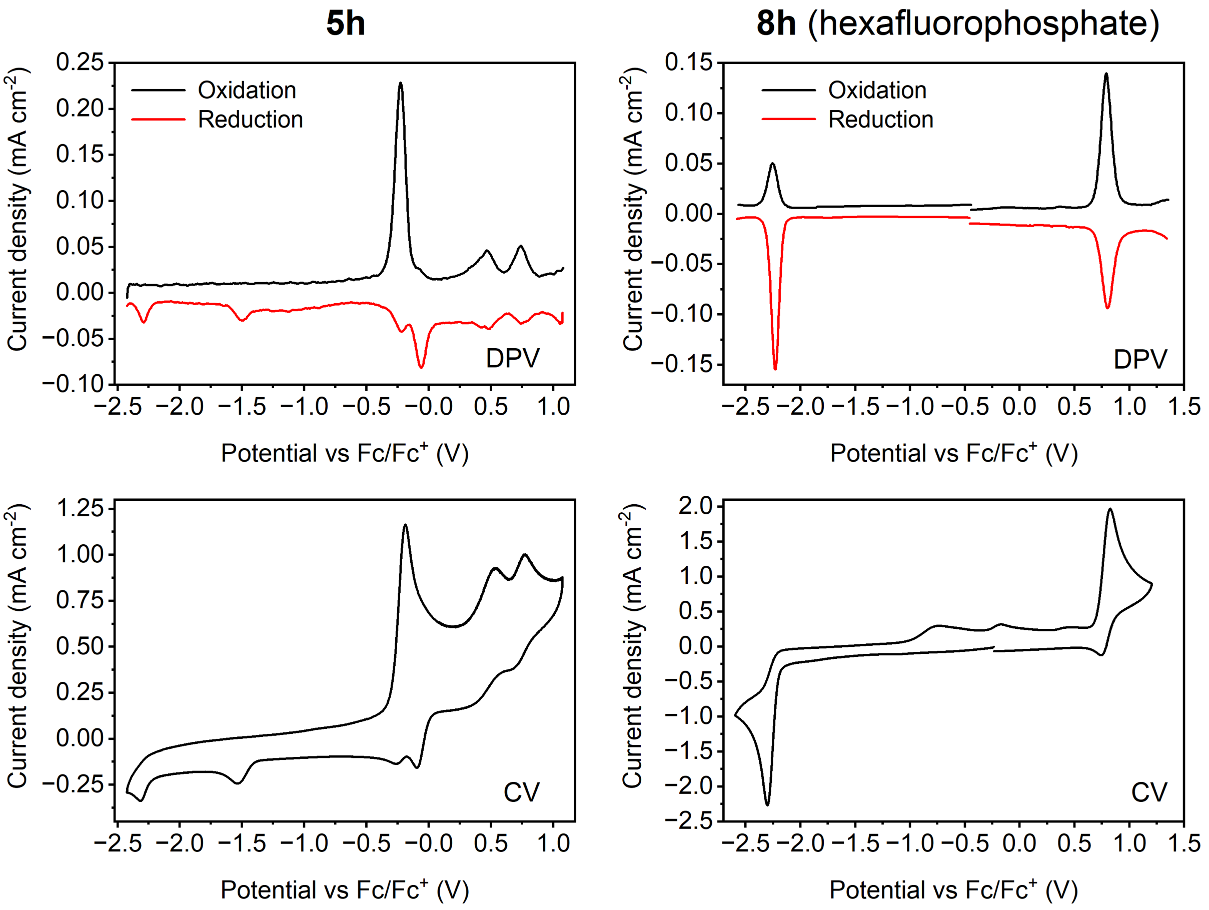

	E_{Ox} (V)	HOMO (eV)[a]	E_{Red} (V)	SOMO (eV)[a]
DPV	-0.22	-4.6 (-5.0)	-2.24	-2.6 (-3.0)
CV	-0.22	-4.6 (-5.0)	-2.19	-2.6 (-3.0)

[a]Values in brackets are evaluated using the formula: E_{vac}=-e(E_0 + 5.2 V)

5.0.9 Derivative 5i

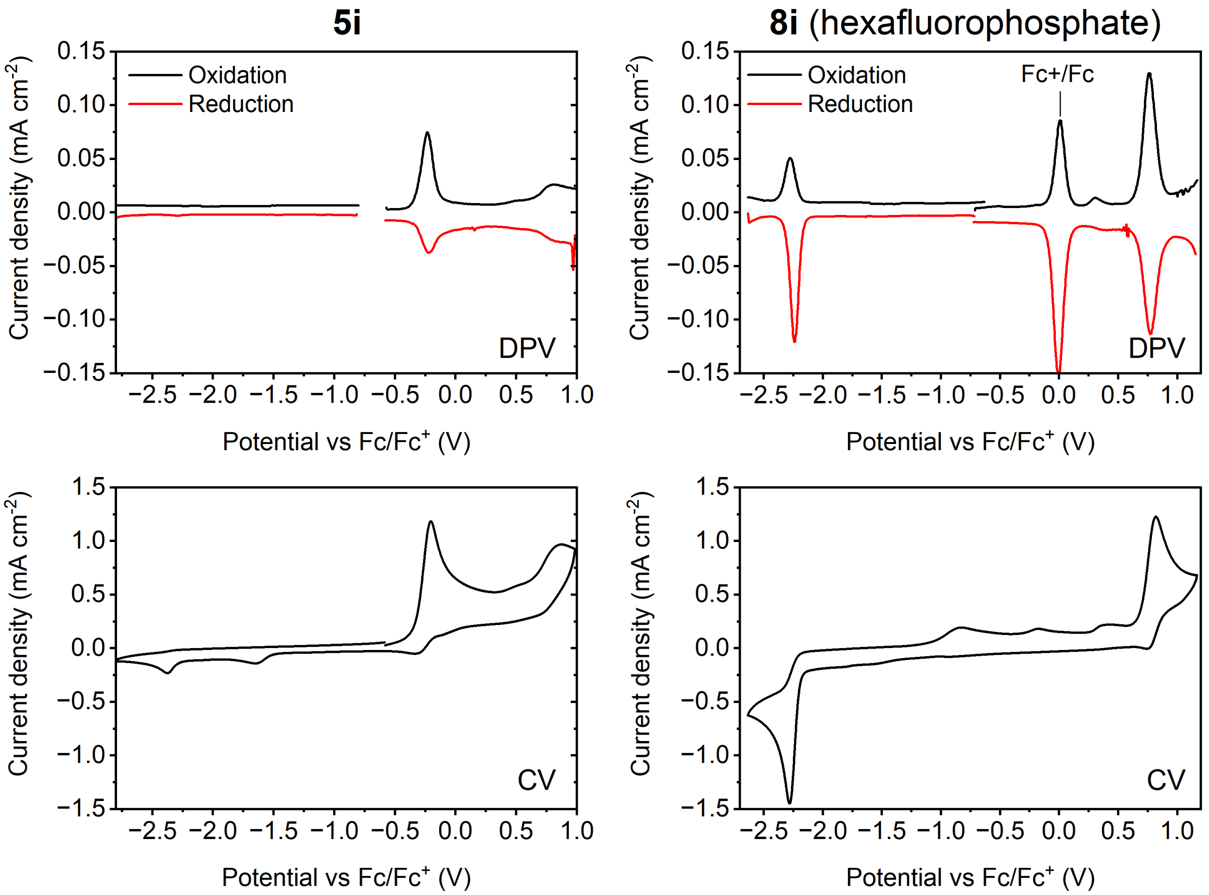

	E_{Ox} (V)	HOMO (eV)[a]	E_{Red} (V)	SOMO (eV)[a]
DPV	-0.23	-4.6 (-5.0)	-2.26	-2.5 (-2.9)
CV	-0.34	-4.5 (-4.9)	-2.18	-2.6 (-3.0)

[a]Values in brackets are evaluated using the formula: E_{vac}=-e(E_0 + 5.2 V)

5.0.10 Derivative 5l

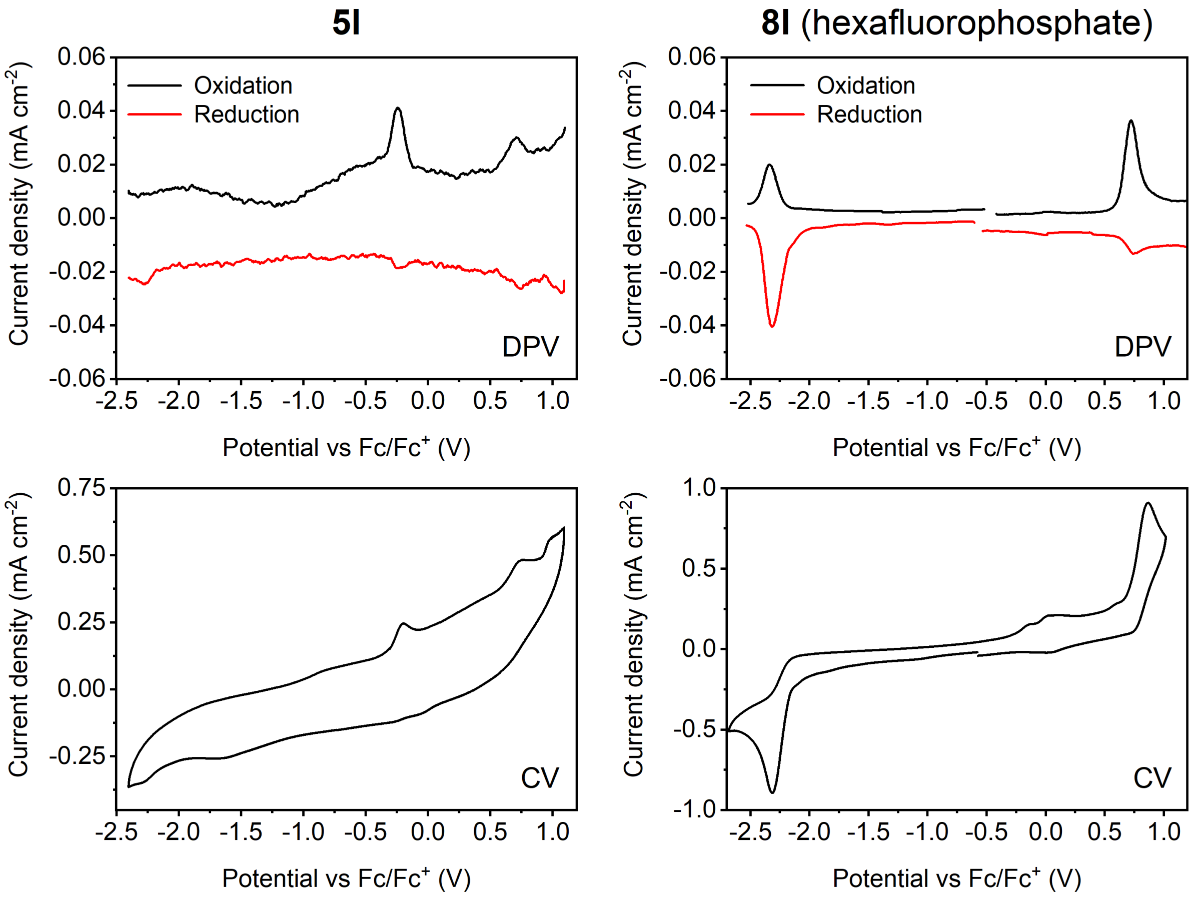

	E_{Ox} (V)	HOMO (eV)[a]	E_{Red} (V)	SOMO (eV)[a]
DPV	-0.23	-4.6 (-5.0)	-2.32	-2.5 (-2.9)
CV	-0.25	-4.6 (-5.0)	-2.13	-2.7 (-3.1)

[a]Values in brackets are evaluated using the formula: E_{vac}=-e(E_0 + 5.2 V)

5.0.11 Derivative 5m

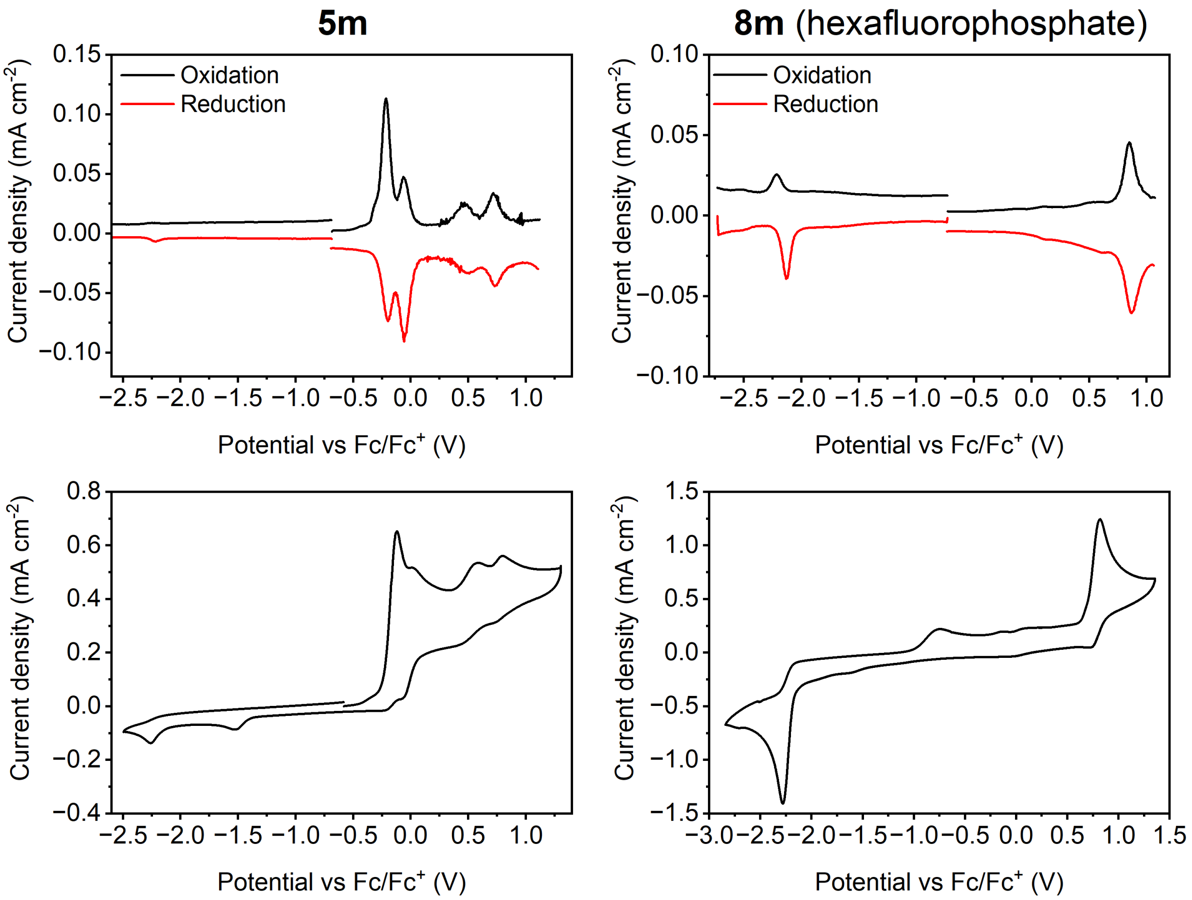

	E_{Ox} (V)	HOMO (eV)[a]	E_{Red} (V)	SOMO (eV)[a]
DPV	-0.20	-4.6 (-5.0)	-2.17	-2.6 (-3.0)
CV	-0.23	-4.6 (-5.0)	-2.15	-2.7 (-3.1)

[a]Values in brackets are evaluated using the formula: E_{vac}=-e(E_0 + 5.2 V)

5.0.12 Derivative 5n

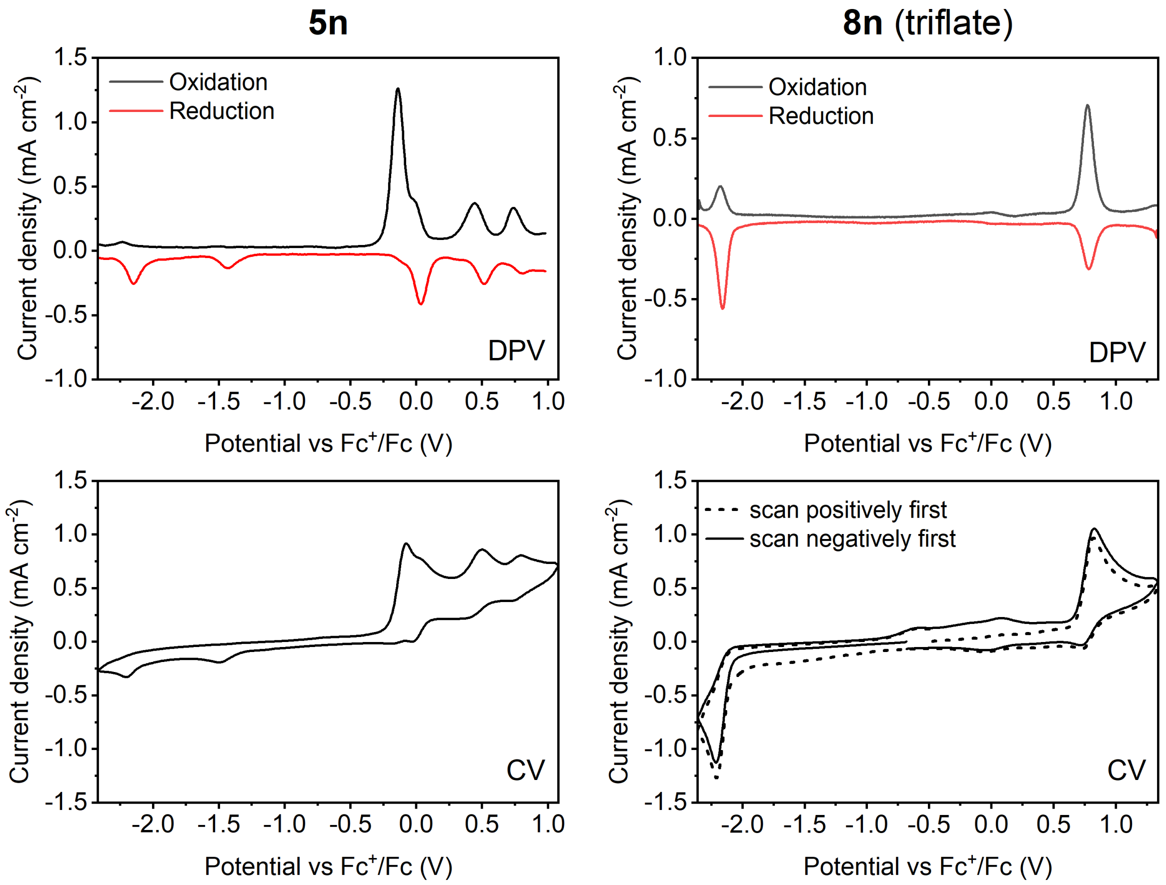

	E_{Ox} (V)	HOMO (eV)[a]	E_{Red} (V)	SOMO (eV)[a]
DPV	-0,11	-4.7 (-5.1)	-2,15	-2.7 (-3.1)
CV	-0,09	-4.7 (-5.1)	-2,13	-2.7 (-3.1)

[a]Values in brackets are evaluated using the formula: E_{vac}=-e(E_0 + 5.2 V)

6 Computational methods

Atomistic simulations of dopants have been carried out using a multi-level protocol. The most stable conformers of pristine (**R-H**) and activated (**R**·) dopant molecules have been individuated using a conformer-rotamer ensemble sampling tool (CREST),[S24] based on the GFN2-xTB tight-binding Hamiltonian as "engine" for the calculations of energies and forces.[S24,S25] The electronic properties of the most stable structures found by CREST have been investigated in the framework of density functional theory simulations, using the ORCA suite of programs.[S26,S27] In detail, the Kohn-Sham orbitals have been expanded on the all-electron def2-TZVPP Gaussian-type basis set.[S28,S29] The corresponding def2/J basis has been also used as an auxiliary basis set for Coulomb fitting in a resolution-of-identity/chain-of-spheres (RIJCOSX) level of approximation. Molecular geometries have been fully optimized using the B3LYP functional,[S30] with the addition of the pairwise D3 correction for the calculation of dispersion forces.[S31] Redox potentials of all the molecules have been calculated using the M06-2X hybrid functional[S32] and the same combination of def2-TZVPP-def2/J basis sets, with all the investigated neutral and charged species immersed in an implicit CH_2Cl_2 solvent using a conductor-like polarizable continuum model (CPCM)[S33] to calculate electronic and solvation energies. Thermochemical properties of the same molecules have been calculated using the B3LYP functional introduced above. Redox potentials have been then calculated as ΔG values between neutral and charged species.

Derivative	**R**· SOMO (B3LYP, eV)	**R-H** HOMO (B3LYP, eV)	**R**+ LUMO (B3LYP, eV)	**ΔH**[a] (M062X, eV)	**E**[·/+][b] (M062X, eV)	**E**[**0**/·+][c] (M062X, eV)
**5a**	-2.65	-4.86	-2.02	1.01	-2.54	-4.78
**5b**	-2.68	-4.88	-2.07	0.96	-2.64	-4.78
**5c**	-2.95	-4.92	-2.20	0.85	-2.80	-4.84
**5d**	-3.11	-4.98	-2.38	0.80	-2.97	-4.90
**5e**	-3.07	-4.97	-2.35	0.80	-2.95	-4.89
**5f**	-2.61	-4.85	-1.98	1.20	-2.50	-4.77
**5g**	-2.74	-4.89	-2.09	1.01	-2.65	-4.78
**5h**	-2.64	-4.64	-1.94	0.98	-2.48	-4.77
**5i**	-2.63	-4.63	-1.95	0.87	-2.48	-4.69
**5l**	-2.97	-4.55	-1.79	1.02	-2.54	-4.65
**5m**	-2.64	-4.66	-2.00	0.93	-2.52	-4.71
**5n**	-2.75	-4.65	-2.06	0.91	-2.63	-4.79

[a]Reaction: **R-H** $[CH_2Cl_2] \longrightarrow$ **R**· $[CH_2Cl_2] + \frac{1}{2} H_{2(g)}$

[b]Oxidation potential of the radical (corresponding to "SOMO" level measured by CV)

[c]Oxidation potential of the neutral dopant (corresponding to "HOMO" level measured by CV)

7 Dopants stability

Stability in chloroform in air was studied by mean of ^{1}H NMR for a subset of products. Solutions of the selected derivatives were prepared at a concentration of 10 mg mL^{-1} in deuterated chloroform and spectra were acquired immediately after preparation (around 15 minutes from dissolution) and then over time. Figure S4-9 shows the NMR spectra evolution over time for the selected derivatives, together with the structure of the plausible oxidation side products. Figure S10 shows the relative species concentration in solution over time. Note that in the case of derivatives **5d**, **5e** and **5i**, superimposition of the pristine dopant and its oxidation by-products spectra did not allow pecise assignation of NMR peaks to the indicated species. As such, relative species composition was evaluated considering only aliphatic peaks, assigned on the basis of previous works on oxidation of product **5a**.[S1,S2] Nature of the oxidation products, as well as the degradation kinetic, appear dopant dependent, as evidenced in Figure S10. In particular, derivatives **5d**, **5e** and **5m** show a discrete stability, with only 2% (**5d**, **5e**) and 5% (**5m**) of oxidized species after 42 hours in chloroform (Figure S10 A,B and F). Conversely, derivative **5f** and **5i** appear particularly sensitive to oxidation, with around 14% and 9% of oxidized species respectively after 2 hours in solution (Figure S10 C and D). Derivative **5l** shows an intermediate behavior, with around 14% of oxidized species after 15 hours.

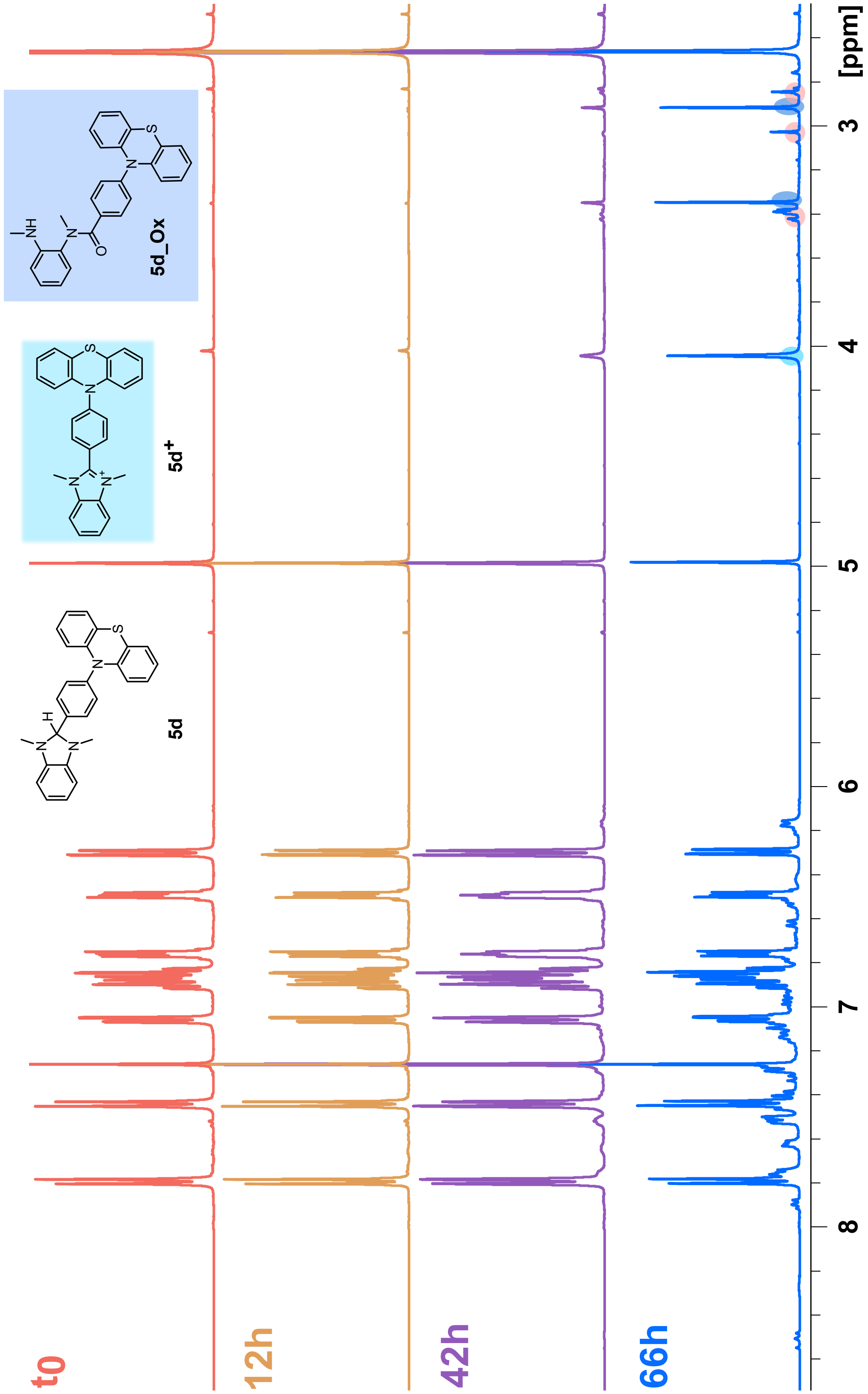

Figure S4: Evolution of ^{1}H NMR of derivative **5d** in $CDCl_3$. Peaks associated to plausible oxidation byproduct are highlighted in light blue and blue colour. Peaks associated to an unknown oxidation impurity are highlighted in pink.

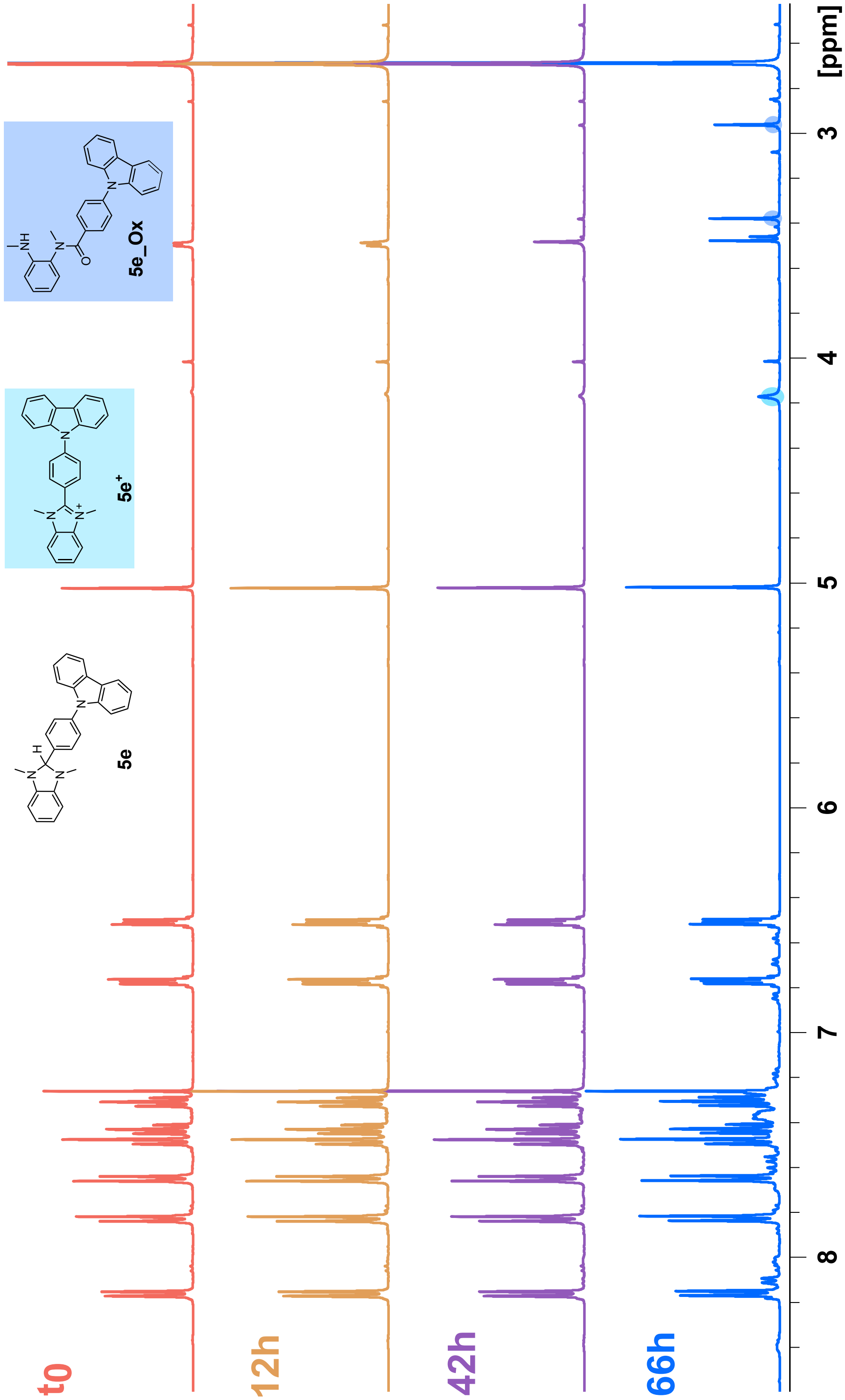

Figure S5: Evolution of ^{1}H NMR of derivative **5e** in $CDCl_3$. Peaks associated to plausible oxidation byproduct are highlighted in light blue and blue.

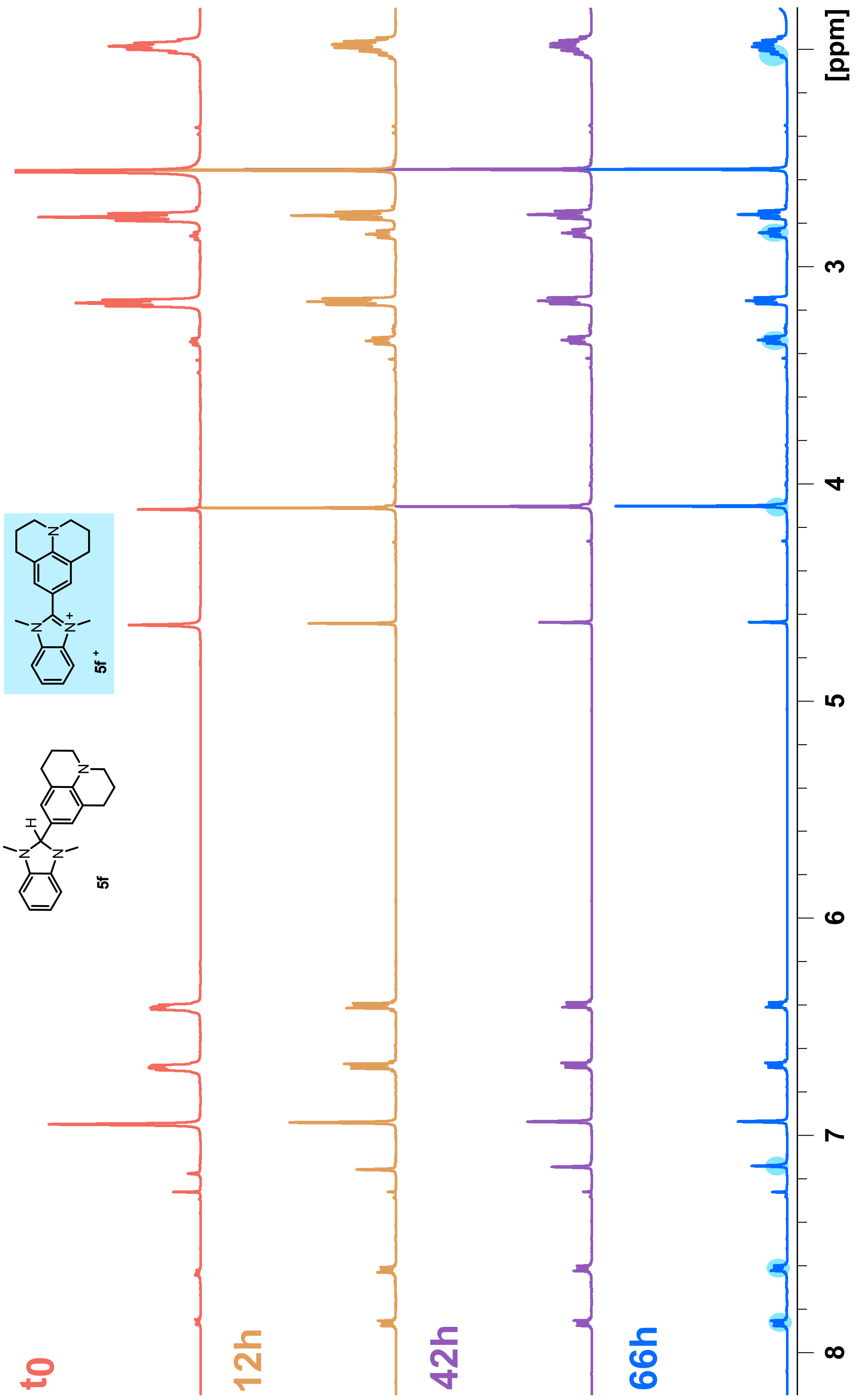

Figure S6: Evolution of ^{1}H NMR of derivative **5f** in $CDCl_3$. Peaks associated to oxidation byproduct are highlighted in light blue.

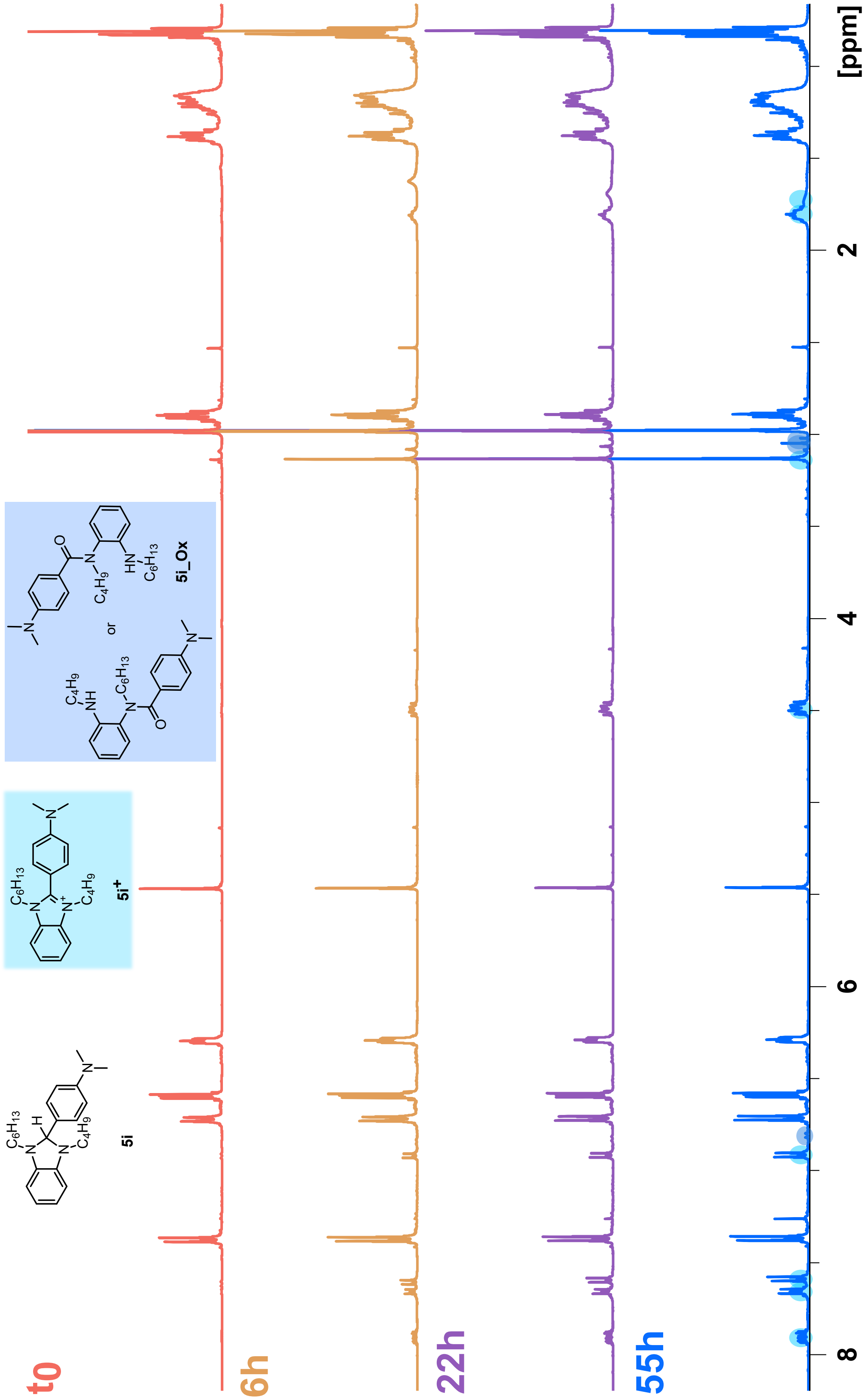

Figure S7: Evolution of ^{1}H NMR of derivative **5i** in $CDCl_3$. Peaks associated to the main oxidation byproduct **51**$^{+}$ are highlighted in light blue. Due to the low concentration of **5i_Ox** species, only easy to distinguish peaks are highlighted for this byproduct.

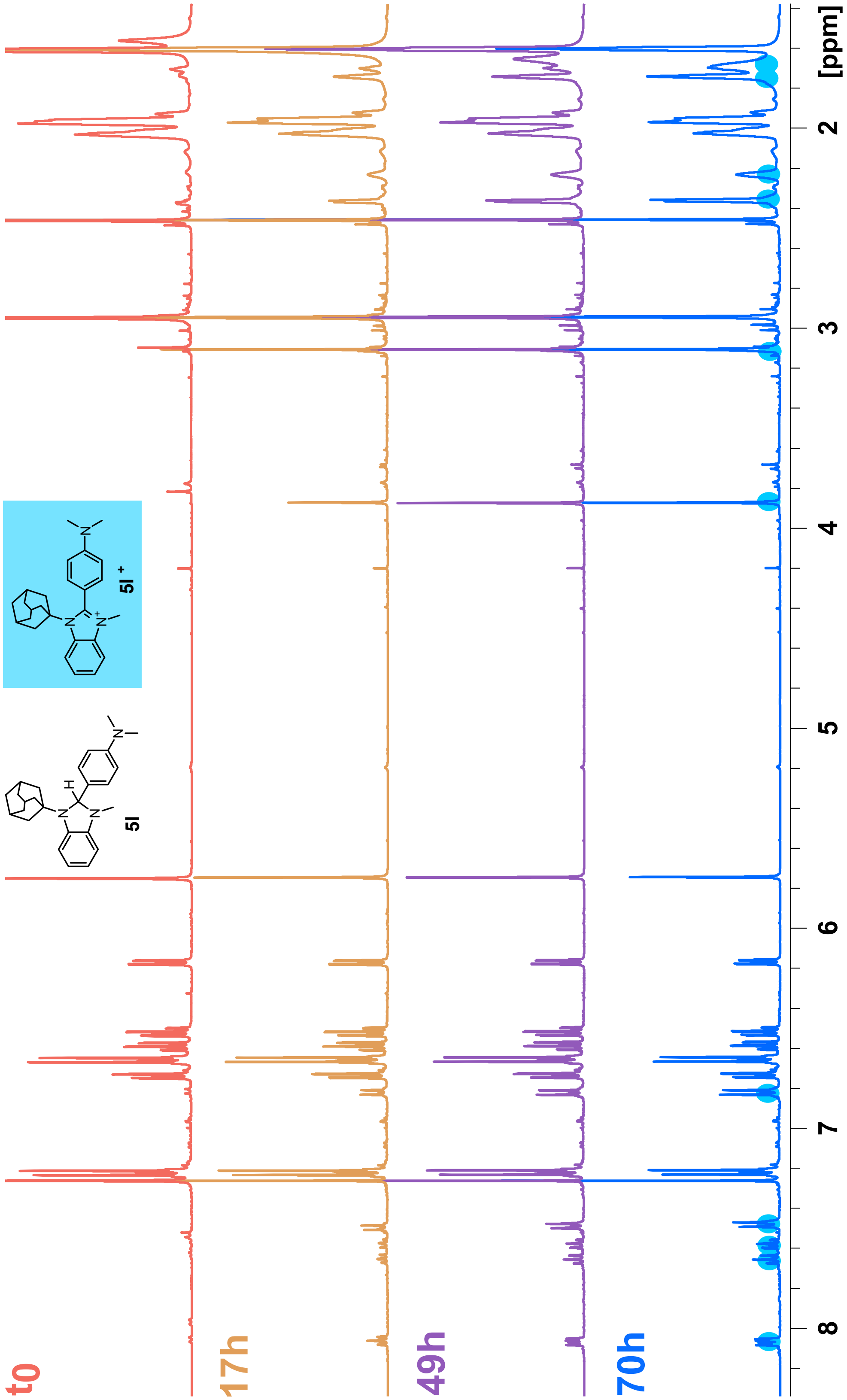

Figure S8: Evolution of ^{1}H NMR of derivative **5l** in $CDCl_3$. Peaks associated to oxidation byproduct are highlighted in light blue.

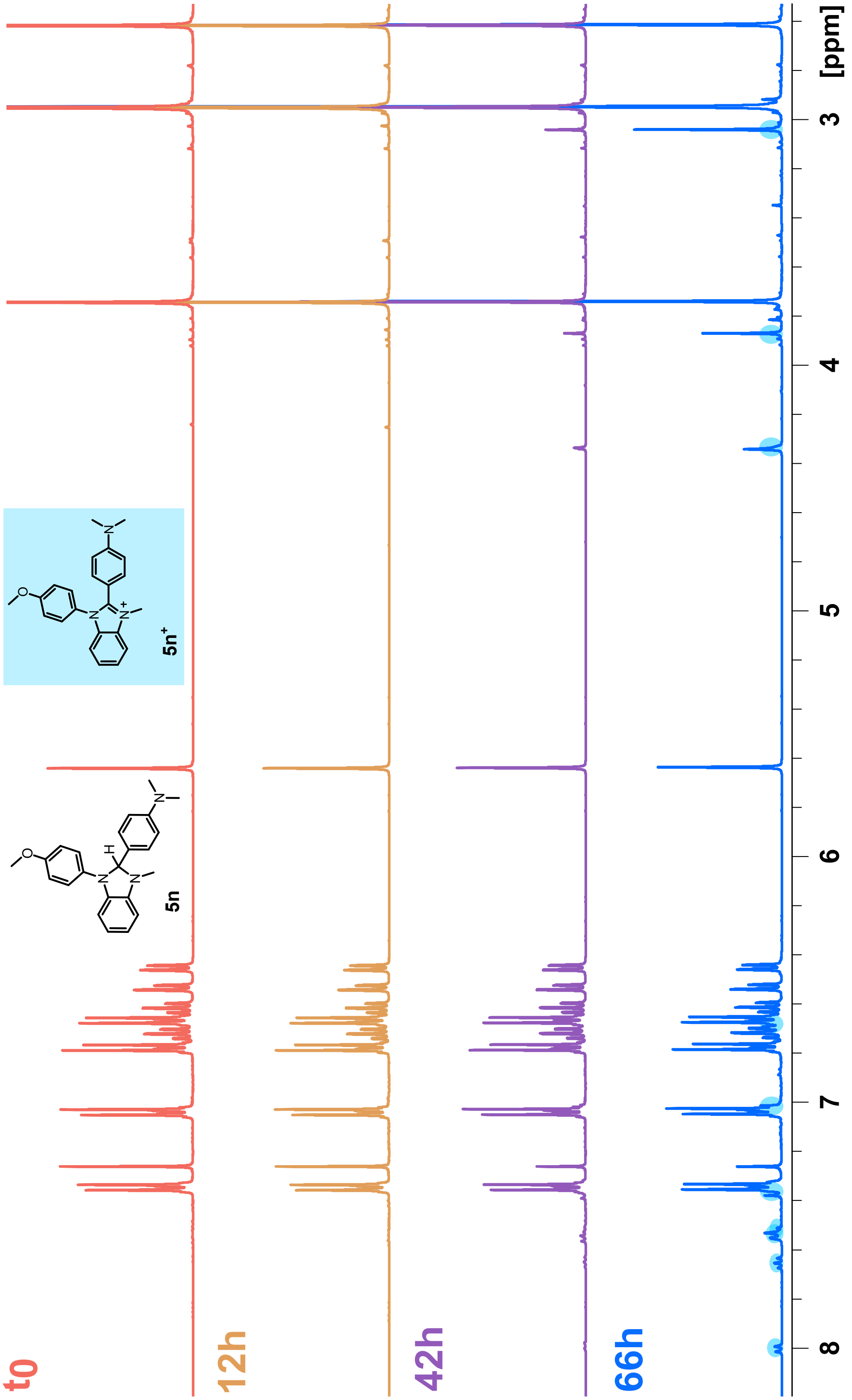

Figure S9: Evolution of ^{1}H NMR of derivative **5n** in $CDCl_3$. Peaks associated to oxidation byproduct are highlighted in light blue.

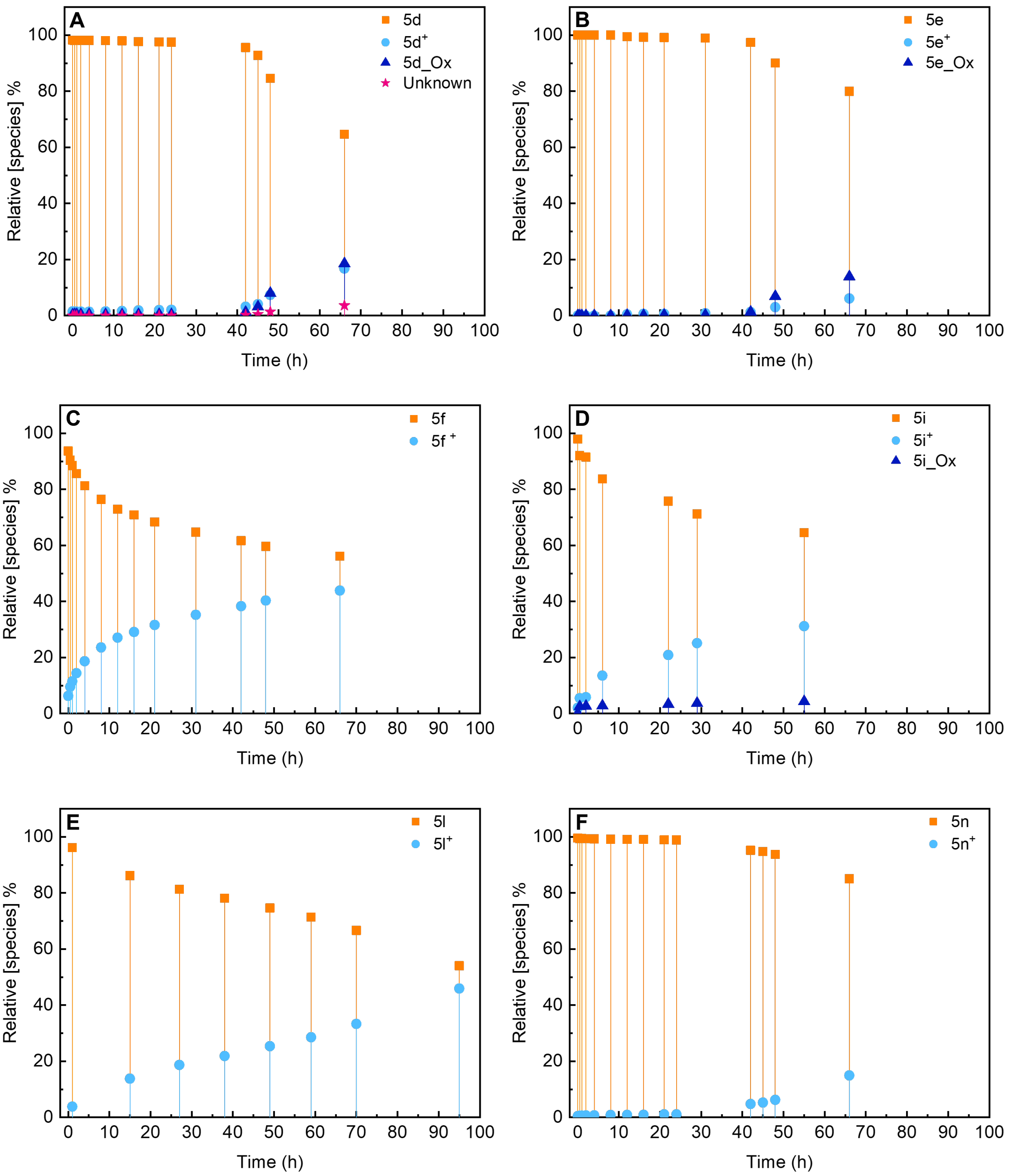

Figure S10: Degradation kinetic of products **5d** (A), **5e** (B), **5f** (C), **5i** (D), **5l** (E) and **5n** (F) dissolved in chloroform.

8 NMR spectra

- Derivative **2m**, ^{1}H NMR: S-99
- Derivative **2m**, ^{13}C NMR: S-100
- Derivative **2p**, ^{1}H NMR: S-101
- Derivative **3m**, ^{1}H NMR: S-102
- Derivative **3m**, ^{13}C NMR: S-103
- Derivative **3p**, ^{1}H NMR: S-104
- Derivative **3p**, ^{13}C NMR: S-105
- Derivative **7b**, ^{1}H NMR: S-106
- Derivative **7b**, ^{13}C NMR: S-107
- Derivative **7d**, ^{1}H NMR: S-108
- Derivative **7d**, ^{13}C NMR: S-109
- Derivative **7f**, ^{1}H NMR: S-110
- Derivative **7f**, ^{13}C NMR: S-111
- Derivative **7h**, ^{1}H NMR: S-112
- Derivative **7h**, ^{13}C NMR: S-113
- Derivative **7l**, ^{1}H NMR: S-114
- Derivative **7l**, ^{13}C NMR: S-115
- Derivative **7m**, ^{1}H NMR: S-116
- Derivative **7m**, ^{13}C NMR: S-117

- Derivative **7n**, ^{1}H NMR: S-118
- Derivative **7n**, ^{13}C NMR: S-119
- Derivative **8c**, ^{1}H NMR: S-120
- Derivative **8c**, ^{13}C NMR: S-121
- Derivative **8d**, ^{1}H NMR: S-122
- Derivative **8d**, ^{13}C NMR: S-123
- Derivative **8d**, ^{19}F NMR: S-124
- Derivative **8e**, ^{1}H NMR: S-125
- Derivative **8e**, ^{13}C NMR: S-126
- Derivative **8e**, ^{19}F NMR: S-127
- Derivative **8f** (iodide salt), ^{1}H NMR: S-128
- Derivative **8f** (iodide salt), ^{13}C NMR: S-129
- Derivative **8f** (methylsulfate salt), ^{1}H NMR: S-130
- Derivative **8f** (methylsulfate salt), ^{13}C NMR: S-131
- Derivative **8h**, ^{1}H NMR: S-132
- Derivative **8h**, ^{13}C NMR: S-133
- Derivative **8i**, ^{1}H NMR: S-134
- Derivative **8i**, ^{13}C NMR: S-135
- Derivative **8l**, ^{1}H NMR: S-136
- Derivative **8l**, ^{13}C NMR: S-137

- Derivative **8m**, ^{1}H NMR: S-138
- Derivative **8m**, ^{13}C NMR: S-139
- Derivative **8n**, ^{1}H NMR: S-140
- Derivative **8n**, ^{13}C NMR: S-141
- Derivative **5a**, ^{1}H NMR: S-142
- Derivative **5b**, ^{1}H NMR: S-143
- Derivative **5c**, ^{1}H NMR: S-144
- Derivative **5c**, ^{13}C NMR: S-145
- Derivative **5d**, ^{1}H NMR: S-146
- Derivative **5d**, ^{13}C NMR: S-147
- Derivative **5e**, ^{1}H NMR: S-148
- Derivative **5e**, ^{13}C NMR: S-149
- Derivative **5f**, ^{1}H NMR: S-150
- Derivative **5g**, ^{1}H NMR: S-151
- Derivative **5g**, ^{13}C NMR: S-152
- Derivative **5h**, ^{1}H NMR: S-153
- Derivative **5h**, ^{13}C NMR: S-154
- Derivative **5i**, ^{1}H NMR: S-155
- Derivative **5i**, ^{13}C NMR: S-156
- Derivative **5l**, ^{1}H NMR: S-157

- Derivative **5l**, ^{13}C NMR: S-158
- Derivative **5m**, ^{1}H NMR: S-159
- Derivative **5m**, ^{13}C NMR: S-160
- Derivative **5n**, ^{1}H NMR: S-161
- Derivative **5n**, ^{13}C NMR: S-162

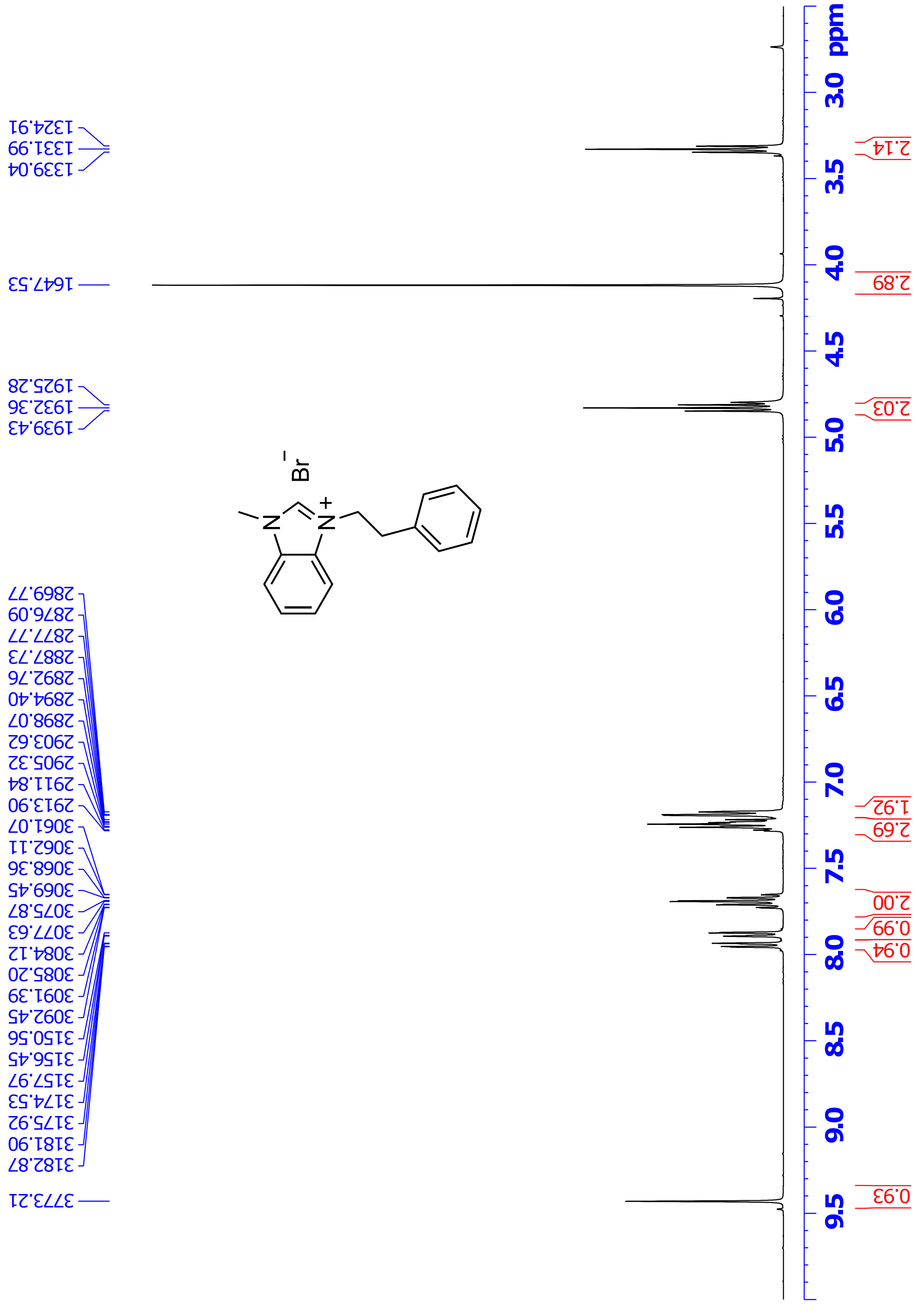

Figure S11: ^{1}H NMR of derivative **2m** in CD_3OD

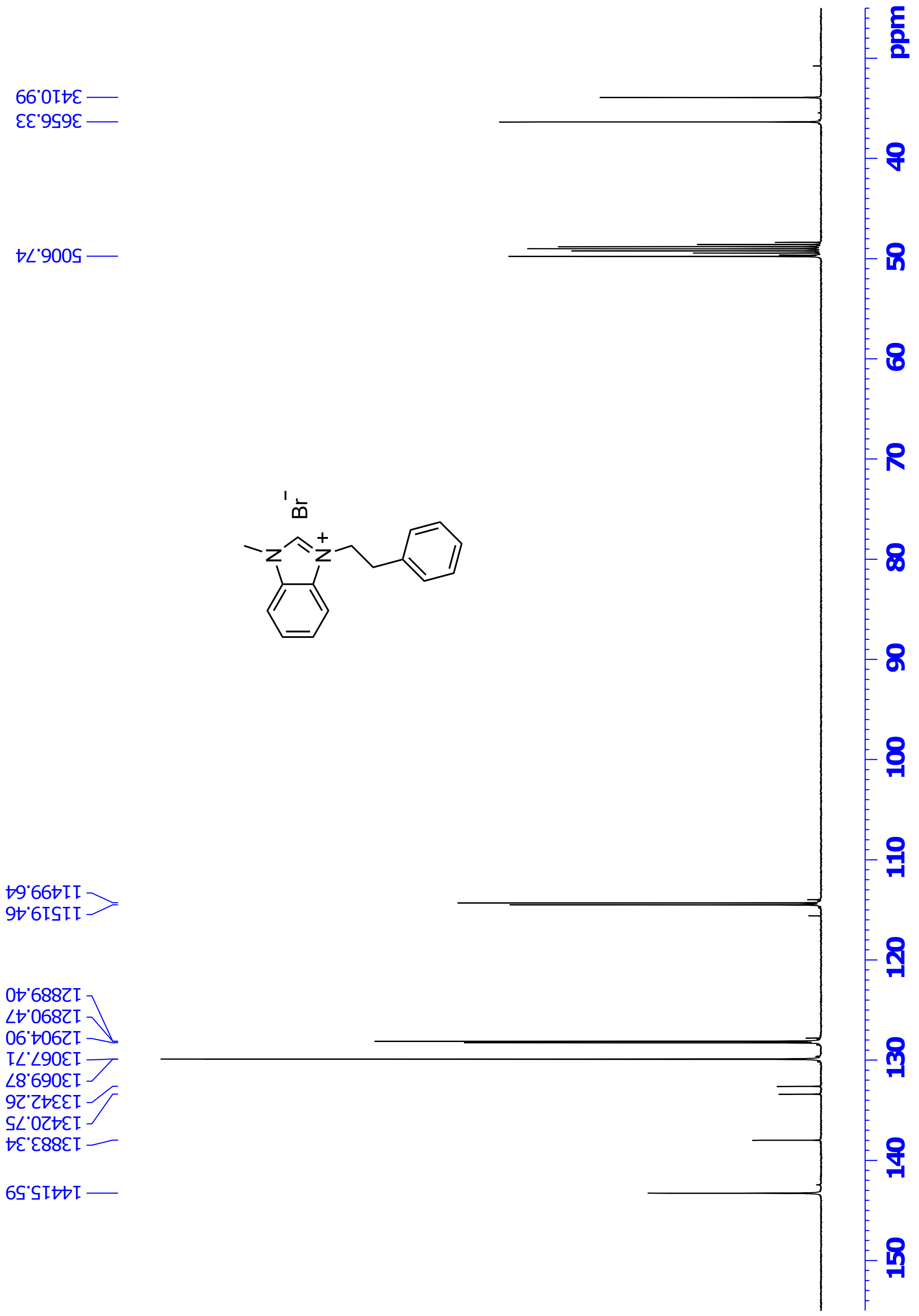

Figure S12: $^{13}C\{^{1}H\}$ NMR of derivative **2m** in CD_3OD

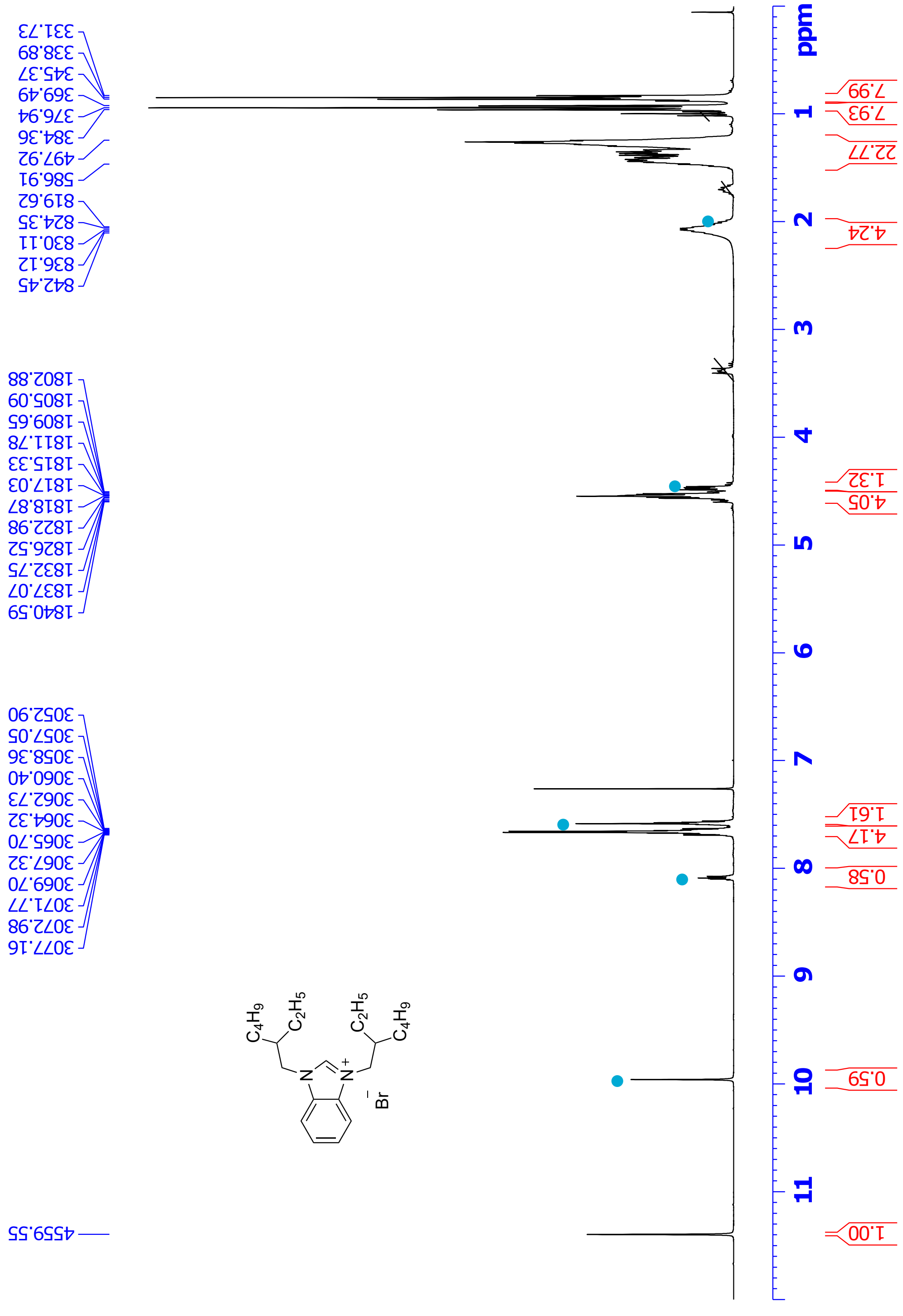

Figure S13: ^{1}H NMR of derivative **2p** in $CDCl_3$. Blue dots highlight peaks associated to 1-ethylhexylbenzimidazolium bromide. Integrals of peaks in the aliphatic region results overabundant due to superimposition with peaks associated to this impurity.

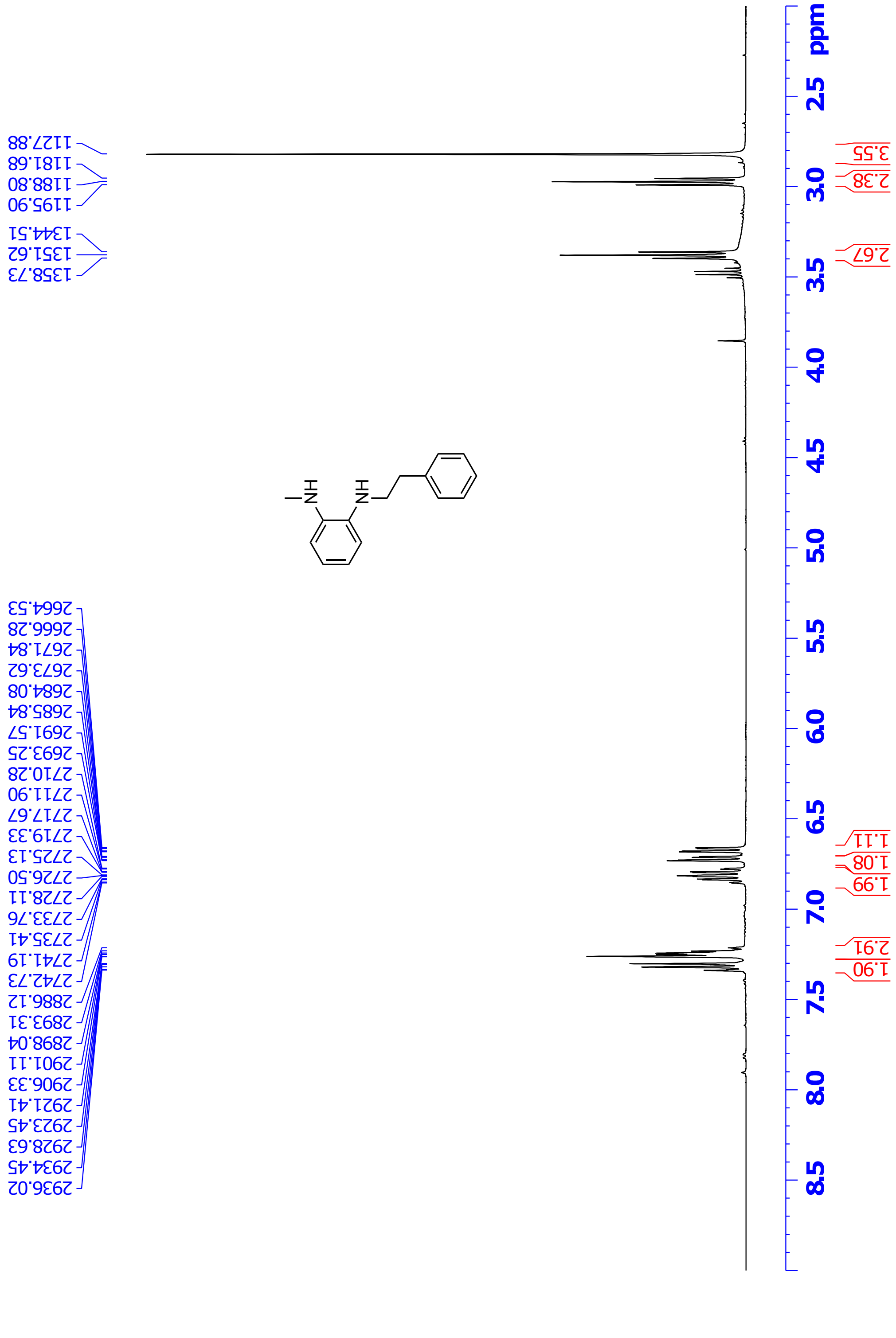

Figure S14: ^{1}H NMR of derivative **3m** in $CDCl_3$. Peak at 3.48 ppm (q) is associated to residual Et_2O.

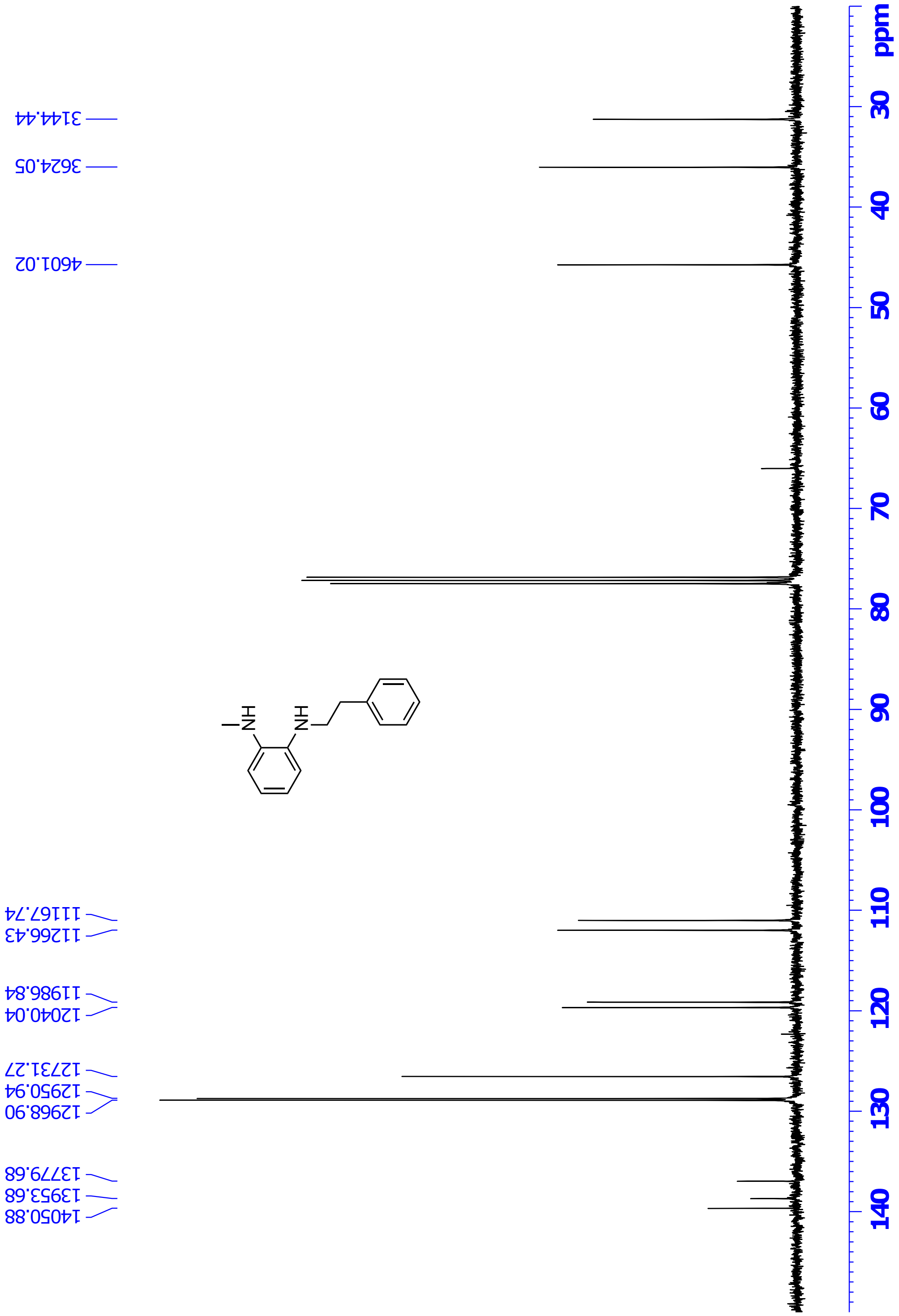

Figure S15: $^{13}C\{^{1}H\}$ NMR of derivative **3m** in $CDCl_3$. Peak at 65.91 ppm is associated to residual Et_2O.

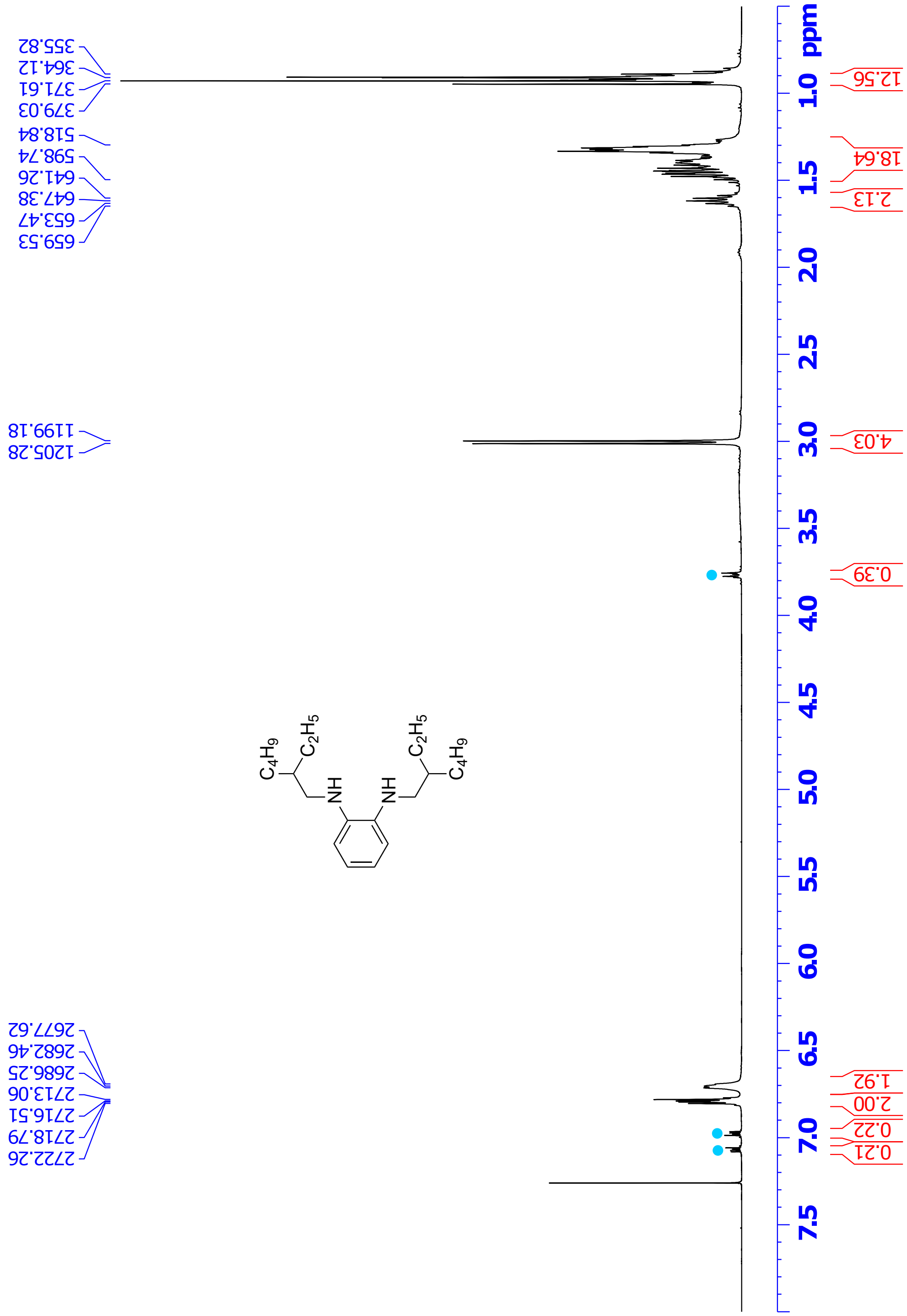

Figure S16: ^{1}H NMR of derivative **3p** in $CDCl_3$. Peaks associated to an impurity are highlighted with light blue dots.

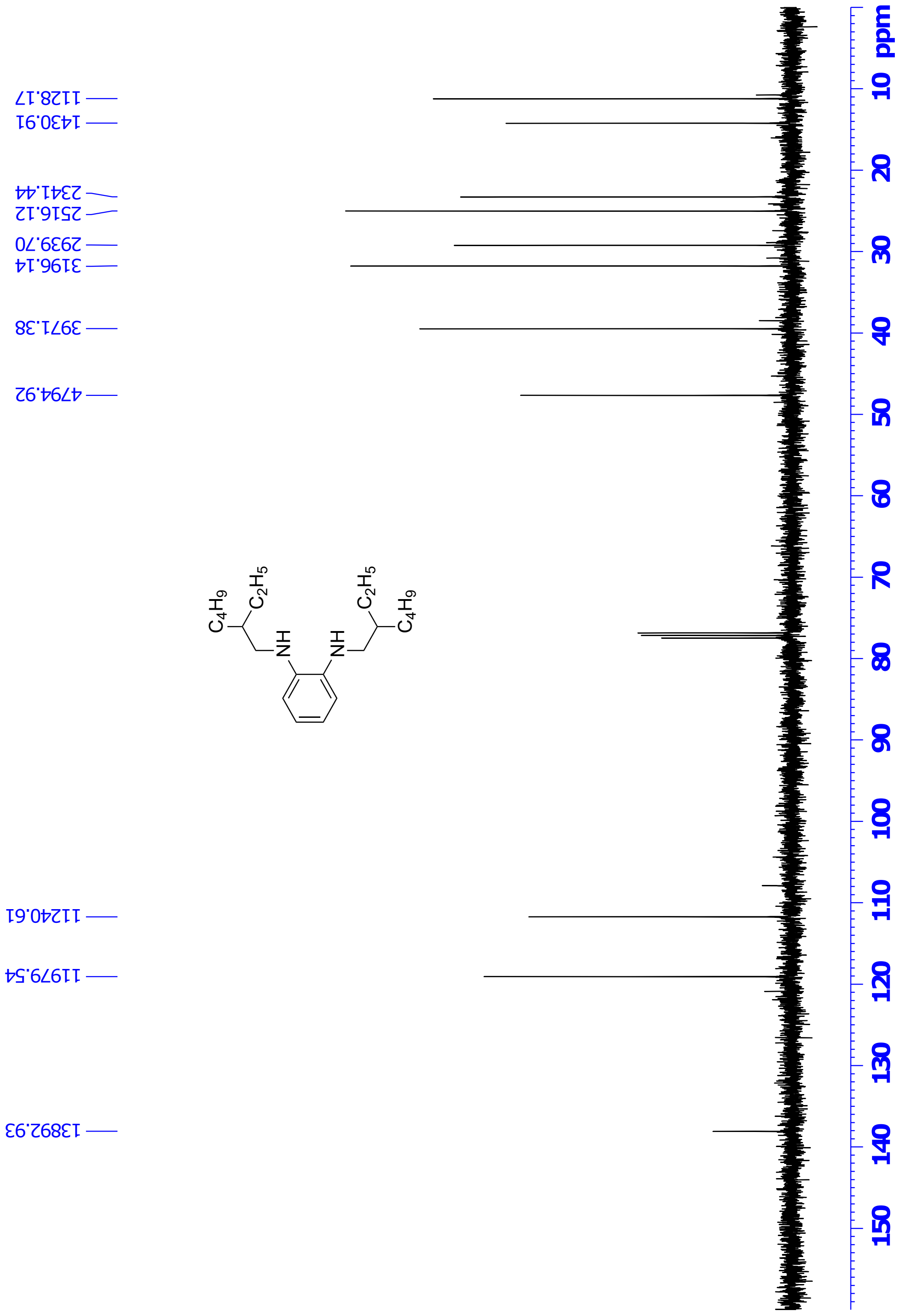

Figure S17: $^{13}C\{^{1}H\}$ NMR of derivative **3p** in $CDCl_3$.

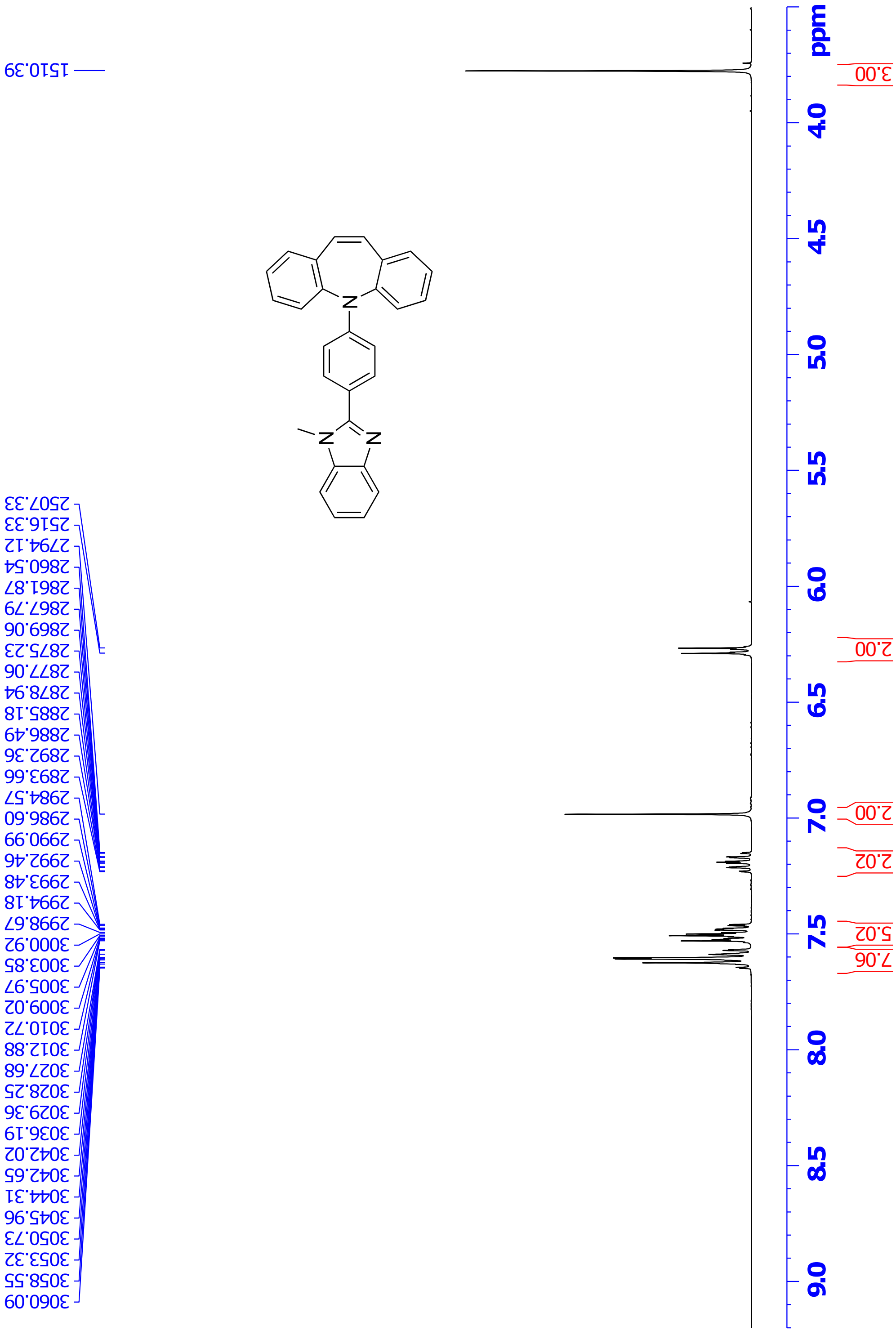

Figure S18: ^{1}H NMR of derivative **7b** in DMSO$-$d$_6$.

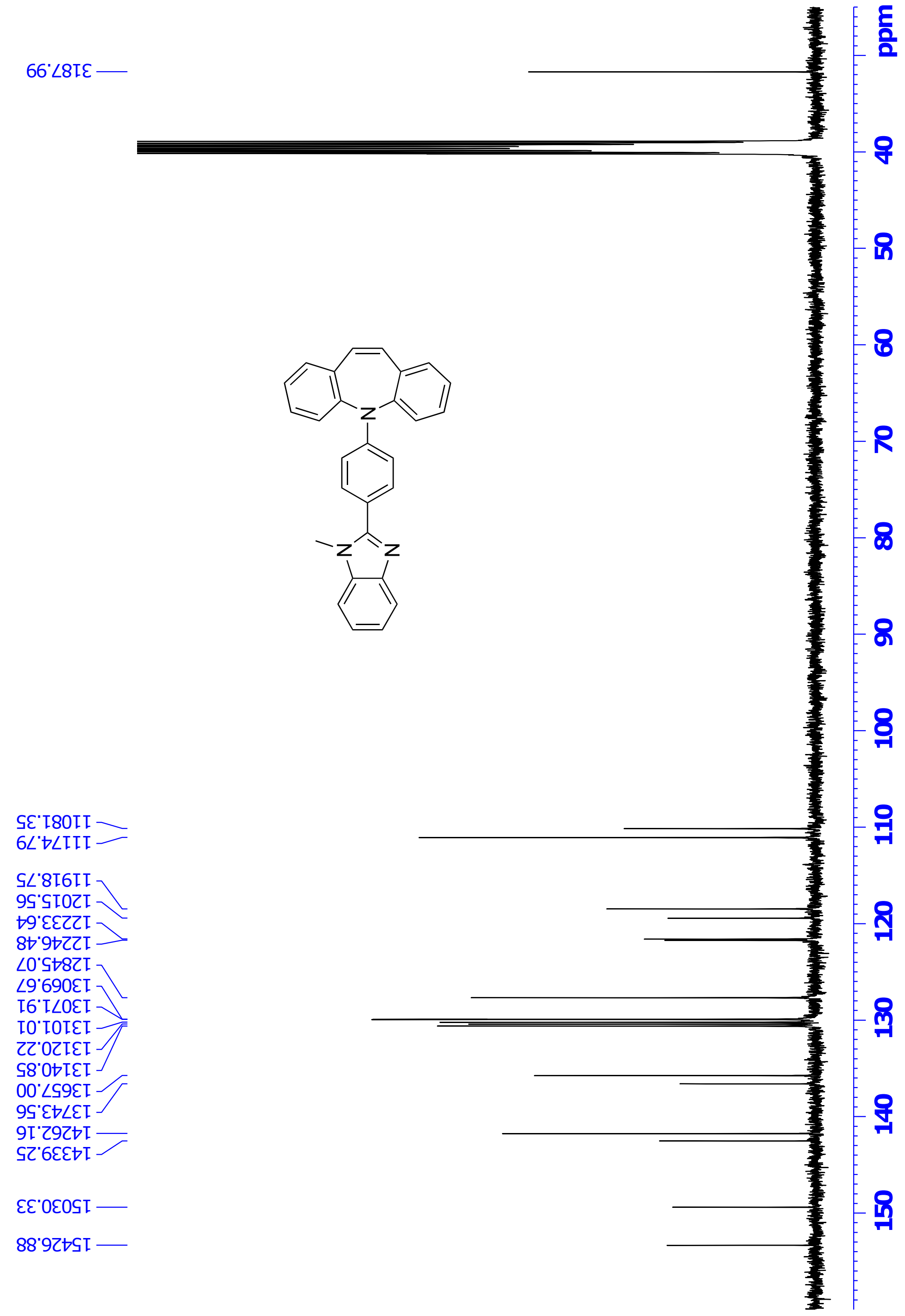

Figure S19: $^{13}C\{^{1}H\}$ NMR of derivative **7b** in DMSO$-d_6$.

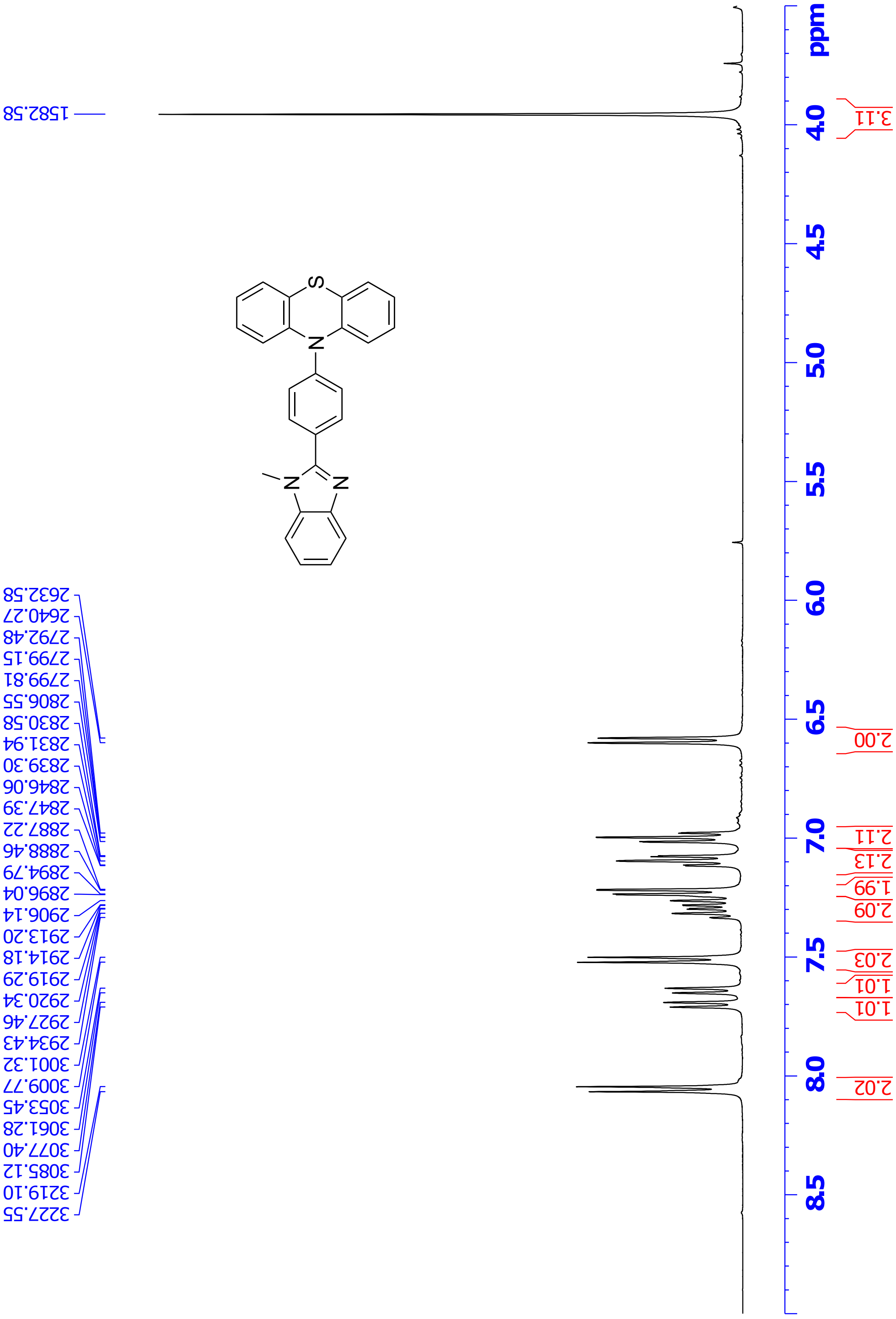

Figure S20: ^{1}H NMR of derivative **7d** in DMSO−d_6.

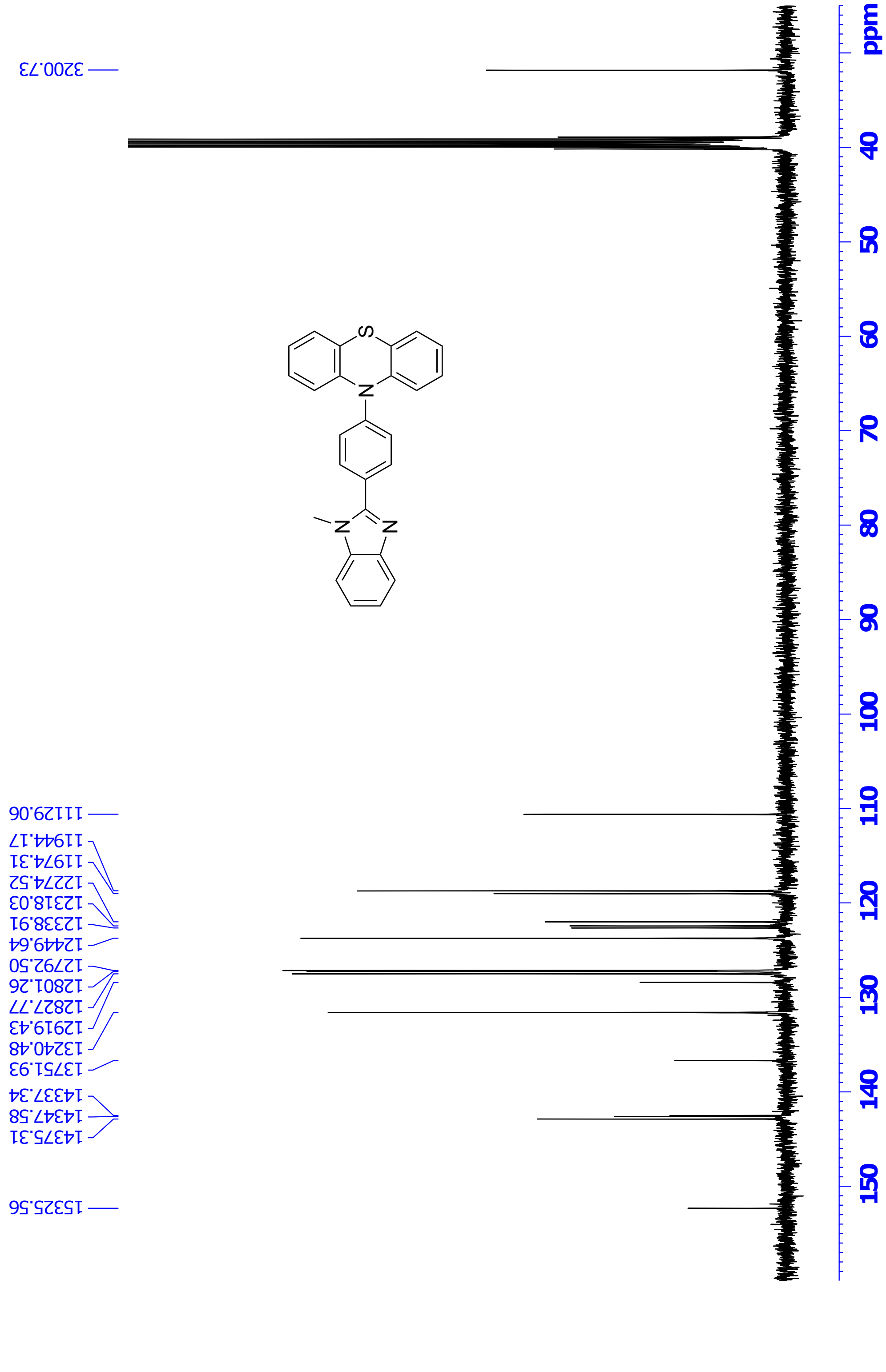

Figure S21: $^{13}C\{^{1}H\}$ NMR of derivative **7d** in DMSO$-d_6$.

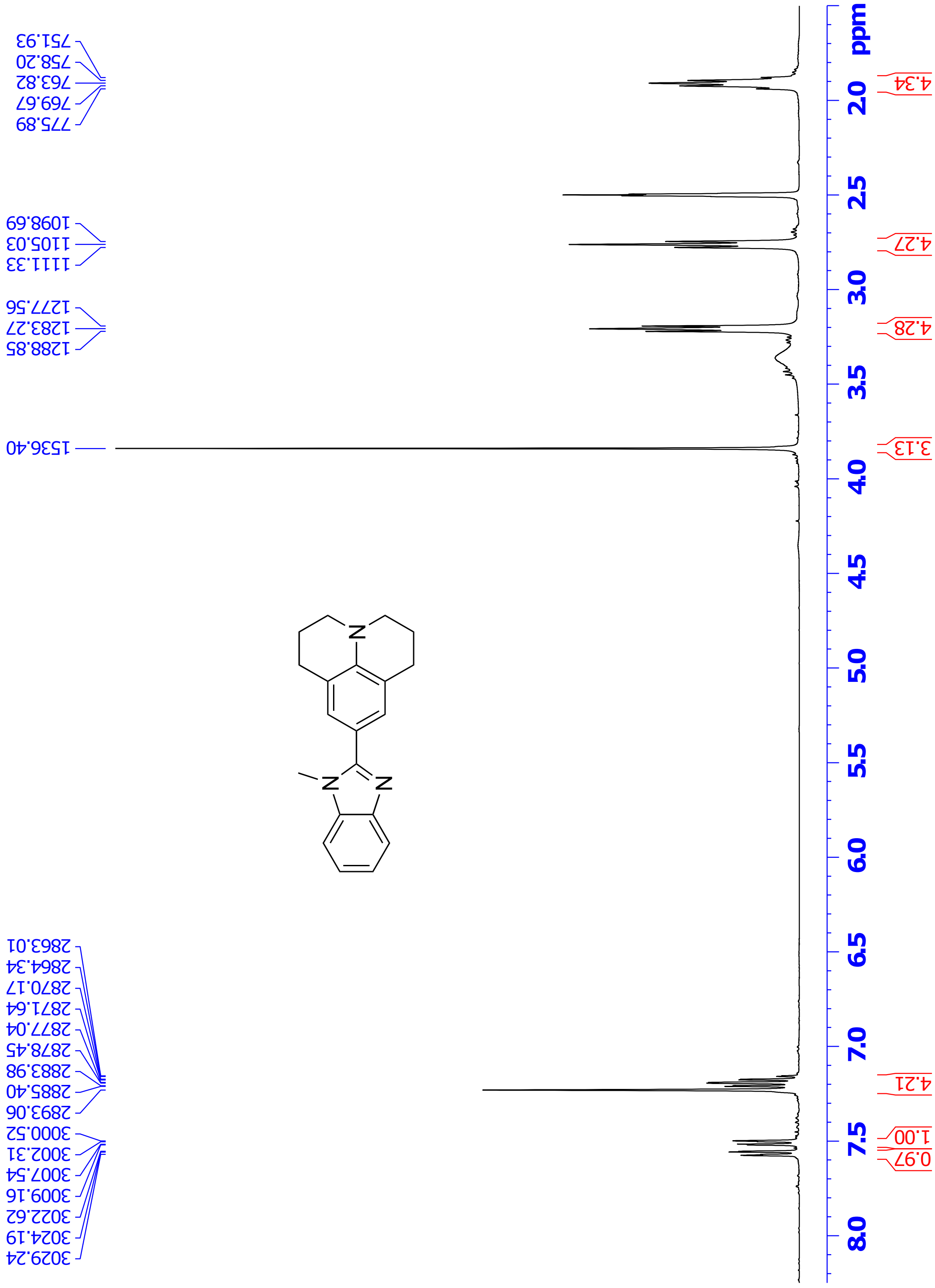

Figure S22: ^{1}H NMR of derivative **7f** in DMSO−d_6.

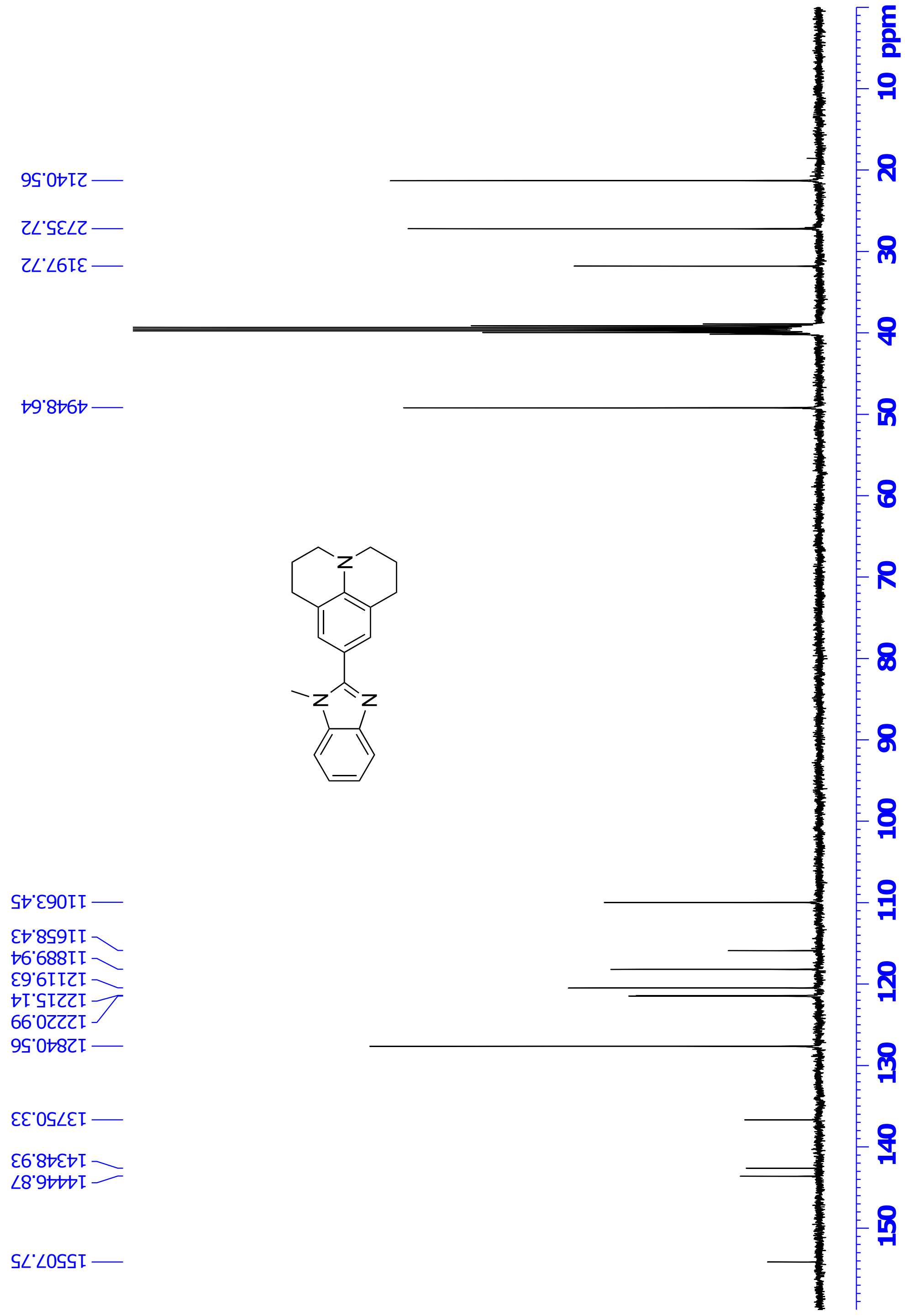

Figure S23: $^{13}C\{^{1}H\}$ NMR of derivative **7f** in DMSO$-d_6$.

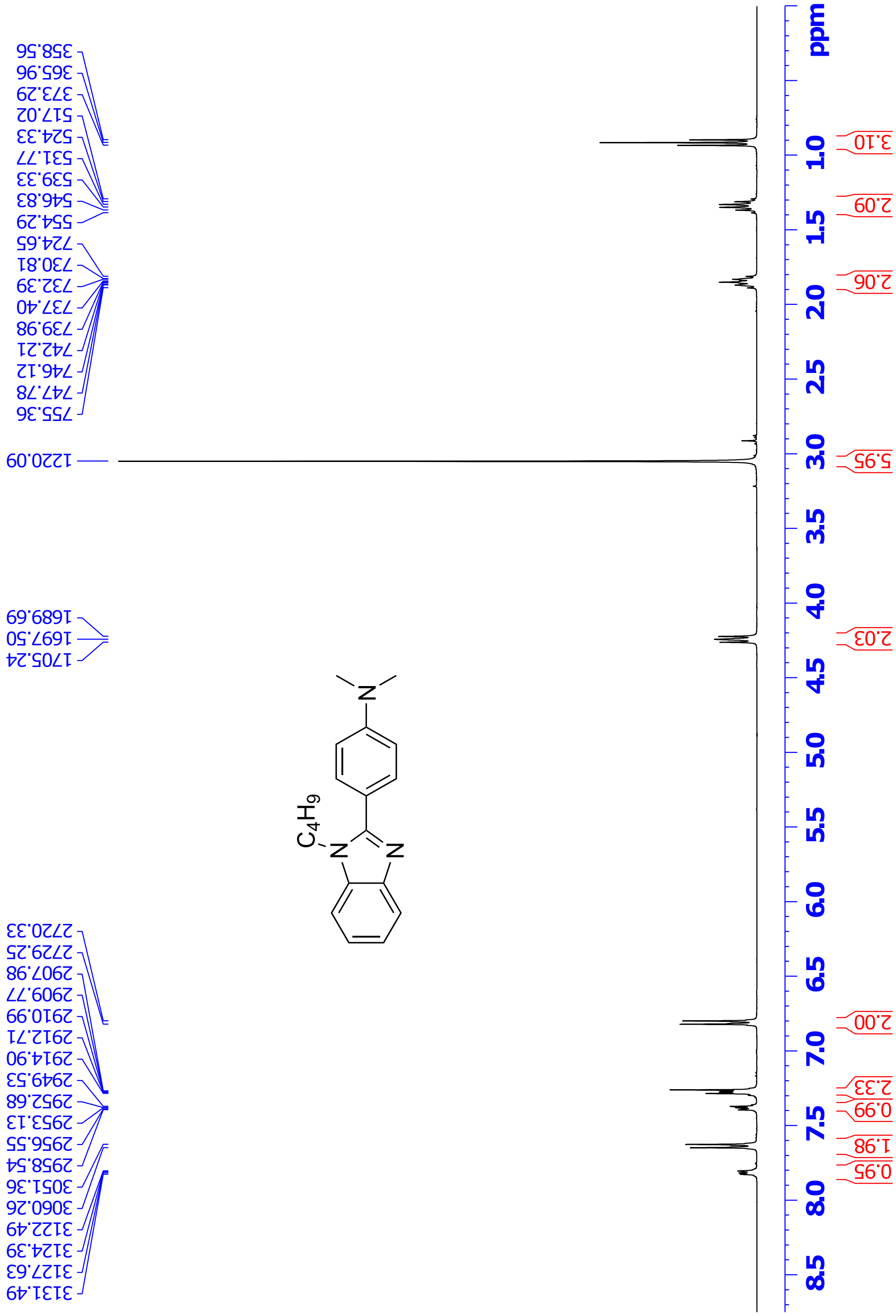

Figure S24: ^{1}H NMR of derivative **7h** in $CDCl_3$.

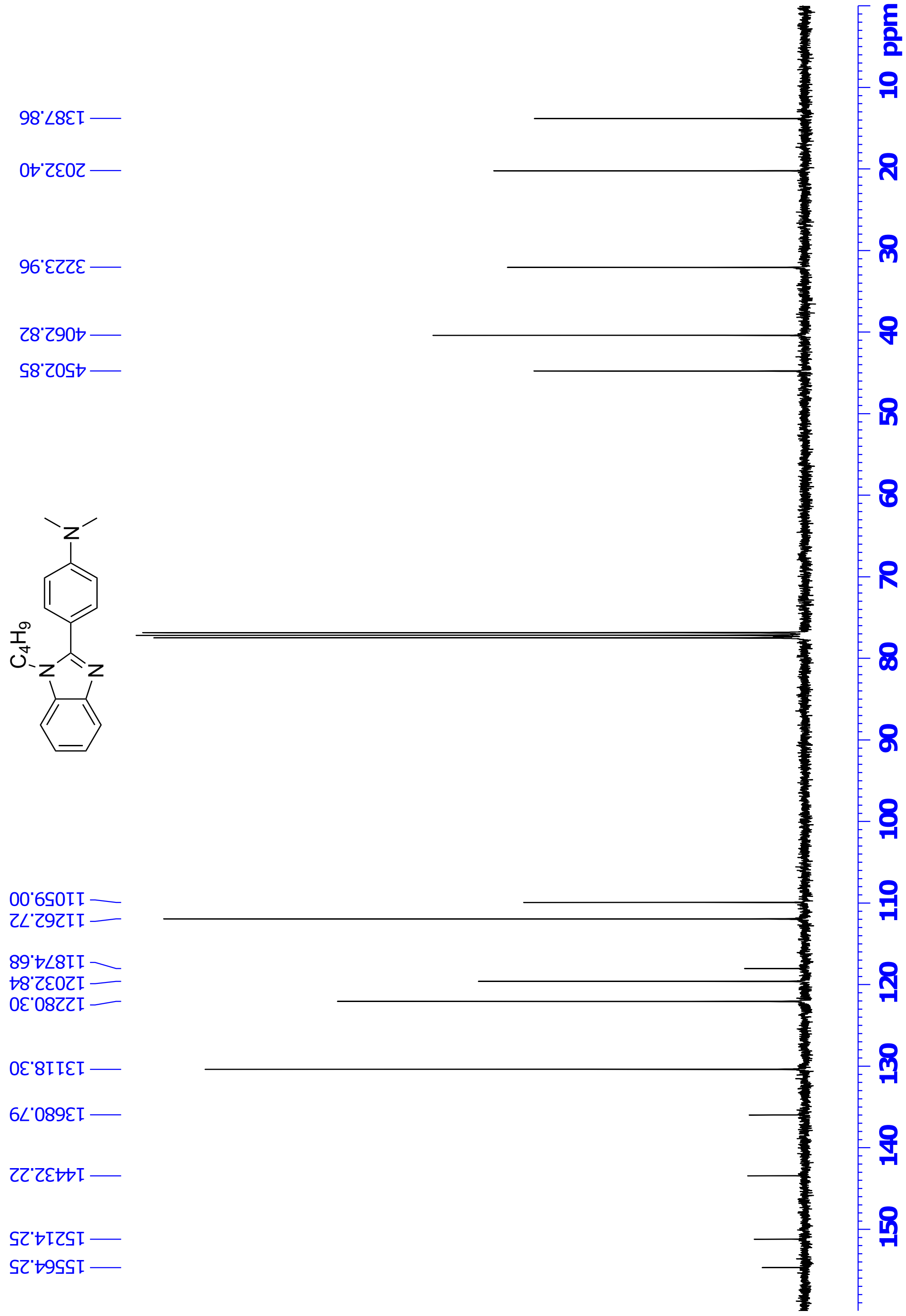

Figure S25: $^{13}C\{^{1}H\}$ NMR of derivative **7h** in $CDCl_3$.

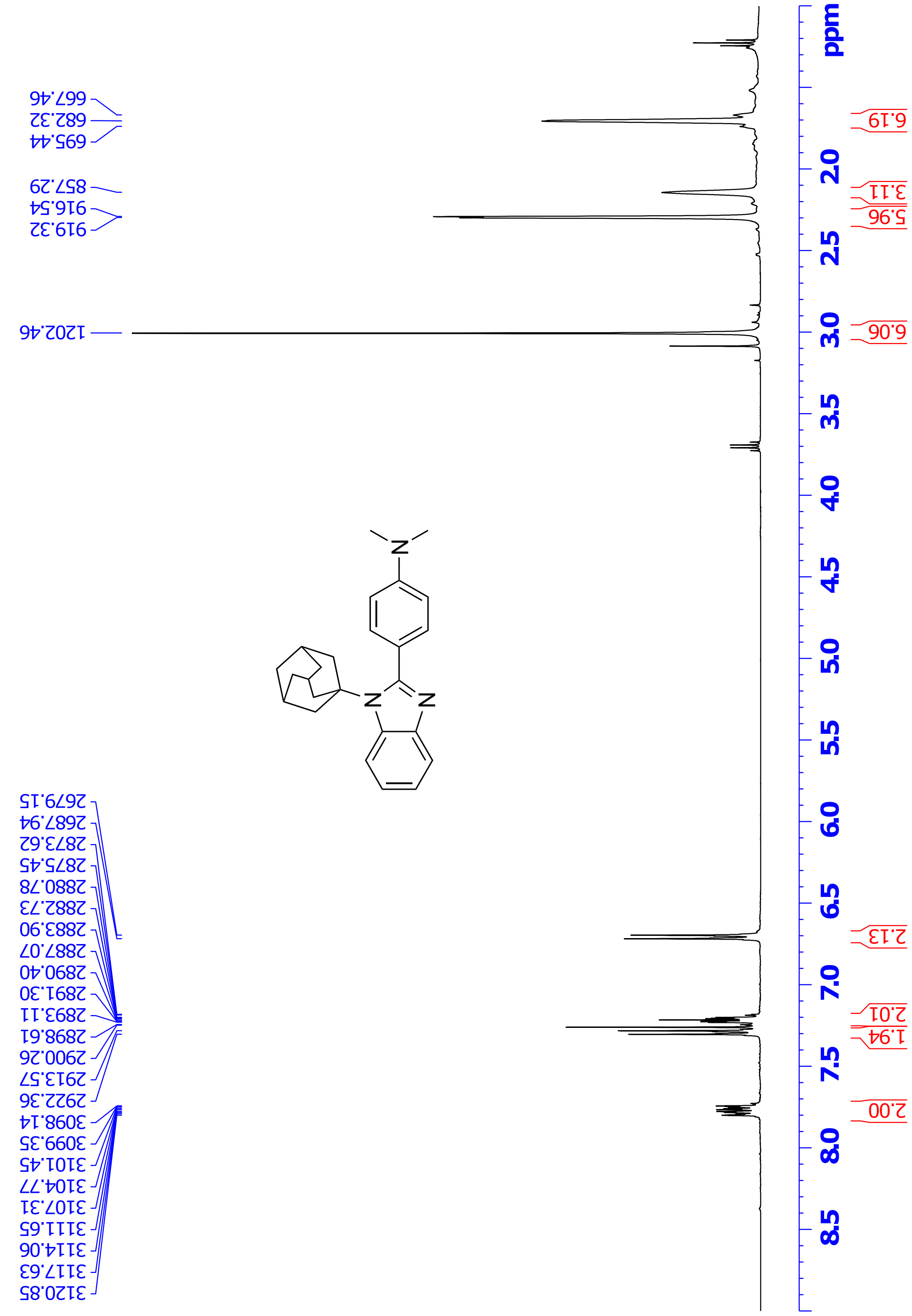

Figure S26: ^{1}H NMR of derivative **7l** in $CDCl_3$. Peaks at 3.70 ppm (q) and 1.23 ppm (t) are associated to residual ethanol traces.

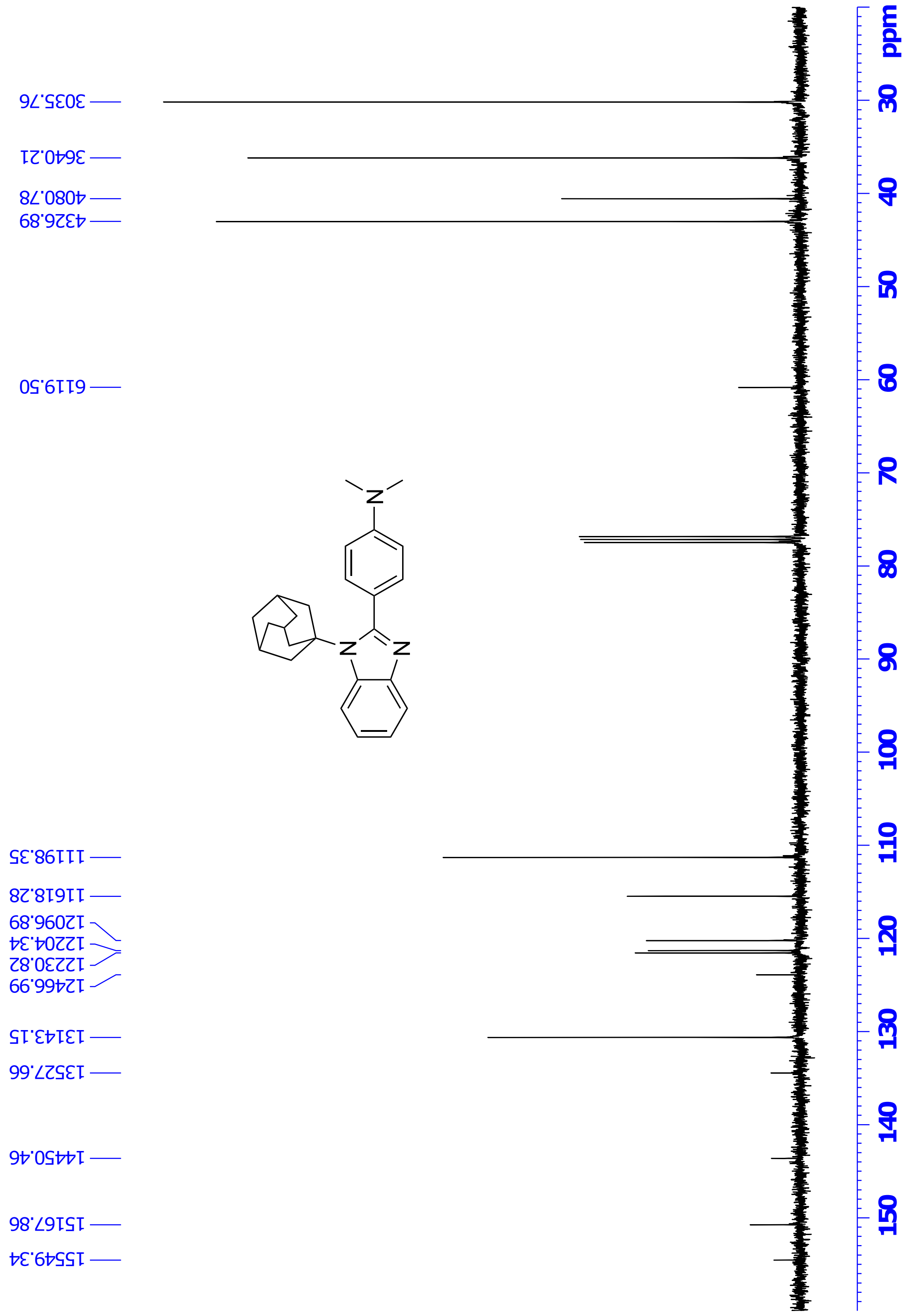

Figure S27: $^{13}C\{^{1}H\}$ NMR of derivative **7l** in $CDCl_3$.

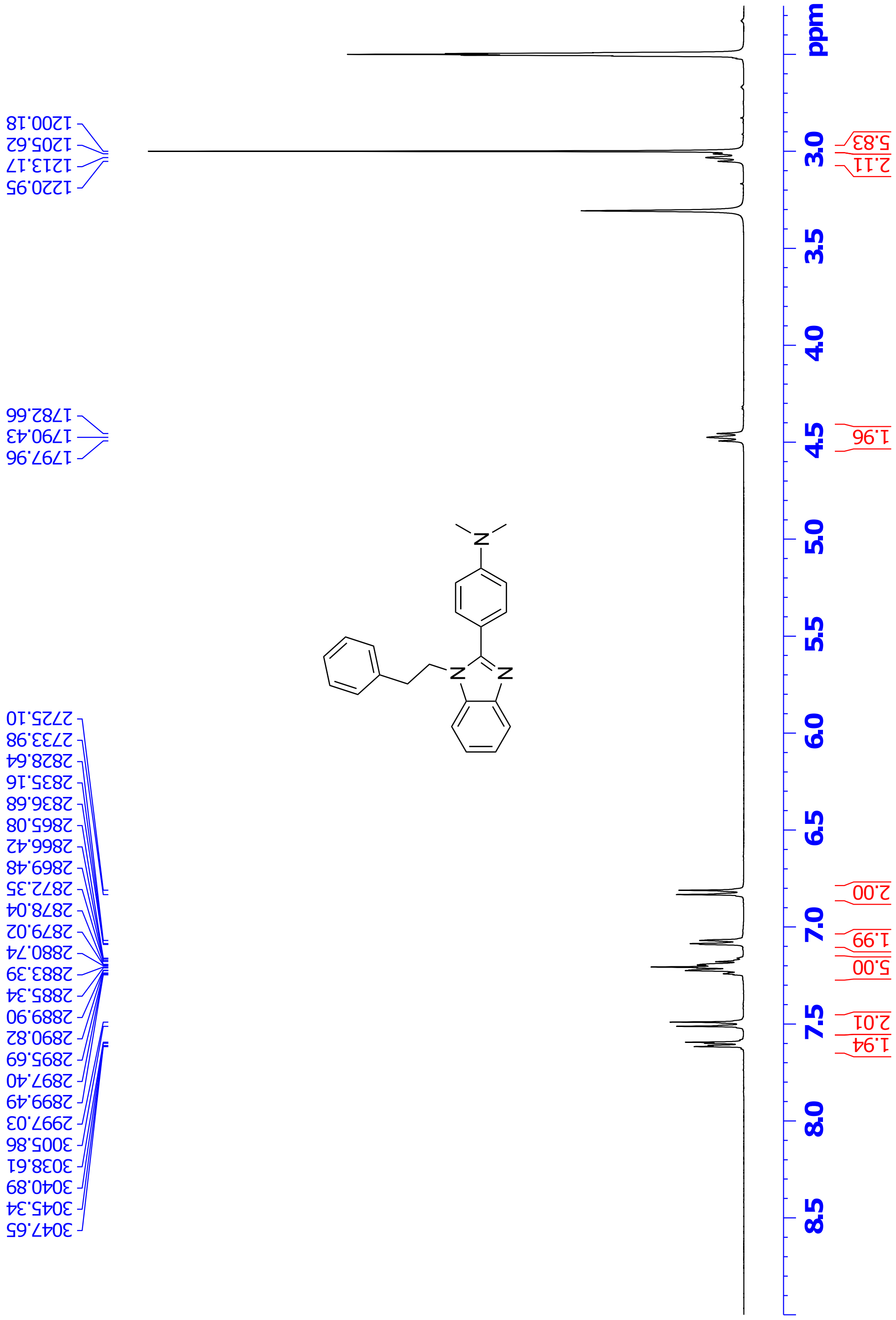

Figure S28: ^{1}H NMR of derivative **7m** in DMSO−d_6.

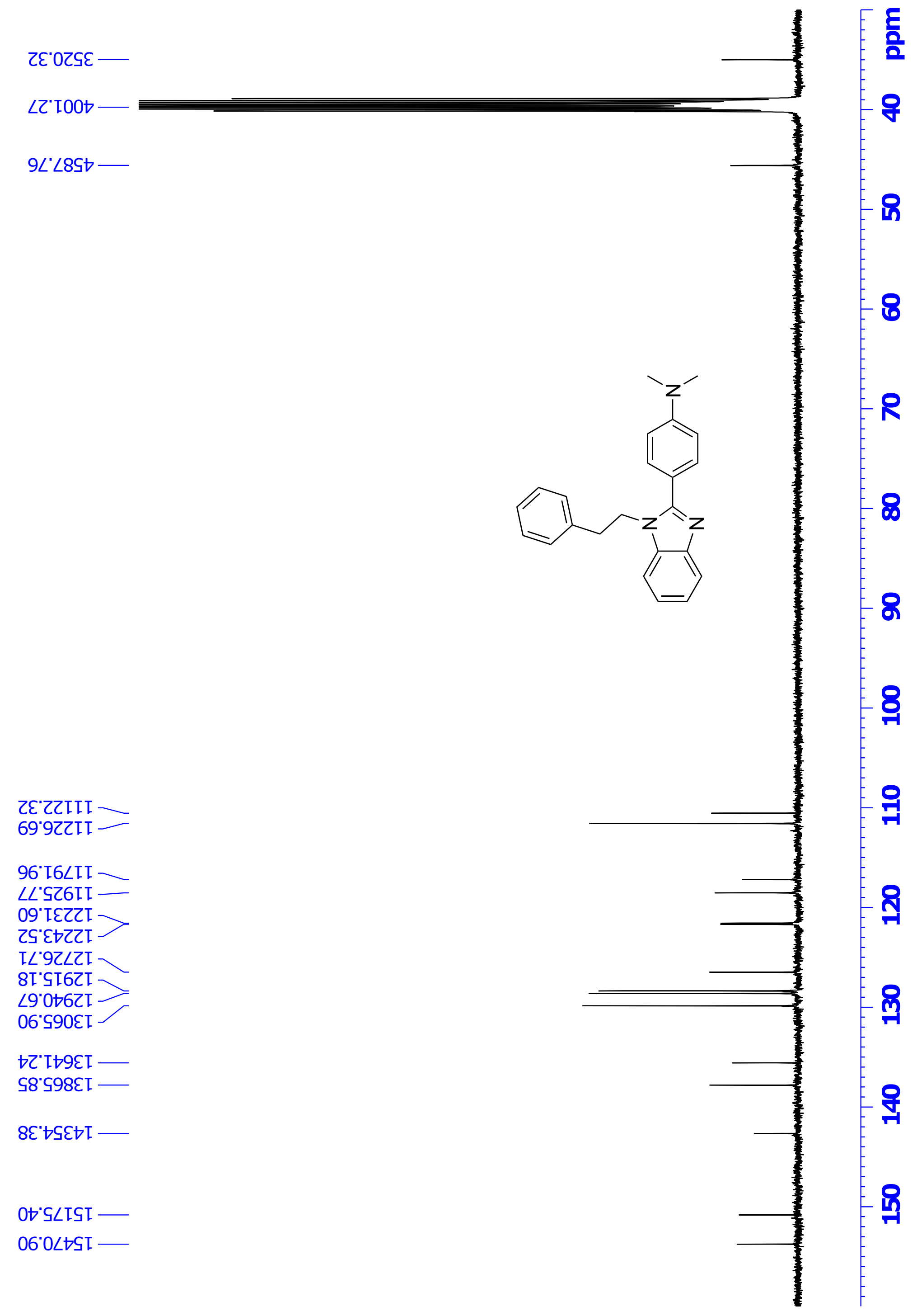

Figure S29: $^{13}C\{^{1}H\}$ NMR of derivative **7m** in DMSO$-d_6$.

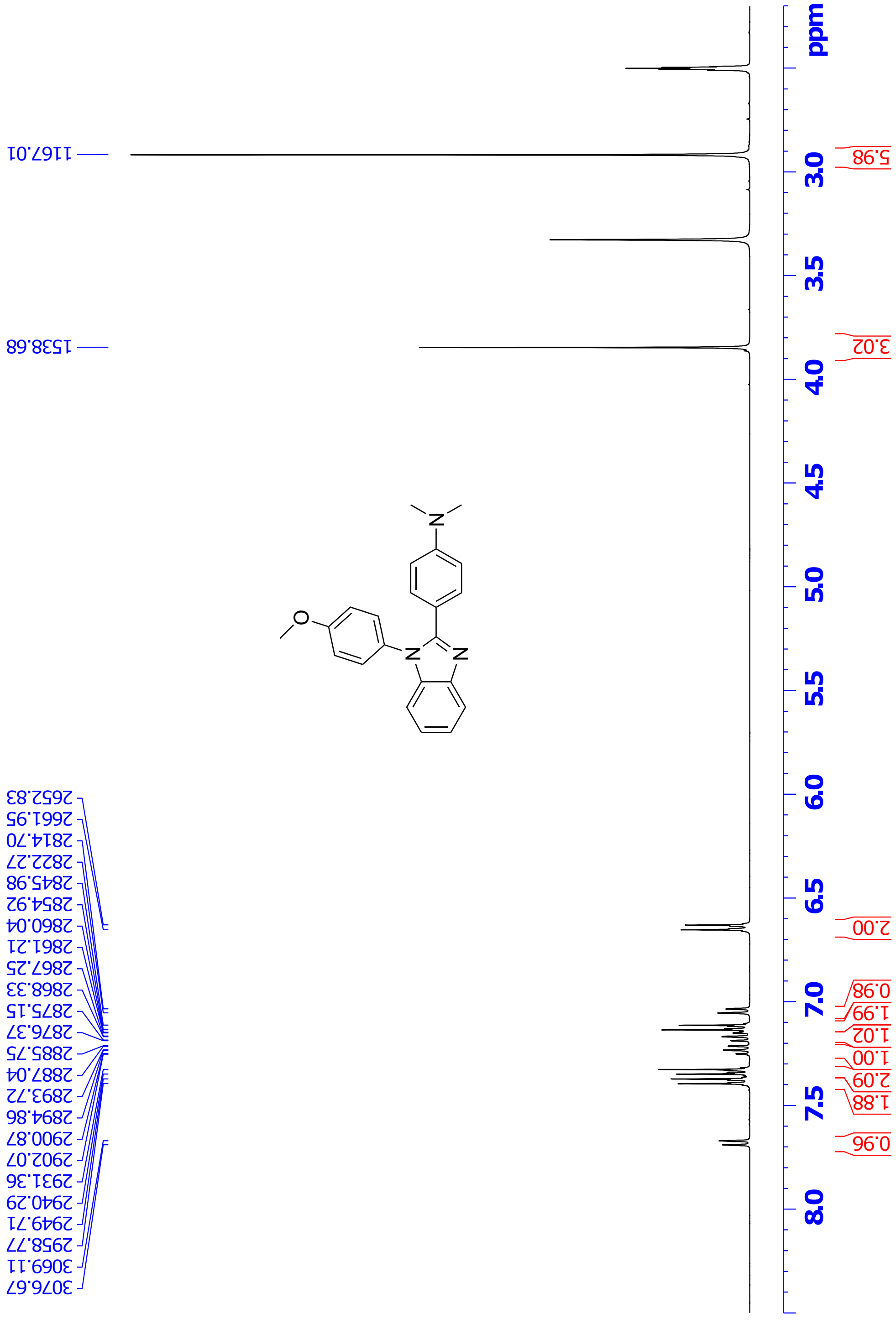

Figure S30: ^{1}H NMR of derivative **7n** in DMSO$-$d$_6$.

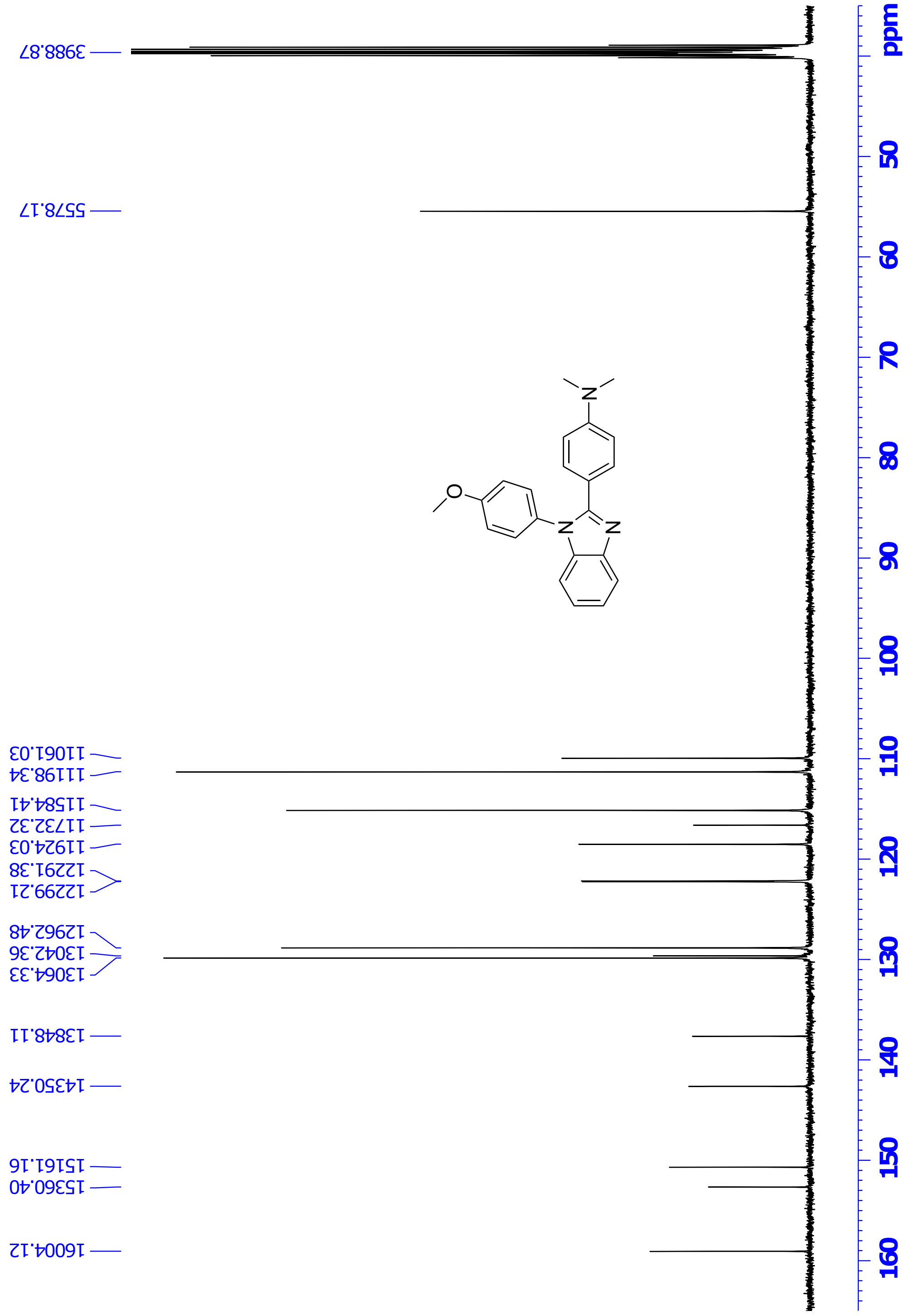

Figure S31: $^{13}C\{^1H\}$ NMR of derivative **7n** in DMSO−d_6.

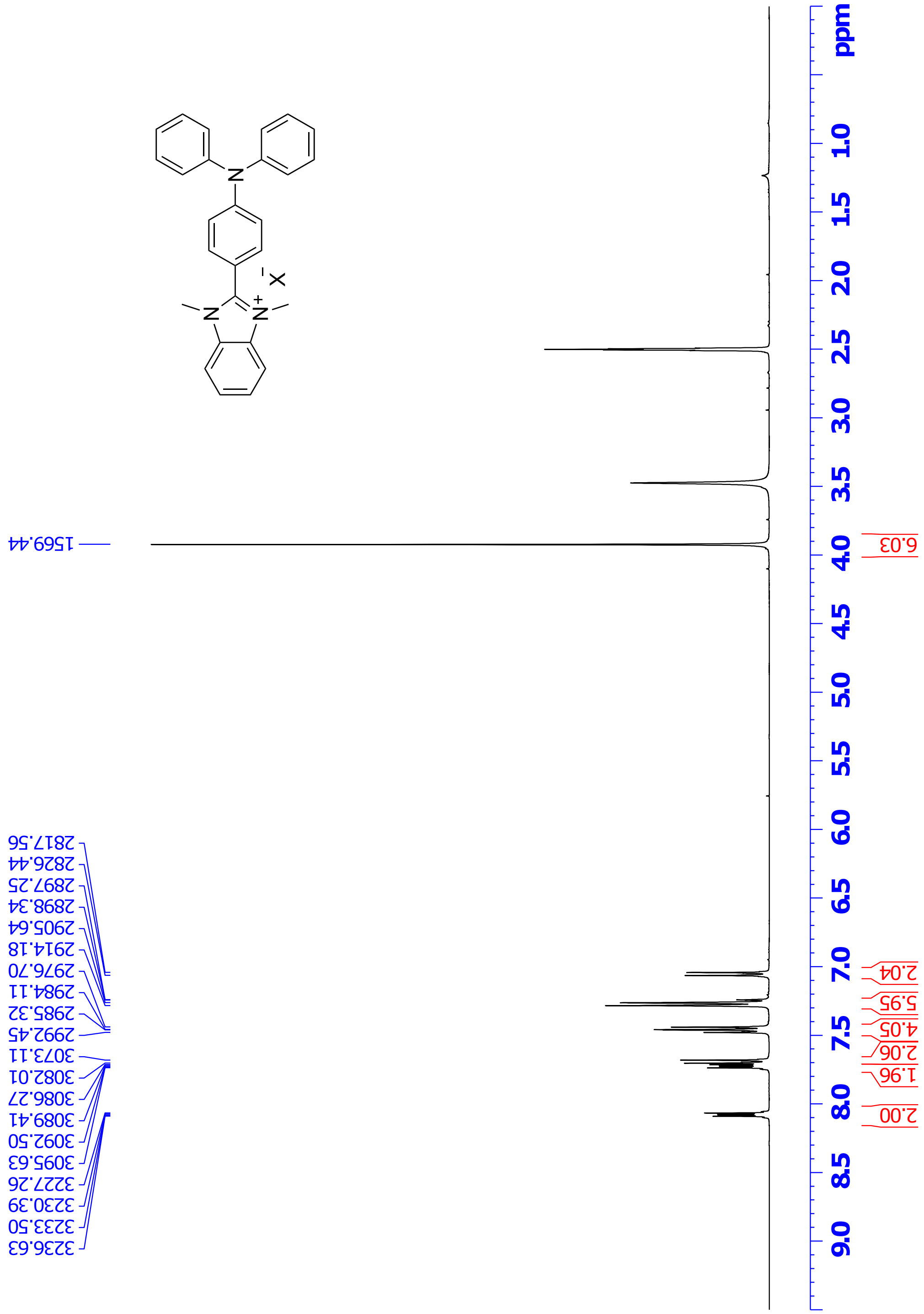

Figure S32: ^{1}H NMR of derivative **8c** in DMSO$-d_6$.

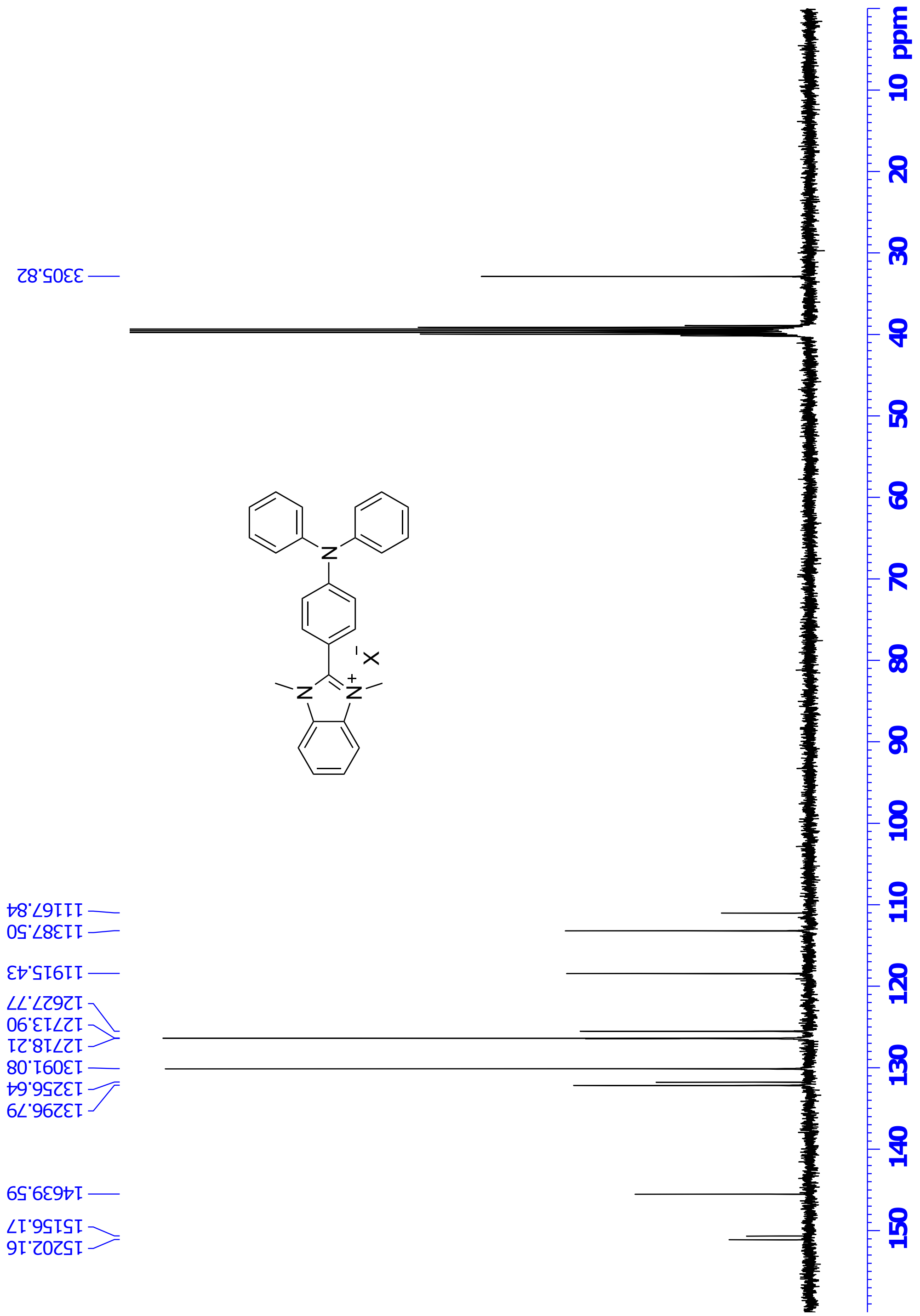

Figure S33: $^{13}C\{^{1}H\}$ NMR of derivative **8c** in DMSO$-d_6$.

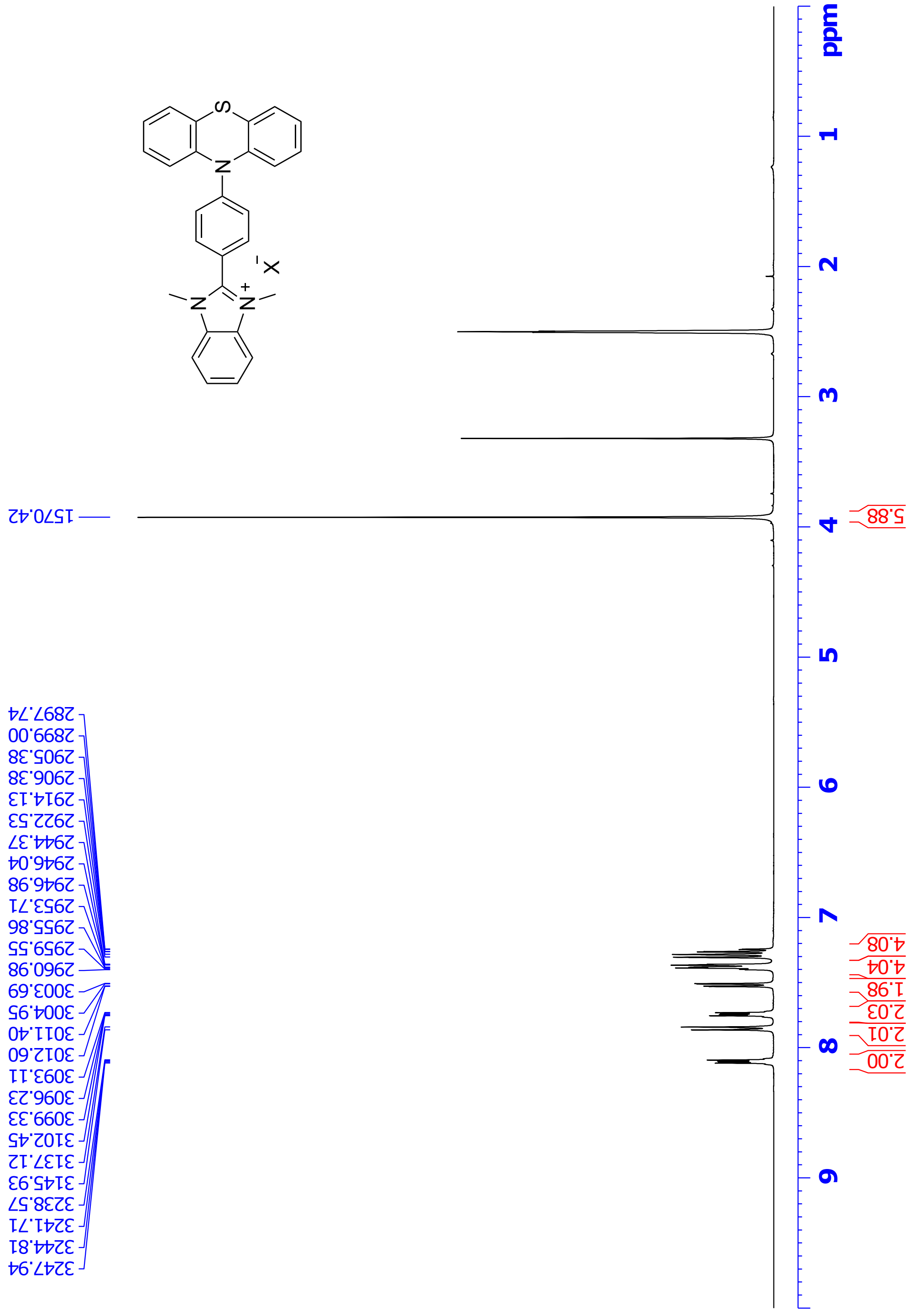

Figure S34: ^{1}H NMR of derivative **8d** in DMSO−d_6.

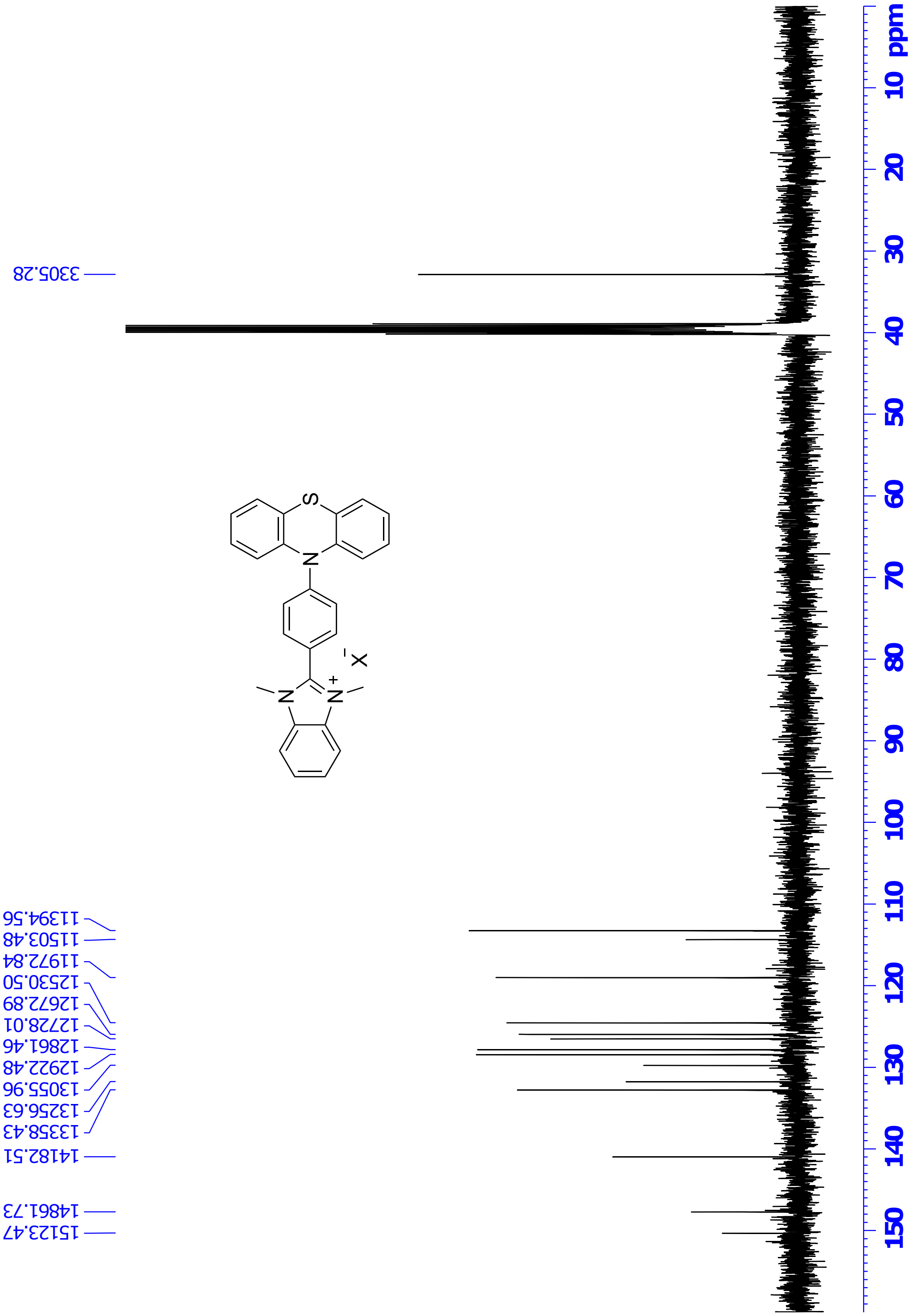

Figure S35: $^{13}C\{^1H\}$ NMR of derivative **8d** in DMSO$-d_6$.

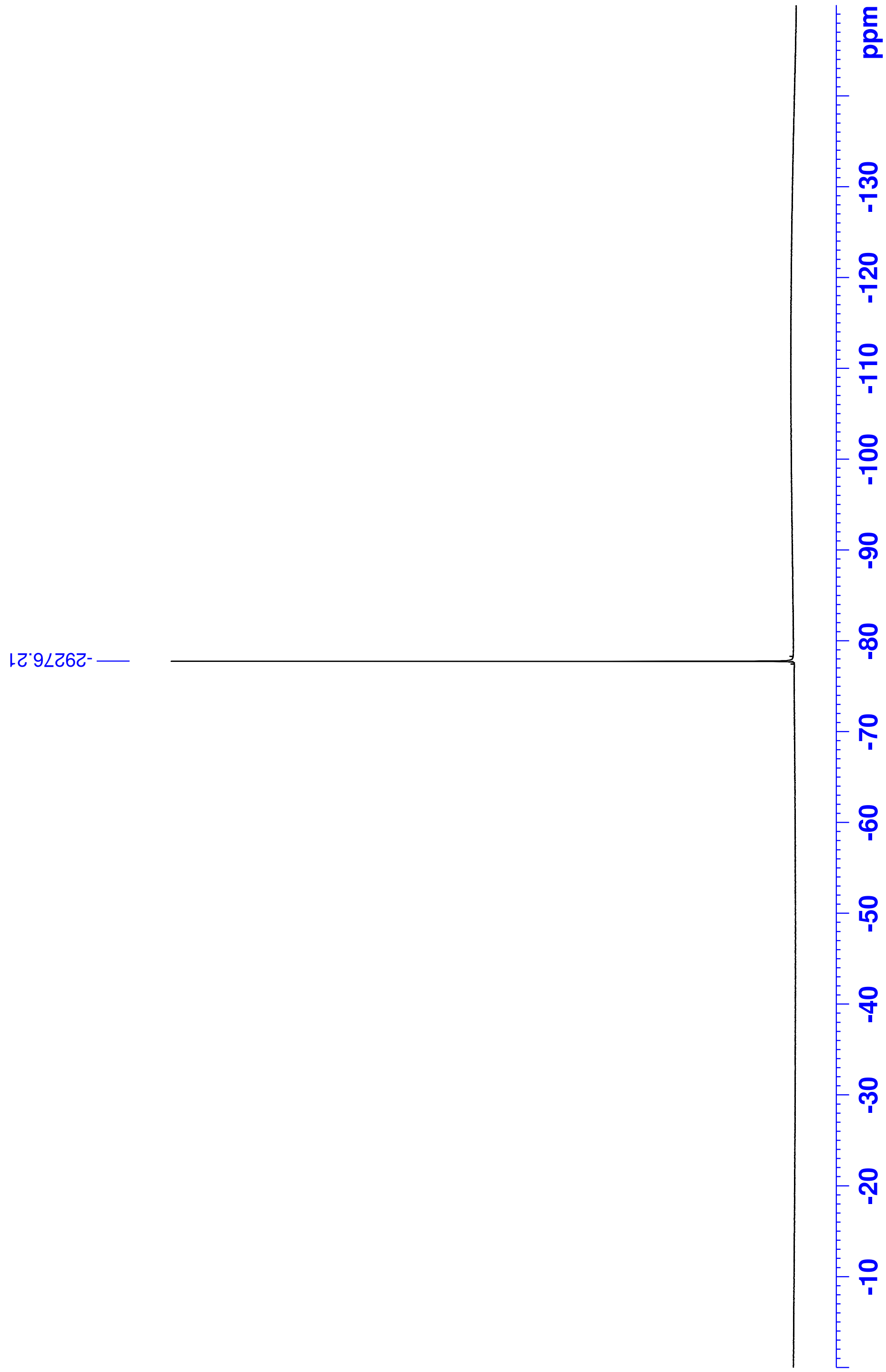

Figure S36: ^{19}F NMR of derivative **8d** as triflate salt in DMSO$-d_6$.

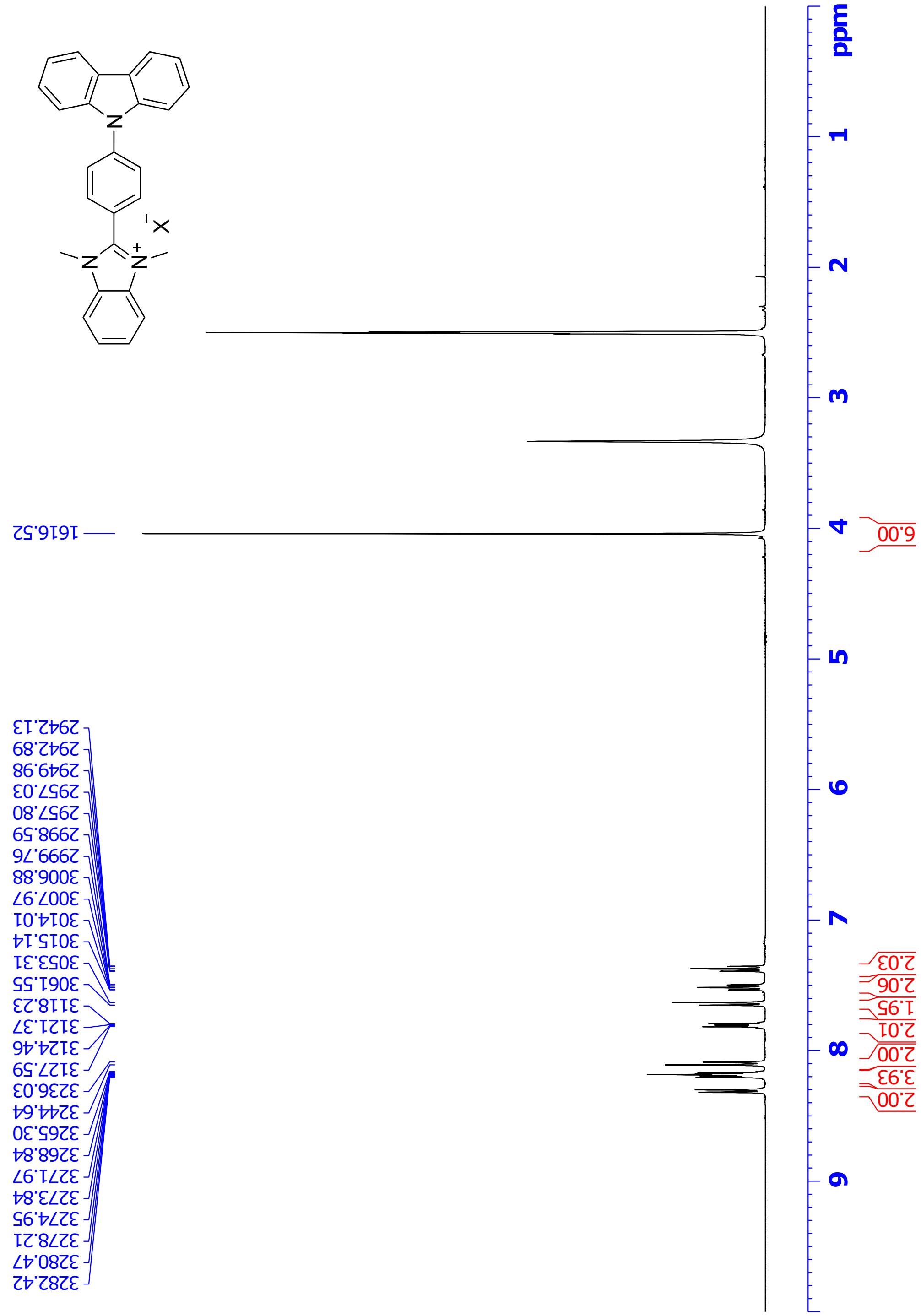

Figure S37: ^{1}H NMR of derivative **8e** in DMSO$-d_6$.

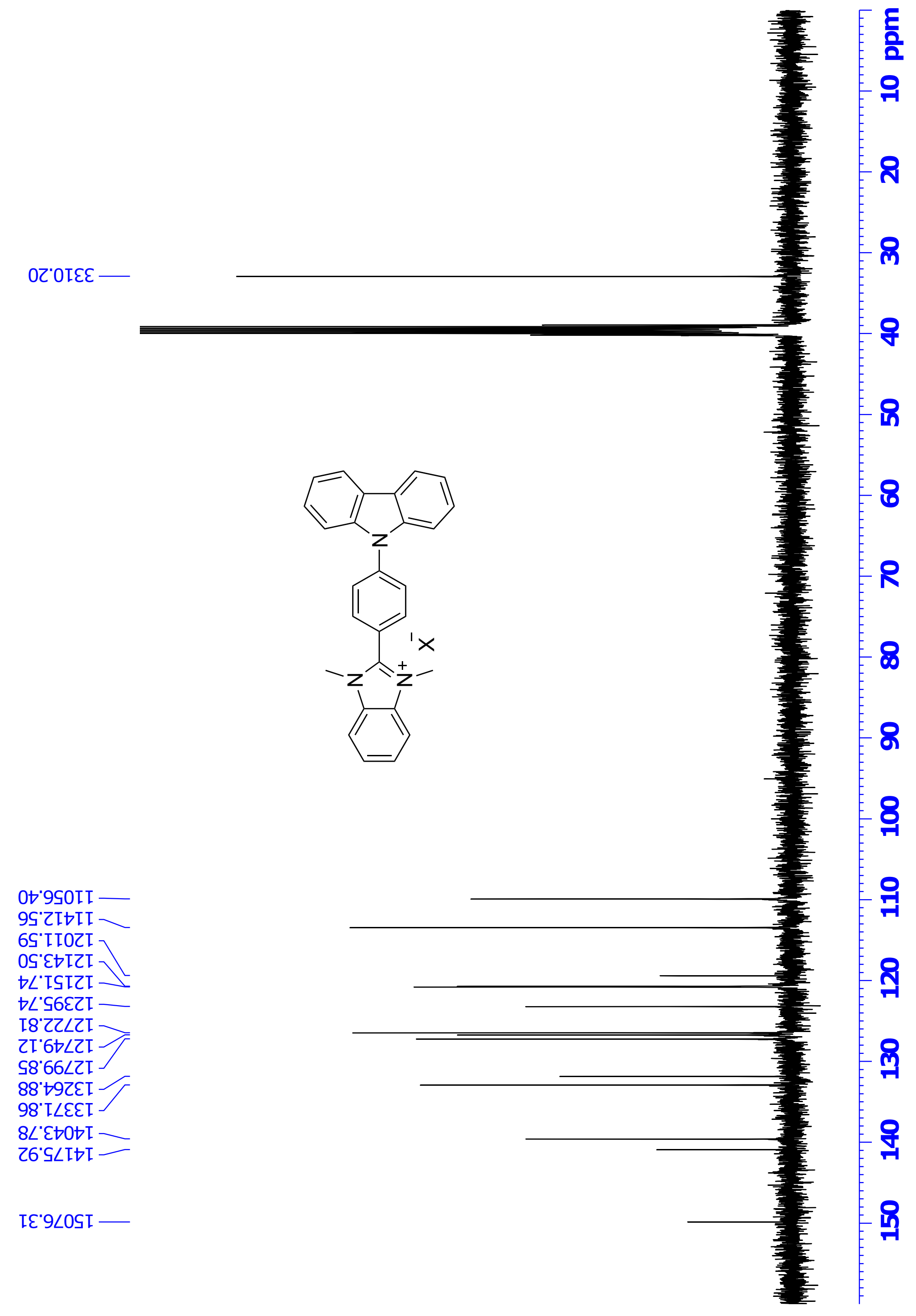

Figure S38: $^{13}C\{^{1}H\}$ NMR of derivative **8e** in DMSO$-d_6$.

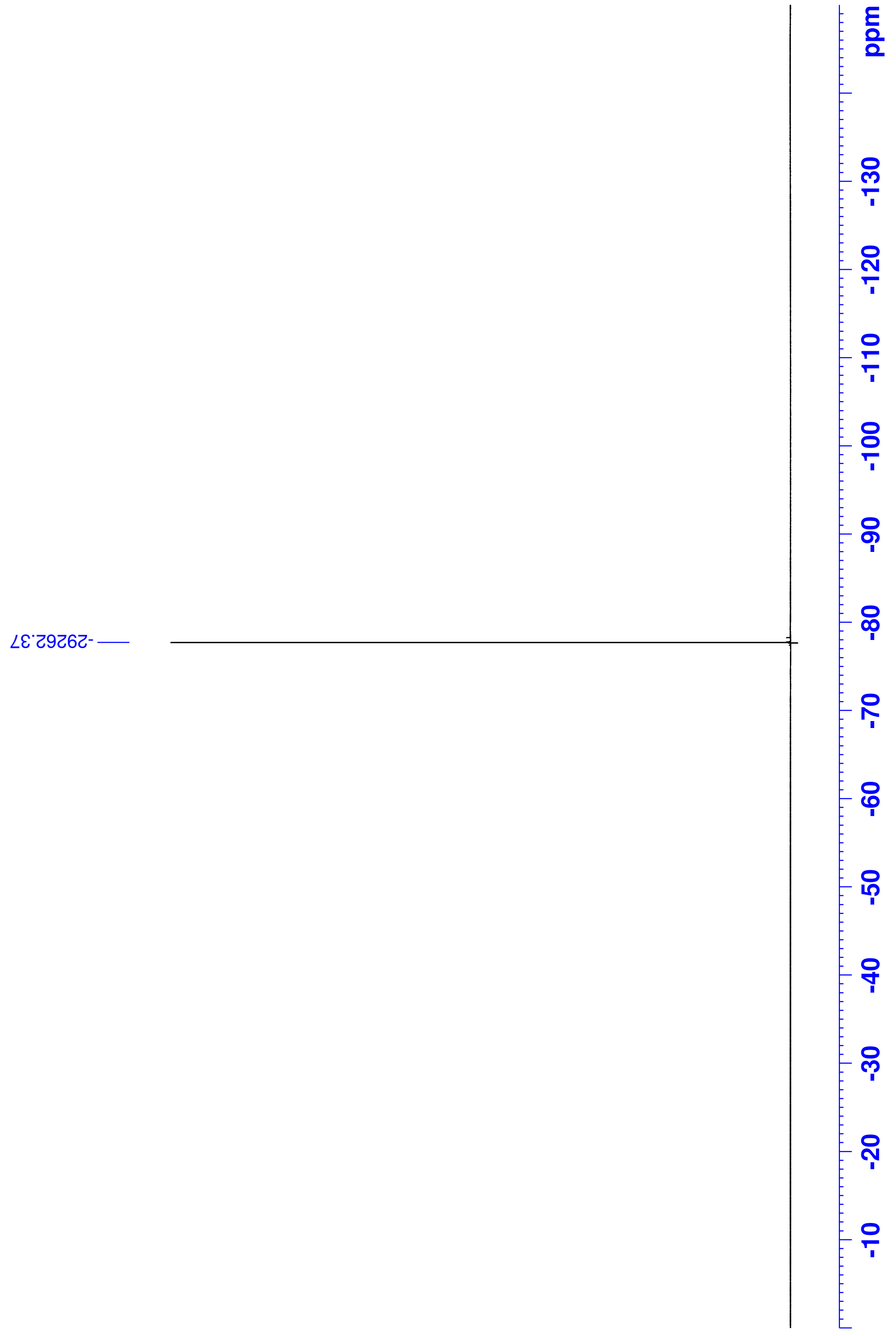

Figure S39: ^{19}F NMR of derivative **8e** as triflate salt in DMSO$-$d$_6$.

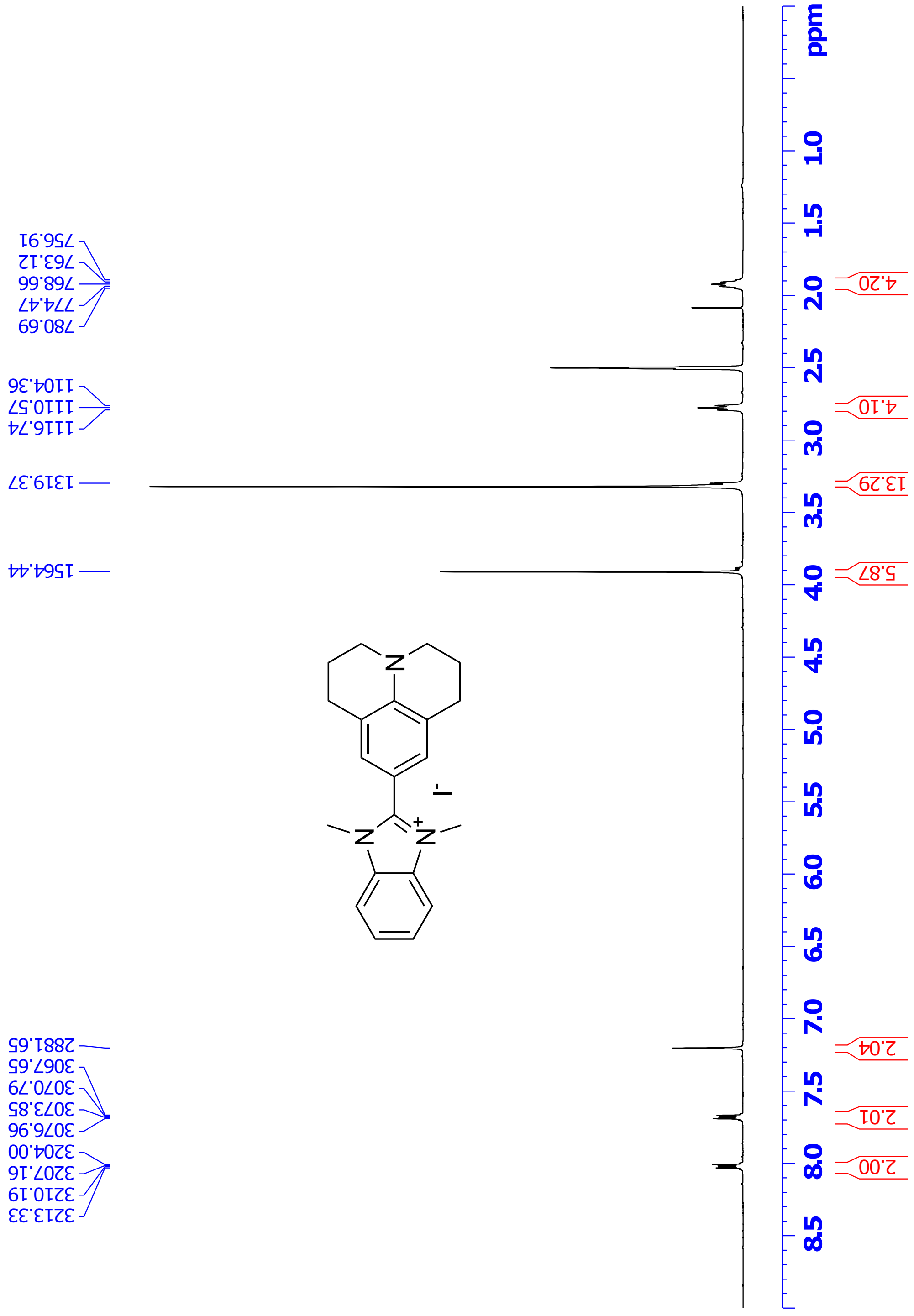

Figure S40: ^{1}H NMR of derivative **8f** as iodide salt in DMSO$-$d$_6$. Integral of multiplet located at 3.31 ppm results overabundant due to superimposition with peak of water present in the deuterated solvent.

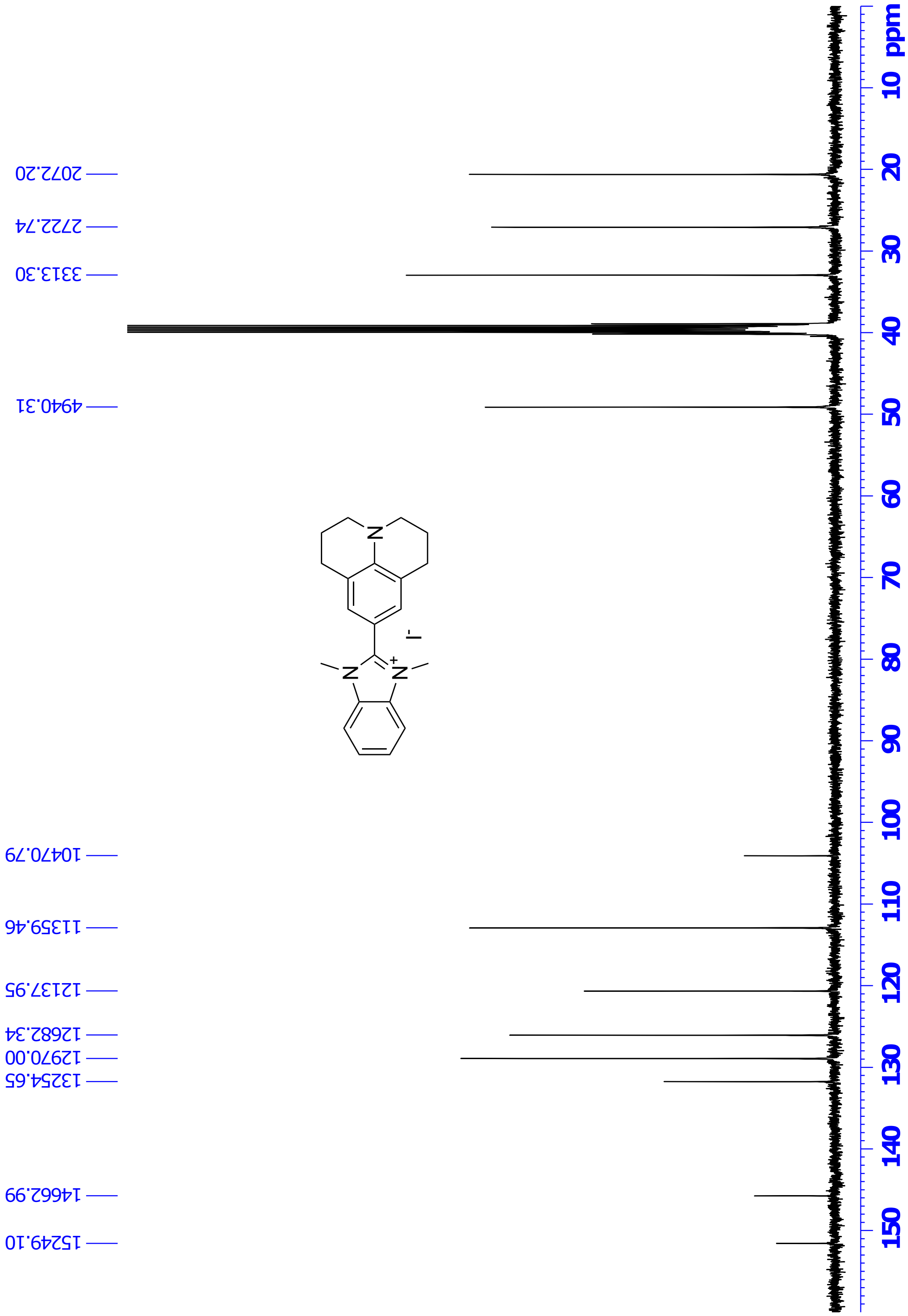

Figure S41: $^{13}C\{^{1}H\}$ NMR of derivative **8f** as iodide salt in DMSO$-d_6$.

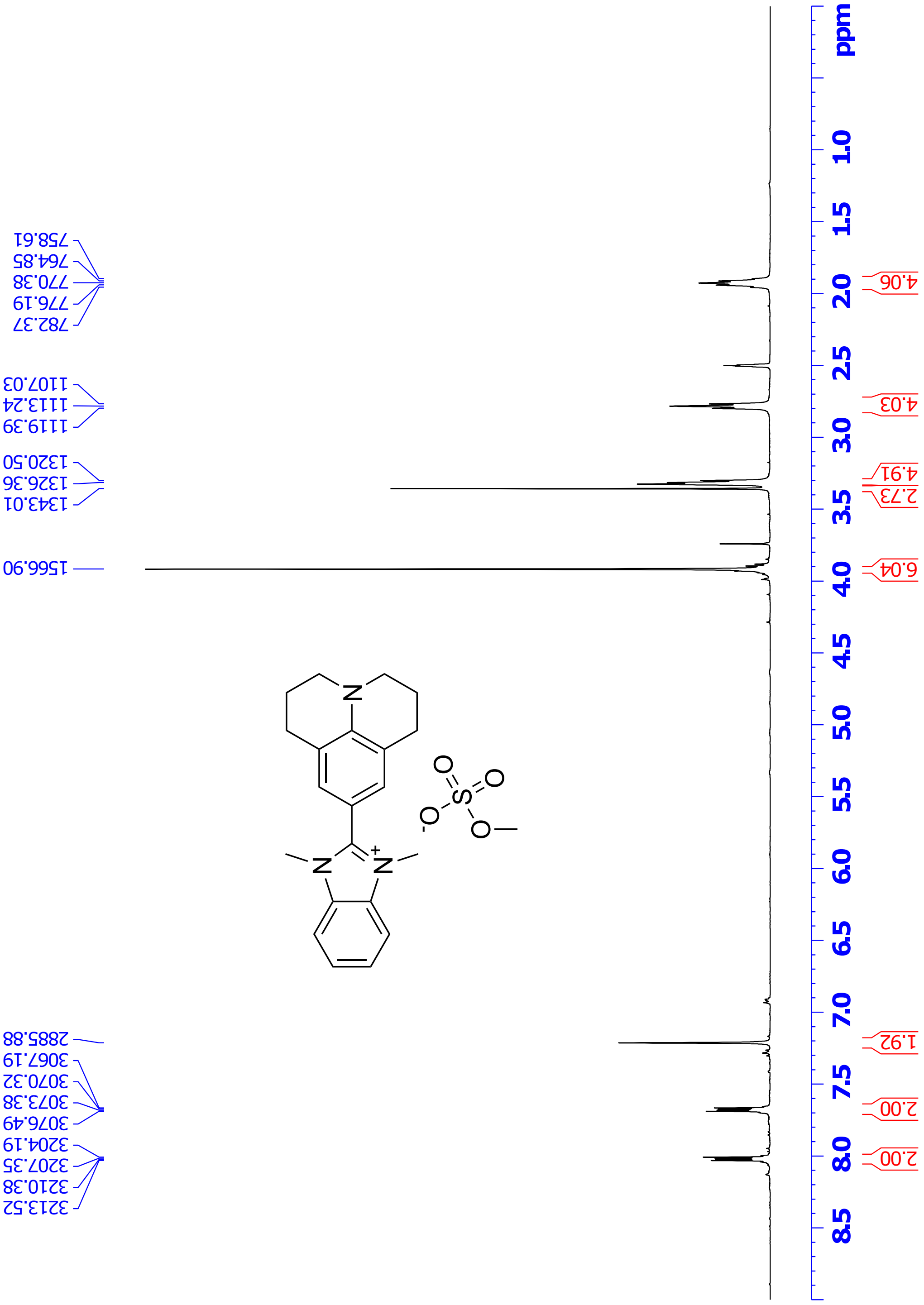

Figure S42: ^{1}H NMR of derivative **8f** as methyl sulfate salt in DMSO$-d_6$. Peaks located at 7.28 (m), 6.93-6.90 (m) and 3.74 (s) ppm are associated to residual anisole.

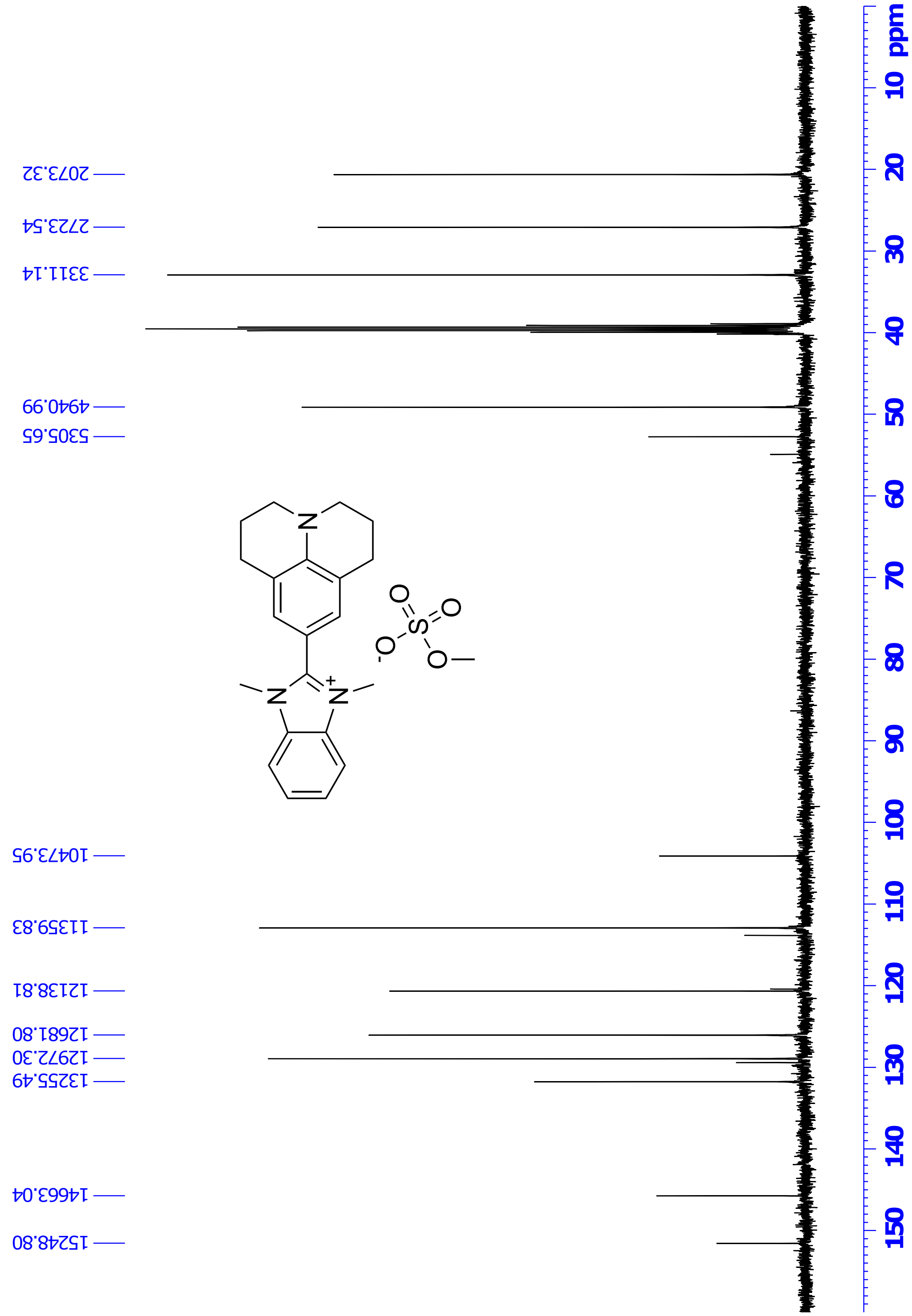

Figure S43: $^{13}C\{^{1}H\}$ NMR of derivative **8f** as methyl sulfate salt in DMSO$-d_6$. Peaks located at 54.89, 113.85, 120.44 and 129.44 ppm are associated to residual anisole.

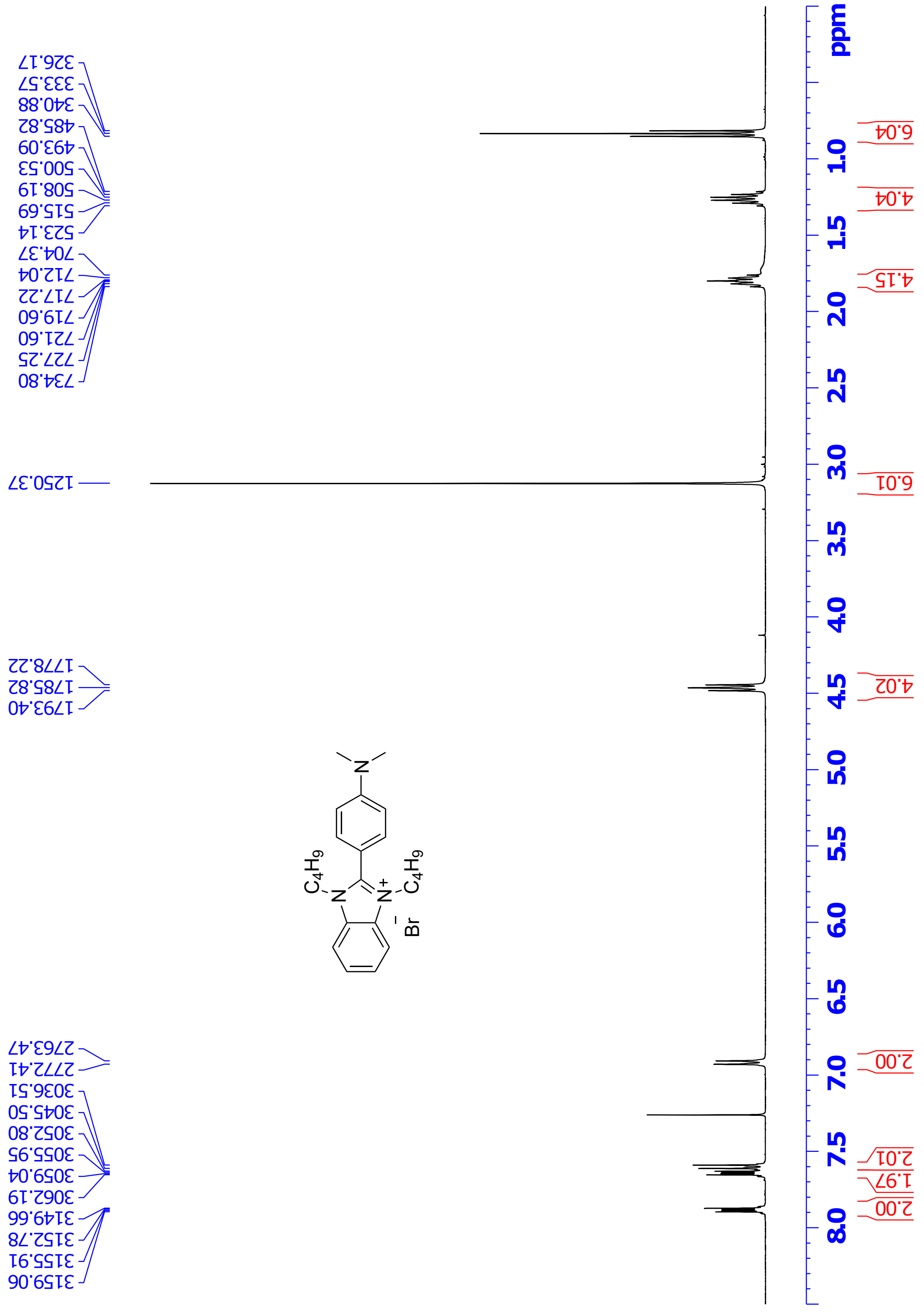

Figure S44: ^{1}H NMR of derivative **8h** in $CDCl_3$.

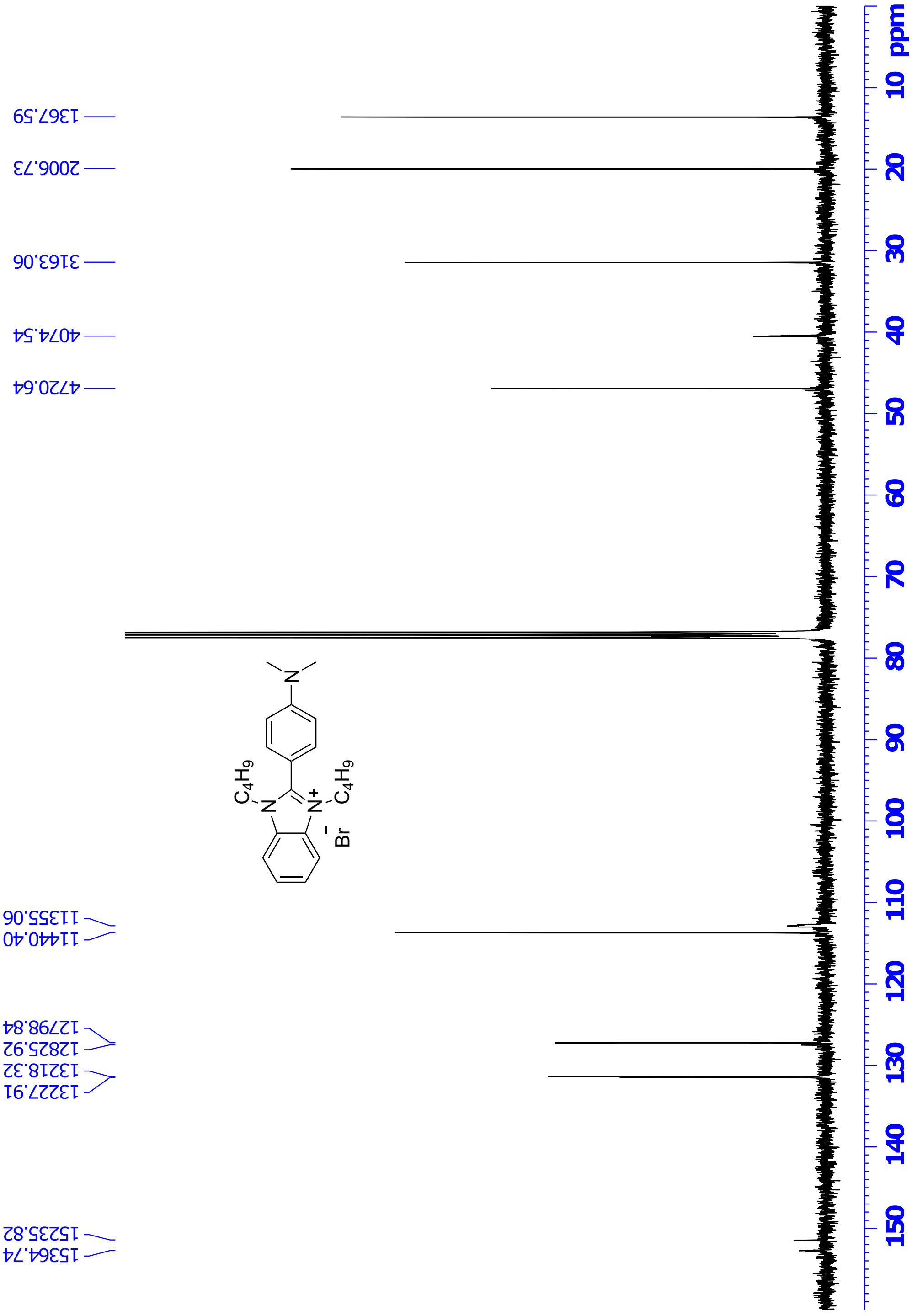

Figure S45: $^{13}C\{^{1}H\}$ NMR of derivative **8h** in $CDCl_3$.

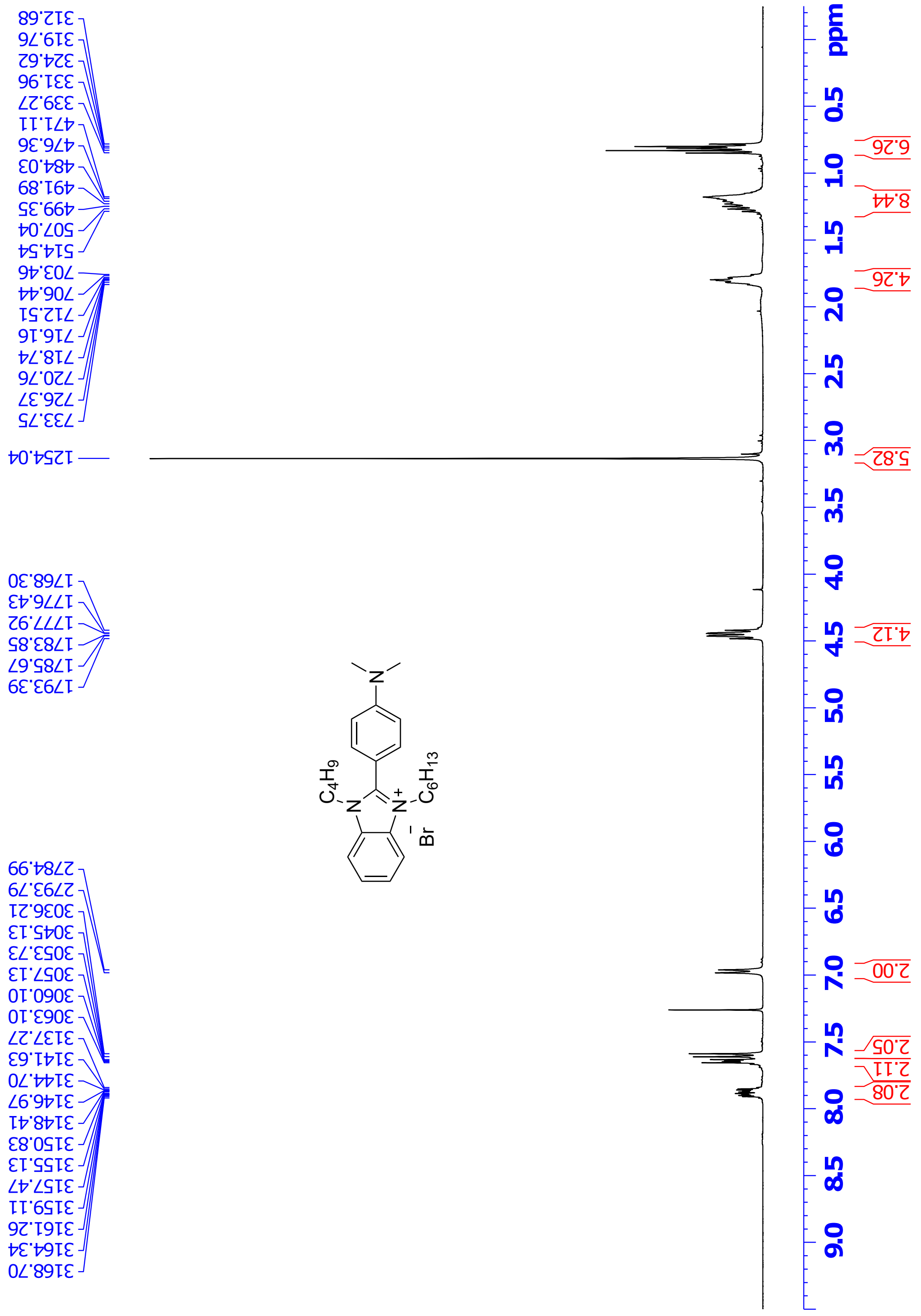

Figure S46: ^{1}H NMR of derivative **8i** in $CDCl_3$.

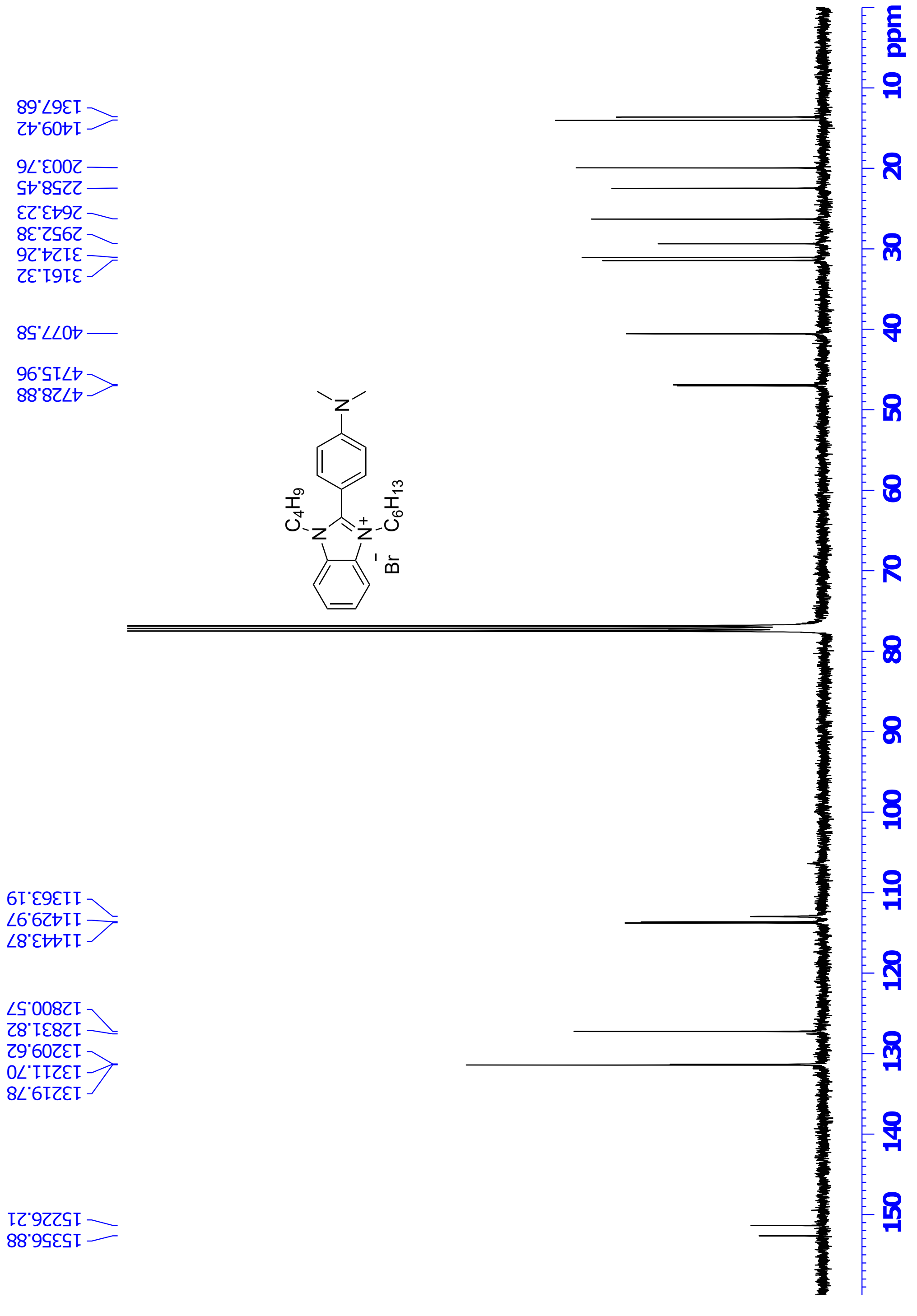

Figure S47: $^{13}C\{^1H\}$ NMR of derivative **8i** in $CDCl_3$.

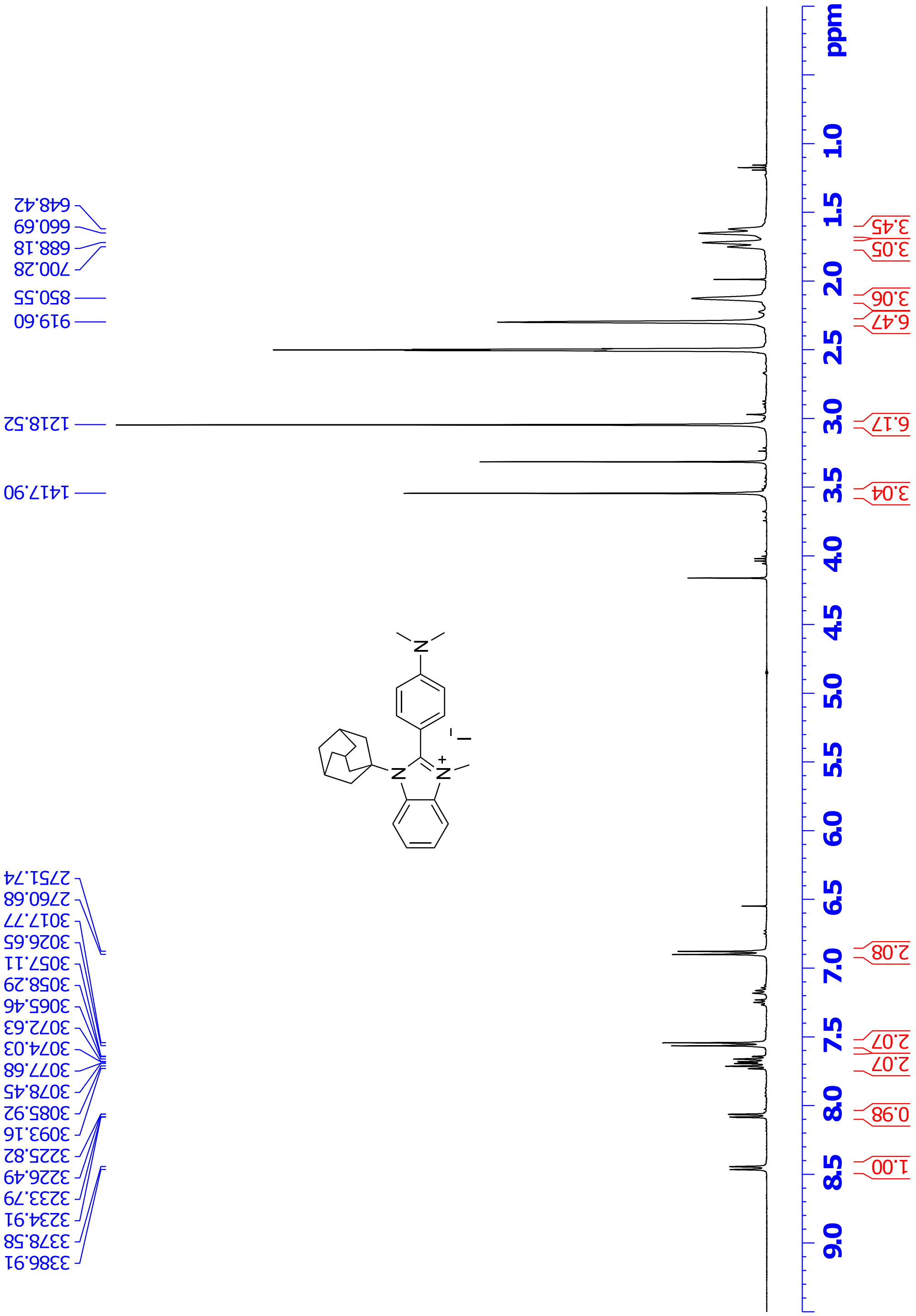

Figure S48: ^{1}H NMR of derivative **8l** in DMSO$-d_6$. Peaks associated to residual ethyl acetate (1.17, 1.99, 4.03 ppm) and toluene (7.18, 7.25 ppm) are visible in the spectrum.

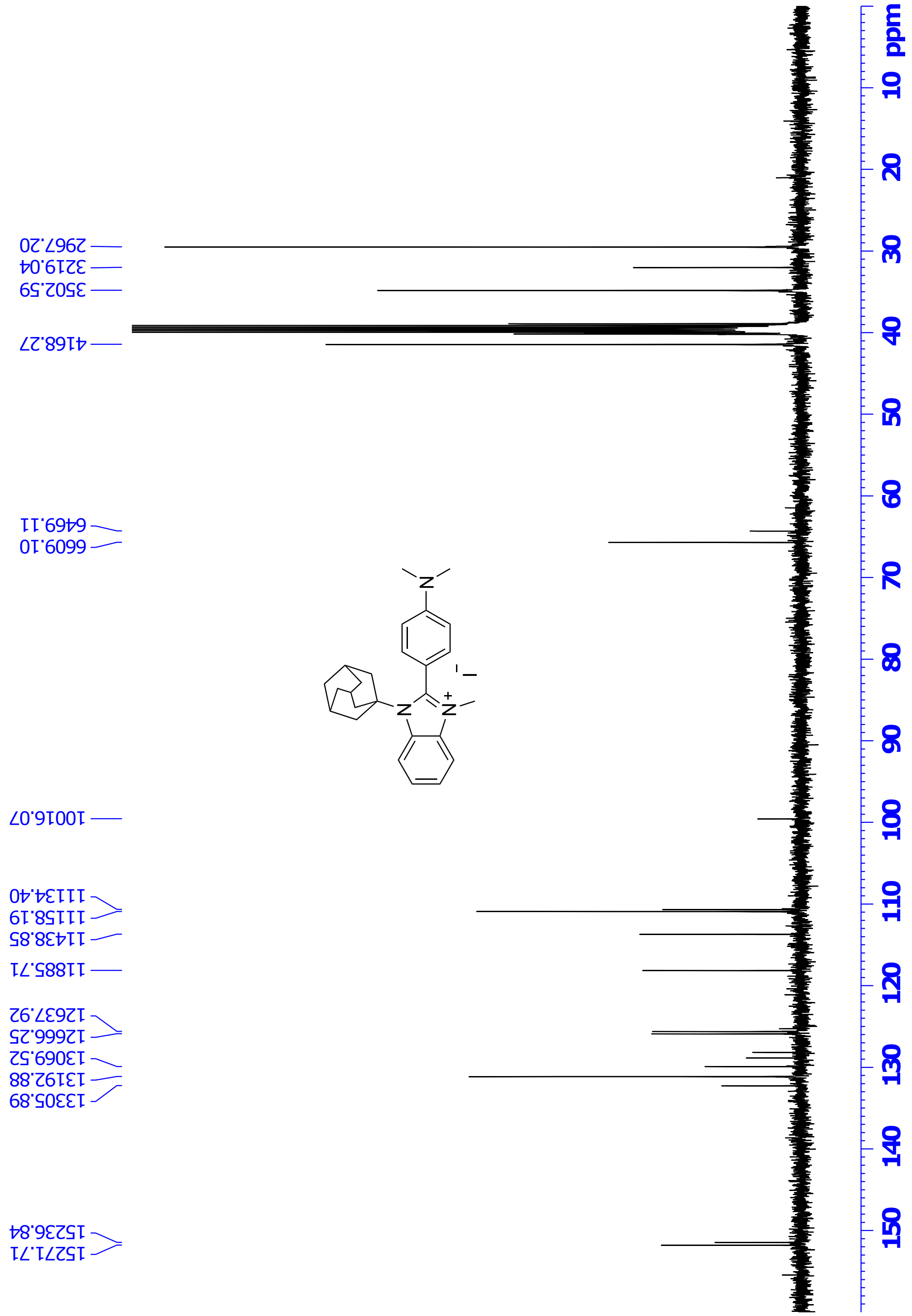

Figure S49: $^{13}C\{^{1}H\}$ NMR of derivative **8l** in DMSO−d_6. Peaks associated to residual toluene (128.88, 128.18 ppm) are visible in the spectrum.

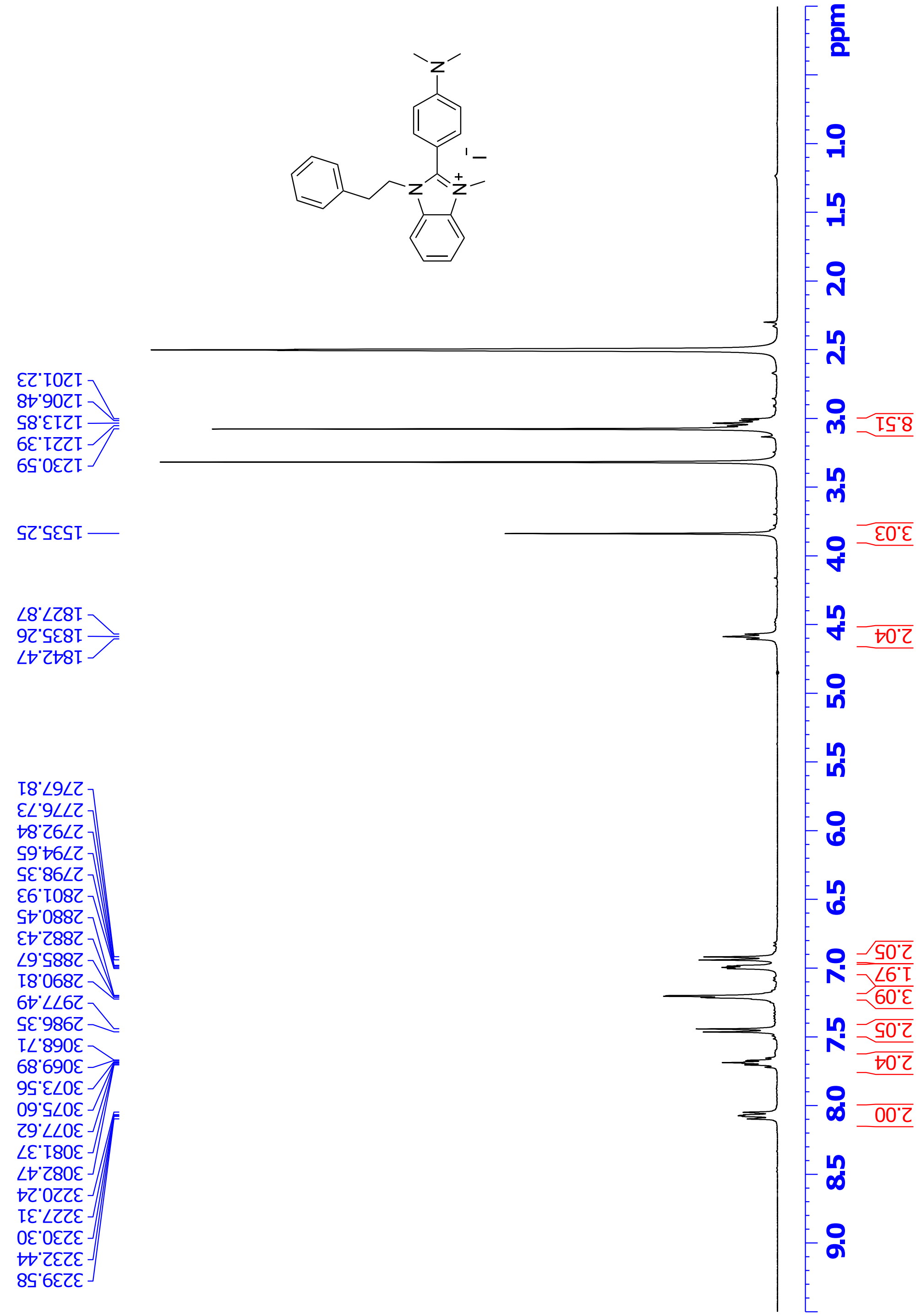

Figure S50: ^{1}H NMR of derivative **8m** in DMSO$-$d$_6$.

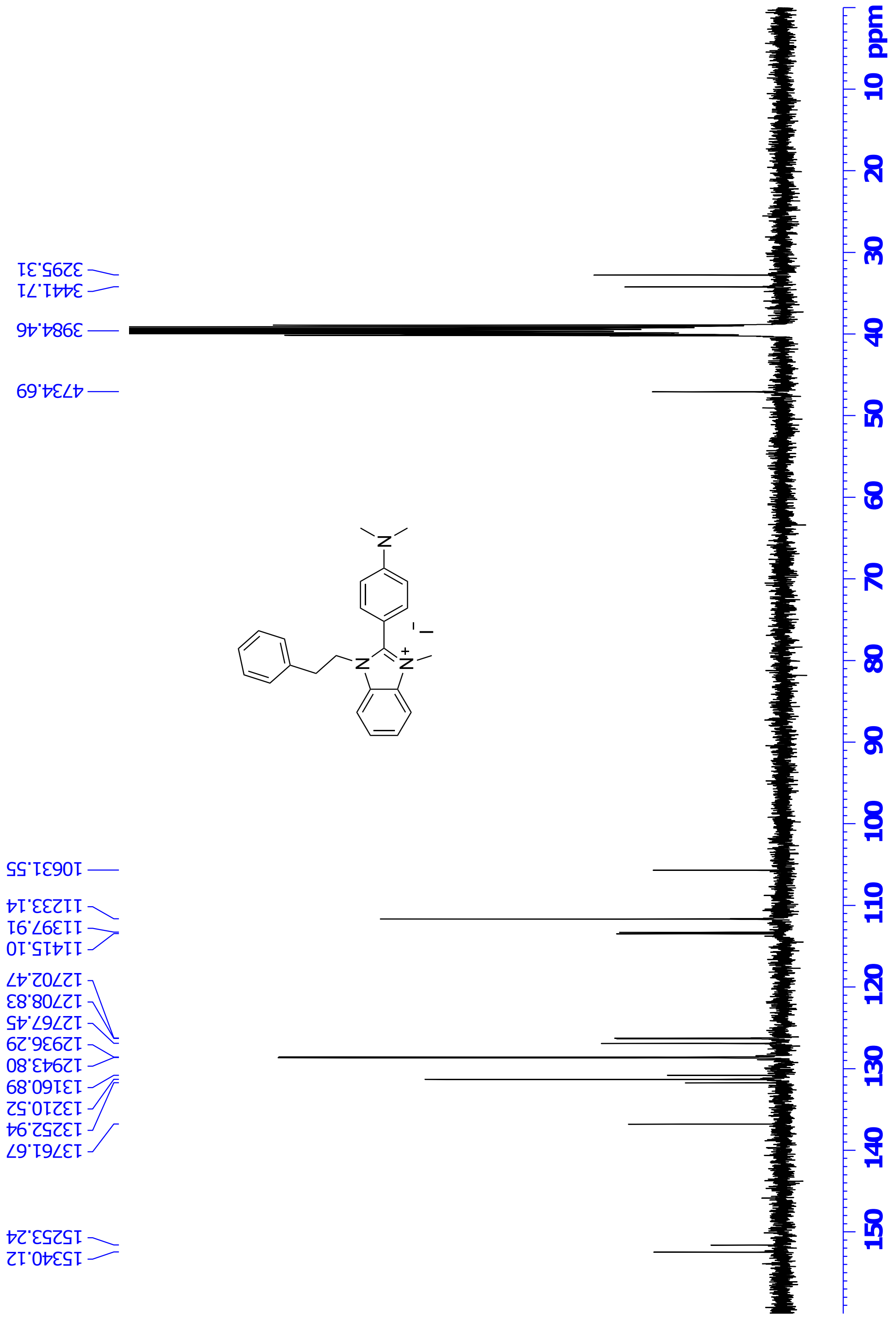

Figure S51: $^{13}C\{^{1}H\}$ NMR of derivative **8m** in DMSO$-d_6$.

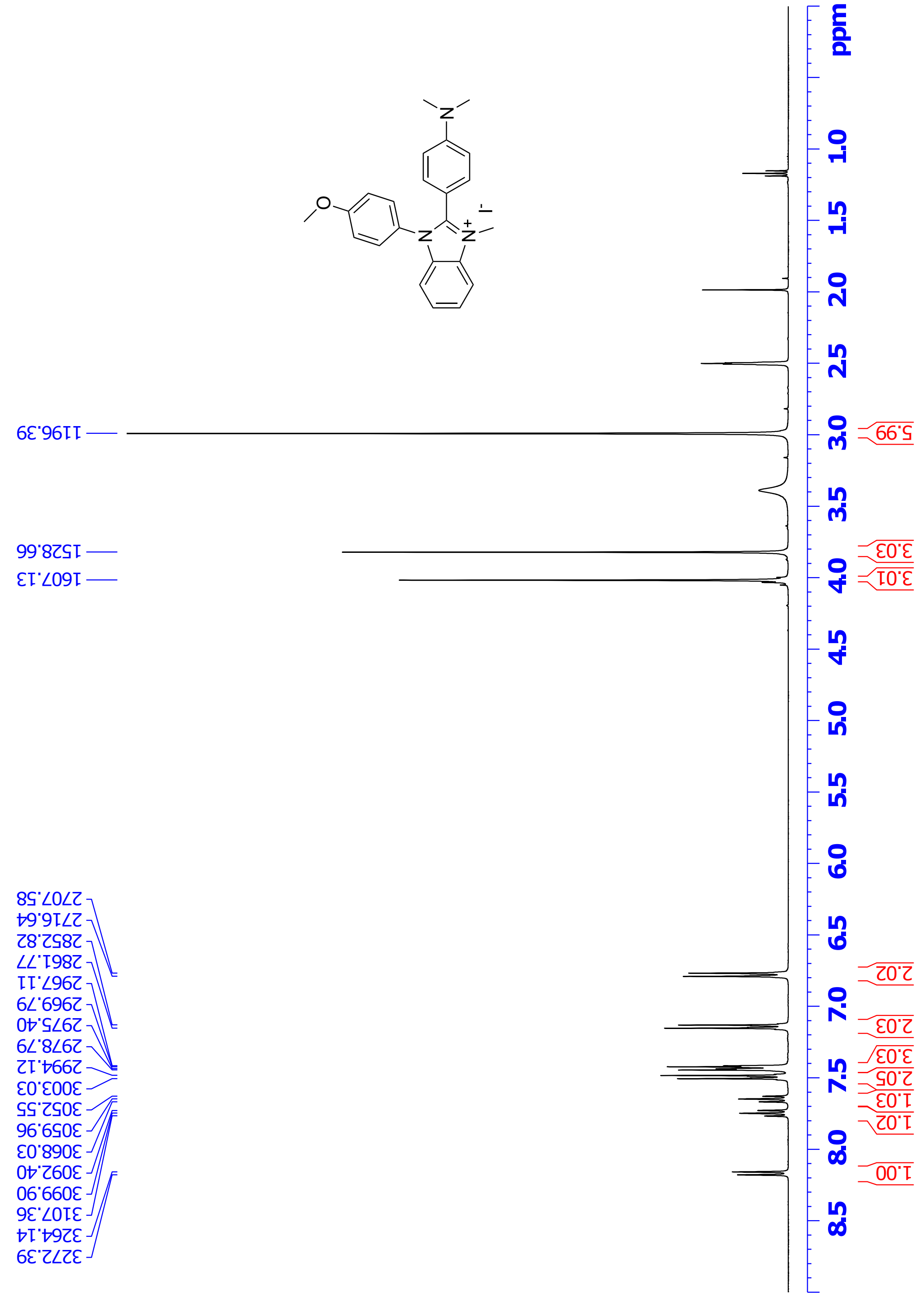

Figure S52: ^{1}H NMR of derivative **8n** in DMSO−d$_6$. Peaks associated to residual ethyl acetate (1.17, 1.99 ppm) are visible in the spectrum.

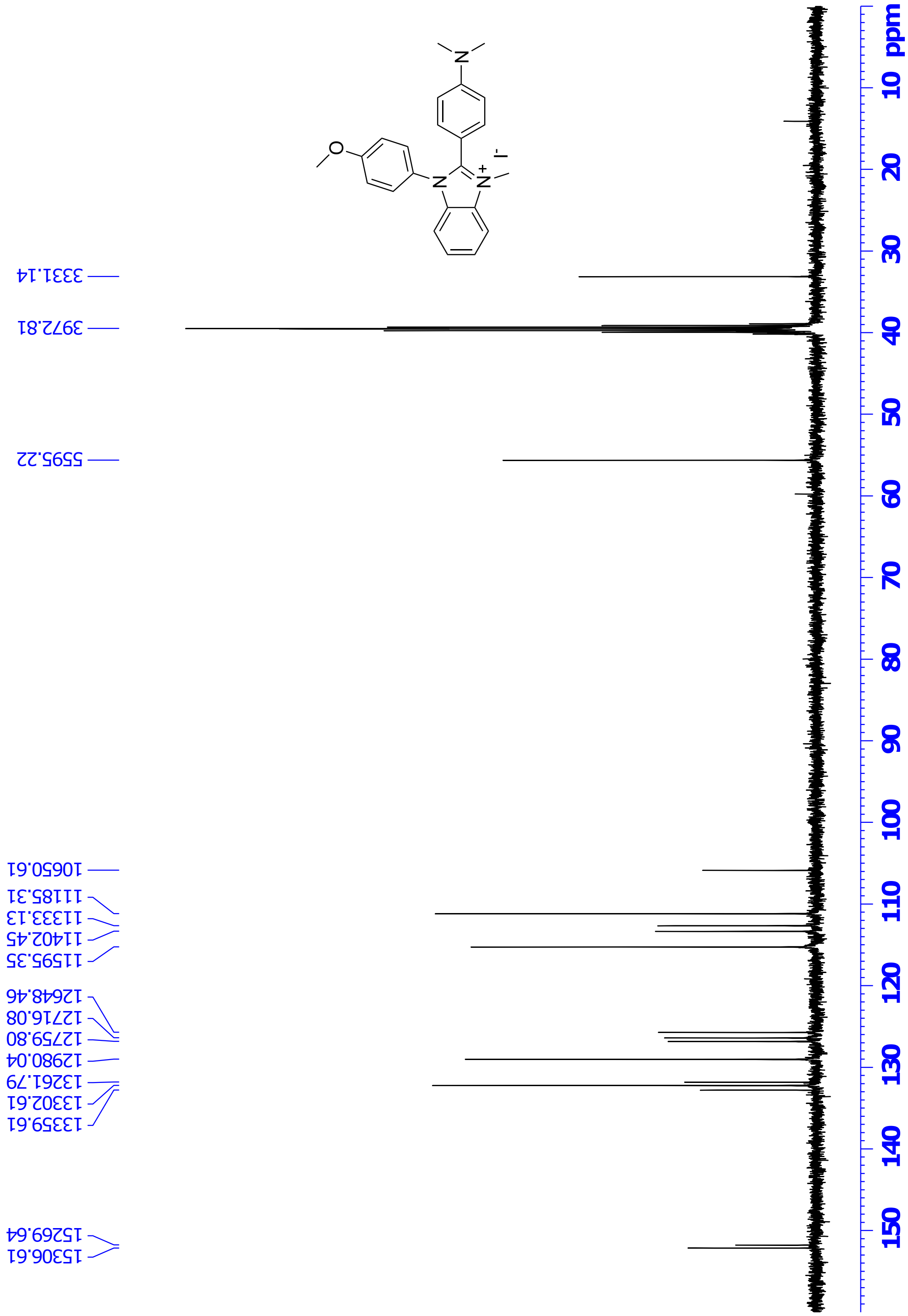

Figure S53: $^{13}C\{^{1}H\}$ NMR of derivative **8n** in DMSO$-d_6$. Peaks associated to residual ethyl acetate (14.40, 59.74 ppm) are visible in the spectrum.

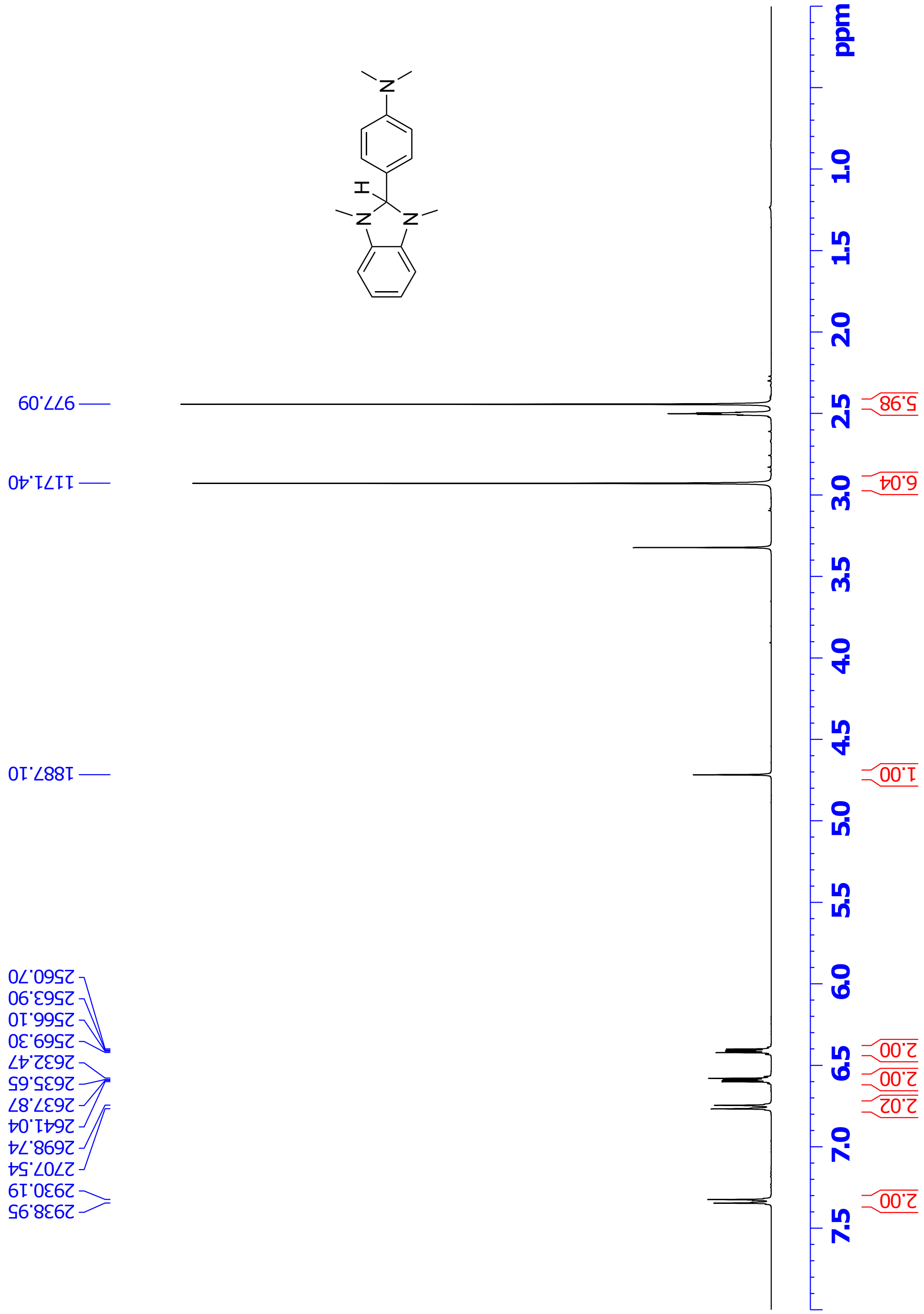

Figure S54: ^{1}H NMR of derivative **5a** in DMSO$-$d$_6$.

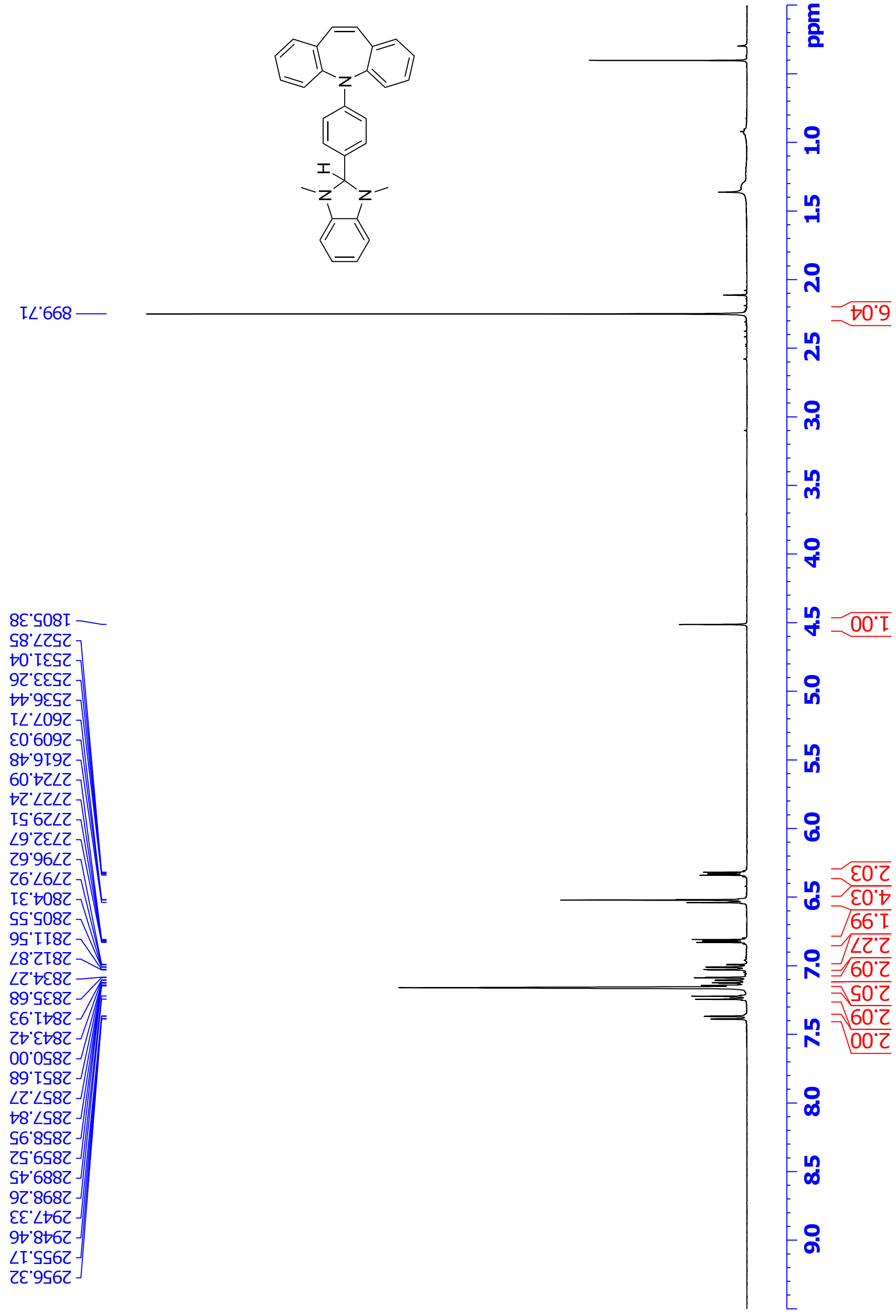

Figure S55: ^{1}H NMR of derivative **5b** in C_6D_6. Peaks visible at 0.29, 0.92 and 1.36 ppm are associated with presence of grease traces in the NMR tube. Peak located at 2.11 ppm is attributed to presence of residual toluene

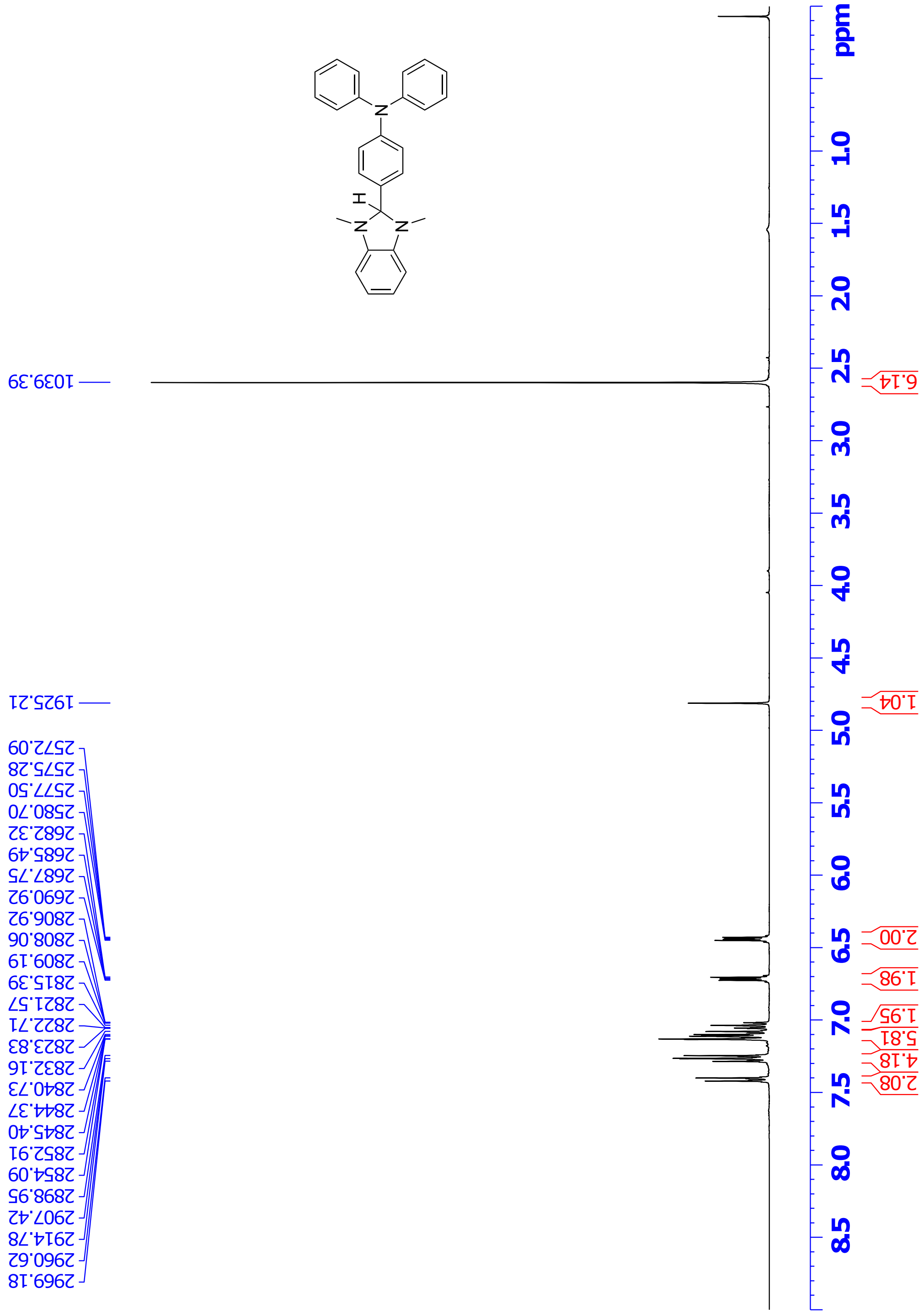

Figure S56: ^{1}H NMR of derivative **5c** in $CDCl_3$.

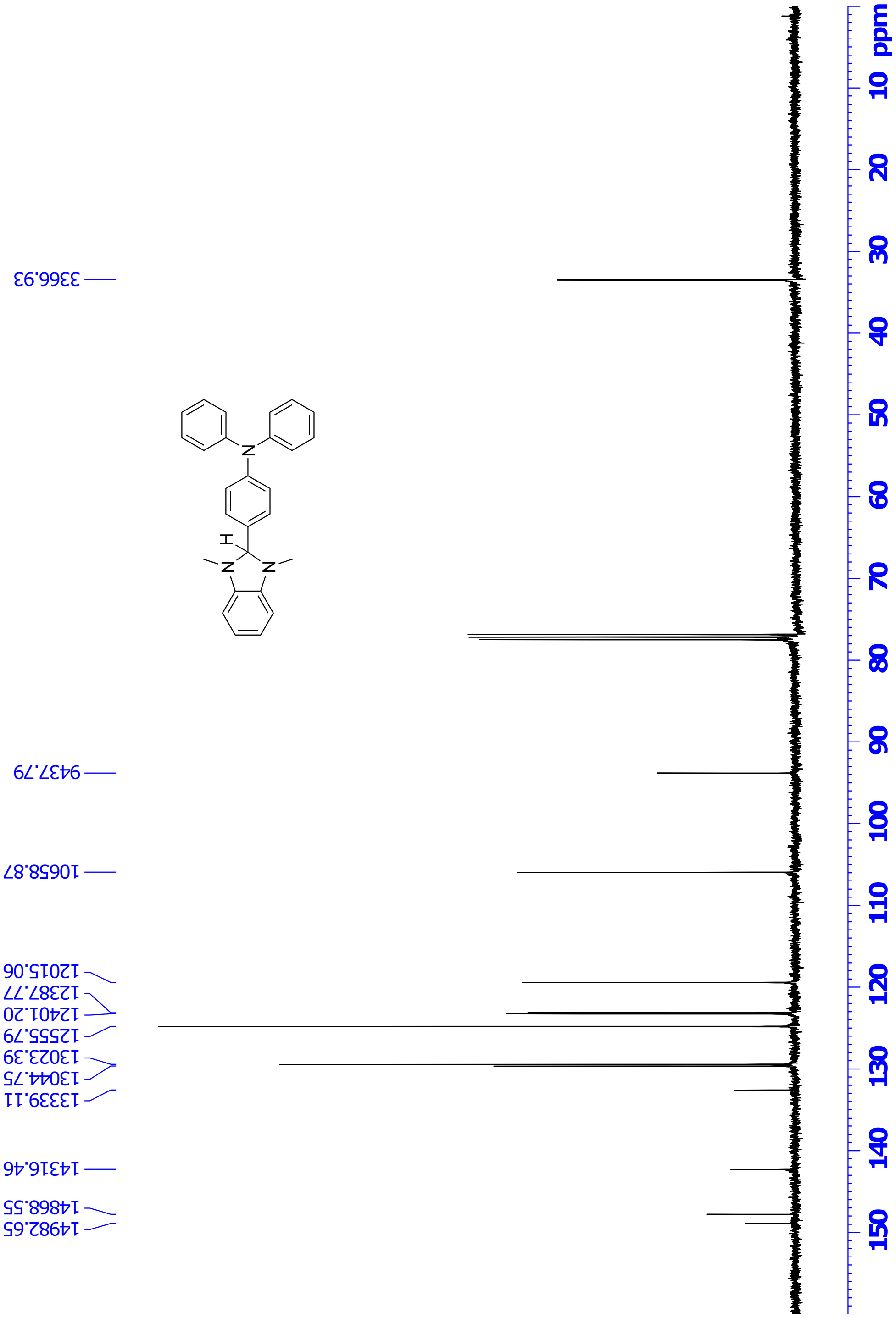

Figure S57: $^{13}C\{^{1}H\}$ NMR of derivative **5c** in $CDCl_3$.

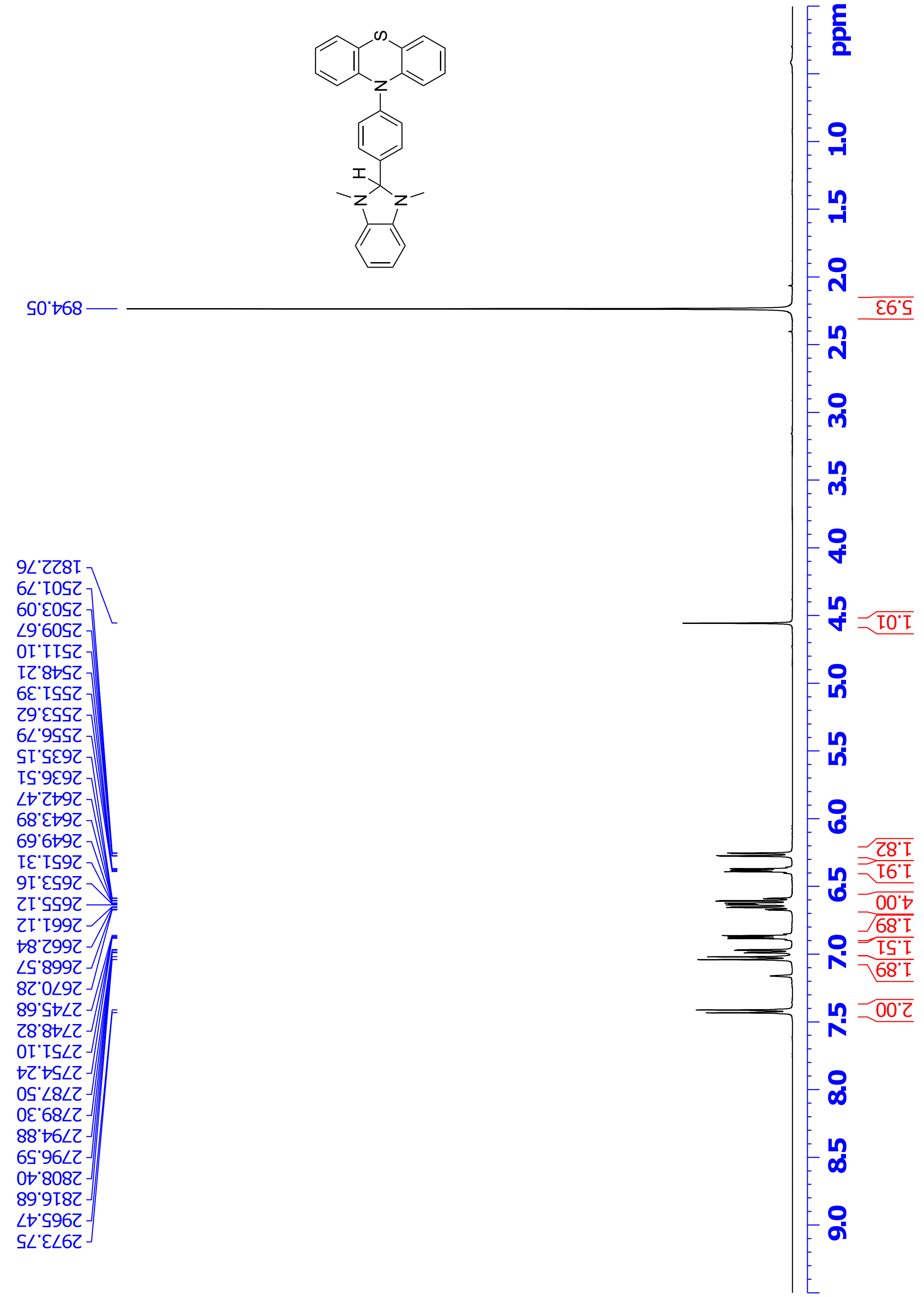

Figure S58: ^{1}H NMR of derivative **5d** in C_6D_6.

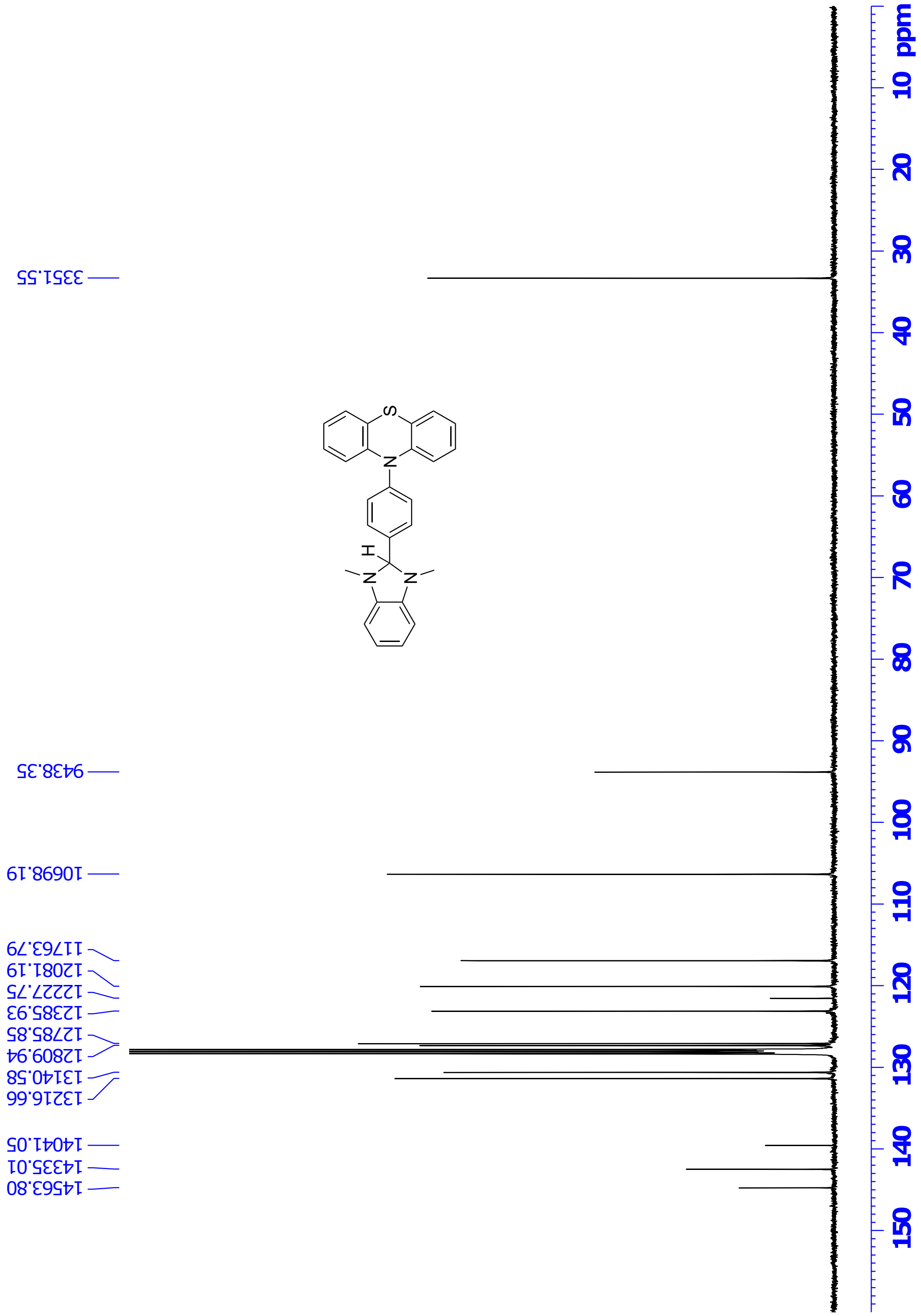

Figure S59: $^{13}C\{^{1}H\}$ NMR of derivative **5d** in C_6D_6.

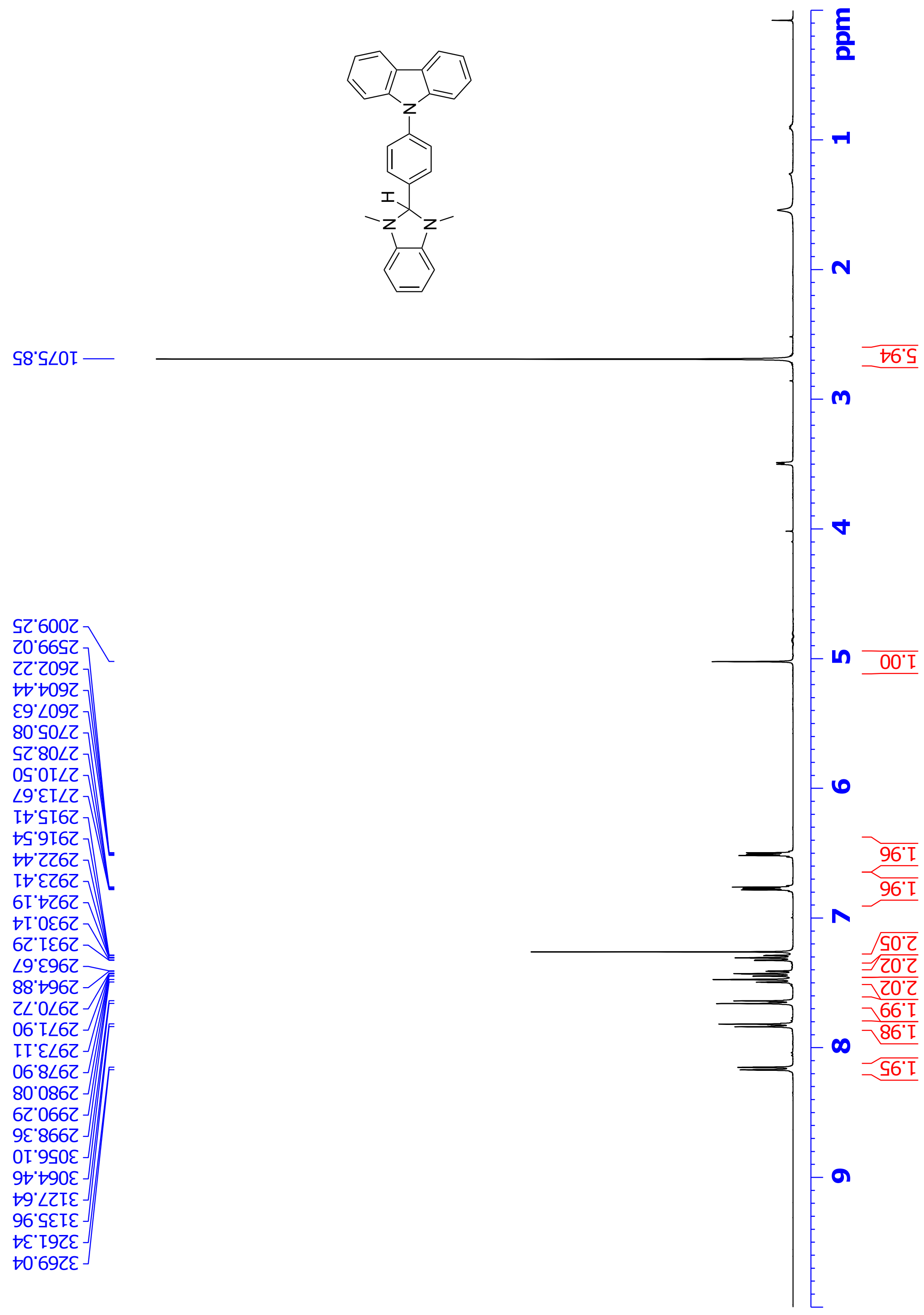

Figure S60: ^{1}H NMR of derivative **5e** in $CDCl_3$.

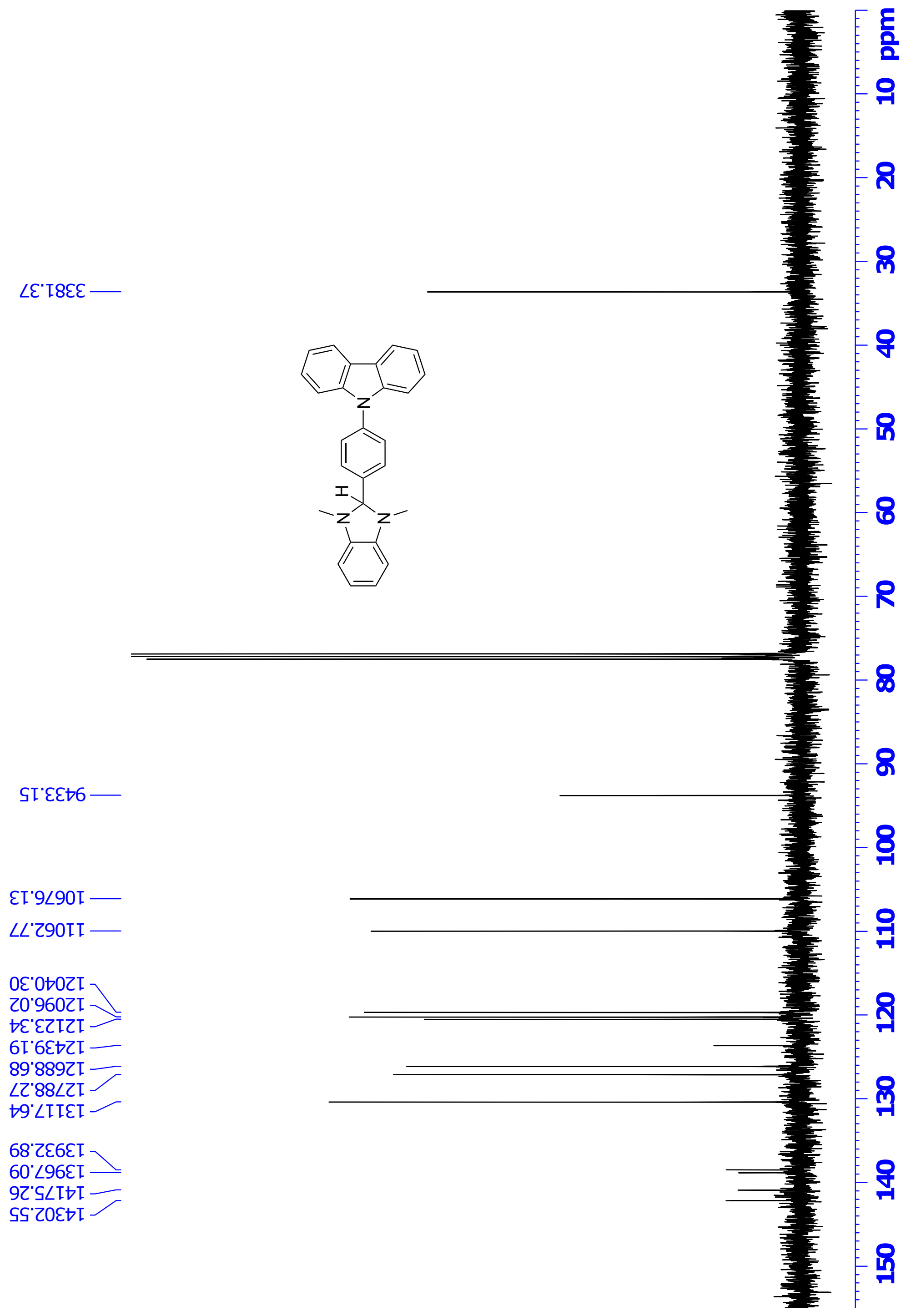

Figure S61: $^{13}C\{^{1}H\}$ NMR of derivative **5e** in $CDCl_3$.

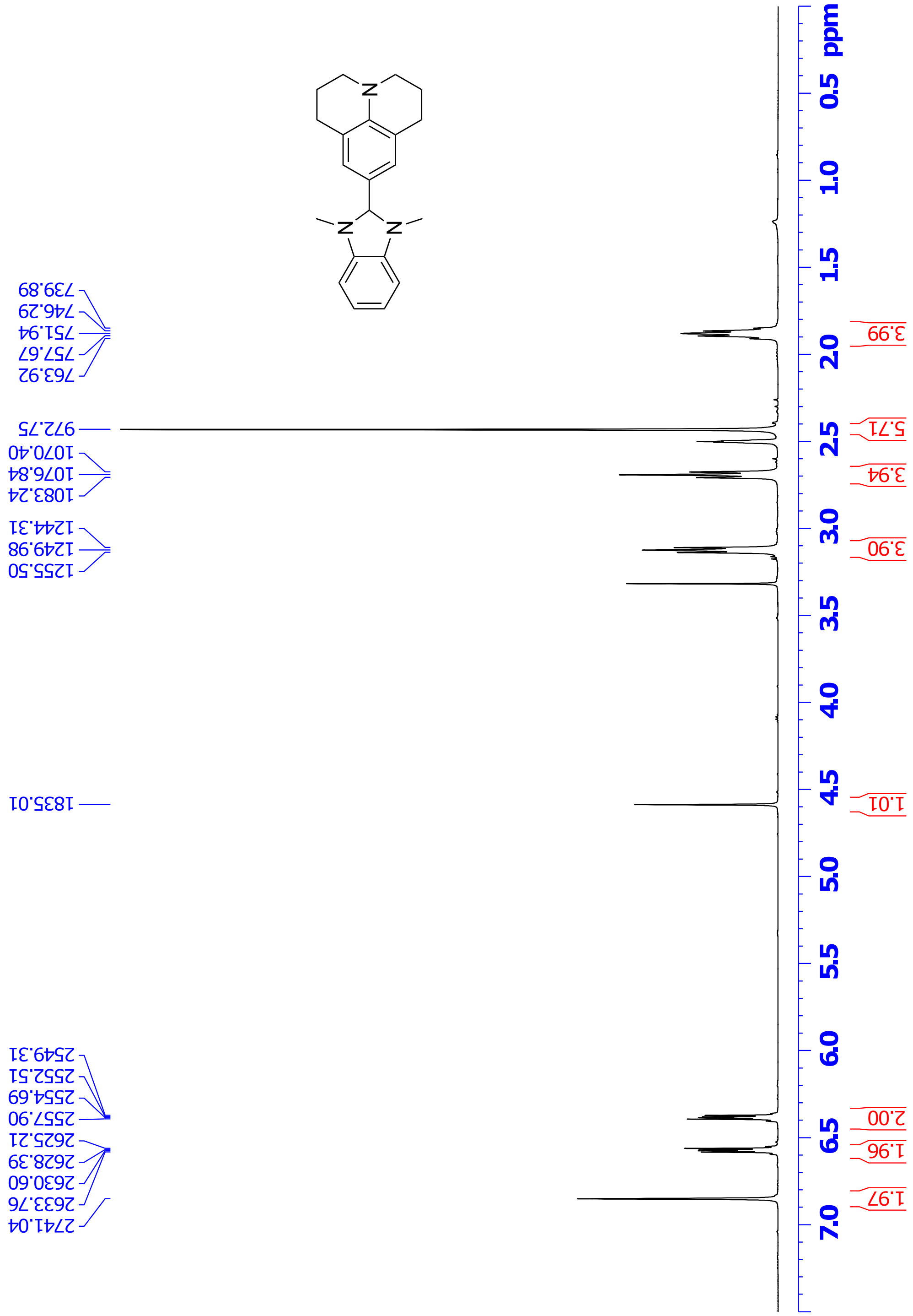

Figure S62: ^{1}H NMR of derivative **5f** in DMSO$-$d$_6$.

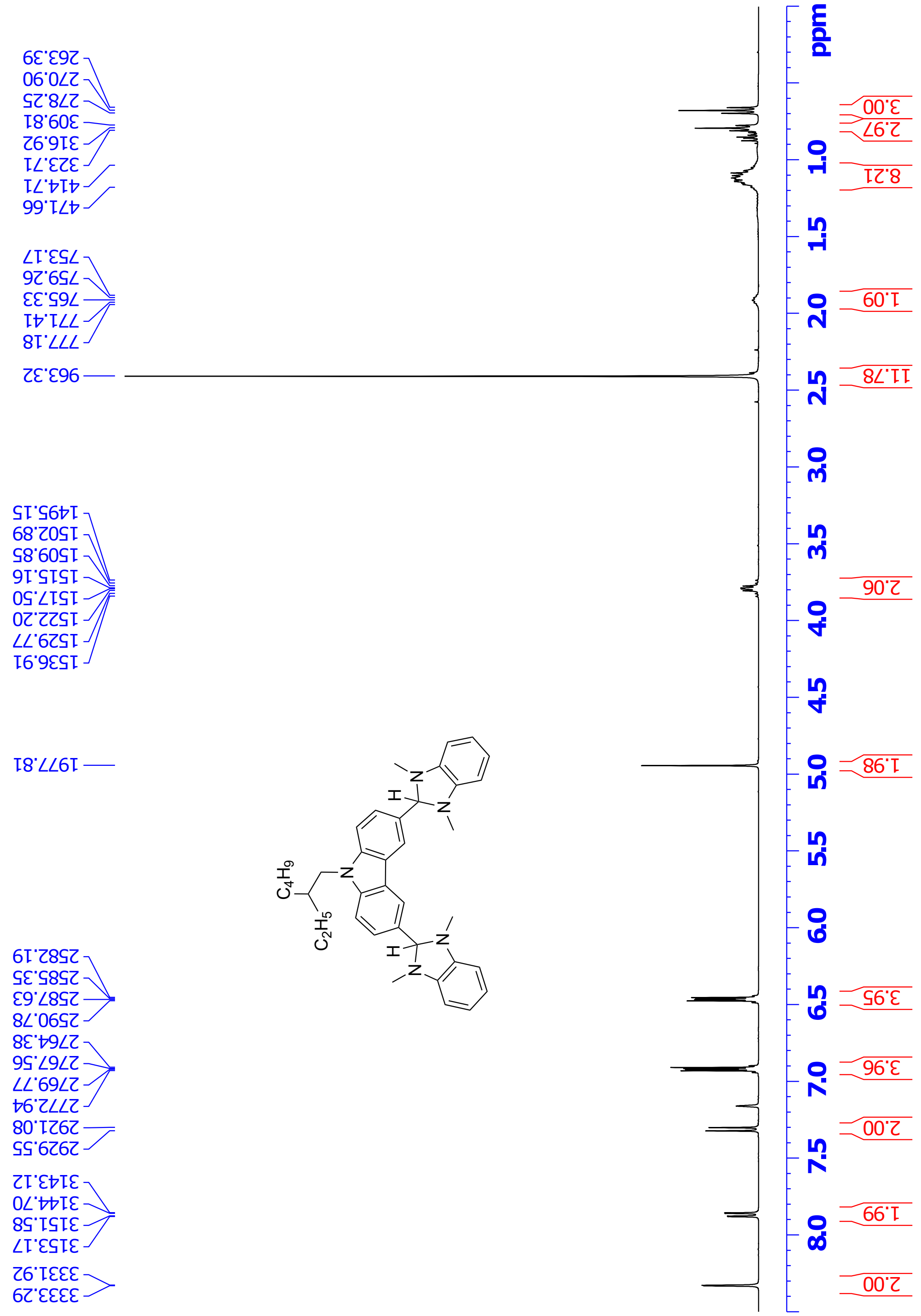

Figure S63: ^{1}H NMR of derivative **5g** in C_6D_6. Multiplet located at 0.89-0.82 ppm is attributed to residual petroleum ether.

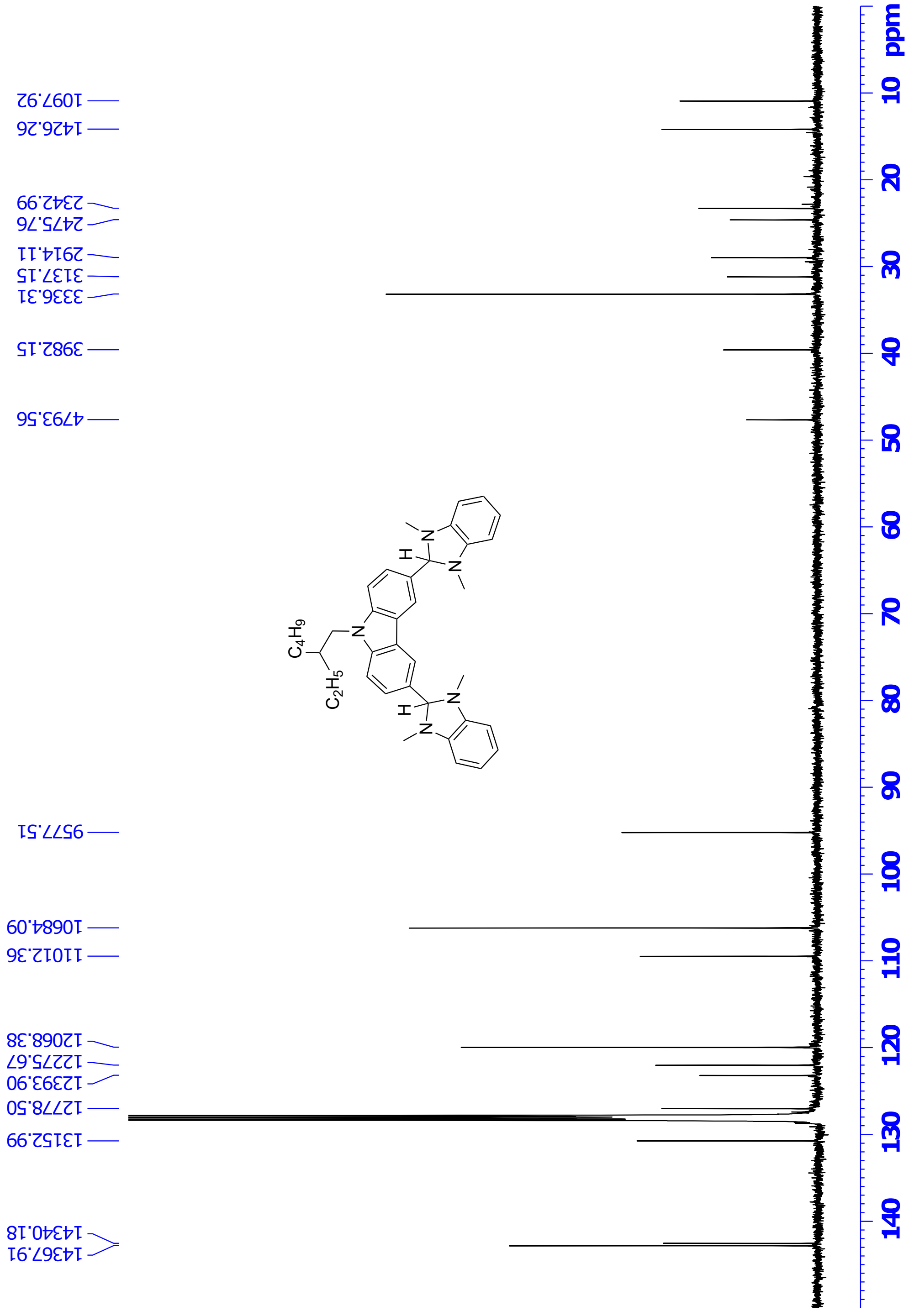

Figure S64: $^{13}C\{^{1}H\}$ NMR of derivative **5g** in C_6D_6.

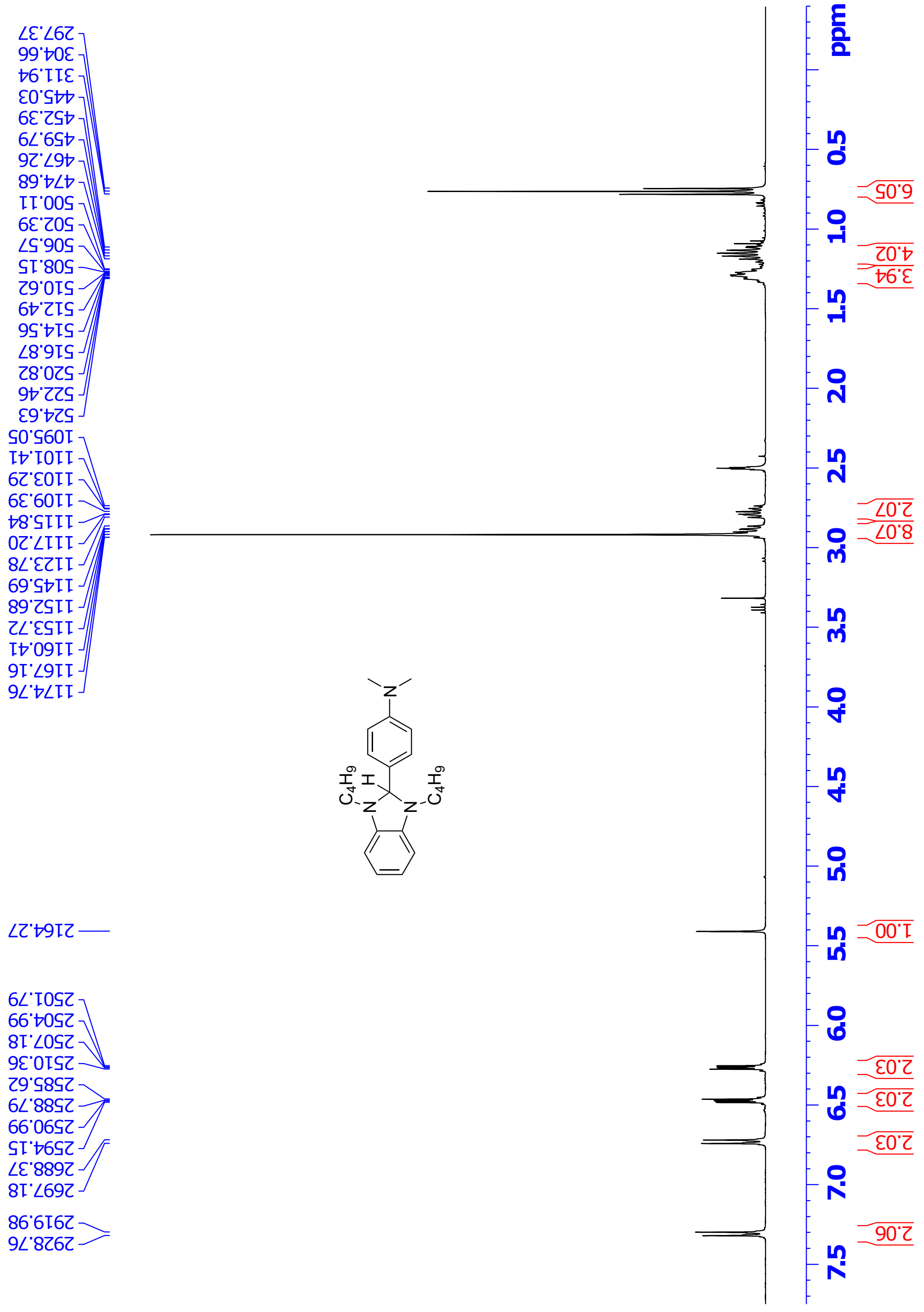

Figure S65: ^{1}H NMR of derivative **5h** in DMSO−d_6.

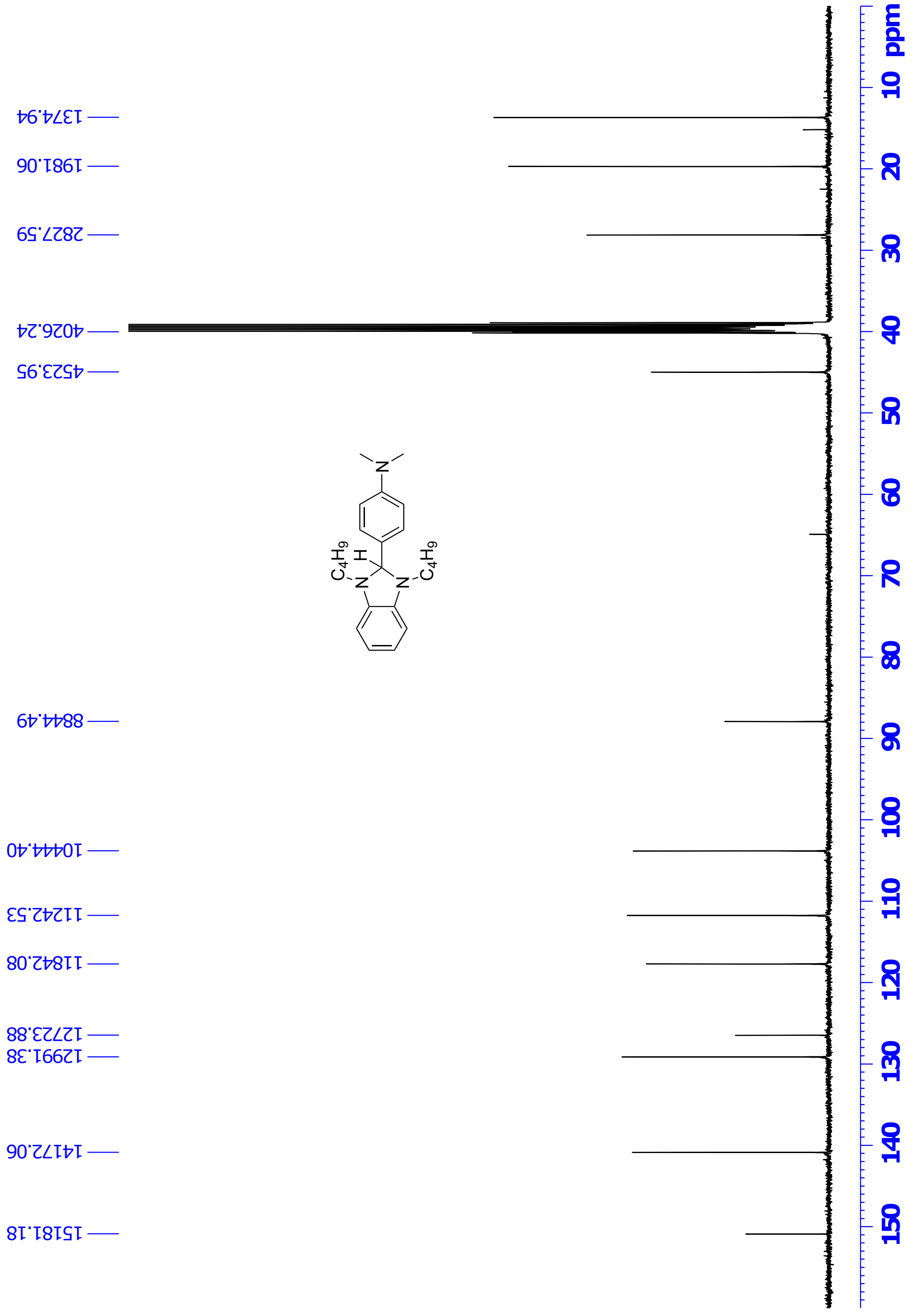

Figure S66: $^{13}C\{^{1}H\}$ NMR of derivative **5h** in DMSO$-d_6$.

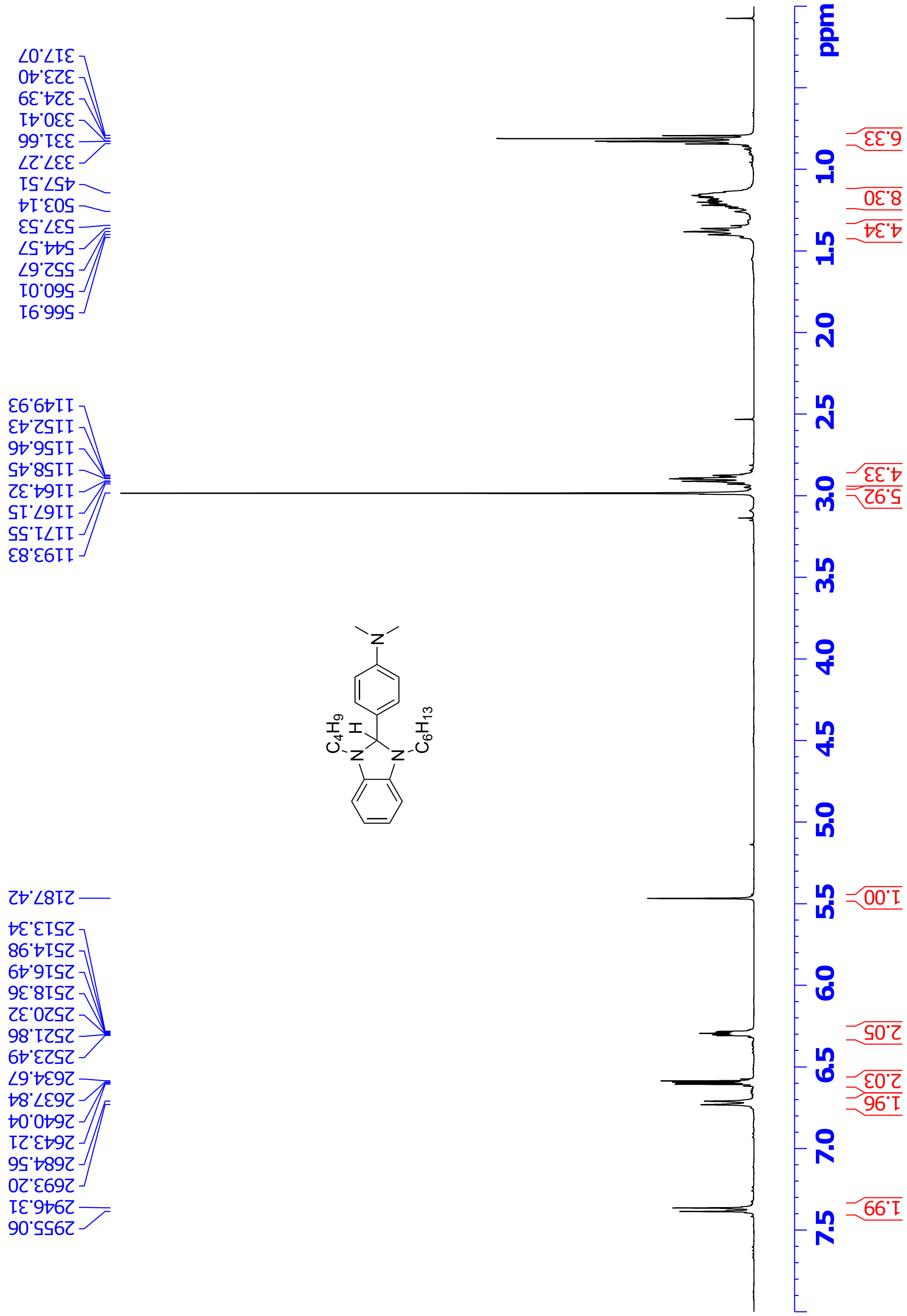

Figure S67: ^{1}H NMR of derivative **5i** in $CDCl_3$.

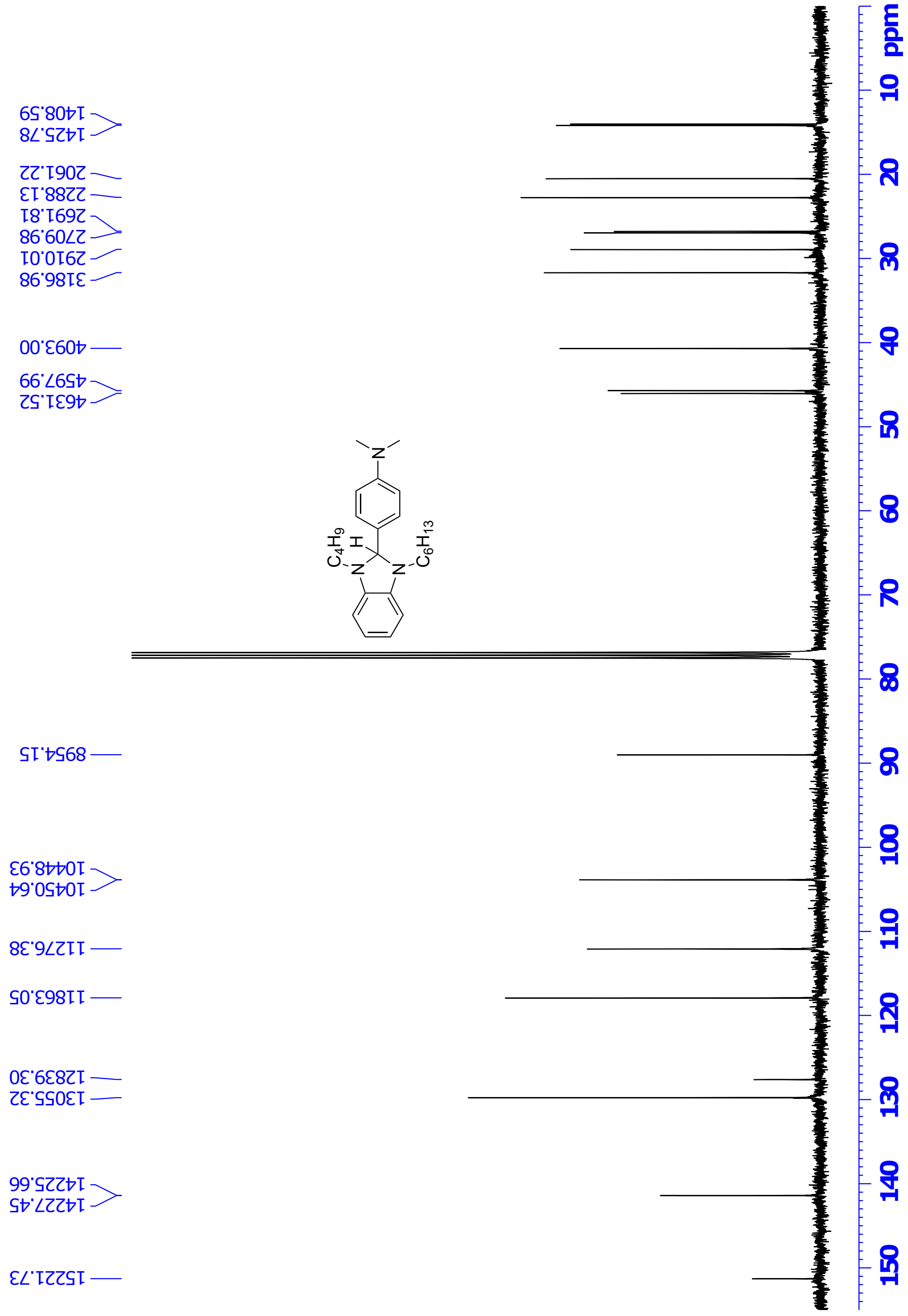

Figure S68: $^{13}C\{^{1}H\}$ NMR of derivative **5i** in $CDCl_3$.

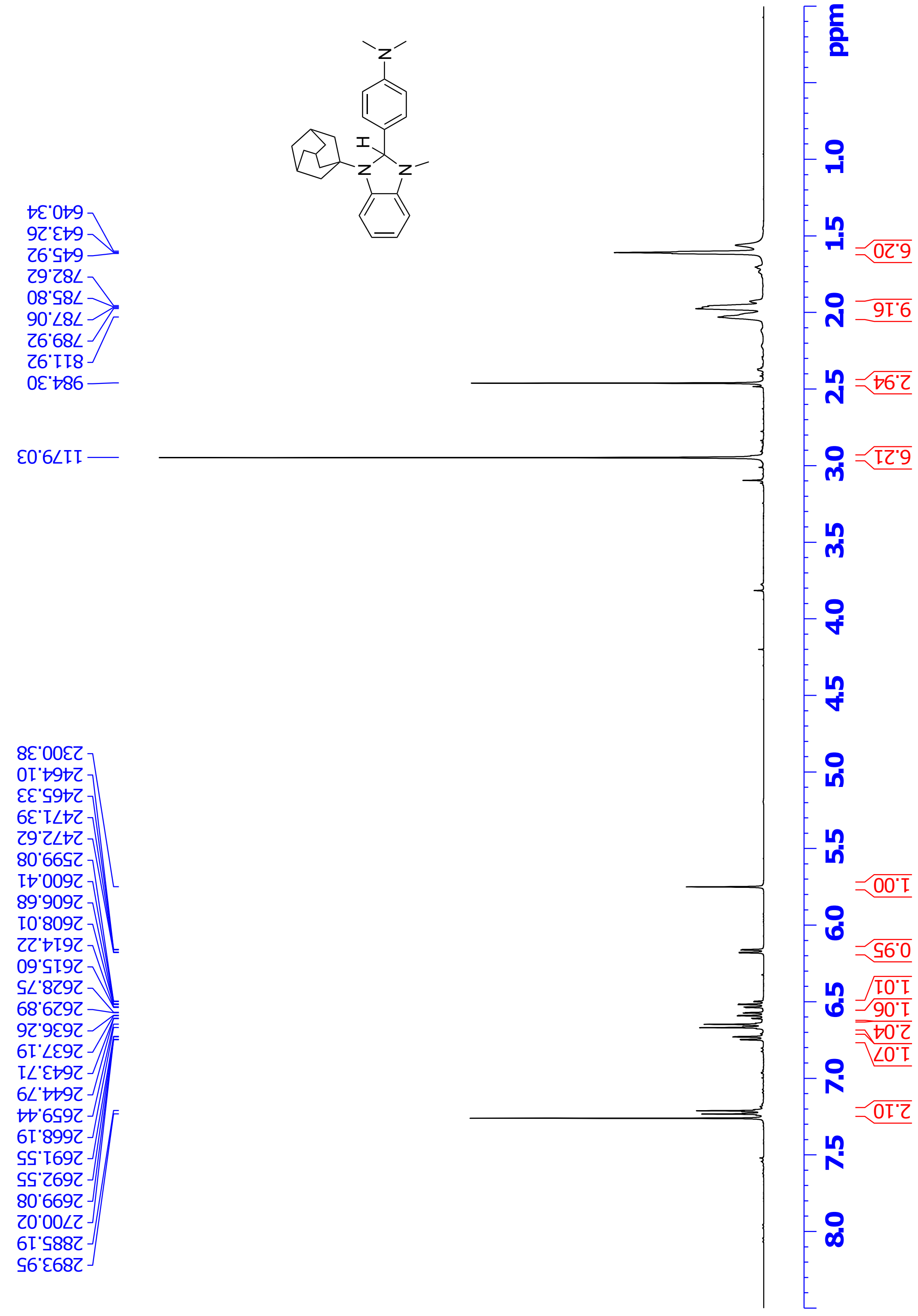

Figure S69: ^{1}H NMR of derivative **5l** in $CDCl_3$.

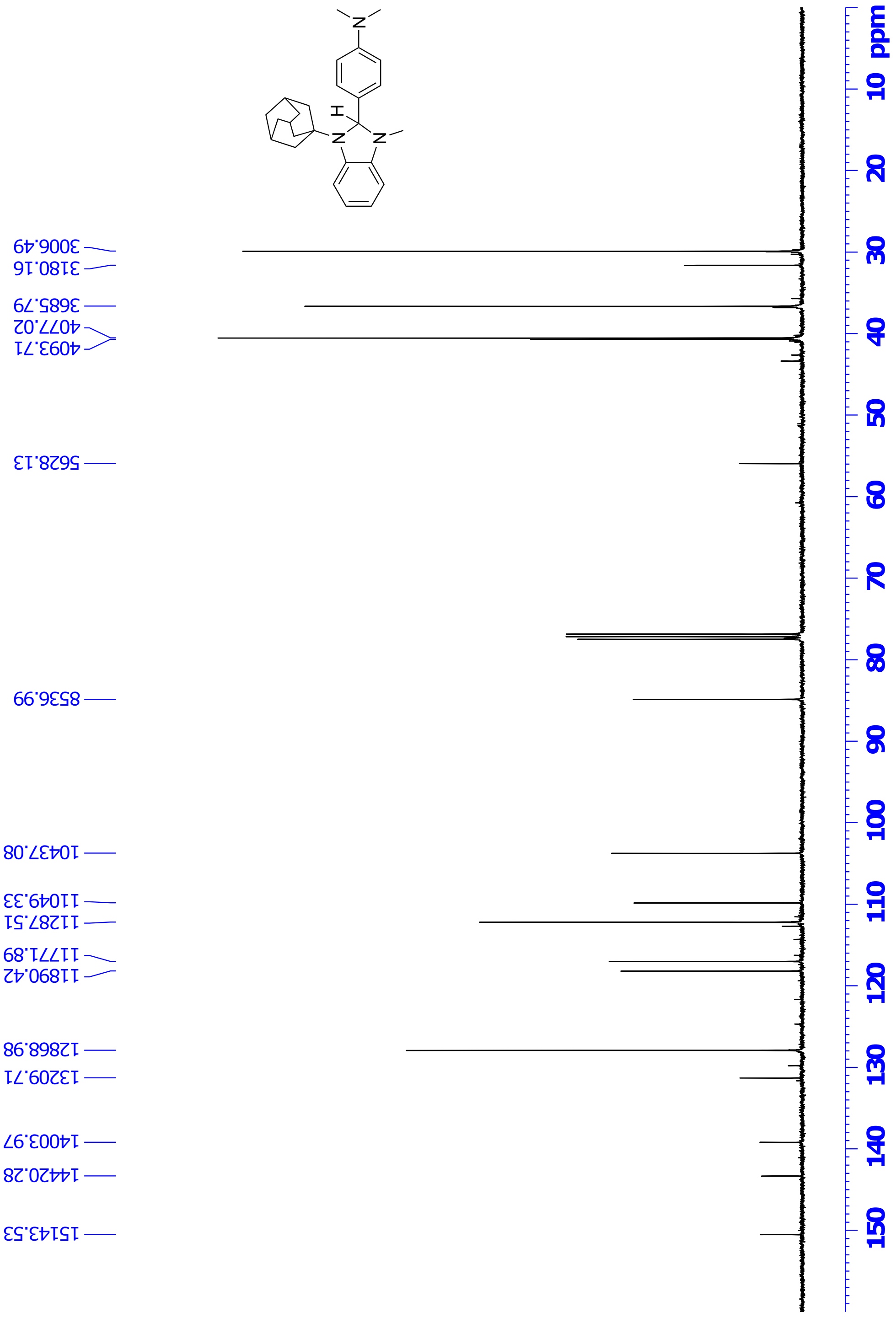

Figure S70: $^{13}C\{^{1}H\}$ NMR of derivative **5l** in $CDCl_3$.

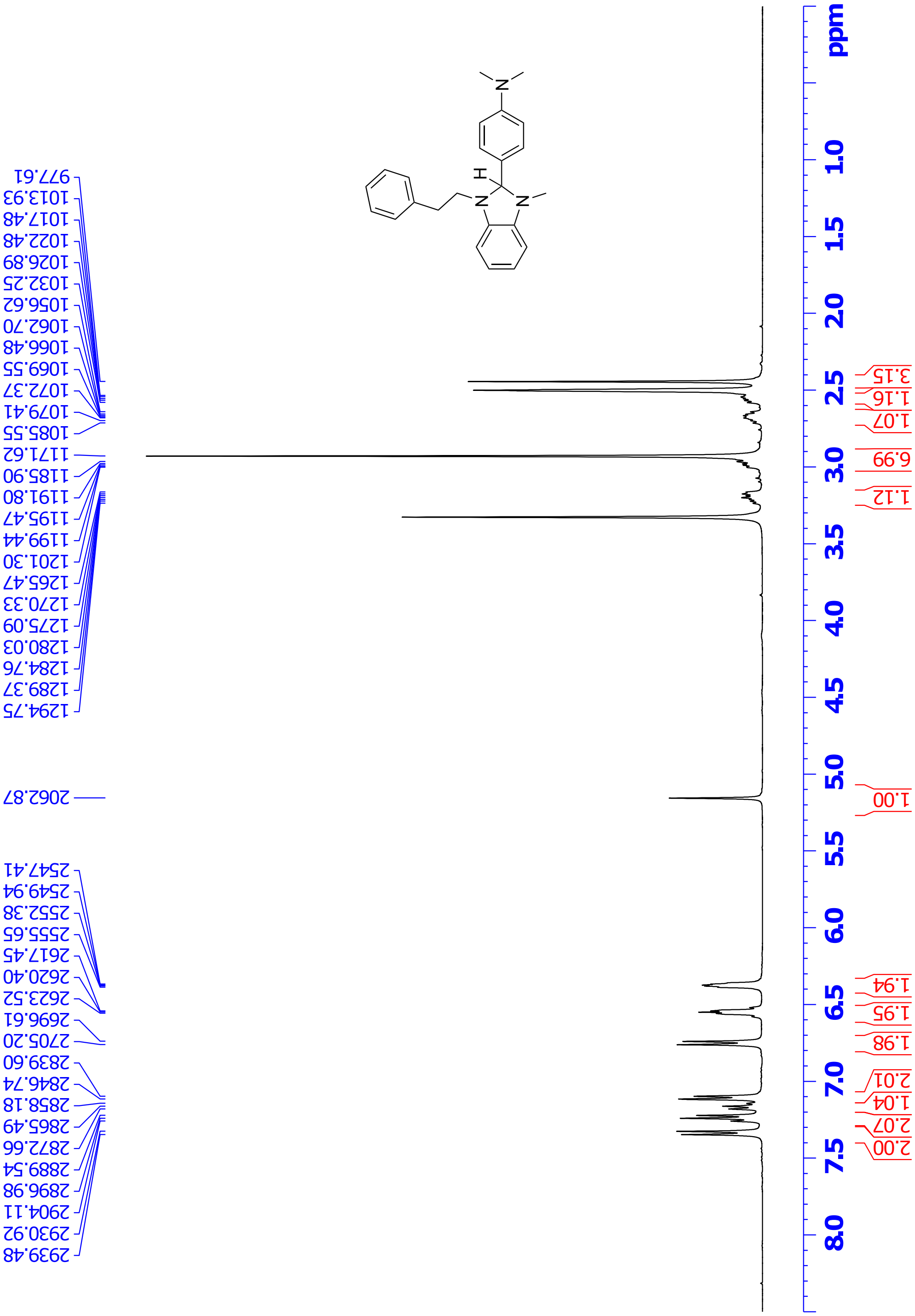

Figure S71: ^{1}H NMR of derivative **5m** in DMSO−d$_6$.

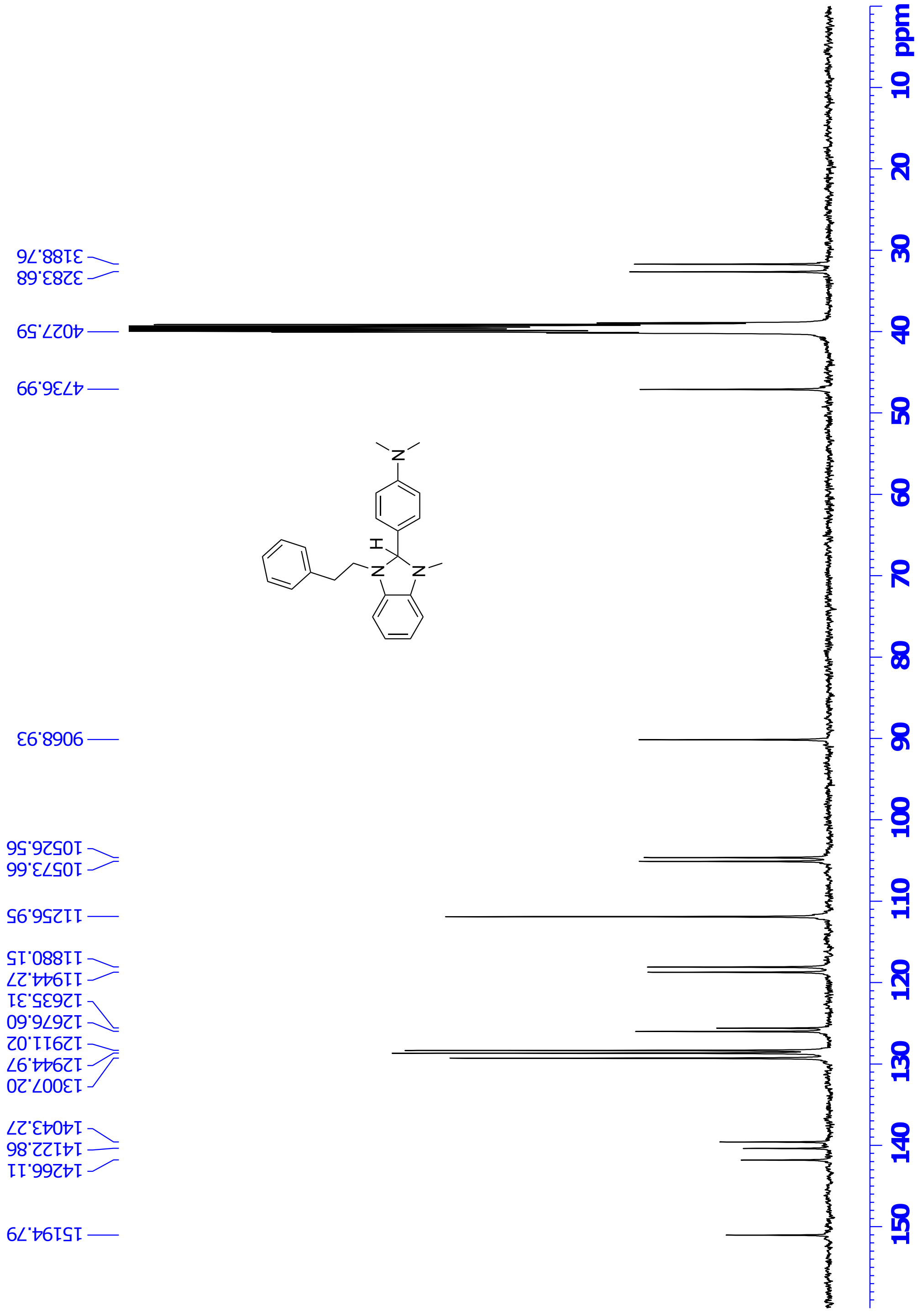

Figure S72: $^{13}C\{^{1}H\}$ NMR of derivative **5m** in DMSO$-d_6$.

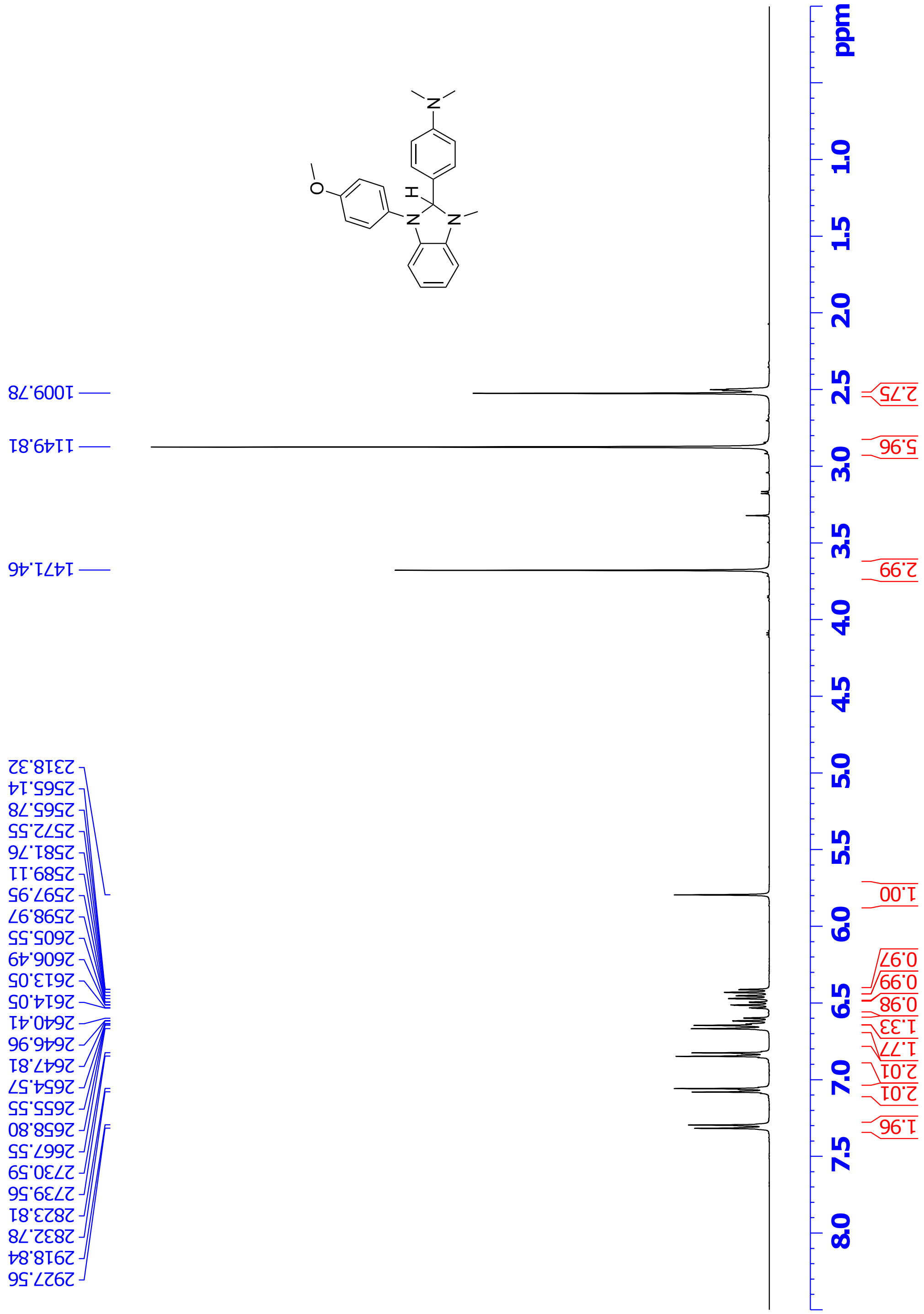

Figure S73: ^{1}H NMR of derivative **5n** in DMSO$-d_6$.

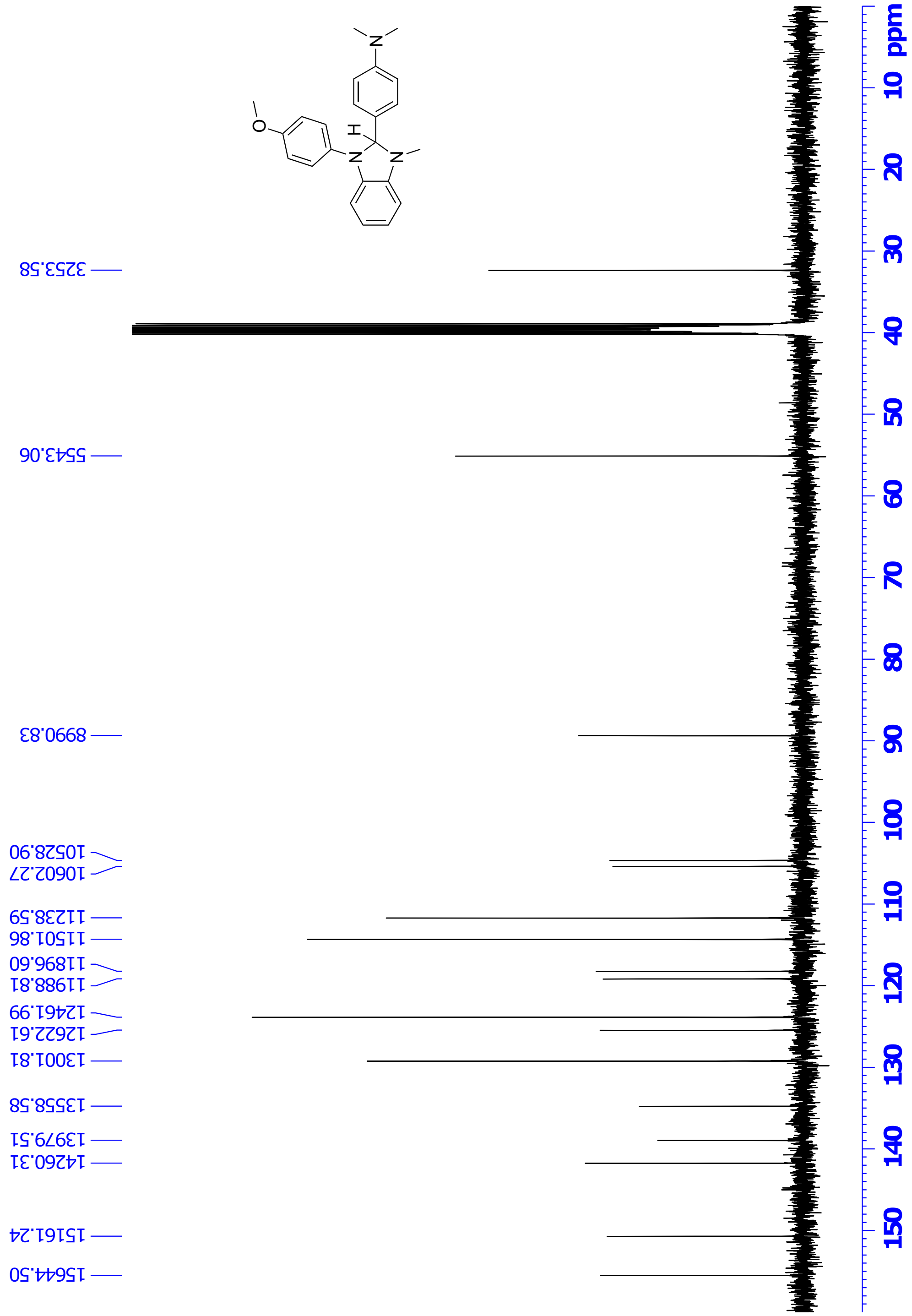

Figure S74: $^{13}C\{^{1}H\}$ NMR of derivative **5e** in DMSO$-d_6$.

References for the Supporting Information

(S1) Bardagot, O.; Aumaître, C.; Monmagnon, A.; Pécaut, J.; Bayle, P.-A.; Demadrille, R. Revisiting doping mechanisms of ntype organic materials with N-DMBI for thermoelectric applications: Photoactivation, thermal activation, and air stability. Applied Physics Letters **2021**, 118.

(S2) Pallini, F.; Sangalli, E.; Sassi, M.; Roth, P. M. C.; Mattiello, S.; Beverina, L. Selective photoredox direct arylations of aryl bromides in water in a microfluidic reactor. Org. Biomol. Chem. **2021**, 19, 3016–3023.

(S3) Rossi, P.; Pallini, F.; Coco, G.; Mattiello, S.; Tan, W. L.; Mezzomo, L.; Cassinelli, M.; Lanzani, G.; McNeill, C. R.; Beverina, L.; Caironi, M. An Iminostilbene Functionalized Benzimidazoline for Enhanced n-Type Solution Doping of Semiconducting Polymers for Organic Thermoelectrics. Advanced Materials Interfaces **2023**, 10.

(S4) von der Heiden, D.; Detmar, E.; Kuchta, R.; Breugst, M. Activation of Michael acceptors by halogen-bond donors. Synlett **2018**, 29, 1307–1313.

(S5) Keita, H. Synthesis and thermal characterization of solid state organic electrolytes for their potential lithium ion battery applications. Materials Letters: X **2021**, 12, 100093.

(S6) Mingqin Huang, J.-J. L.; Zhang, C. Halogen-Bonding-Mediated Synthesis of Amides and Peptides. Green Chemistry **2023**, 25, 9187–9193.

(S7) Astakhov, A. V.; Khazipov, O. V.; Degtyareva, E. S.; Khrustalev, V. N.; Chernyshev, V. M.; Ananikov, V. P. Facile Hydrolysis of Nickel(II) Complexes with N-Heterocyclic Carbene Ligands. Organometallics **2015**, 34, 5759–5766.

(S8) Lu, H.; Brutchey, R. L. Tunable Room-Temperature Synthesis of Coinage Metal

Chalcogenide Nanocrystals from N-Heterocyclic Carbene Synthons. Chemistry of Materials **2017**, 29, 1396–1403.

(S9) Wisser, F. M.; Duguet, M.; Perrinet, Q.; Ghosh, A. C.; Alves-Favaro, M.; Mohr, Y.; Lorentz, C.; Quadrelli, E. A.; Palkovits, R.; Farrusseng, D.; Mellot-Draznieks, C.; de Waele, V.; Canivet, J. Molecular Porous Photosystems Tailored for Long-Term Photocatalytic CO_2 Reduction. Angewandte Chemie International Edition **2020**, 59, 5116–5122.

(S10) Thapa, P.; Palacios, P. M.; Tran, T.; Pierce, B. S.; Foss, F. W. J. 1,2-Disubstituted Benzimidazoles by the Iron Catalyzed Cross-Dehydrogenative Coupling of Isomeric o-Phenylenediamine Substrates. The Journal of Organic Chemistry **2020**, 85, 1991–2009.

(S11) Mattiello, S.; Lucarelli, G.; Calascibetta, A.; Polastri, L.; Ghiglietti, E.; Podapangi, S. K.; Brown, T. M.; Sassi, M.; Beverina, L. Sustainable, Efficient, and Scalable Preparation of Pure and Performing Spiro-OMeTAD for Perovskite Solar Cells. ACS Sustainable Chemistry & Engineering **2022**, 10, 4750–4757.

(S12) Huang, W.; Buchwald, S. L. Palladium-Catalyzed N-Arylation of Iminodibenzyls and Iminostilbenes with Aryl- and Heteroaryl Halides. Chemistry - A European Journal **2016**, 22, 14186–14189.

(S13) Fan, M.; Cheng, Y.; Fang, B.; Lai, L.; Yin, M. Multicolor mechanochromism of a phenothiazine derivative through molecular interaction and conformational modulations. Dyes and Pigments **2021**, 190, 109311.

(S14) Wang, Y.; Lai, G.; Li, Z.; Ma, Y.; Shen, Y.; Wang, C. Novel A-π-D-π-A type molecules based on diphenylamine and carbazole with large two-photon absorption cross section and excellent aggregation-induced enhanced emission property. Tetrahedron **2015**, 71, 2761–2767.

(S15) Wang, H.; Ryu, J.-T.; Kwon, Y. Synthesis of oxadiazole-based polymers containing a carbazole-vinylene or fluorene-vinylene group and their hole-injection/transport behavior in light-emitting diodes. Journal of Applied Polymer Science **2011**, 119, 377–386.

(S16) Akhrem, I. S.; Avetisyan, D. V.; Kagramanov, N. D.; Petrovskii, P. V.; Mysova, N. E. One-pot N-acylation and N-alkylation of o-nitroaniline with saturated hydrocarbons in the presence of carbon monoxide. Mendeleev Communications **2010**, 20, 257–259.

(S17) Rao, H.; Fu, H.; Jiang, Y.; Zhao, Y. Copper-Catalyzed Arylation of Amines Using Diphenyl Pyrrolidine-2-phosphonate as the New Ligand. The Journal of Organic Chemistry **2005**, 70, 8107–8109.

(S18) Wang, T.-H.; Ambre, R.; Wang, Q.; Lee, W.-C.; Wang, P.-C.; Liu, Y.; Zhao, L.; Ong, T.-G. Nickel-Catalyzed Heteroarenes Cross Coupling via Tandem C-H/C-O Activation. ACS Catalysis **2018**, 8, 11368–11376.

(S19) Zhou, Q.; Liu, S.; Ma, M.; Cui, H. Z.; Hong, X.; Huang, S.; Zhang, J. F.; Hou, X. F. N-Heterocyclic Carbene (NHC)-Catalyzed One-Pot Aerobic Oxidative Synthesis of 2-Substituted Benzo[d]oxazoles, Benzo[d]thiazoles and 1,2-Disubstituted Benzo[d]imidazoles. Synthesis (Germany) **2018**, 50, 1315–1322.

(S20) Riera-Galindo, S.; Orbelli Biroli, A.; Forni, A.; Puttisong, Y.; Tessore, F.; Pizzotti, M.; Pavlopoulou, E.; Solano, E.; Wang, S.; Wang, G.; Ruoko, T.-P.; Chen, W. M.; Kemerink, M.; Berggren, M.; di Carlo, G.; Fabiano, S. Impact of Singly Occupied Molecular Orbital Energy on the n-Doping Efficiency of Benzimidazole Derivatives. ACS Applied Materials & Interfaces **2019**, 11, 37981–37990.

(S21) Miyajima, R.; Ooe, Y.; Miura, T.; Ikoma, T.; Iwamoto, H.; Takizawa, S.-y.; Hasegawa, E. Correction to "Triarylamine-Substituted Benzimidazoliums as Electron

Donor–Acceptor Dyad-Type Photocatalysts for Reductive Organic Transformations". Journal of the American Chemical Society **2023**, 145, 16284–16284.

(S22) Li, C.; Wang, W.; Zhan, C.; Zhou, Q.; Dong, D.; Xiao, S. A julolidine functionalized benzimidazoline n-dopant: optimizing molecular doping in fullerene derivatives by modulating miscibility. Journal of Materials Chemistry C **2023**, 11, 15599–15607.

(S23) Sawyer, D. T.; Sobkowiak, A.; Roberts, J. L. Electrochemistry for Chemists, 2nd ed.; Wiley, 1995.

(S24) Pracht, P.; Bohle, F.; Grimme, S. Automated exploration of the low-energy chemical space with fast quantum chemical methods. Phys. Chem. Chem. Phys. **2020**, 22, 7169–7192.

(S25) Bannwarth, C.; Ehlert, S.; Grimme, S. GFN2-xTB—An Accurate and Broadly Parametrized Self-Consistent Tight-Binding Quantum Chemical Method with Multipole Electrostatics and Density-Dependent Dispersion Contributions. Journal of Chemical Theory and Computation **2019**, 15, 1652–1671.

(S26) Neese, F. Software update: the ORCA program system, version 4.0. WIREs Computational Molecular Science **2018**, 8, e1327.

(S27) Neese, F. The ORCA program system. WIREs Computational Molecular Science **2012**, 2, 73–78.

(S28) Weigend, F.; Ahlrichs, R. Balanced basis sets of split valence, triple zeta valence and quadruple zeta valence quality for H to Rn: Design and assessment of accuracy. Phys. Chem. Chem. Phys. **2005**, 7, 3297–3305.

(S29) Schäfer, A.; Horn, H.; Ahlrichs, R. Fully optimized contracted Gaussian basis sets for atoms Li to Kr. The Journal of Chemical Physics **1992**, 97, 2571–2577.

(S30) Becke, A. D. Density-functional thermochemistry. III. The role of exact exchange. The Journal of Chemical Physics **1993**, 98, 5648–5652.

(S31) Grimme, S.; Antony, J.; Ehrlich, S.; Krieg, H. A consistent and accurate ab initio parametrization of density functional dispersion correction (DFT-D) for the 94 elements H-Pu. The Journal of Chemical Physics **2010**, 132, 154104.

(S32) Zhao, Y.; Truhlar, D. G. The M06 suite of density functionals for main group thermochemistry, thermochemical kinetics, noncovalent interactions, excited states, and transition elements: two new functionals and systematic testing of four M06-class functionals and 12 other functionals. Theoretical Chemistry Accounts **2008**, 120, 215–241.

(S33) Barone, V.; Cossi, M. Quantum Calculation of Molecular Energies and Energy Gradients in Solution by a Conductor Solvent Model. The Journal of Physical Chemistry A **1998**, 102, 1995–2001.
